# Authenticating coins of the ‘Roman emperor’ Sponsian

**DOI:** 10.1371/journal.pone.0274285

**Published:** 2022-11-23

**Authors:** Paul N. Pearson, Michela Botticelli, Jesper Ericsson, Jacek Olender, Liene Spruženiece

**Affiliations:** 1 Department of Earth Sciences, University College London, London, United Kingdom; 2 Kelvin Centre for Conservation and Cultural Heritage Research, School of Culture and Creative Arts, University of Glasgow, Glasgow, United Kingdom; 3 Curator of Numismatics, The Hunterian, University of Glasgow, Glasgow, United Kingdom; 4 School of Geographical and Earth Sciences, University of Glasgow, Glasgow, United Kingdom; Sapienza University of Rome: Universita degli Studi di Roma La Sapienza, ITALY

## Abstract

The ‘Roman emperor’ Sponsian is known only from an assemblage of coins allegedly found in Transylvania (Romania) in 1713. They are very unlike regular Roman coins in style and manufacture, with various enigmatic features including bungled legends and historically mixed motifs, and have long been dismissed as poorly made forgeries. Here we present non-destructive imaging and spectroscopic results that show features indicative of authenticity. Deep micro-abrasion patterns suggest extensive circulation-wear. Superficial patches of soil minerals bound by authigenic cement and overlain by oxidation products indicate a history of prolonged burial then exhumation. These observations force a re-evaluation of Sponsian as a historical personage. Combining evidence from the coins with the historical record, we suggest he was most likely an army commander in the isolated Roman Province of Dacia during the military crisis of the 260s CE, and that his crudely manufactured coins supported a functioning monetary economy that persisted locally for an appreciable period.

## Introduction

**Fig 1:**
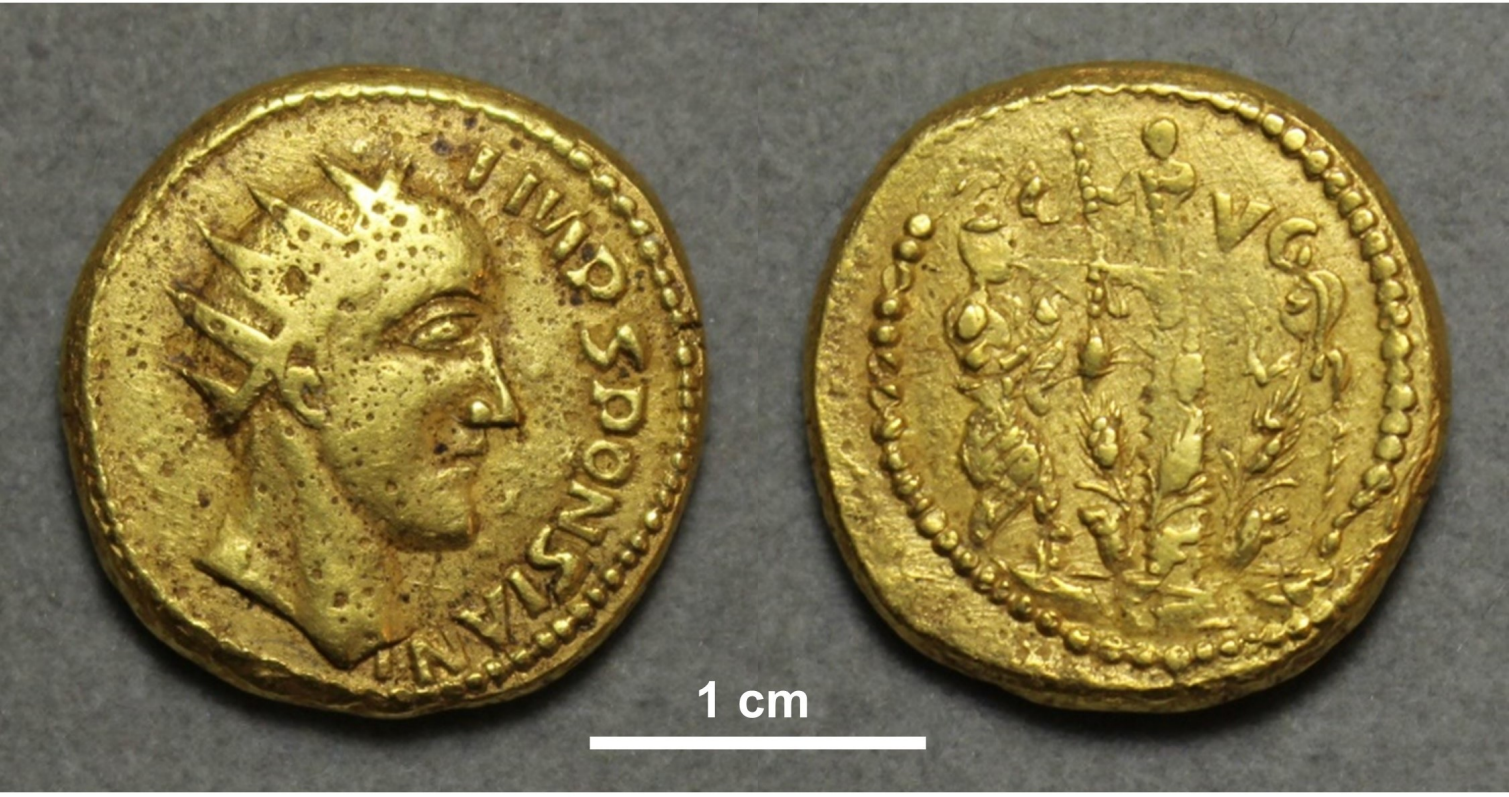
Coin of the ‘emperor’ Sponsian, currently in The Hunterian, University of Glasgow, UK, catalogue number GLAHM:40333. Reproduced from Ref. [1].

Evidence surrounding the possible historicity of the long disputed ‘emperor’ Sponsian was recently reviewed as part of a study of Rome’s third century crisis [1]. That work included publication of the first high-resolution colour photograph of a Sponsian coin from the collection of The Hunterian, University of Glasgow (reproduced in Fig 1). Although this coin and others associated with it have long been regarded as eighteenth century fakes, we were surprised to see apparent superficial wear scratches and ‘earthen deposits’ (adhering matrix) that seemed to warrant further investigation [1]. Historical forgers are known to have used artificial ageing methods, including abrasion to simulate wear and staining to give a patinated appearance (as discussed further below). Although no instance of rubbing in or gluing on of dirt or soil has been reported, as far as we know, it is not beyond the bounds of possibility. The motivation for our study was that modern imaging and analytical techniques should be able to detect such treatment, especially when questionable coins are compared with genuine pieces of the period. If the coins proved to be fakes, they would make a particularly interesting case study in antiquarian forgery; if authentic, they would be of clear historical interest. Either way, it is important to clear up an issue that was described as an ‘unsolved mystery’ in the standard catalogue of Roman Imperial Coins issued in 1948 [2] and has received relatively little attention since.

Details of the supposed discovery of the Sponsian coins and related pieces come from a handwritten note by Carl Gustav Heraeus (1671–1725), Inspector of Medals for the Imperial Collection in Vienna. In March 1713, he documented the acquisition of eight gold coins of five different design types, only one of which features Sponsian, that were allegedly found in Transylvania and acquired from ‘Hof-Cammer-Rath Palm’ [3]. We identify this person as the senior Habsburg finance minister (Hof-Cammer-Rath = Court Chamber Advisor) Johann David von Palm (1657–1721) whose responsibilities included banking, mines and precious metals [4]. This is interesting in itself because it implies the alleged find was processed through official channels. As many as 15 other coins similar to those described by Heraeus are known from other collections, appearing in the published record from 1730 onward (previously reviewed in [3, 5]), hence it seems likely that Heraeus selected a representative sample from a larger group (which we call the ‘wider assemblage’) which was dispersed on the market at the time.

Four coins now in The Hunterian in Glasgow were identified as part of the wider assemblage in 1997 [6] and form the basis of this study. These had been part of an extensive collection of ancient coins acquired in 1782 by William Hunter (1718–1783) from the estate of noted Viennese antiquarian Joseph De France (1691–1761) [7]. De France may have purchased the four coins of interest in 1713 or acquired them from a third party. Here we report a series of non-destructive micro-analytical investigations of these coins and two undoubtedly genuine Roman gold coins for comparison. Before reporting the results, we review the coins of the wider assemblage, their known provenances, and the puzzling features that have led specialists down to the present day to question their authenticity.

## Coins of the wider assemblage

**Fig 2:**
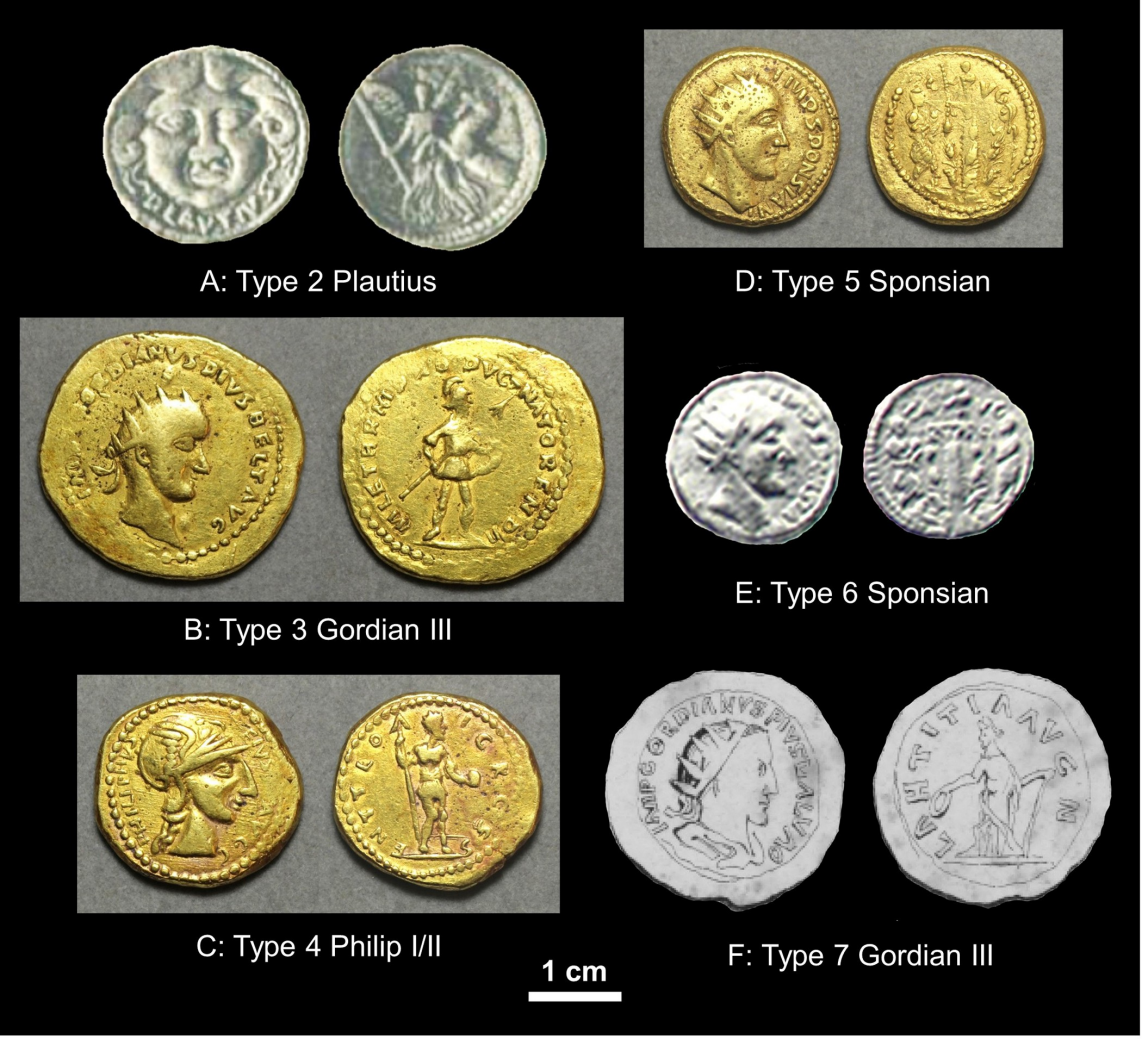
Coins of the wider assemblage, and their typology. Note that no image exists of the lost Type 1 of Alexander the Great. A) Type 2 of Plautius, Specimen 2.4 in Table 1, greyscale photograph reproduced from Ref. [3], scale approximate; B) Type 3 of Gordian III, Specimen 3.2 in Table 1, GLAHM:29596, photographed for this study; C) Type 4 of Philip I or Philip II, Specimen 4.4 in Table 1, GLAHM:29820, photographed for this study; D) Type 5 of Sponsian, Specimen 5.3 in Table 1, GLAHM:40333, photographed for this study (same as Fig 1); E) Type 6 of Sponsian, Specimen 6.1 in Table 1, greyscale photograph reproduced from Ref. [28], scale approximate; F) Type 7 of Gordian III, Specimen 7.1 in Table 1, greyscale line drawing reproduced from Ref. [29], scale approximate.

**Fig 3:**
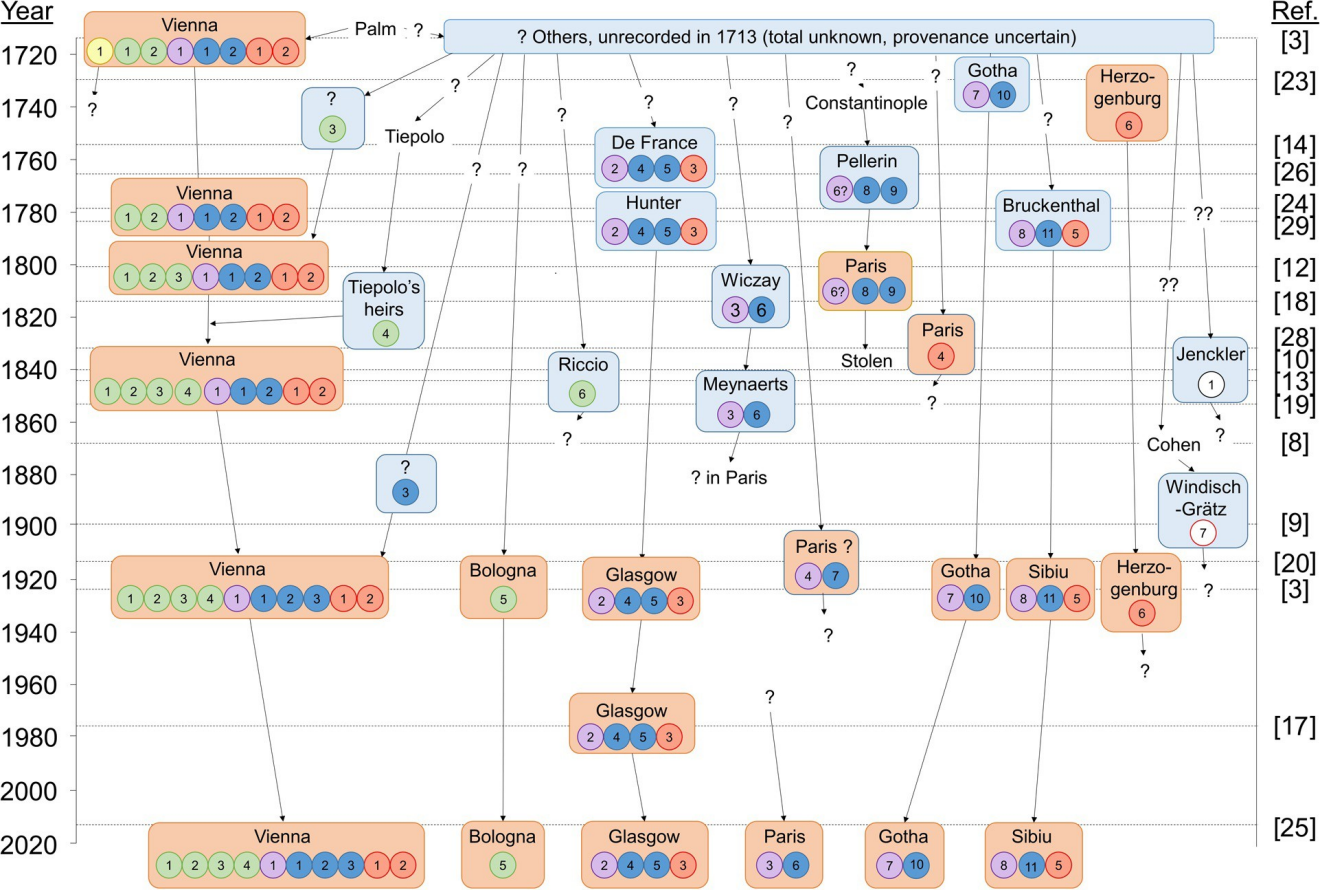
Diagrammatic representation of known and likely provenance trails of the wider assemblage. Yellow = Type 1, green = Type 2, purple = Type 3, dark blue = Type 4, dark orange = Type 5, white with red outline = Type 6, white with black outline = Type 7. Numbers correspond to individual coins of each type as given in Table 1. Orange boxes = public collections, light blue boxes = private collections. Apart from the Glasgow group discussed herein, confirmed coins are Vienna Münzkabinett RÖ 32.227 and RÖ 32.228; Bologna, Museo Civico Archeologico MCABo Num 26424, Bibliothèque nationale de France K 2339 and number unknown; Münzkabinett der Stiftung Schloss Friedenstein Gotha, 1.Co14611 and 1.Co14612; and Brukenthal National Museum, Sibiu.

The various types of coin from the wider assemblage are illustrated in Fig 2 and described in Table 1 along with a catalogue of known specimens and their weights. These include the five types described by Heraeus (which we call Types 1–5) plus a single silver Sponsian coin known from later references [8, 9] (Type 6) and another unique silver coin, now lost, which on stylistic grounds appears to be related to the group [10] (Type 7). While all of these coins have long been assumed to be part of the 1713 ‘discovery’, independent provenances are possible, especially for the silver coins because one might have expected Heraeus to have included examples in the Vienna Collection if they had been known to him. The provenance trails as currently understood are illustrated in Fig 3. We know the current whereabouts of only four coins of Sponisan–two in Vienna and one each in Glasgow and Sibiu.

**Table 1:** List of known coins from the wider assemblage, classified according to type. Note that when more than one weight is given in the literature the most recent is preferred. References are for specimens illustrated by line drawing or photography (‘P’ denotes a photograph) except where no illustration exists when the earliest published reference to the coin is given.

**Type 1: Alexander the Great (r. 336 BCE– 323 BCE)**			
Description: Gold. Design unknown.			
Discussion: A single specimen of this type is attested in the handwritten note of 1713 by Heraeus and was later reported as missing [3]. It was described as beautiful (*schöne*) by Heraeus, suggesting that it was an authentic Macedonian gold stater [3].			
Number	Group	Weight (g)	Reference
1.1	Vienna (lost)	?	[3 (not illustrated)]
**Type 2: *L. Plautius Plancus, Roman Republic,* 47 BCE**			
Description: Gold. Obverse, PLAVTIVS, facing mask of Medusa, hair in thick streams without snakes. Reverse, winged figure of Aurora facing, flying left, holding staff to left, rearing horse to right, facing left; second horse’s head above goddess visible on some specimens.			
Discussion: Both the obverse and reverse are based on a Roman Republican silver denarius issued by L. Plautius Plancus in 47 BCE (‘Minucia 3’ in Ref. [11]). The obverse design is crudely engraved and the legend differs from genuine Plautius coins which read L PLAVTIVS. The reverse design of genuine Plautius coins features the winged figure of Aurora flying to the right between four horses rearing to the right, with Aurora holding the horses by the reins and sometimes also a palm frond. This type, in contrast, features the goddess flying to the left with one horse to the right, rearing to the left. This mirror image effect suggests that the engraver began by copying from a real coin in negative relief. The goddess holds a long staff in her right hand, which is unknown in genuine Plautius coins, on which there are two more horses in this position. Perhaps the engraver abandoned the detailed work and chose to balance the composition with a staff. It also lacks the legend PLANCVS which is part of the original design, possibly because insufficient space was left for it.			
Münsterberg [3] rejected the notion that these coins are genuinely of Plautius and cited various earlier authorities who had declared them to be either ancient ‘barbarous’ copies or fakes. He suggested that there were two variants of reverse, one in which there is just one horse and one with a second horse’s head above the head of the goddess. Examination of Münsterberg’s plate, however, shows that the designs are identical in all other respects (the precise tracing of the drapery and wings, for instance) and so must have been produced from the same stamps. The lack of a second horse’s head on some may be due to partial impression.			
Number	Group	Weight (g)	Reference
2.1	Vienna	6.91 [3]	[3 (P)]
2.2	Vienna	12.55 [3]	[3 (P)]
2.3	Vienna	6.91 [3]	[3 (P)]
2.4	Tiepolo	9.92 [3]	[3 (P)]
2.5	Bologna	10.33 [3]	[3 (P)]
2.6	Riccio	?	[12, 13]
	Average:	9.32	
	%CV:	25.96	
**Type 3: Gordian III, Roman Imperial, 238–244 CE**			
Description: Gold. Obverse, IMP GORDIANVS DIVS BELT AVG (or similar), radiate head right. Reverse, MLETHRM PRODVGNATOREN DII (or similar), Mars standing right with shield and oblique spear.			
Discussion: The design is copied from a silver antoninianus of Gordian III (238–244) (‘RIC147’ in [13]), albeit in crude style and with blundered legends and at almost twice the original diameter. The obverse shows a radiate crown, which ostensibly suggests that the denomination is a double-aureus or ‘binio’, but could simply be because the silver coin from which it is copied has a radiate crown. The obverse legend should be IMP GORDIANVS PIVS FEL AVG. The reverse has Mars standing rather than advancing to the right with billowing cape as is usual. The reverse legend should read MARTEM PROPVGNATOREM. The bungling gives the impression that the engraver was illiterate. The final extraneous letters may have been added to balance the composition.			
Number	Group	Weight (g)	Reference
3.1	Vienna	22.73 [3]	[3, 14–16 (P)]
3.2	Glasgow	18.87 [17]	[this study (P)]
3.3	Wiczay	17.20 [18]	[18, 19]
3.4	Gnecchi	16.79 [3]	[20]
3.5	Paris	13.75	[21]
3.6	Mionnet	?	[22]
3.7	Gotha	12.50 [2]	[23]
3.8	Sibiu	?	[24, 25 (not illustrated)]
	Average:	16.97	
	%CV:	24.14	
**Type 4: Philip I and/or II, Roman Republican/Imperial, 244–249 CE**			
Description: Gold. Obverse, PHILIPHVS FIVS AVGG (or similar), head of Roma right with winged helmet. Reverse, ENTTLOICKCSS (or similar), emperor with radiate crown standing right with vertical spear upward and globe.			
Discussion: the obverse legend apparently refers to the Emperor Philip I and/or his son Philip II (244–249 CE), albeit misspelt and with an extra ‘G’ at the end (it should probably read PHILIPPVS PIVS AVG, although that would still be a unique legend for either emperor). Various authors [e.g., 11] described the obverse as the head of Philip I in a winged helmet but Philip is never shown as such in other coins, and always has a stubbly beard. It has also been described as the head of a female [18]. We interpret it as the head of Roma copied from a Republican denarius of the first century BCE with the curious legend squeezed to left and right as an afterthought, and we note that apart from the legend it would be an appropriate obverse for the Sponsian reverse (see Type 5, below). The reverse is based on the PRINCIPI IVVENT design (common for Philip II, and other junior emperors of the period; see ‘RIC218’ in [13]), except that the figure faces right, the spear points upward, and the legend is seemingly a meaningless jumble of letters, including what appears to be a ‘K’, which would be exceptionally rare in Latin script.			
Number	Group	Weight (g)	Reference
4.1	Vienna	10.66	[3, 15, 16 (P), 24]
4.2	Vienna	10.96	[3 (P), 25 (P)]
4.3	Vienna	16.65	[20 (not illustrated)]
4.4	Glasgow	14.83	[25 (P), this study (P)]
4.5	Glasgow	11.77	[25 (P), this study (P)]
4.6	Wiczay	11.72	[18, 25]
4.7	Paris	16.79	[20]
4.8	Pellerin	12.64	[26]
4.9	Pellerin	10.43	[26 (not illustrated)]
4.10	Gotha	11.53	[23 (not illustrated)]
4.11	Sibiu	?	[24 (not illustrated)]
	Average:	12.80	
	%CV:	18.84	
**Type 5: Sponsian, Roman Republican/Imperial, date uncertain**			
Description: Gold; obverse IMP SPONSIANI (or similar), radiate head right. Reverse C AVG, togate figures standing facing one another, right and left of a beaded column; the one on right holds lituus, the one on left an uncertain object; on column, statue with beaded staff; to right and left bells above, corn ears below.			
Discussion: The obverse shows a radiate head, as is very common in third century silver coinage where it denotes a double-denarius or ‘antoninianus’. The material is gold, however, ostensibly suggesting that the denomination is a double-aureus or ‘binio’, as in Type 3. The obverse legend is highly aberrant in being placed right of the bust only, and apparently being in the genitive case (= ‘of the imperator Sponsian’). The reverse design is taken from a Republican denarius of Caius Minucius Augurinus minted in 135 BCE, depicting a monument in Rome dedicated to the moneyer’s ancestor (‘Minucia 3’ in [9]). The C AVG in the original represents C[AIVS] AVG[VRINVS]. The handwritten note of Heraeus from 1713 reproduced in [2] describes two coins of the same reverse design but different imprints (*gepräges*), but all published photographs, including the two Vienna specimens [2], are of the same imprint, so we presume Heraeus was mistaken.			
Number	Group	Weight (g)	Reference
5.1	Vienna	9.38	[3 (P)]
5.2	Vienna	10.07	[3 (P) 27 (?)]
5.3	Glasgow	10.84	[6 (P), this study (P)]
5.4	Paris	?	[28]
5.5	Sibiu	?	[29]
5.6	Herzogenburg	9.80	[3 (P)]
	Average:	10.02	
	%CV:	6.13	
**Type 6: Sponsian, Roman Republican/Imperial (imitation), date uncertain**			
Description: As Type 5 except silver.			
Discussion: Cohen [25] mentioned having seen a silver specimen similar to the gold coins of Sponsian but did not record any further details about it. Voetter [28] illustrated a silver Sponsian coin (weight unknown) in a catalogue of the collection of Prince Ernst Windisch-Grätz (1872–1897) which claims to be ‘one of’ the silver specimens mentioned in Cohen and found in Transylvania in 1713 (*Eines von den Silberstücken*, *die in Cohen erwähnt werden und 1713 in Siebenbürgen gefunden worden sind*) although this is doubtful because Cohen mentioned only one coin and Heraeus did not record any silver pieces among the 1713 group. We note that this specimen is of the same obverse and reverse stamps as the published gold coins of Type 5. Its subsequent whereabouts is unknown.			
Number	Group	Weight (g)	Reference
6.1	Windisch-Grätz	?	[9, P]
**? Type 7: Gordian III, Roman Imperial, 238–244 CE**			
Description: Silver, obverse, IMP GORDIANVS PIVS LALVAO (or similar), radiate head right. Reverse, LAHTITIA AVG N (or similar), Laetitia facing left with wreath and anchor.			
Discussion: This coin, now lost, is copied from a regular antoninianus of Gordian III (‘RIC86’ in [2]) at enlarged scale. The obverse legend is a bungled version of IMP GORDIANVS PIVS FELIX AVG, ending with meaningless letters. The reverse should read LAETITIA AVG N. This type is included here as a possible part of the wider assemblage. A single example is known, described as ‘pure’ silver and illustrated by Jenckler [10]. It was considered by Jenckler to be of the same manufacture as the Gordian III and Philip types discussed above, albeit minted in silver. Jenckler did not outline his reasoning, but the ‘barbarous’ legends and style of engraving is similar, judging from his illustration, and although no scale was provided, the coin was depicted as being significantly larger than regular anotoniniani on the same engraved plate, which is reminiscent of the Type 3 coins. We note that the irregular outline shows that the reverse must be in a 12 o’clock orientation like all known coins of the wider assemblage. In a hitherto overlooked publication, Krafft [30] added the significant detail that the coin was ‘from Trier’, implying that it was found there, hence if it is part of the wider assemblage it appears to have a separate provenance. Alternatively, it may be an entirely unrelated ‘barbarous’ type.			
Number	Group	Weight(g)	Reference
7.1	Jenckler	?	[8]

Apart from a lost coin of Alexander the Great (Type 1) which may have been a genuine Macedonian stater, the coins are ostensibly Roman although of peculiar workmanship. They have many common stylistic features including bold portraits with prominent chins and bulging eyes, and characteristically chunky lettering (the tapering ‘S’ is particularly distinctive), which makes us confident they are the work of a single engraver. The group features two well-known emperors, Gordian III (r. 238–244 CE) and Philip I (and/or his son Philip II, r. 244–249 CE), making the oldest possible date of manufacture 244 CE. Perplexingly, however, one type (Type 2) is copied from a Republican silver coin from the first century BCE, and Types 5 and 6, featuring the unknown ‘emperor’ Sponsian in mid-third century style, have a reverse design copied from a Republican issue that would have been over 370 years old at the apparent time of manufacture! Type 4 also mixes Republican and Imperial elements. Another unusual feature of the group is their heaviness and high variability in weight, both between types and between coins of the same type (see Table 1). The known weights of coins of the entire assemblage average 12.66 g with a coefficient of variation of 29.70%, which compares with 4.89 g, and 5.8% for regular gold coins (aurei) of Gordian III, and 4.62 g, and 7% for regular aurei of Philip I [31].

Early specialists accepted the coins as genuine products of antiquity and classified them alongside so-called ‘barbarous’ imitations of Roman coins that were frequently manufactured beyond the fringes of the empire [e.g., 23, 32]. Sponsian came to be regarded as a short-lived local ‘usurper’, otherwise unknown to history, who may have made a bid for supreme power during the civil wars of 248–249 CE that ended Philip’s reign [e.g., 28]. However in 1868, Henry Cohen, the leading expert of the time, declared the coins as poorly made and ridiculously imagined modern fakes (*je regarde ces pièces comme des coins modernes ridiculement imaginés*, *et très-mal faits*) (p. 264 in [8]). A series of other specialists agreed, up to and including a short but important 1923 publication by Münsterberg [3] who pointed out that the coins appear to have been cast from moulds, as is common in historical fakes, as opposed to being struck, which was the normal practice in antiquity. He also argued that the large weight variation meant they could not have had a meaningful face value.

Imitations and forgeries of ancient coins are known to have been made from the Renaissance period onward [33, 34]. Prominent early examples of the former are the so-called ‘Paduan’ medallions of Giovanni Cavino who struck an extensive series of highly accomplished Greek and Roman imitations, mostly in bronze, in the period from 1560–1590 [35]. Although apparently not intended to deceive, some of these nevertheless passed as genuine ancient coins among subsequent collectors [36]. Other workshops from the sixteenth century onward produced outright forgeries for sale to wealthy collectors. These were generally cast rather than struck using real coins as hubs in the casting process, thereby obviating the need for new engraving [33]. Detailed methods for detecting cast fakes were published as early as 1558 [37]. Because freshly cast copper or bronze lacks the surface oxidation layer (patina) typical of ancient coins, forgers of the period used paints and heat treatments to simulate it [37]. It has also been reported that newly forged coins were artificially abraded to simulate wear [33].

In the most recent detailed discussion of the problems posed by the Sponsian assemblage, Bursche [5] suggested they were the work of a sophisticated fraudster operating in early eighteenth century Vienna who serially duped antiquarian collectors. By this hypothesis, the coins were consciously intended to appear ‘barbarous’ with illiterate scripts and enigmatic designs so as to confuse the experts. The centrepiece of the deception was the fabricated emperor Sponsian, who was presumably intended to appeal to the curiosity of potential collectors, attracting a rarity premium. The fakery–if that is what it was–must have required considerable investment of time and money: the weight of gold in the known assemblage exceeds $20,000 in modern value. This also seems to rule out an alternative possibility that the coins are some kind of flight of fancy on the part of some eccentric antiquarian of the past.

Nevertheless, arguments against the fakery hypothesis can also be raised. The coins of the Sponsian assemblage are highly atypical of early cast forgeries in that they used newly engraved designs as hubs rather than real coins. The standard of engraving is not very accomplished and is not aligned with the classical aesthetics that dominated the early collecting market. There was little public interest in third century Roman history in the eighteenth century; all the early fakery that we know of featured Greek or Roman figures of greater renown from a more classical period [38]. It also seems odd that Sponsian was given such an involved context of other fake designs, that his coins are numerically in a minority among the known wider assemblage (the most common design among surviving pieces being the Type 2 Plautius coins), that they are the least impressive of the various designs, and that no special care was taken either in the engraving (especially the obverse legend down one side of the head only) or manufacture (the hub slip on GLAHM:40333, for instance, seems careless). If early price catalogues from 1823 onward [21] are taken as a guide, the Sponsian coins were not especially valued by collectors in comparison to those of well-known emperors.

The name Sponsian itself is highly peculiar and a far from obvious choice from the point of view of a hypothetical forger. Only one other instance of it is known, from a first century funerary inscription in Rome which names an obscure individual called Nicodemus Sponsian [39]. Here we emphasize the fact that the inscription was excavated in the 1720s [40] so could not have been known to a hypothetical forger, who would therefore have to have invented a peculiar name that later proved genuine. Perhaps because of these anomalies, Sponsian was retained in the authoritative catalogue of Roman Imperial Coins issued in 1948 [13] and his possible existence has been entertained by some authors [e.g., 6, 41, 42]. But because his historicity relies entirely on the coins, most modern works on third century history omit mention of him [e.g., 43–45]. The latest Roman prosopography (catalogue of people) follows the current consensus and lists him under ‘disregarded persons’ on the grounds that the coins are almost certainly fakes [46].

## Imaging and spectroscopy

We undertook a detailed investigation of the four Glasgow specimens (one of Sponsian, one of Gordian III and two of Philip I/II) plus two genuine Roman gold coins of the third century (of Gordian III and Philip I) for comparison (Table 2). The two genuine coins are also from Hunter’s eighteenth century collection but their earlier provenance is unknown. Separate reports for each analytical method are presented in S1–S3 and S6 Files, where all the data and images gathered in the study are presented. Because the different techniques produce overlapping and complementary evidence, the results are synthesized and presented here by topic. All necessary permits were obtained for sampling and analysis from The Hunterian, University of Glasgow, for the described study, which complied with all relevant regulations.

**Table 2:** List of specimens analysed. All specimens are in the collection of The Hunterian, Glasgow, UK.

Catalogue number	Type	Status
GLAHM:29540	Gordian III gold aureus	Genuine
GLAHM:29697	Philip I gold aureus	Genuine
GLAHM:29596	Gordian III ‘medallion’ / binio	Questionable
GLAHM:29820	Philip I/II ‘medallion’	Questionable
GLAHM:29821	Philip I/II ‘medallion’	Questionable
GLAHM:40333	Sponsian medallion / binio	Questionable

## Methods

### Visible Light microscope (LM) and Ultra-Violet (UV) imaging

LM and UV imaging was conducted at the Kelvin Centre for Conservation and Cultural Heritage Research, School of Culture and Creative Arts, University of Glasgow. Initial photography was with a Canon EOS 700D camera equipped with a Canon EFS 18–55 lens to obtain general images of obverse and reverse surfaces. Coins were further imaged using an optical Leica M165C stereomicroscope with MC 190 HD 10-megapixel digital camera, Tango Mini automatic stage and LASX software v. 3.0.9 using magnifications ~30x - ~350x under white LED ring light. Images were taken in single mode and extended depth of field (multifocus) mode. Various images of the coins were taken at different magnification, aimed at documenting their characteristic features, and a standardized set of images was made at the highest available magnification consisting of two images on each side, one of the most exposed flat area of relief and another on a relatively protected flat area of the field. Additionally, three multifocus images perpendicularly to the edges were taken on each coin, under dispersed white light in ~30x magnification. For UV imaging, coins were photographed in a dark room under a Sylvania F18 T8 BLB UV light (spectral peak 365 nm) with a Canon EOS 700D camera equipped with a Canon EFS 18–55 lens and Calumet UV filter. All images are presented in S1 File (LM) and 2 (UV).

### Scanning electron microscopy (SEM)

SEM analysis was conducted at the Imaging, Analysis and Spectroscopy Centre (ISAAC), University of Glasgow with a Zeiss Sigma VP Field-Emission SEM, equipped with an Oxford Instruments X-Max 80 mm^2^ Energy Dispersive (EDS) detector. Wear patterns on metal were imaged with a secondary electron (SE) detector in high vacuum conditions at a working distance of ~8.5 mm, accelerating voltages of 15–20 kV and aperture size of 60 μm. The same settings were used for EDS analysis, except that the accelerating voltage was kept at 20 kV. Variable-pressure mode was utilized for SE imaging and EDS analysis of earthen deposits to minimise undesirable charging effects caused by electrons not being conducted away from the analysed surfaces. In variable-pressure (low-vacuum mode), nitrogen gas is introduced in the chamber where it absorbs and neutralizes negatively charged electrons on the sample surface. The use of variable-pressure mode allows analysis of non-conductive materials without applying conducive coating.

EDS analysis was performed using Aztec software from Oxford Instruments with process time 4 and analysis acquisition stopped after 300,000 counts with “pulse pile up correction” enabled. Data processing involved normalization of elemental compositions to 100%, and stoichiometric correction for those measurements of earthen deposits assumed; because oxygen cannot be correctly measured by the EDS technique due to its low atomic weight, its amount is calculated based on the number of cations detected in the measured spectra. We also detected that in many measurements the Aztec software misidentified the gold N4-N6 peak at 0.265 keV as the carbon Kα peak at 0.227 keV, hence during data processing the option to label this peak as carbon was removed.

Other sources of potential error in EDS measurements arise from the nature of the analysed specimens, notably the irregular three-dimensional surfaces analysed and the presence of mixed materials that include fine-grained nanoparticles of various compositions. In addition, most EDS measurements of earthen deposits indicated a presence of Au, Ag, Cu and C, which we interpret as a signal from the underlying coin surface or nano-scale gold flakes that have been displaced from the coin. Therefore to interpret the compositions of earthen deposits during data processing we removed Au, Ag, Cu and C from the normalized data by a deconvolution method. The topography of sample surfaces affects the accuracy of EDS measurements because surfaces that are not horizontal scatter and absorbs X-rays in unexpected ways. To reduce this source of error, we focused measurements on areas with low local topography (maximum of 1–2 μm in cases with some of the earthen deposits) and performed multiple measurements at various spots of each phase to test the consistency of the data. Because of these difficulties, and despite the precautions taken in data processing, the compositional data of the earthen deposits should be considered only as indicative for the major elements in the analysed minerals. Images and all spectra obtained, including the raw unprocessed data, are presented and discussed in S3 File. Data, including raw unprocessed data, are available in S4 File and a summary of coin metal analyses is given in S5 File.

### Reflection mode Fourier Transform Infra-red spectroscopy (r-FTIR)

Analysis was carried out at the Kelvin Centre for Conservation and Cultural Heritage Research, School of Culture and Creative Arts, University of Glasgow using a Nicolet iN10 FTIR AutoImage microscope equipped with cooled mercury-cadmium-telluride (MCT) detector, which operates in the 4000–675 cm^-1^ spectral range. The parameters chosen for the analysis at high spectral resolution were: 22 sec, 64 scans, 4 cm^-1^ resolution, 100x100 μm aperture, with Beer-Norton strong apodization. No further spectral correction was applied for data interpretation. Points of interest were chosen based on the results of microscopic investigation by visible and UV light.

To our knowledge, no previous attempt has been made to analyse earthen deposits on coins using micro-FTIR in reflection mode (r-FTIR), i.e. in a completely non-destructive way. Previous studies on corrosion on copper coins and other artefacts have used Attenuated Total Reflection (ATR)-FTIR on micro-samples scraped from surfaces [47–50]. The use of r-FTIR offers the chance to analyse micrometric residues of soil when destructive sampling is prevented due to small sample size or museum restrictions. r-FTIR spectra are usually more difficult to interpret, as they present spectral distortions due to selective absorption phenomena when organic or oxyanion-containing inorganic compounds are present, and appropriate spectral libraries lack for comparison [51]. However, gold coins offer the advantage of having a highly reflective surface, which limits the number of spectral distortions, making r-FTIR spectra more easily comparable to standard ATR or transmission spectra, for which extensive libraries are available. Images and spectra are presented in S6 File.

## Results

### Composition

**Fig 4:**
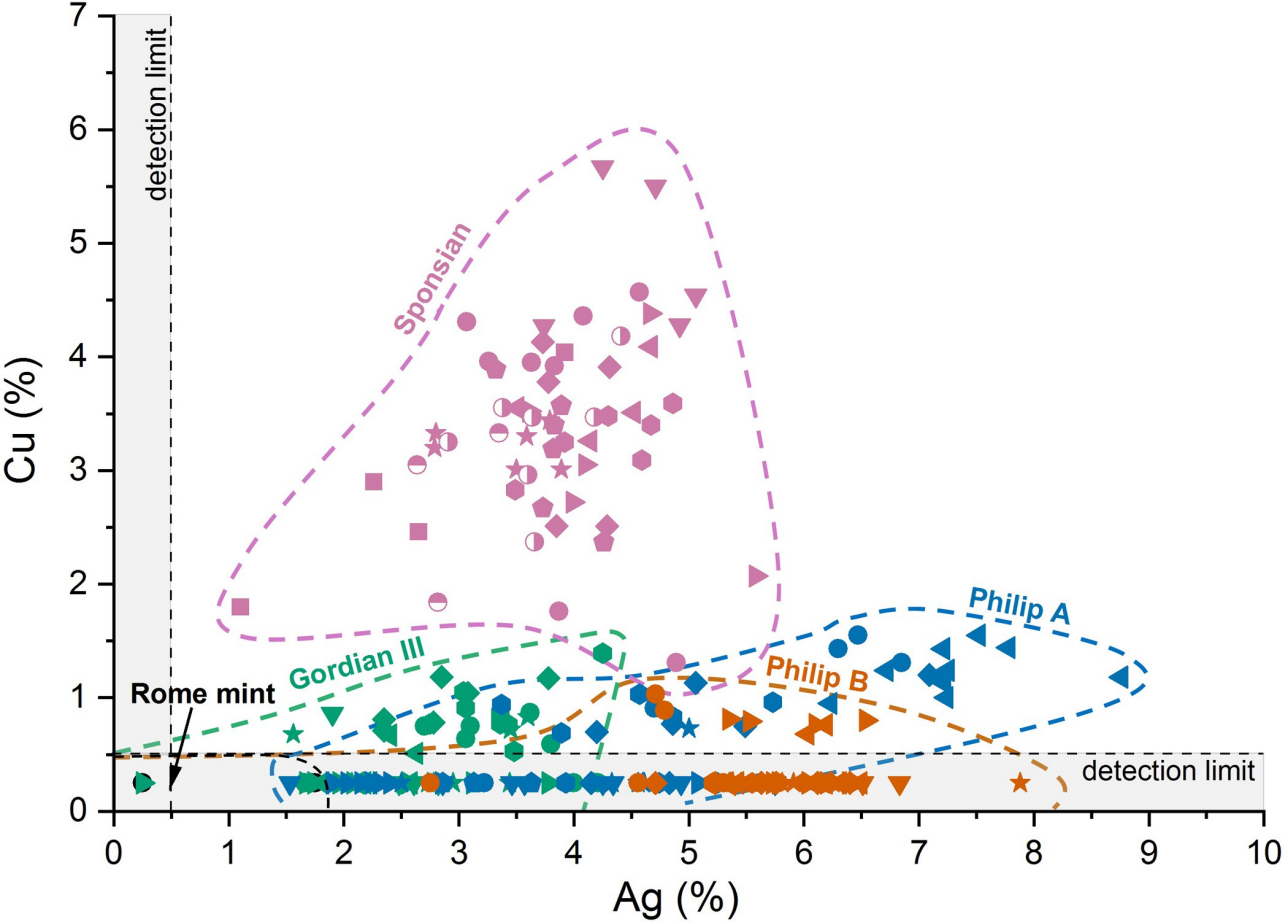
Variation of silver and copper content of the six coins analysed in this study by SEM-EDX point analyses. Gordian III = GLAHM:29596; Philip A = GLAHM:29820; Philip B = GLAHM:29821; Sponsian = GLAHM:40333. Different symbols refer to different zones within each coin; each point is a different surface analysis. For each coin, circles = zone 1, down triangles = zone 2, diamonds = zone 3, left triangles = zone 4, right triangles = zone 5, hexagons = zone 6, stars = zone 7, pentagons = zone 8, squares = zone 9, horizontal half-filled circles = zone 10, vertical half-filled circles = zone 11. Coins of the Rome mint have no detectable copper and either low or undetected silver. Data are presented by coin and zone in S5 File and average compositions are given in Table 3.

Although the four questionable coins have always been described as gold, we wanted to determine the level of purity in comparison to the genuine Roman aurei. We also wanted to test the suggestion that they may have had a large base metal content that could have produced a reddish oxidation product of the surface, as has previously been suggested [5]. We analysed the metal composition of all six coins by SEM-EDX. For consistency this was done by a standard procedure on worn upper surfaces of the obverse bust, avoiding superficial deposits where possible. A series of spot tests was taken which may not, however, be representative of the bulk composition if there are significant heterogeneities, as was found to be the case for the questionable coins. Therefore, a second series of analyses was conducted in various other areas widely distributed across the reverse side of these four coins. Results are plotted in Fig 4 and summarized in Table 3.

**Table 3:** Composition of the six coins analysed in this study according to SEM-EDX (wt%—weight percent, n = number of analysis points averaged, s.d. = standard deviation). Note that because a value of 0 wt% is automatically returned for analyses below the practical detection limit in the range 0.0–0.5 wt%, such results were assigned a value of 0.25 wt% for the purposes of plotting and the calculation of averages.

Coin GLAHM:	29540	29697	29596	29820	29821	40333
	(n = 7)	(n = 4)	(n = 48)	(n = 55)	(n = 45)	(n = 59)
Type	Gordian III	Philip I	Gordian III	Philip I	Philip I	Sponsian
Status	Genuine	Genuine	Questionable	Questionable	Questionable	Questionable
Au (wt%)	100.00	99.01	96.70	94.64	93.90	92.78
	(s.d. = 0)	(s.d. = 0.74)	(s.d. = 0.96)	(s.d. = 2.11)	(s.d. = 0.76)	(s.d. = 1.27)
Ag (wt%)	Undetected	0.99	2.77	4.73	5.76	3.83
		(s.d. = 0.74)	(s.d. = 0.78)	(s.d. = 1.74)	(s.d. = 0.75)	(s.d. = 0.75)
Cu (wt%)	Undetected	undetected	0.54	0.63	0.35	3.39
			(s.d. = 0.32)	(s.d. = 0.45)	(s.d. = 0.22)	(s.d. = 0.84)

It is evident from this exercise that the two genuine coins from the Rome mint are essentially gold of very high purity. No copper was detected in either coin and just a little silver (<2%) was detected in two of the four measurements taken on GLAHM:29697. The four questionable coins, on the other hand, have measurable silver and copper which varies markedly between coins and also between areas on the same coins. The Gordian III medallion is the purest of the four, with an average gold content of 96.70%, relatively low silver (2.77%) and a little copper (0.78%). The two Philip coins are also relatively low in copper but have significantly higher silver contents (averaging 4.73% and 5.76% respectively). The compositions of these two coins are sufficiently similar, given the levels of heterogeneity in both of them, that their bulk compositions may be the same. The Sponsian coin on the other hand is not only moderately high in silver (averaging 3.83%) but also high in copper (averaging 3.39%), giving it a distinctive composition unlike any of the others.

### Manufacture

**Fig 5:**
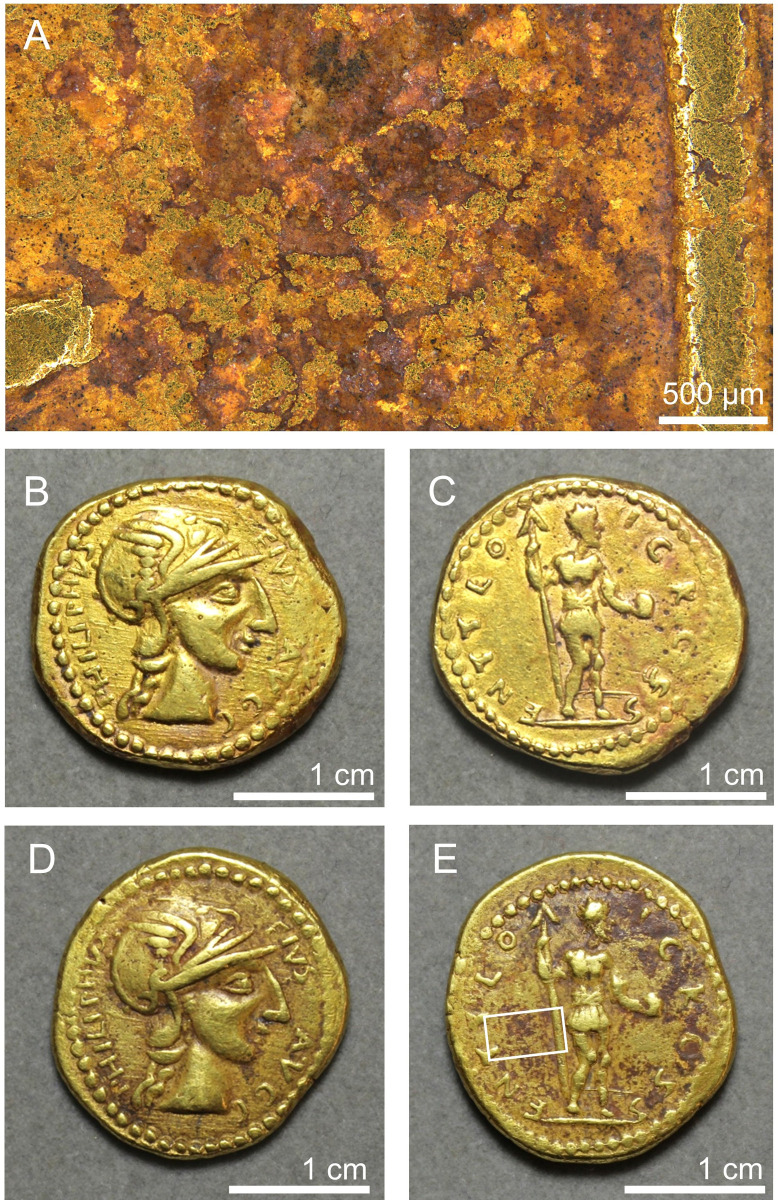
Evidence for casting. A, D, E, GLAHM:29821 (the box on E is the area shown in A); B, C, Coin GLAHM:29820 (same as Fig 2).

We wanted to establish if the coins were cast or struck and to investigate any other aspects of the mode of manufacture that might be determined. Unsurprisingly, the two undoubtedly genuine coins show characteristic features of striking such as radial smearing at the edges caused by expansion of the blank. However, our observations strongly support the suggestion [3, 5] that all the disputed coins were cast from moulds impressed by a master design, conventionally called a ‘hub’ (Fig 5). Evidence includes: a general lack of surface detail; small roughly circular pits of diameter 50–500 μm on GLAHM:40333 (see Fig 1) and to a lesser extent GLAHM:29596 and GLAHM:29820 which are interpreted as relict air bubbles; irregular edges to some features indicating incomplete filling of the mould, especially on GLAHM:29820 and GLAHM:29821; irregular cracking and flaking away of parts of the upper surfaces on all the questionable coins but especially GLAHM:29820, indicating instability of the original cast surfaces; curvilinear surface cracking patterns on GLAHM:29821 (Fig 5A) interpreted as caused by rapid cooling against the mould; widespread reddish-brown residues on the same coin in and around these cracks that are interpreted as the mould adhering in places (Fig 5A); and ‘hub slips’ such as on the reverse of GLAHM:40333 where the hub seems to have moved during soft impression, causing smearing of the outline and repeated impression of parts of the design (see Fig 1). Finally, the two Philip medallions in our study differ in aspect ratio by about 10% indicating horizontal plastic compression of the obverse and reverse designs on Specimen GLAHM:29821 (compare Fig 5B and 5D and 5C and 5E). Similar compression can be seen in published photographs of some of the others (Coins 2.3, 3.1, 3.4, 4.2, and 4.7 of Table 1) which may point to a small-batch manufacturing process wherein wet clay moulds were aligned vertically and tightened laterally in frames before firing.

Additional evidence of the manufacturing process begins with the fact that the designs have evidently been copied from genuine coins but are not of normal style, quality, or level of detail. The legends are frequently poorly spelled, ungrammatical or even meaningless in one instance, and in some places have been omitted, apparently through lack of space, squeezed in, or filled out with arbitrary (see Table 1). Some of the design elements have been reproduced in mirror image as would happen if an engraver began the work in negative relief and did not take particular care to flip the image. Another characteristic feature of the coins is that the blank fields often feature one or two sets of linear ridges (for example, see Fig 5B and 5D and S.1.6.3 in S1 File). The precise pattern of these ridges is common to multiple coins, including previously photographed specimens in other collections [25], hence they must have been inherited from a common hub. These lines are truncated by the engraving and so seem to have been present on the sheet (presumably metal) onto which the designs were first engraved. As far as we can determine, having examined all available photographs, only one obverse and reverse hub was used for each type of coin.

Building on these observations, we suggest that the manufacture involved a minimum of six stages:

1. engrave obverse and reverse designs in negative relief on a hard material such as bronze;
2. impress a soft substance, possibly pre-prepared clay tablets, into the engraving to produce positive relief hubs;
3. trim, then harden the hubs by firing;
4. impress the ceramic hubs into soft clay tablets;
5. align and assemble the obverse and reverse tablets in a frame, creating one-time moulds, leaving voids in between and channels to the outside, then harden by firing;
6. cast in molten metal, cool, extract the coins and clean up irregularities and spillage.

### Wear

**Fig 6:**
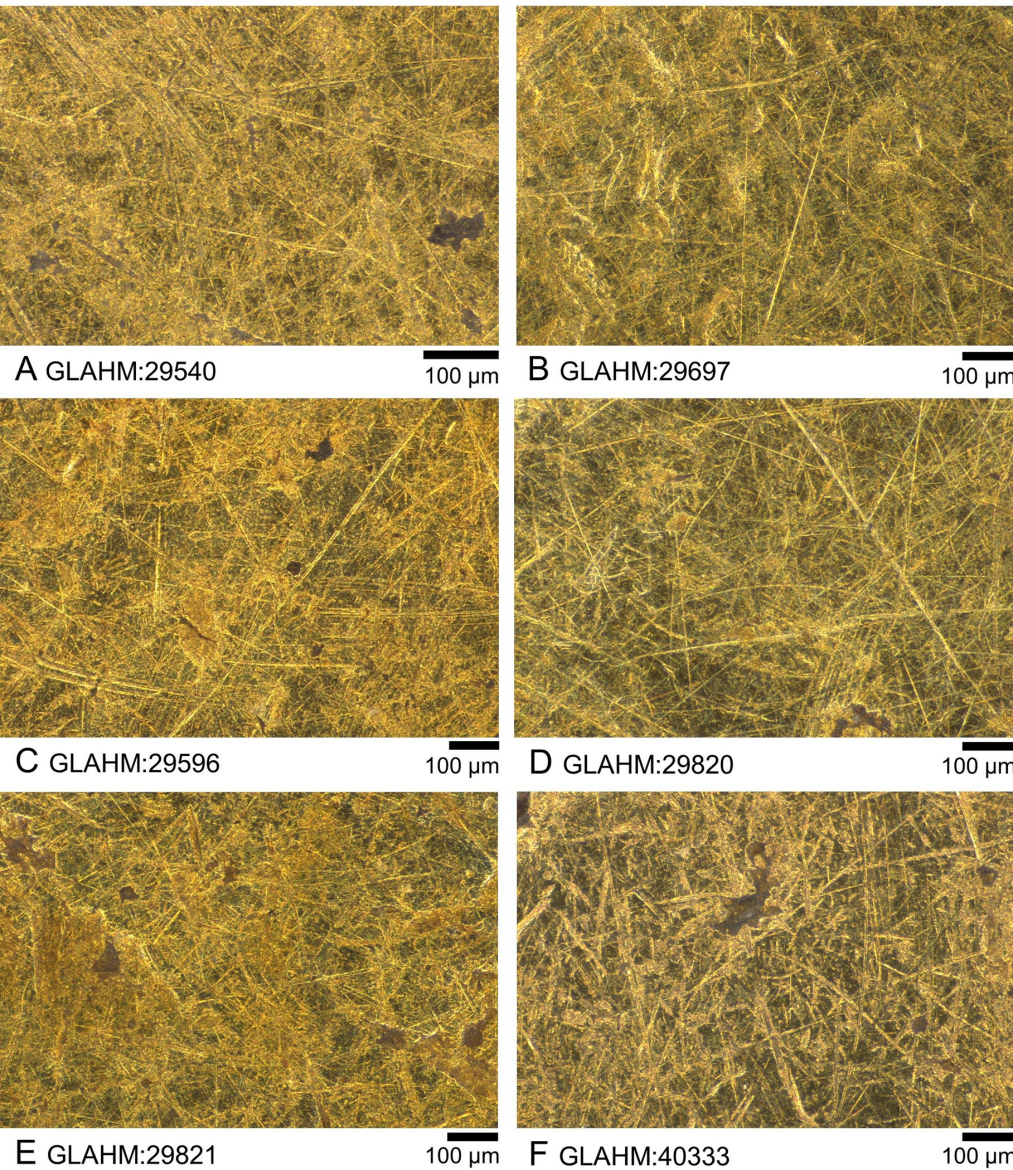
Light microscope images of exposed upper areas on the obverse of the six coins. The two unquestionably genuine coins are at the top. A) GLAHM:29540; B) GLAHM:29697; C) GLAHM:29596; D) GLAHM:29820; E) GLAHM:29821; F) GLAHM:40333.

We investigated wear patterns using LM and SEM imaging at increasing magnification, focusing in particular on the most exposed surfaces of the portrait for consistency (see S1 and S3 Files for the full set of standardised images across a wide range of magnifications). The objective was to characterise and compare all six coins. In particular we were interested in detecting signs of deliberate manual abrasion which we would expect to show uneven spatial distribution and deeper scratches of more consistent length and orientation than occur in natural wear. Typical results are shown in Figs 6 (LM) and 7 (SEM). All coins are deeply abraded by overlapping scratches and craters down to the highest available magnification. Scratches occur in all orientations and there is little parallelism in the imaged areas except among directly adjacent grooves which were presumably produced by the same abrading object. Scratches are linear or gently curving and have ‘V’-shape or ‘U’-shape profiles. They occur evenly on all exposed surfaces and to a lesser extent on the edges of the coins. As well as scratches and craters, we also noticed numerous brittle fractures around protruding elements such as the edges of lettering on all the coins.

**Fig 7:**
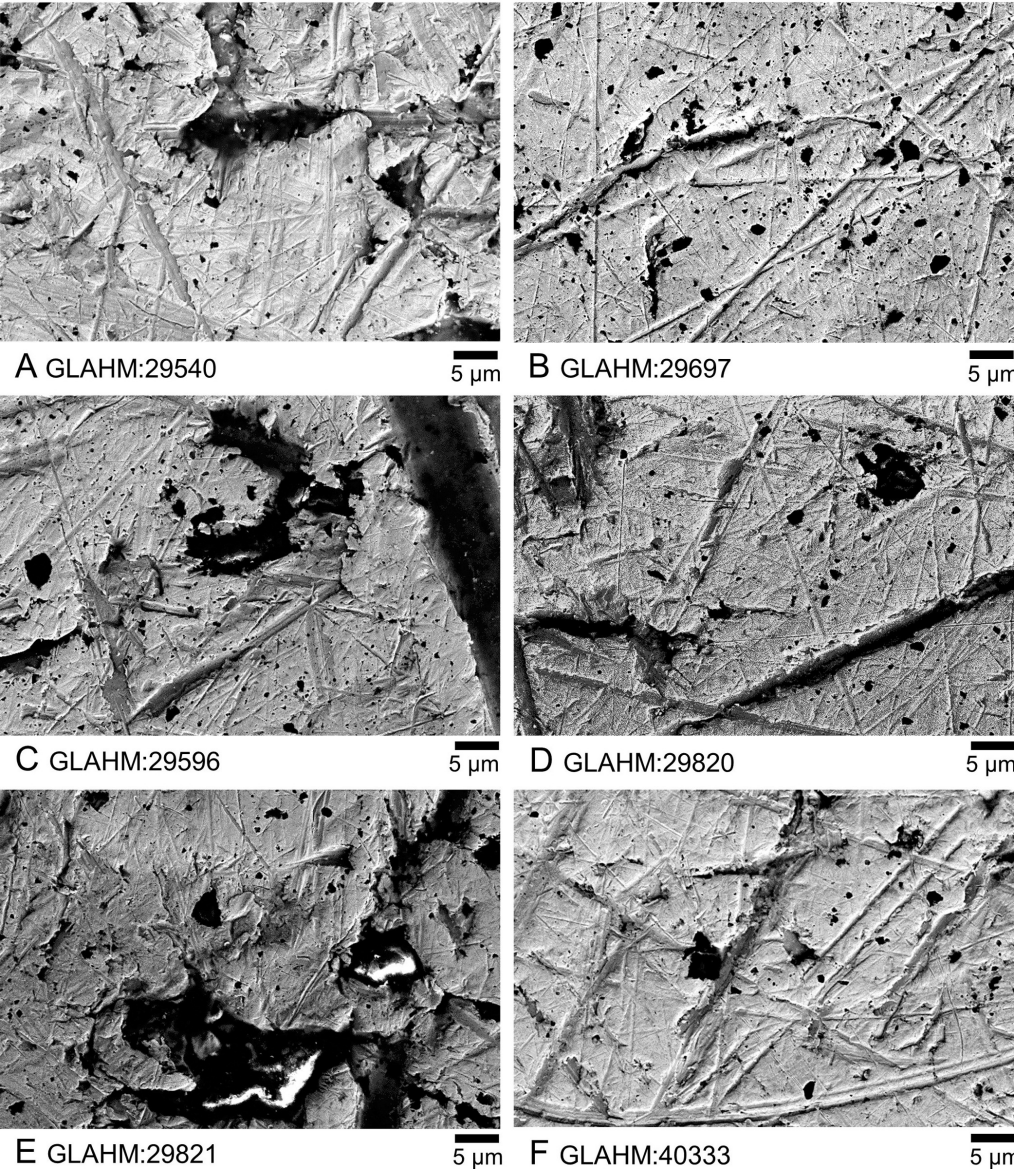
High magnification electron microscope images of exposed upper areas on the obverse of the six coins. The two unquestionably genuine coins are at the top: A) GLAHM:29540; B) GLAHM:29697; C) GLAHM:29596; D) GLAHM:29820; E) GLAHM:29821; F) GLAHM:40333.

### Superficial deposits

**Fig 8:**
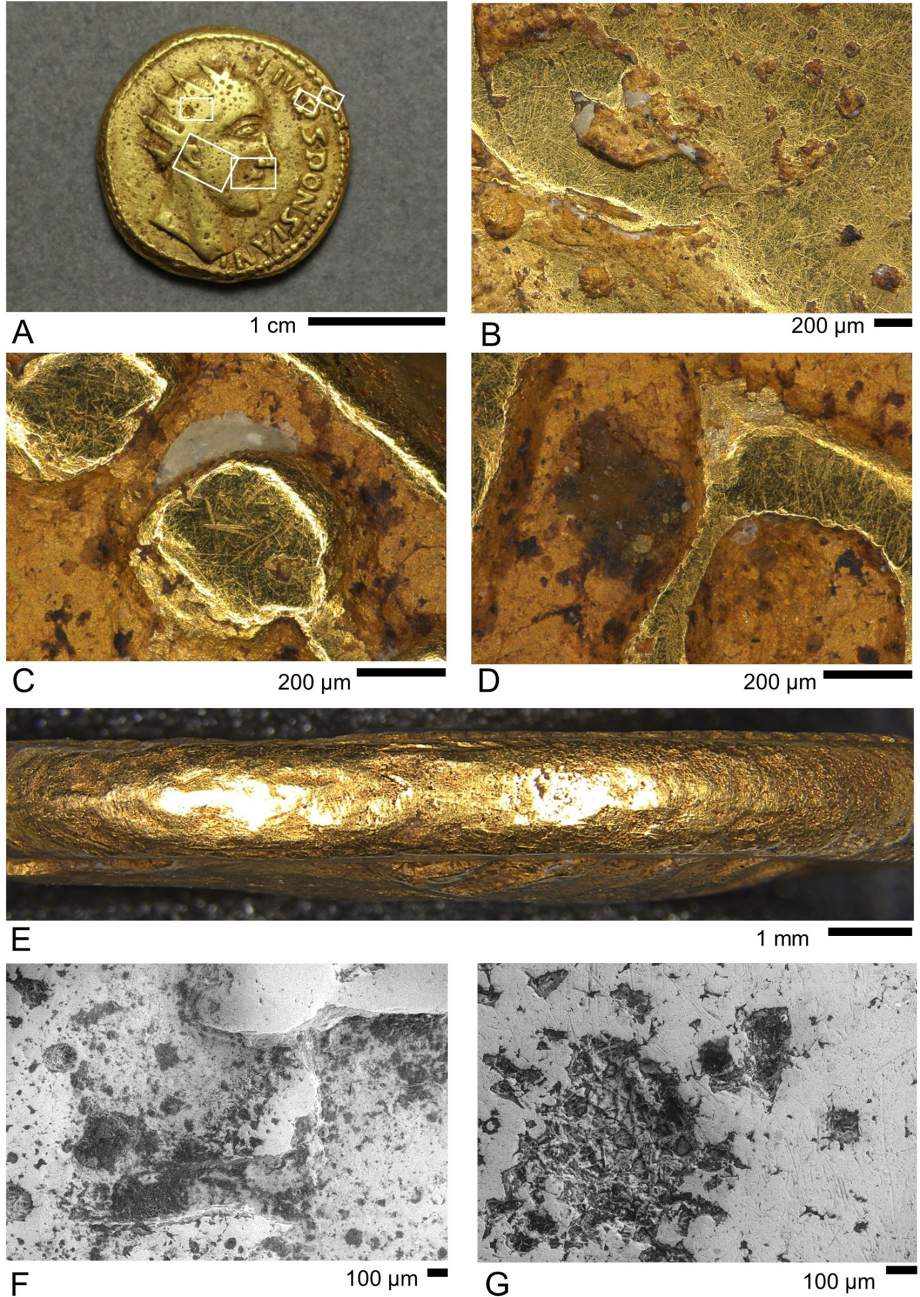
LM images of the obverse surface and edge of Sponsian coin GLAHM:40333. A) key to highlighted areas; B) detail of ear and cheek showing subcircular pits interpreted as casting air bubbles, within which the metal has an irregular surface where it did not impress the mould, wear scratches and areas of possible wax residue; C) detail of beading and edge showing wear scratches, possible wax residue and cementation spots; D) detail of lettering showing scratches, chipped edges, earthen deposit between letters and cementation spots; E) perpendicular view of edge; F) area of Sponsian’s mouth showing granular patches in recesses and remnant air bubbles; G) detail of large remnant air bubble.

Ancient coins are generally cleaned by their discoverers, but small adhering patches of soil (‘earthen deposits’) often remain in recesses, especially if the original matrix is relatively well cemented. The genuine Gordian aureus (GLAHM:29540) in particular has many such deposits on both sides, around the lettering and elsewhere. Smaller areas resembling earthen deposits were observed on the genuine Philip aureus (GLAHM:29697) and all the questionable coins. We are aware of no published literature specifically on the analysis of earthen deposits on coins. The aim of our investigation was to characterise and compare the deposits on both the real and questionable coins and in particular to test the hypothesis that dirt might have been rubbed, glued or baked into the questionable coins. The appearance of some of these features on the Sponsian coin (GLAHM:40333) are shown in Fig 8, which also shows an unexpected amorphous white material (Fig 8B and 8C) that we wanted to identify. Low magnification electron micrographs (Fig 8F and 8G) show that the larger patches of adhering material on this coin have a microgranular appearance with individual mineral grains visible in places. Tightly bound granular material overlays much of the surface and occurs in all recesses.

**Fig 9:**
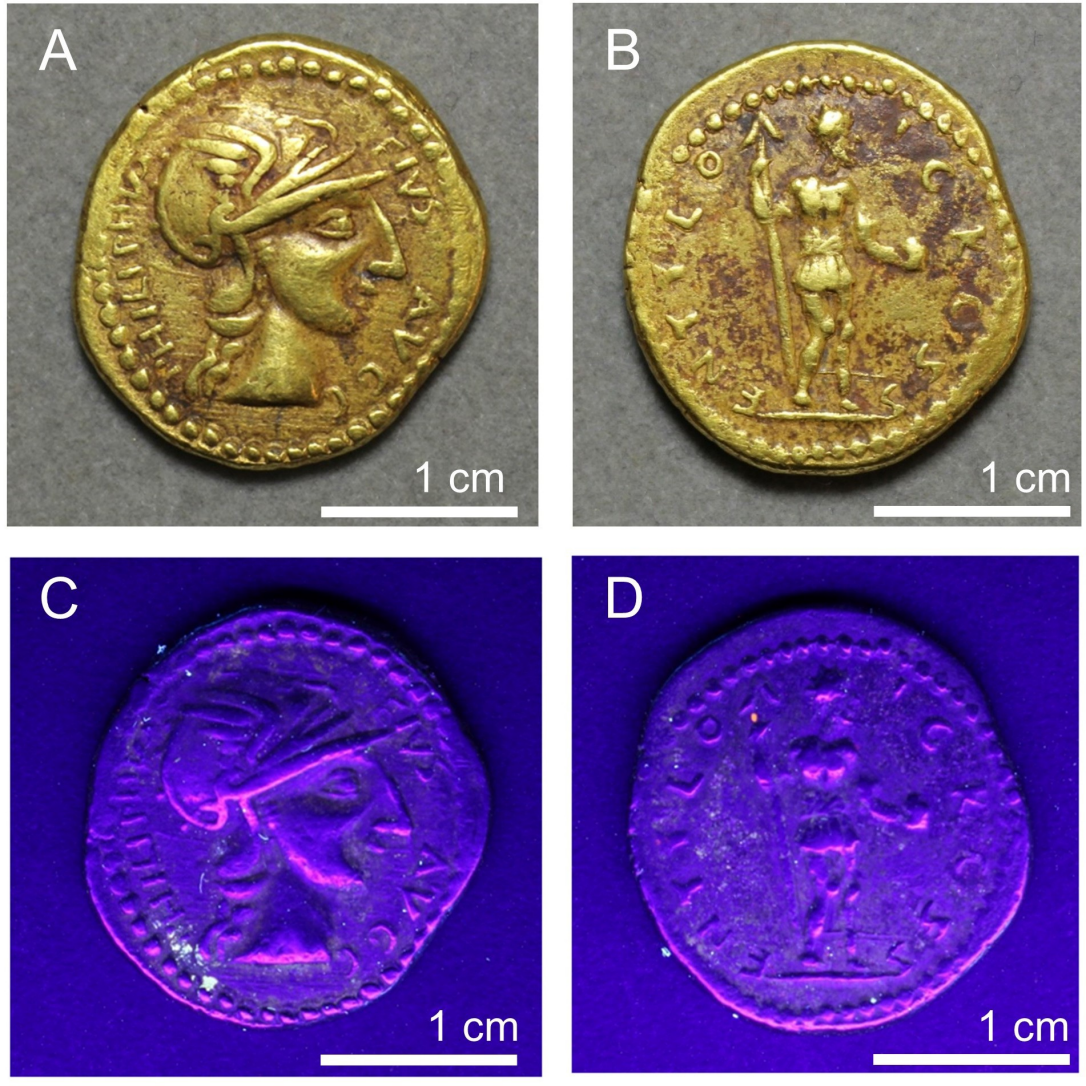
Coin GLAHM:29821 in visible and UV light. Areas of white fluorescence at the bottom of the bust on the obverse and in the left field of the reverse correspond to areas of reddish deposit, although not all such areas fluoresce as brightly. Note the small area of bright orange fluorescence near the spear point which is discussed in the text.

UV photography revealed several areas of white fluorescence on the various coins. Some of these correspond to places where we observe reddish deposits in LM, suggesting the presence of a fluorescent mineral such as calcite (Fig 9). Apart from very bright, white fluorescence visible in some places, most of the coins show a faint, orange glow, a colour that might be distorted away from whiter, paler hues due to the thinness of the glowing layer. This indicates the widespread presence of fluorescent minerals in small patches across the coin surfaces. The amorphous white substance on the Sponsian coin (GLAHM:40333) also fluoresces in white (Supporting Information S.2.6) and was confirmed to be wax by r-FTIR (S.6.6 in S6 File). We speculate that at some stage a wax impression was made of this coin, possibly the cast viewed by Cohen in Paris [8]. A very small ovoid patch on the reverse of one of the Philip coins (GLAHM:29821) below the spearhead fluoresced brightly in orange (see Fig 9D). Analysis by r-FTIR (see S.6.5.2 in S6 File) confirmed this as shellac resin, a substance that is known to fluoresce strongly (e.g., [52]) and is very common in conservation environments: it is interpreted as adventitious.

**Fig 10:**
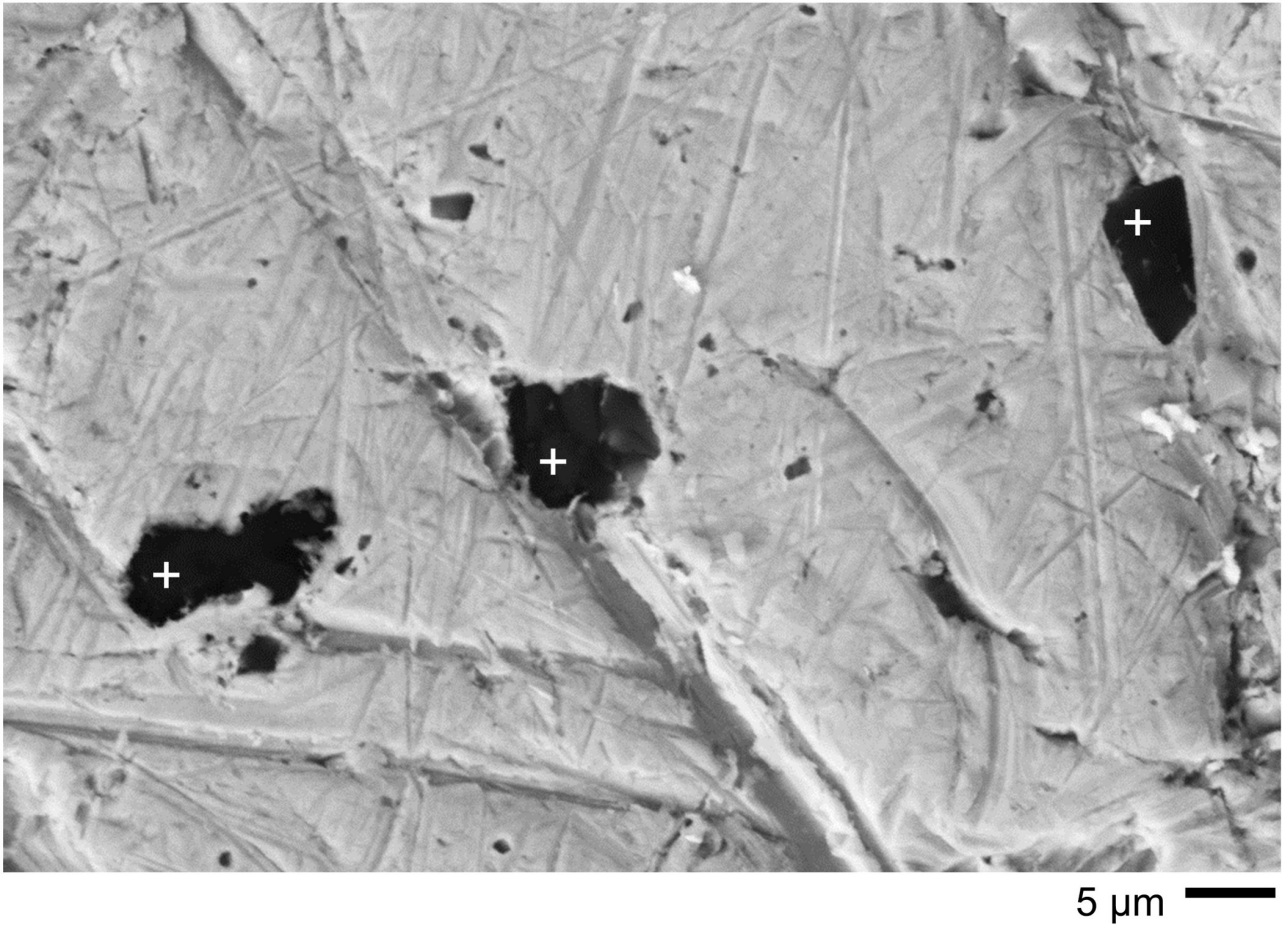
Examples of small patches on the surface of coin GLAHM:40333 by SEM. The spots analysed (crosses) all indicated the presence of Si and O with minor contributions of Au from the underlying metal, suggesting that such patches are cementation spots composed of opaline silica. The three spectra (given in S3 File with data in S4 File) show Si = 46 +/-1.2 wt% and O = 54 +/- 1.2 wt%, consistent with silica.

One very common feature that became apparent during LM imaging was the presence of small irregular dark brown or black patches distributed unevenly over all the coins, including the worn upper areas (see Figs 6 and 8). Imaging by SEM (see Fig 7 and many other images in S3 File) shows that they occur at all scales down to the limit of resolution. They occur in recesses and also on the flat upper surfaces where they overlie the wear scratches in every observed case. A sample area on Coin GLAHM:40333 is shown in Fig 10 with EDX spectra that indicate the presence of Si and O with minor contributions of metals from the underlying surface. We interpret these as spots of opaline silica [53]. Very similar results were obtained from the other coins (see S.3.1.3, S.3.3.3, and S.3.4.2 in S3 File). Larger spots > 5 μm often registered silica with subsidiary cations, especially Al, Fe and Mg (see S.3.1.3, S.3.3.3, and S.3.4.2 in S3 File) hence there is a continuity of appearance from minuscule patches up to distinct composite earthen deposits where granular and crystalline material appears to be cemented by silica.

**Fig 11:**
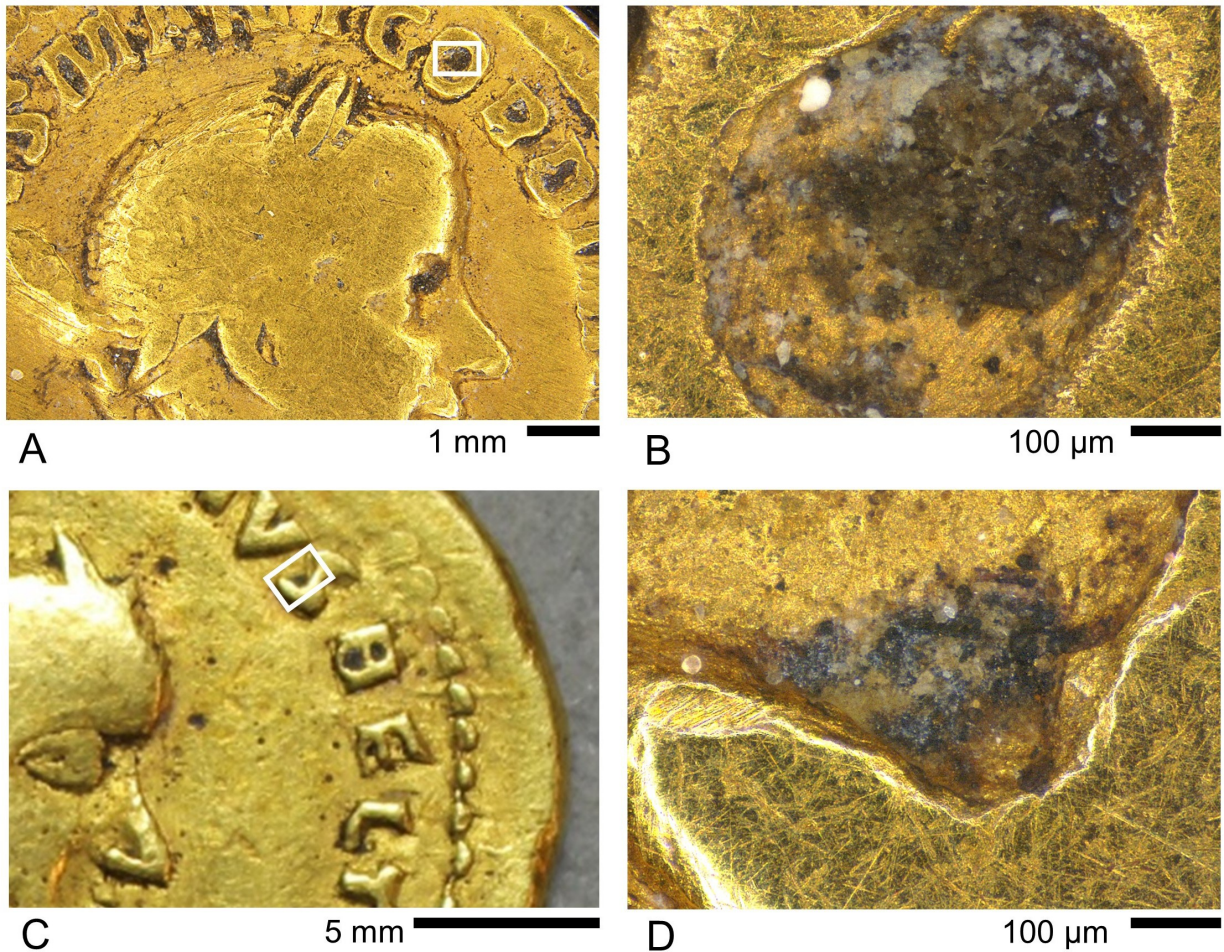
Appearance of larger earthen deposits on a genuine aureus and questionable medallion. A, B) Genuine Gordian III aureus GLAHM:29540; C, D) questionable Gordian III medallion GLAHM:29596.

**Fig 12:**
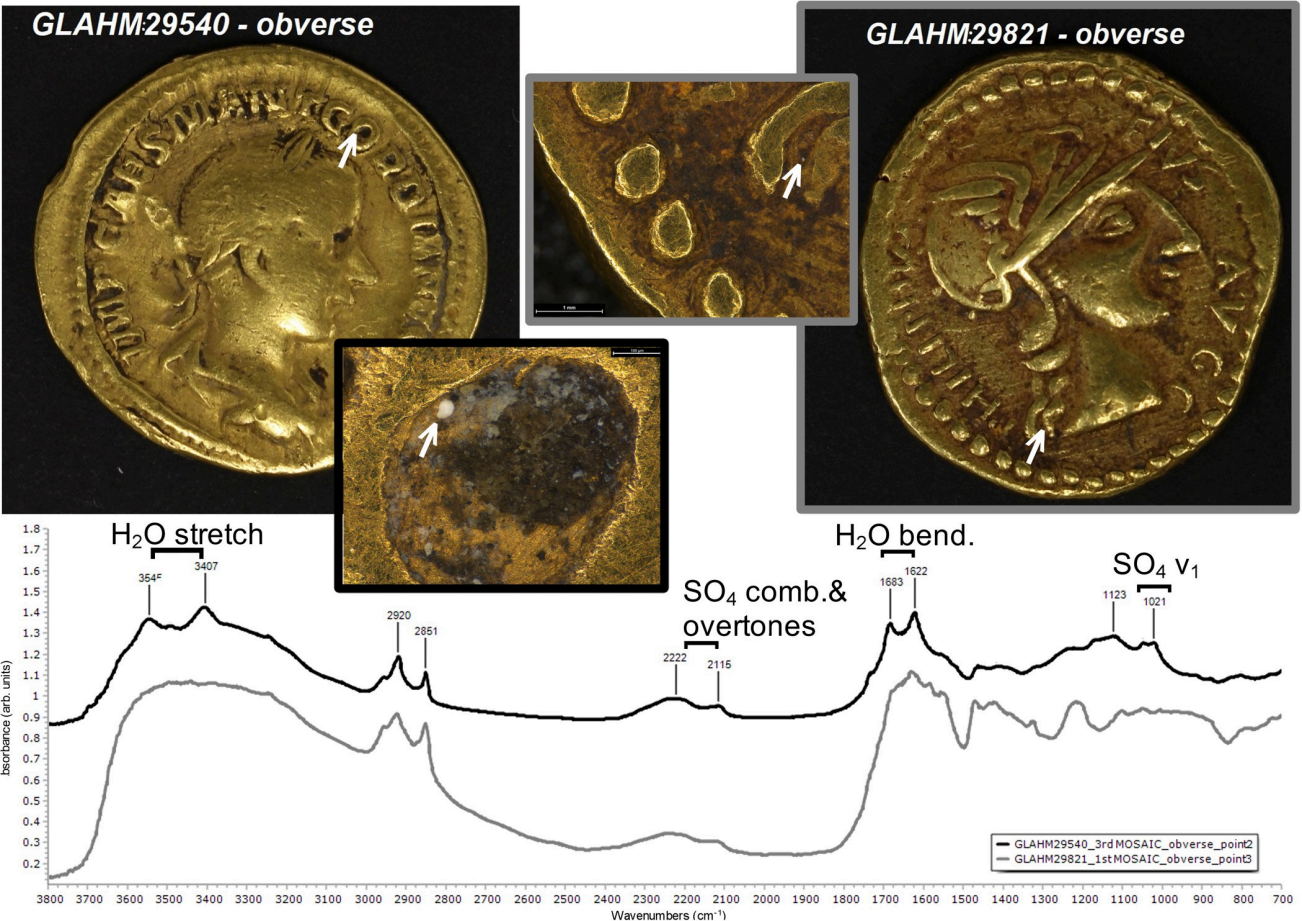
r-FTIR spectrum of light-coloured deposits on the obverse of the genuine Gordian GLAHM:29540 (black) and questionable Philip GLAHM:29821 (grey) coins. The spectra (see arrows for each spot) show gypsum as the predominant compound.

Larger areas of earthen deposit visible to the naked eye occur on the genuine Gordian III aureus (GLAHM:29540) and all four questionable coins (examples are shown in Fig 11; see also Fig 8). These mostly occur in recessed areas such as the lettering. Imaging using LM generally showed an underlying dark heterogeneous mass overlain by cream-coloured material with a variably amorphous or crystalline appearance. Spectroscopic results on Coin GLAHM:29540 using r-FTIR (S.6.1.1 in S6 File) indicated the presence of quartz (SiO_2_), clay minerals, carbonates–probably calcite (CaCO_3_)–and oxalates, which presumably come from soil, as well as gypsum (CaSO_4_·2H_2_O). The picture is further complicated by the presence of an organic compound which is likely calcium distearate, possibly from soap used to clean the coin (albeit not very effectively). Similar results were obtained from all the questionable coins. Typical spectra on a genuine (GLAHM:29540) and questionable (GLAHM:29821) coin are compared in Fig 12 showing the characteristic absorption bands of gypsum. Superficial areas on Coins GLAHM:29821 and GLAHM:40333 (S.6.5 and S.6.6 in S6 File) also showed indications of a potassic sulphate, possibly jarosite (KFe_3_(SO_4_)_2_(OH)_6_).

**Fig 13:**
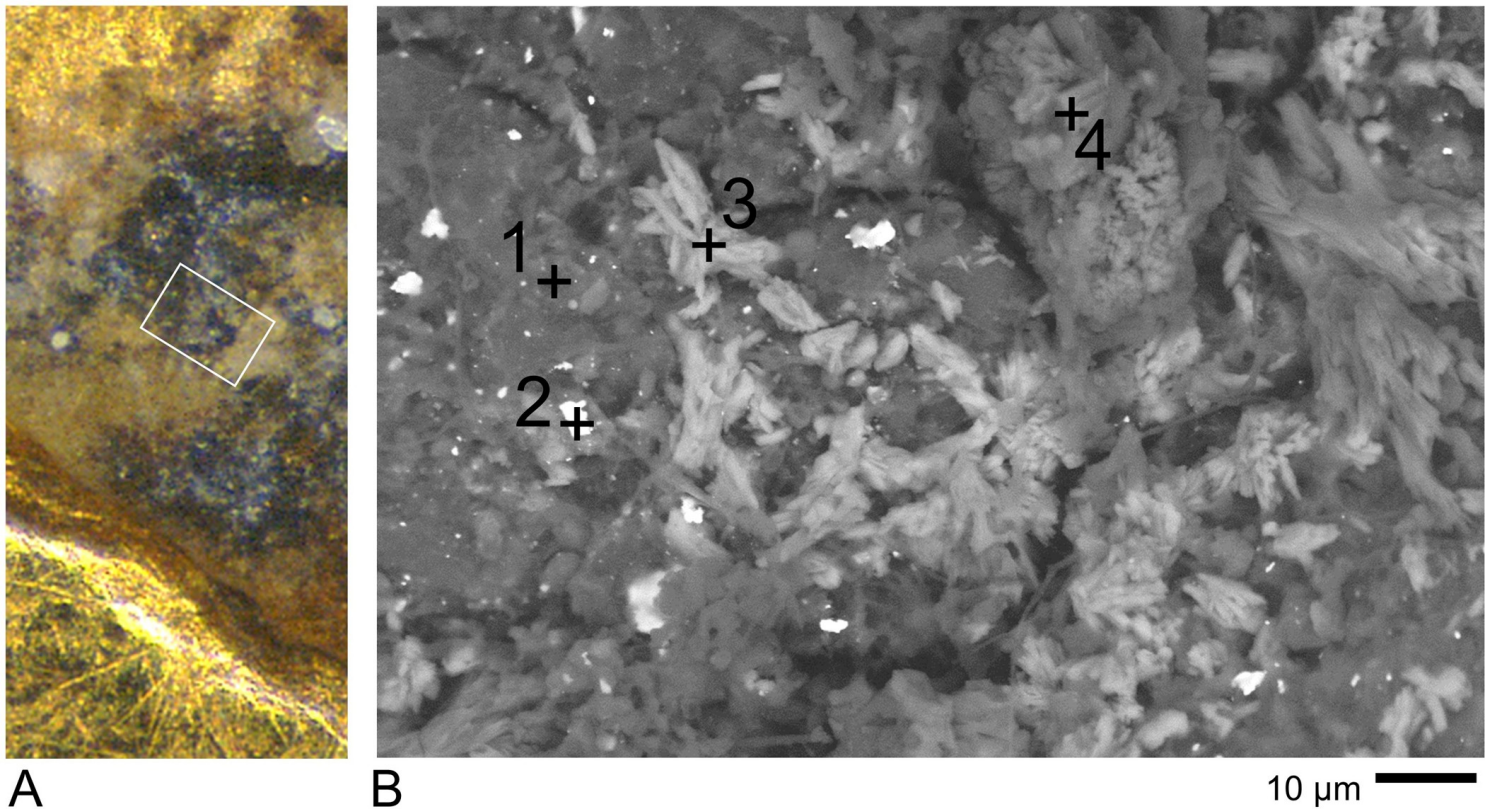
SEM investigation of the earthen deposit on the questionable Gordian III medallion GLAHM:29596. A) LM image showing the area of investigation (compare with Fig 11D); B) SEM image showing a dark material with brighter flecks overlain by lighter crystalline layer. Crosses indicate points where EDX spectra were taken. Point 1 has a complex elemental profile dominated by S and O. Point 2 is a flake of gold, presumably spalled off the surface. Point 3 appears to be a Ca/K sulphate mineral such as syngenite. Point 4 may be calcite and / or gypsum.

The large apparent earthen deposit nestling within the ‘S’ of the questionable medallion of Gordian III (GLAHM:29596) was investigated by SEM-EDX (Fig 13). This showed that the dark underlying material has a complex elemental profile similar to the earthen deposits on the other coins. The overlying lighter-colour patches feature rosettes of lath-shaped crystals, most of which registered Ca, K, S and O, possibly indicating syngenite (K_2_Ca(SO_4_)_2_·H2O). Other crystals in the same area lacked significant K and are interpreted as calcite and/or gypsum.

Finally, a notable feature of questionable coin GLAHM:29821 (Philip I), previously discussed, is a widespread reddish deposit which is closely associated with surface cracking (see Fig 5A and 5E). This area partly fluoresces white in UV indicating a component of calcite (see Fig 9). Imaging indicates that this material is overlain by the darker earthen deposits. Investigation by SEM-EDX yielded results which are consistent with the hypothesis that the reddish material is a residue of the clay casting mould rather than an oxidation product or burial deposit.

## Synthesis and interpretation

### Composition

The minting of gold coins in the Roman Empire was highly centralised, almost always occurring at Rome or, under exceptional circumstances, with the emperor when he was on campaign. However there seems to have been a severe gold crisis in the mid-third century, possibly resulting from reduced output from the mines together with the loss of huge amounts of gold from the treasury, first as an indemnity to the Persians in 244 CE (reviewed in Ref. [1]), and then captured by the Goths at the Battle of Abritus in 251 [1, 54, 55]. It has been noticed that finds of individual stray aurei from the 250s onward become comparatively rare [31, 56], and the weight of individual coins, which had previously been very carefully controlled, began to fluctuate considerably [2, 56]. Nevertheless a very high level of purity was maintained, possibly because merchants across the ancient world were able to detect impurities with surprising accuracy using a simple scratch test [57]. All these factors suggest that after 251 CE, gold became used as bullion, as a means of payment for the elite and a haven of wealth, rather than as an everyday means of exchange against silver and bronze. Meanwhile, inflation became rampant in the wider economy and the silver coinage was rapidly debased [58, 59] so the intrinsic value of silver would also have become detached from the face value of the coinage.

Given these considerations, it is not surprising that the two undoubtedly genuine aurei are very pure gold (see Fig 4). The significant silver and copper content of the questionable coins is further evidence, if it were needed, that they were not minted in ancient Rome. It also seems unlikely that they were made from melted down gold coins. Either they are ‘modern’ forgeries or, if ancient, we suggest they were most likely made from imperfectly refined ore. We note that future analysis of trace elements using other recently developed techniques [e.g., 60, 61] may help fingerprint the metal and identify the source.

The differences in silver and copper content between the three different types of questionable coin (Gordian III, Philip and Sponsian) indicates that they were made in separate batches. The two coins of Philip (Type 4) are sufficiently similar that they may have been made in the same batch although this cannot be known for sure. This might be considered weak evidence in favour of the coins’ authenticity, given that a hypothetical forger would have been likely to have cast all them in one operation.

### Manufacture

Regular Roman coins were made in large secure mint factories by striking pre-prepared blanks between officially sanctioned dies [61]. The dies were made of tough alloys and could be used tens of thousands of times before wearing out. The standard of die engraving was very high because the coins were propaganda tools that reflected the prestige of the emperor and, presumably, also as a security measure to prevent counterfeiting. Nevertheless, unofficial minting and forgery occurred throughout the ancient world and took various forms at different times and for different reasons [34].

Of particular relevance to this study is the widespread striking of gold Roman-style coins in the third and fourth centuries that occurred frequently beyond the empire in eastern Europe, mainly in the area of modern Ukraine, Belarus and Poland. Such ‘barbarous’ coins are generally easy to recognize because they usually lack the deep-relief and high standard of engraving of official issues, the lettering is often poorly executed or meaningless, and many seem to have been intended for use as ornaments with punch holes rather than as currency [5, 62, 63]. The early numismatists classified the Sponsian assemblage as part of this group [12, 13, 18, 23, 24, 26–29, 64] but no other such coins are known to have been cast, and in contrast to the Sponsian group, all known ‘barbarous’ gold imitations are in the weight range of regular aurei, rarely exceeding 6 g [62, 63].

Another important category to consider is the production of illegal counterfeits by casting. These coins were produced by pouring molten base metal into pre-fired clay moulds which had been impressed with a genuine high-value coin as a hub [65]. A thin sheet of silver or gold foil could be compressed onto the surface [34]. The Sponsian group coins are cast, but appear to be made of precious metal throughout, and they have original (and very peculiar) engraved designs, hence they are evidently not counterfeits of this type. We are forced to conclude that either they are outright fakes made to deceive the antiquities market in the eighteenth century [3, 5, 8] or they comprise a unique category of ancient coin.

### Wear

Although the study of coin wear and weight loss was pioneered by luminaries such as Newton, Lavoisier and Cavendish (cited in Ref. [66]), the modern scientific literature is limited. A recent exception is the study of Manas and Velde [66] who studied heavily worn nineteenth century gold coins that were in circulation for up to a century. Those authors showed from SEM imaging that prolonged wear is caused mainly by scratches from grit between coin surfaces (‘three body wear’). This occurs in more or less random orientation and the scratches appear self-similar in length over three orders of magnitude from about 0.5 mm to 0.5 μm. Frequent small craters were also observed by Manas and Velde which they interpreted as due to direct coin-to-coin impact (‘two body wear’).

The wear patterns on the coins of the Sponsian assemblage are similar in all important respects to the heavily worn coins studied by Manas and Velde, most notably the self-similar random scratch patterns down to the highest possible magnification. Indeed, the original upper surfaces of the Sponsian group coins have been entirely worn away. The main difference that we see is more chipping around the lettering, possibly because the surfaces are more brittle than the nineteenth century productions or because they have greater topographic variation. Most of the observed damage is not deep and, in most cases, follows ploughing or wedge forming patterns rather than cutting patterns–i.e. a situation in which the scratches mostly cause displacement of the original material [66, 67]. All coins show wear on the edges, but with a higher ratio of larger shocks to small scratches. That is consistent with the common sense notion that the edges would be largely protected from surface-to-surface grinding against other coins, but more susceptible to sideways collision.

The patterns we observe are consistent with natural wear and there is no obvious evidence for artificial abrasion such as scrubbing or polishing. However it is also possible that these appearances might be simulated. We are told, for instance, that to achieve a realistic and even wear pattern, the notorious nineteenth century forger Wilhelm Becker (1772–1830) placed his fake productions in a box full of iron filings and attached to the axle of his carriage [33]. Similar methods might have been employed by earlier generations of fraudsters. Our own preliminary investigation of suspected forgeries in the Hunter collection suggests that some do indeed have wear scratches, albeit not to the same apparent extent as the deep wear on the four questionable coins. A detailed comparative study of microscopic wear patterns on a range of historical fakes of different types and ages is clearly desirable, but beyond the scope of this investigation. Pending such information, we must view the evidence from wear alone as inconclusive as regards authenticity.

### Burial

The earthen deposits on the questionable and genuine coins are similar in several respects. In all observed cases, they occur above the wear scratches and so must have been formed after the coins had acquired their worn surfaces. They are found in minor recesses across the surfaces and are tightly cemented in place by opaline silica, a ubiquitous binding agent in hydrous soils [53, 68]; and they enclose a variety of mineral grains, the mineralogy and elemental composition of which is indicative of soil. The silica is good evidence that the soil deposits formed in situ, i.e., they were not artificially applied, and there is no evidence of any organic glue. Gypsum occurs atop the larger earthen deposits, sometimes in association with potassic sulphates, which may be jarosite and/or syngenite (see discussion above). Gypsum and jarosite are well-known oxidations products under acidic aqueous conditions of soils originally containing disseminated pyrite and calcite [69, 70]. Syngenite, although less common, has also been observed as a surface weathering product in association with these other sulphates [71]. These observations demonstrate that the earthen deposits formed in an anoxic environment and were subsequently exposed to air. There is also evidence that some of the genuine and questionable coins have been cleaned using soap, leaving probable calcium distearate residues (S.6.5, S.6.6 in S6 File). All these observations are strong evidence that both the genuine and questionable coins have experienced burial, exhumation and cleaning.

How long the questionable coins were buried for is difficult to estimate given the lack of comparative data. Study of coin finds from secure archaeological contexts of different ages and environments may one day help constrain the rate of silica neosynthesis on gold surfaces. The only comparison we can currently draw is with the two genuine Roman aurei in our study, which like the questionable coins are from Hunter’s eighteenth century collection, but are of different, albeit unknown, provenance. Nevertheless they show very similar concentrations and distributions of authigenic minerals on their surfaces to the questionable coins. It is reasonable to conclude, therefore, that the Sponsian group were interred for a prolonged period after they had acquired their worn surfaces, and that the extent of mineral nucleation observed is entirely consistent with burial as long ago as the third century.

### Authenticity

In principle, the Sponsian group coins could have been manufactured at any time between the accession of the Emperor Philip in 244 CE and the first historical record of their existence in 1713. We must, however, allow time for the wear and burial described above. We are unable to devise any remotely plausible scenario that can account for the wear patterns, overlain by cemented earthen deposits, other than that they are products of antiquity. The previous consensus among coin specialists that they were faked in the eighteenth century [3, 5, 8] is clearly untenable.

Authenticity is supported by other more circumstantial arguments; that, as we have shown, the find was reported in 1713 by Johann David von Palm, the senior finance minister responsible for metals and mines, who must have been sufficiently satisfied with the circumstances of their discovery to regard the coins as genuine and suitable for inclusion in the Imperial Cabinet; that the various types of coin were made from compositionally distinct batches of metal; that the subject matter of mixed Republican and Imperial types is an unlikely one for an eighteenth century fraudster, no remotely similar case being known; and that the rare and peculiar name Sponsian is genuinely Roman, although that could not have been known at the time. As such, we conclude they comprise a unique category of ancient coin: heavy cast gold medallions of highly anomalous design that are neither ‘barbarous’ nor counterfeit. Their main significance would appear to be that Sponsian should be rehabilitated as a historical personage. Nothing can be known about him for certain, but the coins themselves, together with the provenance recorded by Heraeus, provide clues as to his possible place in history.

## The historical Sponsian

### Evidence from the coins

Sponsian declared his authority with the legend IMP SPONSIANI wearing the radiate crown of an emperor, thought to denote the power of the sun god, Sol, but more prosaically used on Roman coinage as the symbol for a double denomination. The title IMP is for *imperator* and denotes supreme military command, the root of the English word ‘emperor’. The legend is highly truncated compared to normal Roman coins and is also unique in apparently being in the genitive case (= “of the *imperator* Sponsian”) although that may simply be an eccentricity of the engraver. The legends lack other elements normally used in imperial titulature of the period, most notably abbreviations of CAES(ar), and AVG(ustus). The latter title in particular embodied sweeping civic, religious and legal powers that were distinct, in principle, from military imperium. Curiously, we note that the reverse design from a Republican-era denarius features the legend C AVG which in the original model denotes the moneyer Caius Augurinus, but which would likely have been interpreted by most people in the third century as ‘Caesar Augustus’. It is possible that this was a deliberate act to associate Sponsian with these titles [1], but more likely just coincidence.

The large variation in weight, both between coins of the same type and between the different types, suggests that they could not have a meaningful face value [3] and hence they must have been traded (as the extent of wear indicates they were) as bullion. The most difficult problem to explain about the wider assemblage is why some of the design elements were in faux-Republican style. For this we can posit a tentative explanation, which although speculative, at least provides an account of the sort of thing that might have happened. Official imperial silver had become highly debased by the mid-third century, generally around 40% silver and declining fast thereafter [2, 58, 59]. Writing much earlier, around the end of the first century when the process of debasement was already underway, the historian Tacitus mentioned that the old silver Republican coins were prized outside the empire, presumably for their purity [72]. As discussed above, rapid debasement in the third century promoted a bullion economy within the empire with respect to gold and silver. We propose, therefore, that the intention of the antique iconography was to inspire confidence in the metal.

Intriguingly, the Type 4 obverse (head of Roma) would be a suitable match for the Type 5 Sponsian reverse (Caius Augurinus) except for the lettering naming the emperor Philip which looks squeezed in as an afterthought. This observation hints at a possible sequence of emissions. We suggest that the first issue may have been the wholly Republican Plautius design (Type 2). We can envisage a situation wherein this caused a problem because some people refused to accept the pieces on the grounds that Roman imperial coins almost always featured the bust of an emperor which marked out the money as sacred [73]. A second issue featuring a copy of a Republican Roma / Augurinus coin may have been in preparation but was amended by adding the name of the previous Emperor Philip around the head of Roma, and a newly engraved third century imperial design suitable for Philip was substituted in for the reverse, forming the hybrid Type 4 coin. The wholly Imperial style Type 3 Gordian, which may have constituted the third issue, commemorated the other most recent emperor whose memory was cherished in the area. In this admittedly speculative series, the Type 5 (Sponsian) design may have come last, depicting the man in power but using the reverse hub originally intended for Type 4.

### The Sponsian regime: A hypothesis

Sponsian never controlled an official mint and was unrecorded by all later historians, so he certainly did not rule in Rome. The note of Heraeus describes the find spot as in Siebenburgen (the Habsburg name for Transylvania) [3]. This had been the core of the Roman province of Dacia, the only substantial part of the empire beyond the Danube, which had been conquered by the Emperor Trajan (r. 98–117 CE) for its mineral resources. Because of its exposed position it was heavily militarised with two large legionary bases (Apulum and Potaissa) and numerous auxiliary forts. The total garrison has been estimated at about 50,000 at its peak [74–76]. Many towns and villages developed in the valleys near the mining district and military bases, supplying food, livestock, ceramics, textiles and other local produce. But by the mid-third century the area had become increasingly isolated and surrounded by hostile peoples (Sarmatians, Goths and Carpi) who mounted frequent raids, and the legions had no doubt became depleted by the near-constant warfare since the mid 240s [1].

Most of the early writers who considered Sponsian as a historical usurper (e.g., Ref. [28]) assigned him to the period of strife in the last years of the emperor Philip (around 248 CE) when other short-lived rebellions occurred elsewhere. However it seems unlikely to us that Philip would have featured on the coins of such a rebel. A more likely time is the 260s, during the sole reign of the Emperor Gallienus (260–268), at which time the empire effectively disintegrated into three large chunks. The provinces south of the Danube which ordinarily provided the lifeline for Dacia had become devastated and depopulated by relentless foreign invasions and civil wars [1, 75]. We suggest that Dacia became cut off from the imperial centre around 260 and effectively seceded under its own military regime which initially coined precious metal bullion using old Republican-era designs, then using the names of the most recent previous emperors who had achieved some success in the area, and finally under the name of a local commander-in-chief.

This hypothesis is helpful in several ways. Most significantly, there was an abundant supply of precious metals from the many mines operating in Dacia. Archaeology shows that the gold ore was processed on site [77]. In normal times ingots would have been shipped to Rome with coinage coming back to pay for the operation and the large military presence. This, however, required secure supply lines, whereas archaeological data from coin finds across Dacia indicates that the external money supply terminated around the year 260 [78, 79]. An obvious expedient would have been to use the metal from the mines to pay the troops until order was restored. The only official mint in Dacia, which had previously manufactured large quantities of bronze coins for the local economy, had been shut down in the mid-250s as the crisis developed [79]. There was, however, an artisan jewellery industry at Apulum working in gold using clay moulds [80], so we suggest that the person who manufactured the coins was co-opted from there, using the technique familiar to them.

This also helps explain the relatively high silver content of the coins. Romania’s Apuseni Mountains contain Europe’s largest gold field, but the ore there is also notably rich in silver [77]. The local cupellation process, which separated the precious metals, may have had practical limits of purity below that achievable by the industrial smelters of Rome. The level of silver and copper in the metal, and the range of variation, is very similar to that measured in first century Dacian-era gold objects made in the same area [81]. Silver would have been separated from the ore in significantly larger amounts than gold, hence reports of possible silver coins in the Sponsian series (Types 6 and 7) [8–10] suggest that it was turned to coin also, although no such pieces apparently became a part of the hoard discovered in 1713. We note that the large Type 7 coin featuring Gordian III, now lost, was described as being of pure silver [8], which is quite unlike the regular debased coins of the period, but would fit with the idea that it was intended as bullion.

So, to develop the hypothesis, we suggest that the Sponsian series coinage was used to pay senior soldiers and officials in gold and silver by weight and then traded down at a high premium for regular imperial coins that were already circulating in the province from before the time of crisis. As the only source of new money, the coins would necessarily have gone into active use rather than being used as a store of wealth, which would explain their heavily worn condition. Technically, this process can be considered as a type of quantitative easing, a monetary stimulus that might have prevented the local economy from collapse as well as avoiding military revolt. The state of wear on the coins suggests that the policy was successful. The total volume of original coinage necessary to achieve this is difficult to estimate, but to have been effective it was presumably far larger than the handful of coins that survive today.

For this reason we predict that at some point a Sponsian group coin will be discovered in a secure archaeological context. Indeed it is surprising that no well-attested find of this type has been made in modern times (one of the more compelling reasons they have been regarded as fakes). One way to explain this is to consider what may have happened when the province of Dacia was formally abandoned by the Emperor Aurelian, either in 271 or 275 CE [75, 82–84]. Historical sources (which, for this period, are very short summaries) say that Aurelian organized an orderly withdrawal of the military and civilian population to a newly formed province on the south side of the Danube. There is no suggestion that he conducted a military campaign against a rebel regime in Dacia, as he did both in Gaul and the east [82, 83], so it seems likely that the authorities in Dacia cooperated with this policy. We suggest that nearly all the precious metal in circulation was recalled and exchanged for official currency during the evacuation. The 1713 group may have been buried by a wealthy individual who, for whatever reason, failed to join the exodus.

This scenario may also explain an apparent contradiction in the historical sources that has long puzzled historians (reviewed in [85]). These say on the one hand that the Province of Dacia was lost to the empire in the reign of Gallienus, but also that it was Aurelian who evacuated the people and soldiers to a new province (which he called Dacia for propaganda purposes) south of the Danube, formed out of depopulated areas that had previously been part of the Provinces of Upper and Lower Moesia and a district further south known as Dardania (Table 4). It has therefore been suggested that Dacia may have been abandoned in two stages, but a simpler way of reconciling these statements is to envisage an initial period of isolated secession when supply lines were cut off, followed by a negotiated and orderly withdrawal across the river when the security situation improved.

**Table 4:** Historical accounts pertaining to the loss and abandonment of Transdanubian Dacia in English translation. In chronological order, the surviving sources are Flavius Vopiscus (ostensibly written in 303 CE, although considered late fourth century by most other scholars); Aurelius Victor (361 CE), Eutropius (circa 370 CE); Festus (also circa 370 CE); Orosius (circa 417 CE); and Jordanes (circa 551 CE). Obvious similarities in some of the accounts suggest various authors were either working from common sources or from each other.

**The loss of Dacia under Gallienus (r. 260–268 CE):**
“He shipwrecked the Roman state, so to speak… Even the territories across the Danube, which Trajan had secured, were lost.” Aurelius Victor, *De Caesaribus* 33 [85].
“Dacia, which had been added beyond the Danube by Trajan, was lost at that time.” Eutropius, *Breviarum* 9.8.1 [86].
“It [Dacia] was lost under Emperor Gallienus” Festus, *Breviarum* 8 [87].
“Dacia beyond the Danube was lost forever” Orosius, *Historiam Adversum Paganos* 7.22 [88].
“Gallienus, while he was ruling, lost them [the provinces of Dacia]” Jordanes, *Romana* 217 [89].
**The abandonment of Dacia under Aurelian (r. 270–275 CE):**
“On seeing that Illyricum was devastated and Moesia was in a ruinous state, he [Aurelian] abandoned the province of Trans-Danubian Dacia, which had been formed by Trajan, and led away both soldiers and provincials, giving up hope that it could be retained. The people whom he moved out from it he established in Moesia, and gave to this district, which now divides the two provinces of Moesia, the name of Dacia.” Flavius Vopiscus, *Divus Aurelianus*, 13.7 [90].
“He [Aurelian] gave up the province of Dacia, which Trajan created beyond the Danube, since the whole of Illyricum and Moesia had been devastated and he despaired of being able to retain it, and he withdrew the Romans from the cities and countryside of Dacia, and resettled them in the middle of Moesia and called it Dacia, which now divides the two Moesias and is on the right bank of the Danube as it flows to the sea, where it was previously on the left”. Eutropius, *Breviarum* 9.15 [86].
“After Romans had been transferred from there [Dacia] by Aurelian, two Dacias were made in the region of Moesia and Dardania.” Festus, *Breviarum* 8 [87].
“Emperor Aurelian, recalling the legions thence, stationed them in Moesia, and there made part of it into ‘Mediterranean Dacia’ and ‘Riverbank Dacia’, and attached Dardania.” Jordanes, *Romana* 217 [89].

The two Dacian legions (XIII Gemina and V Macedonica) had remained loyal to Rome in the civil wars of the late 250s and were awarded the titles *pia* (dutiful) and *fidelis* (faithful) on multiple occasions in that period [1]. They also occur in a special series of propaganda coins issued by Gallienus in 260 that feature all the legions that were under his control at that time [91]. Indeed, we note they are marked out as special because they alone have their legionary symbols attended by the goddess Victory, apparently signifying some recent military success achieved in unison. We suggest that Sponsian may have been the commanding officer (*dux*) of these legions and the combined forces of Dacia, and that he led a secessionist regime within a time window extending from 260 to the mid-270s at a time when most of the rest of the empire was wracked by civil war and collapsed frontiers, and secure communication with Rome was impossible. His priority would have been to protect the population and resist being over-run by hostile tribes. In this scenario, he was not technically a usurper challenging central authority, but his imperium might be considered a local necessity.

## Supporting information

S1 File Photography and light microscope (LM) imaging. (PDF)

S2 File Ultraviolet (UV) imaging. (PDF)

S3 File Scanning electron microscopy (SEM) and Energy Dispersive X-ray (EDX) spectra. (PDF)

S4 File Energy Dispersive X-ray (EDX) data. (XLSX)

S5 File Metal bulk composition analyses. (XLSX)

S6 File Reflection mode Fourier Transform Infra-red spectroscopy (r-FTIR). (PDF)
