# Supplementary material for: Authenticating coins of the ‘Roman emperor’ Sponsian
Source: PLoS One. 2022 Nov 23;17(11):e0274285. doi: 10.1371/journal.pone.0274285 (PMC9683583; doi:10.1371/journal.pone.0274285)
Supplement: S1 File — (PDF) [file pone.0274285.s001.pdf]

Supporting Information 1

Photography and light microscope (LM) imaging

Contents

S.1.1 Coin GLAHM:29540 (Genuine Gordian III aureus) 5

S.1.1.1 Whole coin photographs 5

Figure S.1. 1 Obverse of Coin GLAHM:29540 5

Figure S.1. 2 Reverse of Coin GLAHM:29540 5

S.1.1.2 Standardised images 6

Figure S.1. 3 Obverse, exposed area, emperor's forehead. 6

Figure S.1. 4 Obverse, flat field, in front of emperor's mouth. 6

Figure S.1. 5 Reverse, exposed area, shoulder of figure 7

Figure S.1. 6 Reverse, flat field, to left of figure's shoulders 7

Figure S.1. 7 Edge, first image 8

Figure S.1. 8 Edge, second image 8

Figure S.1. 9 *Edge, third image* 9

1.1.3 Additional images 9

Figure S.1. 10 Detail of reverse gouge 9

Figure S.1. 11 Detail of obverse showing scratches and earthen deposits 10

Figure S.1. 12 Detail of earthen deposit in obverse letter 'O' 10

S.1.2 Coin GLAHM:29697 (Genuine Philip I aureus) 11

S.1.2.1 Whole coin photographs 11

Figure S.1. 13 Obverse of Coin GLAHM:29697 11

Figure S.1. 14 Reverse of Coin GLAHM:29697 11

S.1.2.2 Standardised images 12

Figure S.1. 15 Obverse, exposed area, emperor's cheekbone 12

Figure S.1. 16 Obverse, flat field, in front of emperor's eye 12

Figure S.1. 17 Reverse, exposed area, shoulder of goddess 13

Figure S.1. 18 Reverse, flat field, in behind goddess's shoulder 13

Figure S.1. 19 Edge, first view 14

Figure S.1. 20 Edge, second view 14

Figure S.1. 21 Edge, third view 15

S.1.2.3 Additional images 15

Figure S.1. 22 Detail of reverse showing scratches and fractures 15
S.1.3 Coin GLAHM:29596 (Questionable Gordian III medallion / binio) 16
S.1.3.1 Whole coin photographs 16
Figure S.1. 23 Obverse of Coin GLAHM:29596 16
Figure S.1. 24 Reverse of Coin GLAHM:29596 16
S.1.3.2 Standardised images 16
Figure S.1. 25 Obverse, exposed area, to eft of emperor's eye 17
Figure S.1. 26 Obverse, flat field, to right of emperor's nose 17
Figure S.1. 27 Reverse, exposed area, on skirt of goddess 18
Figure S.1. 28 Reverse, flat field, in front of goddess's forehead 18
Figure S.1. 29 Edge, first image 19
Figure S.1. 30 Edge, second image 19
Figure S.1. 31 Edge, third image 20
1.3.3 Additional images 20
Figure S.1. 32 Detail of scratches and earthen deposits in angled light 20
Figure S.1. 33 Detail of earthen deposit beside letter 'S' on obverse 21
Figure S.1. 34 Detail of grooves in protected area of reverse field 21
S.1.4 Coin GLAHM:29820 (Questionable Philip I medallion) 22
S.1.4.1 Whole coin photographs 22
Figure S.1. 35 Obverse of Coin GLAHM:29820 22
Figure S.1. 36 Reverse of Coin GLAHM:29820 22
S.1.4.2 Standardised images 23
Figure S.1. 37 Obverse exposed area, cheekbone of Roma 23
Figure S.1. 38 Obverse flat field to the right of the letter 'H' 23
Figure S.1. 39 Reverse, exposed area, emperor's tunic 24
Figure S.1. 40 Reverse, flat field, to left of spear and emperor's waist 24
Figure S.1. 41 First edge view 25
Figure S.1. 42 Second edge view 25
Figure S.1. 43 Third edge view 26
S.1.4.3 Additional images 26
Figure S.1. 44 Detail of obverse showing earthen deposits in helmet 26
Figure S.1. 45 Detail of obverse showing earthen deposit in 'V'-shape gouge 27
S.1.5 Coin GLAHM:29821 (Questionable Philip I medallion) 28
S.1.5.1 Whole coin photographs 28

Figure S.1. 46 Obverse of Coin GLAHM:29821 28
Figure S.1. 47 Reverse of Coin GLAHM:29821 28
S.1.5.2 Standardised images 29
Figure S.1. 48 Obverse raised area, emperor's cheek 29
Figure S.1. 49 Obverse flat field, to left of emperor's head 29
Figure S.1. 50 Reverse raised area, emperor's tunic 30
Figure S.1. 51 Reverse flat field, to right of emperor's tunic 30
Figure S.1. 52 First edge view 31
Figure S.1. 53 Second edge view 31
Figure S.1. 54 Third edge view 32
1.5.3 Additional images 32
Figure S.1. 55 Detail of obverse showing earthen deposits in helmet 32
Figure S.1. 56 Detail of reverse side showing reddish areas 33
Figure S.1. 57. Detail of reverse side showing cracking of surface and reddish deposit 33
Figure S.1. 58 Detail of reverse side showing reddish areas and earthen deposits 34
Figure S.1. 59 Detail of reverse side showing incomplete casting 34
S.1.6 Coin GLAHM:40333 (Questionable Sponsian medallion) 35
S.1.5.1 Whole coin photographs 35
Figure S.1. 60 Obverse of Coin GLAHM:40333 35
Figure S.1. 61 Reverse of Coin GLAHM:40333 35
1.6.2 Standardised images 36
Figure S.1. 62 Obverse exposed area, emperor's head to right of ear 36
Figure S.1. 63 Obverse flat field, near lowest ray of emperor's crown 36
Figure S.1. 64 Reverse exposed area, lower right of wheat ear 37
Figure S.1. 65 Reverse recessed area, to left of waist of standing figure 37
Figure S.1. 66 First edge view 38
Figure S.1. 67 Second edge view 38
Figure S.1. 68 Third edge view 39
S.1.6.3 Additional images 39
Figure S.1. 69 Detail of obverse showing scratches and wax 39
Figure S.1. 70 Detail of obverse showing scratches under first directional illumination 40
Figure S.1. 71 Detail of obverse showing scratches under second directional illumination 40

Figure S.1. 72 Detail of earthen deposit on obverse 41
Figure S.1. 73 Detail of earthen deposit on obverse 41
Figure S.1. 74 Detail of beading, wax and earthen deposit on obverse 42
Figure S.1. 75 Detail of lettering, fractures and earthen deposit on obverse 42
Figure S.1. 76 Detail of flat field in reverse 43
Figure S.1. 77 Detail of lines in flat field on reverse 43

S.1.1 Coin GLAHM:29540 (Genuine Gordian III aureus)

S.1.1.1 Whole coin photographs

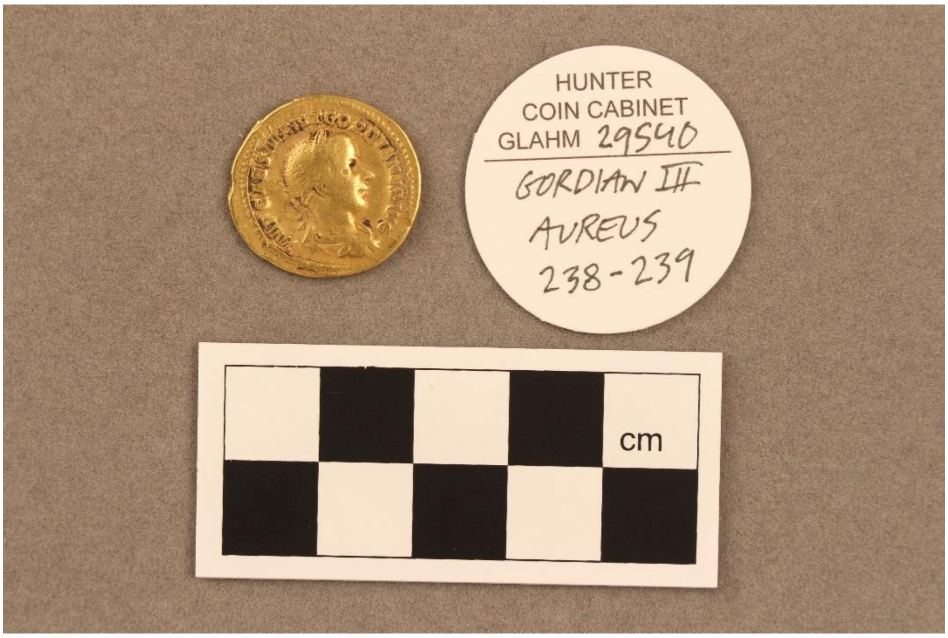

Figure S.1. 1 Obverse of Coin GLAHM:29540

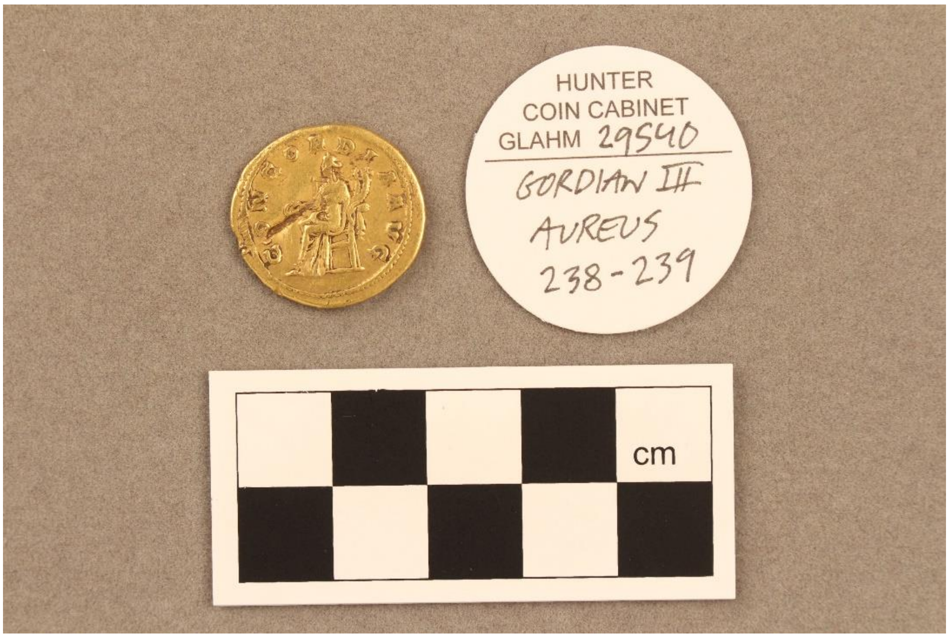

Figure S.1. 2 Reverse of Coin GLAHM:29540

S.1.1.2 Standardised images

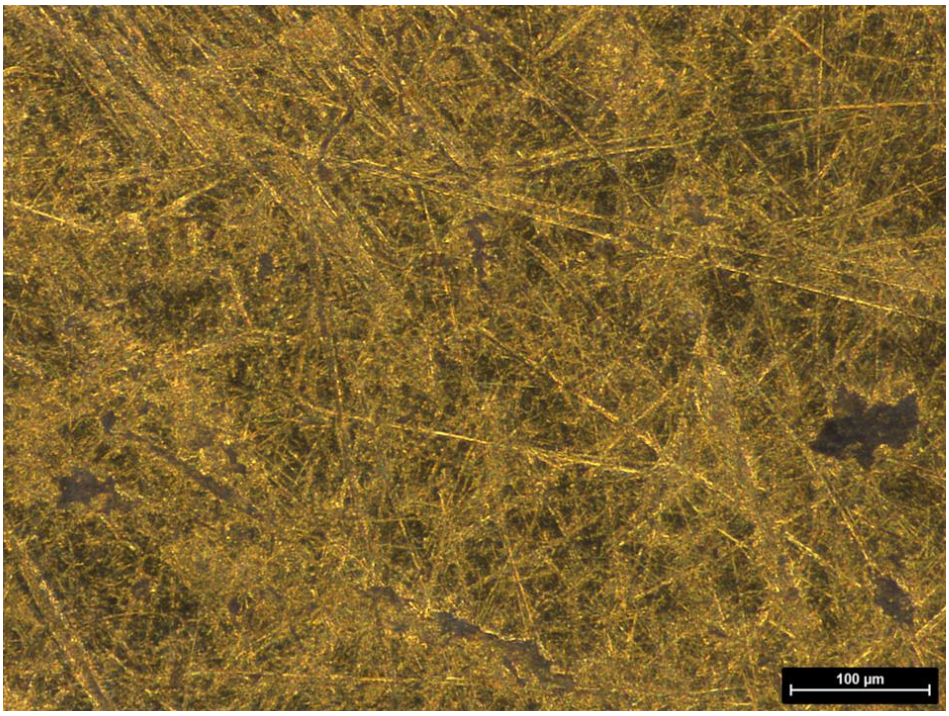

Figure S.1. 3 Obverse, exposed area, emperor's forehead.

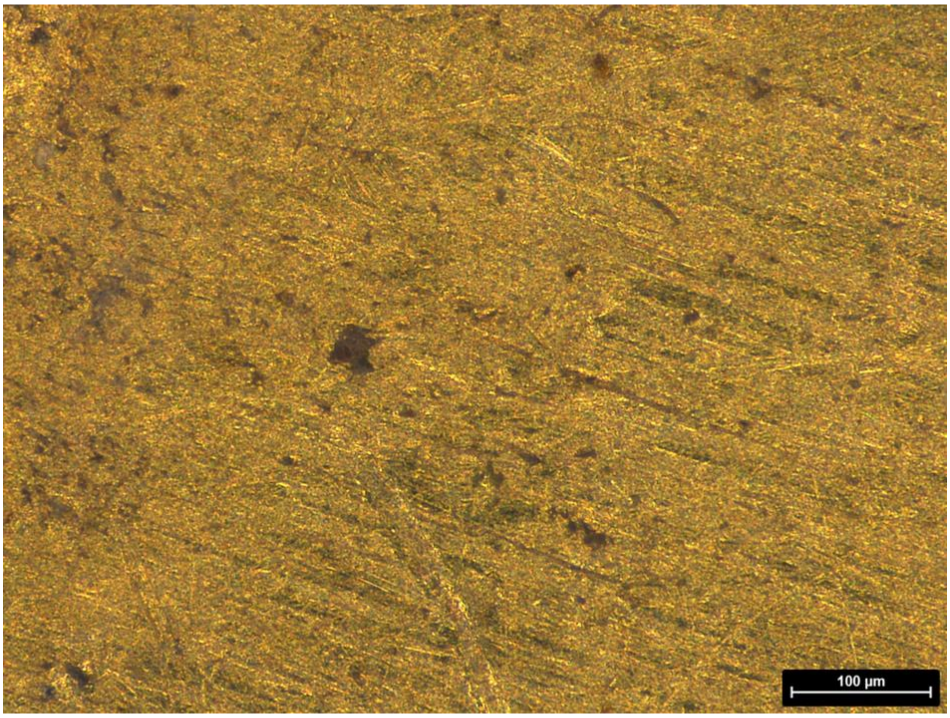

Figure S.1. 4 Obverse, flat field, in front of emperor's mouth.

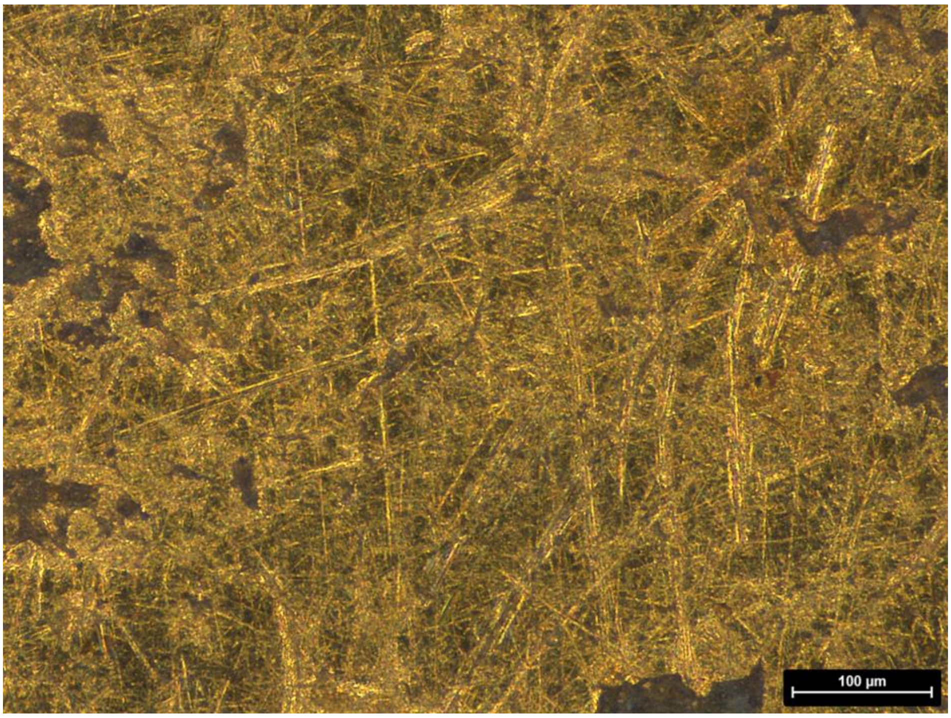

Figure S.1. 5 Reverse, exposed area, shoulder of figure

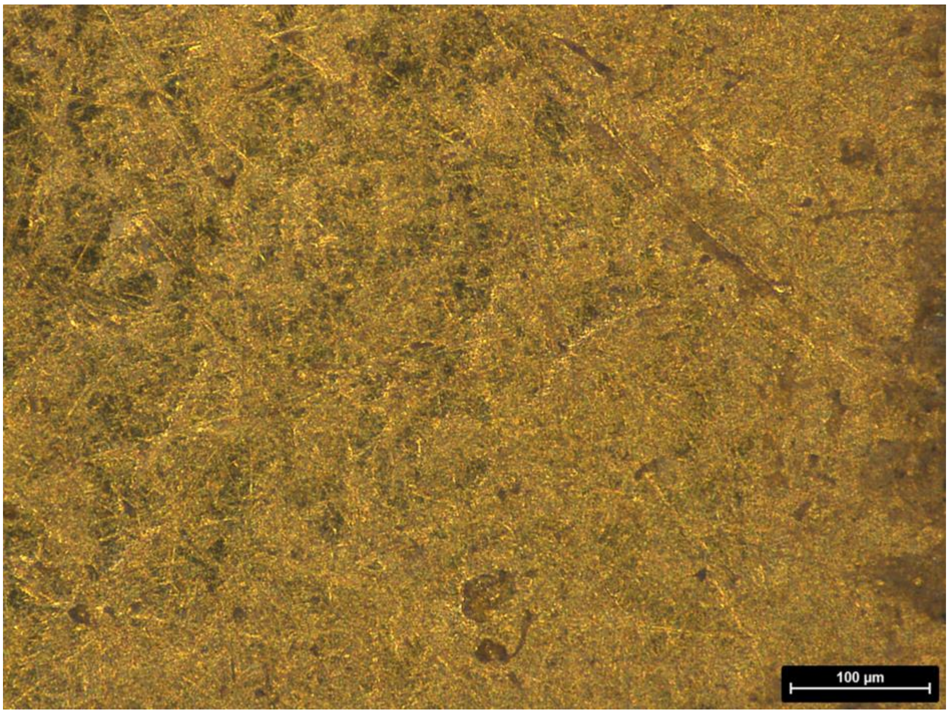

Figure S.1. 6 Reverse, flat field, to left of figure's shoulders

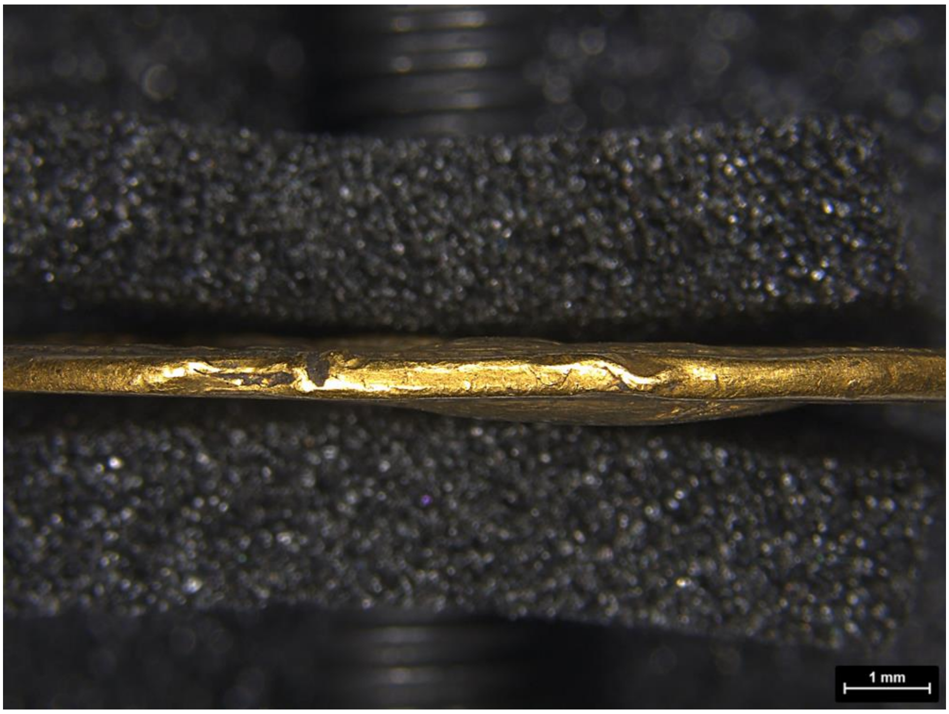

Figure S.1. 7 Edge, first image

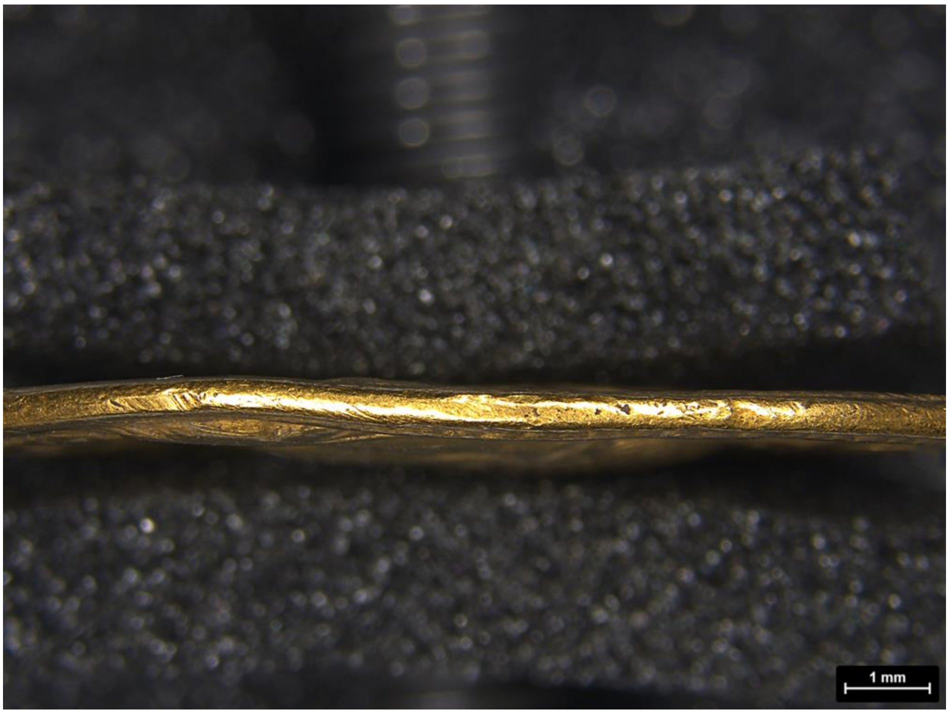

Figure S.1. 8 Edge, second image

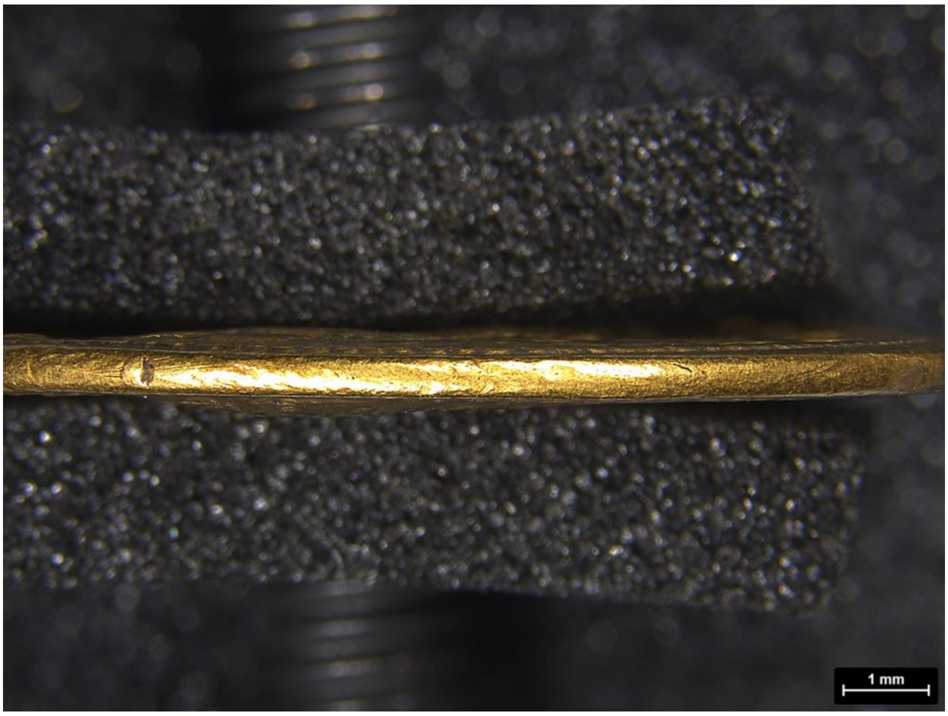

Figure S.1. 9 Edge, third image

1.1.3 Additional images

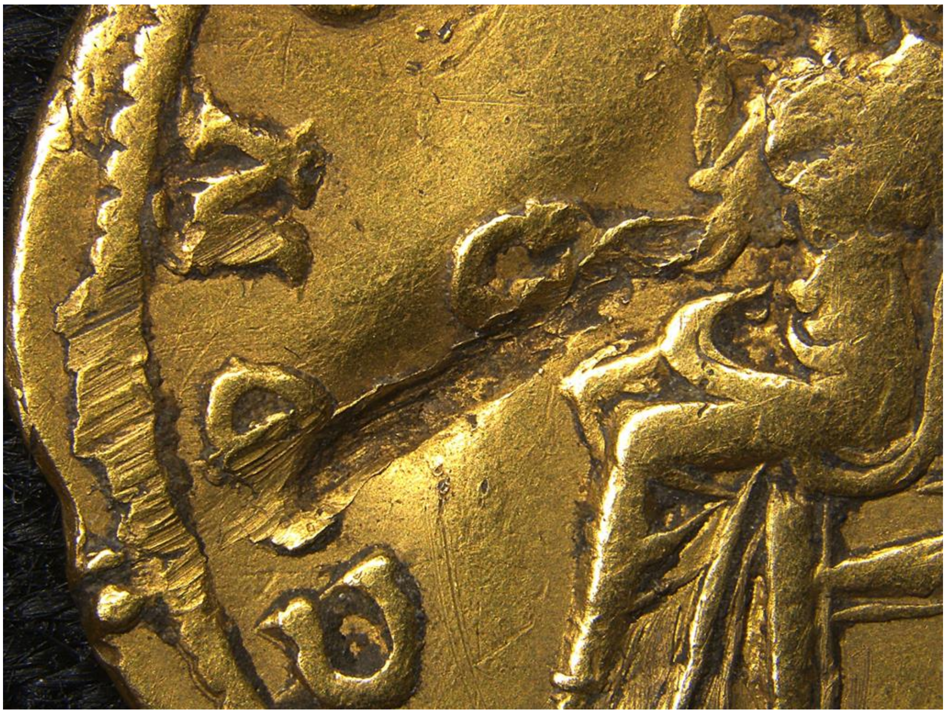

Figure S.1. 10 Detail of reverse gouge

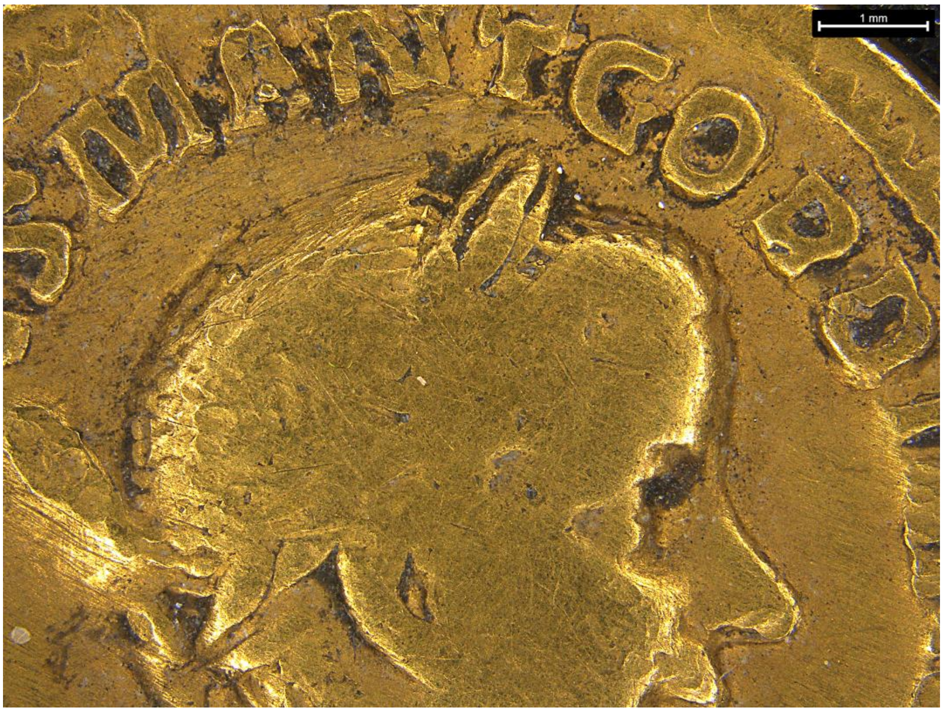

Figure S.1. 11 Detail of obverse showing scratches and earthen deposits

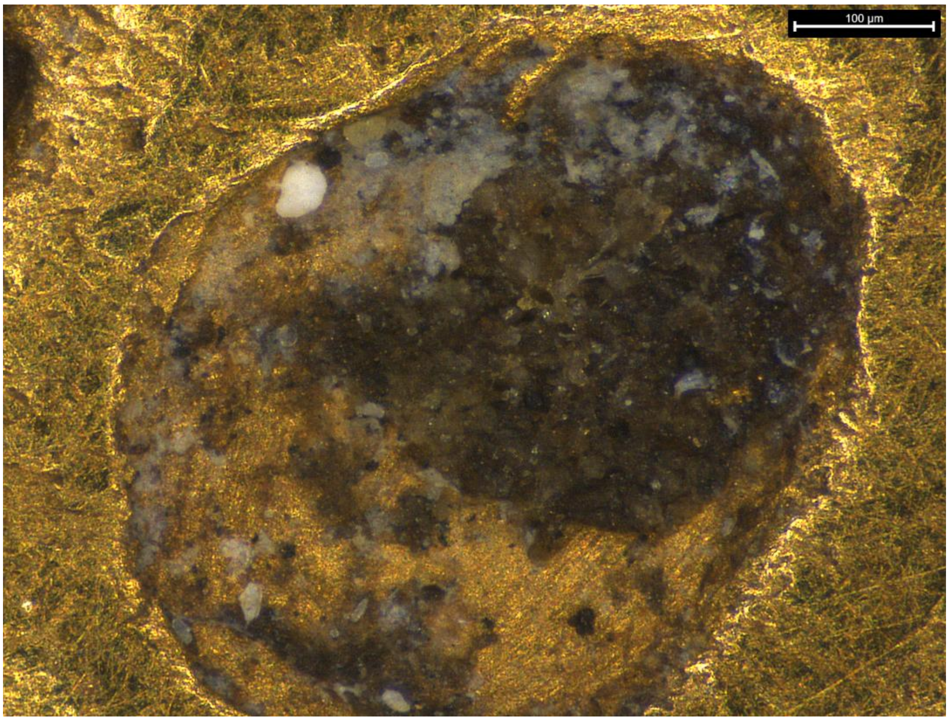

Figure S.1. 12 Detail of earthen deposit in obverse letter 'O'

S.1.2 Coin GLAHM:29697 (Genuine Philip I aureus)

S.1.2.1 Whole coin photographs

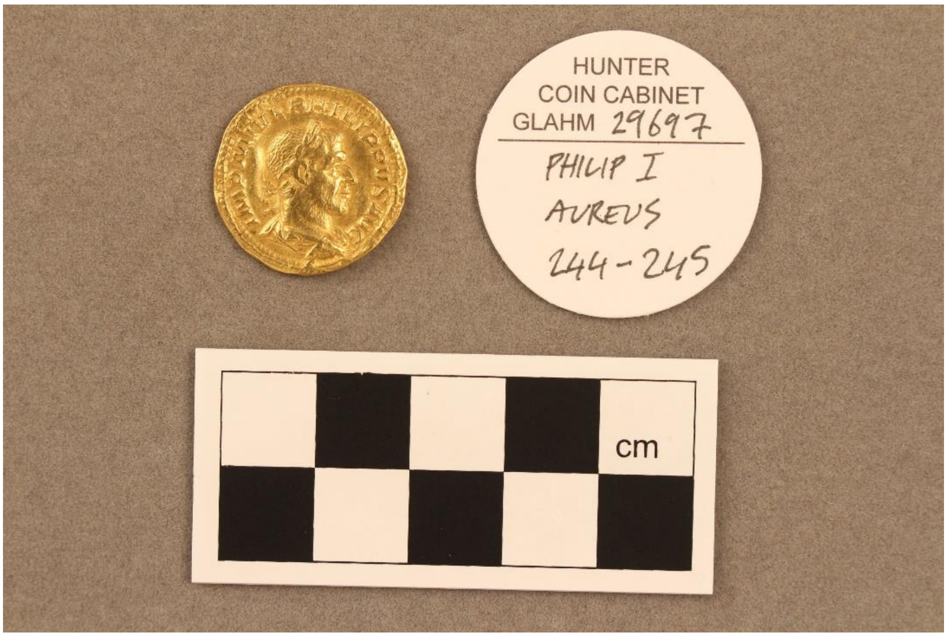

Figure S.1. 13 Obverse of Coin GLAHM:29697

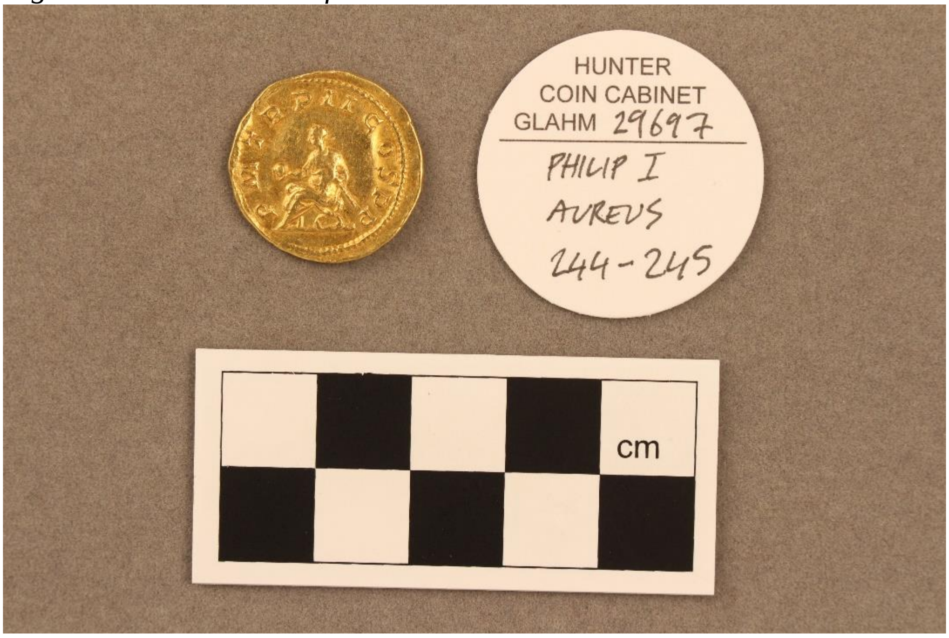

Figure S.1. 14 Reverse of Coin GLAHM:29697

S.1.2.2 Standardised images

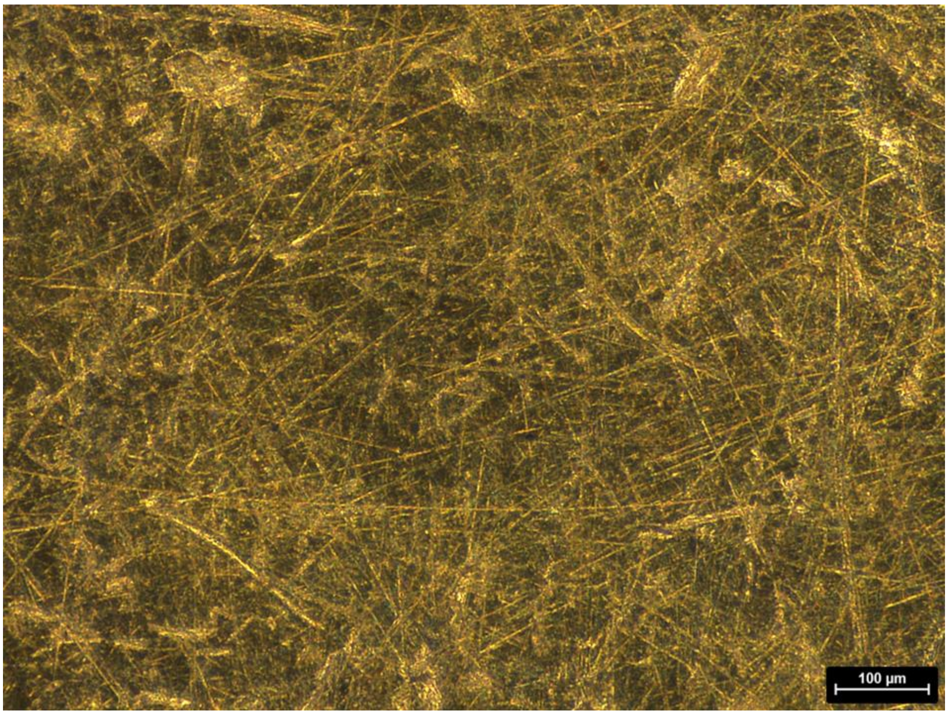

Figure S.1. 15 Obverse, exposed area, emperor's cheekbone

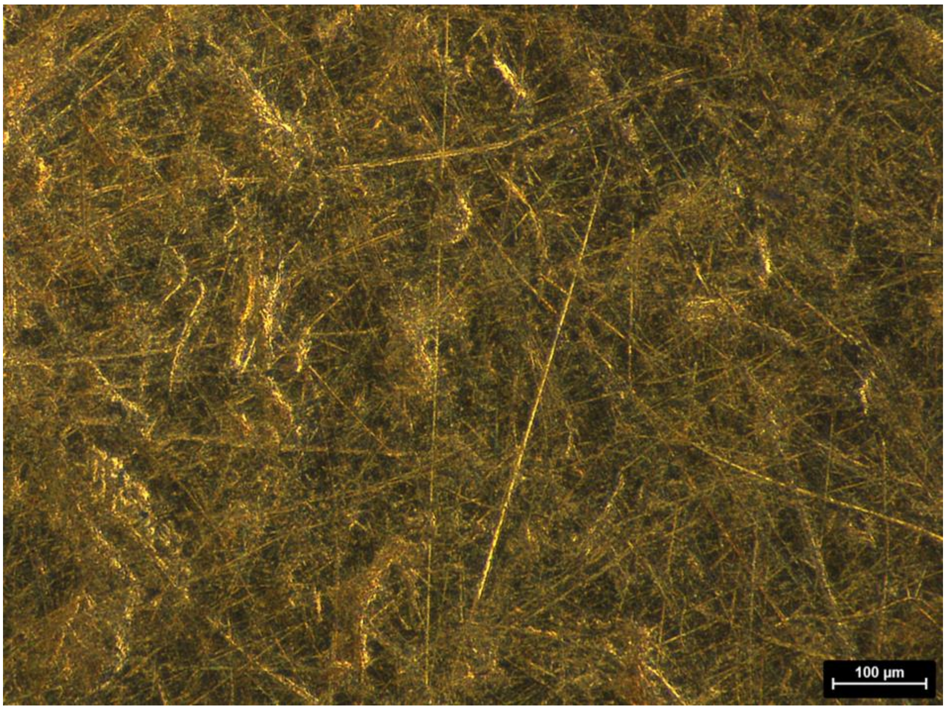

Figure S.1. 16 Obverse, flat field, in front of emperor's eye

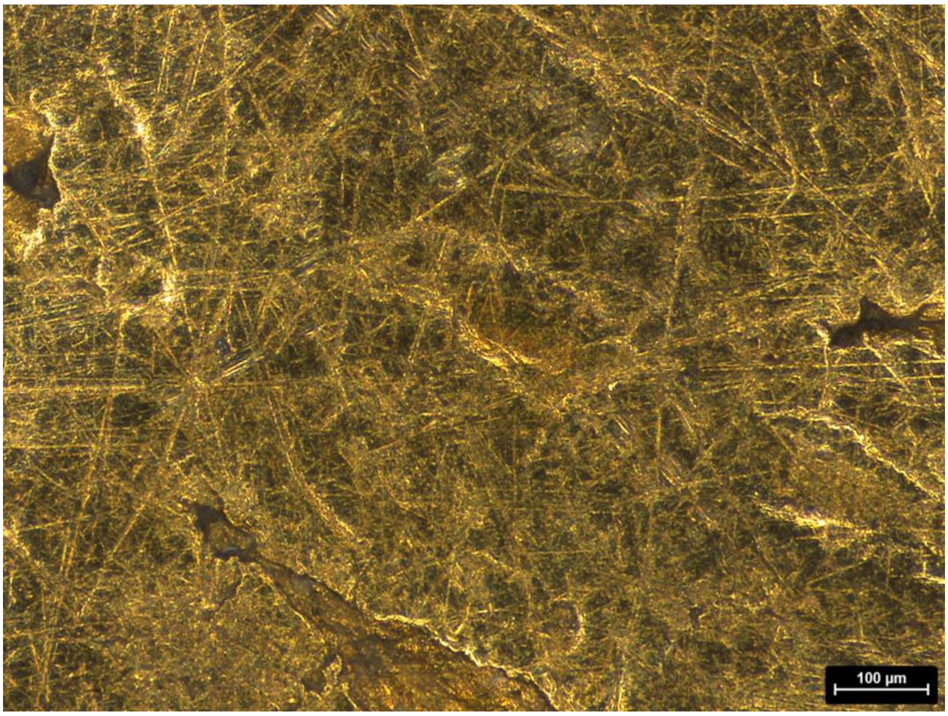

Figure S.1. 17 Reverse, exposed area, shoulder of goddess

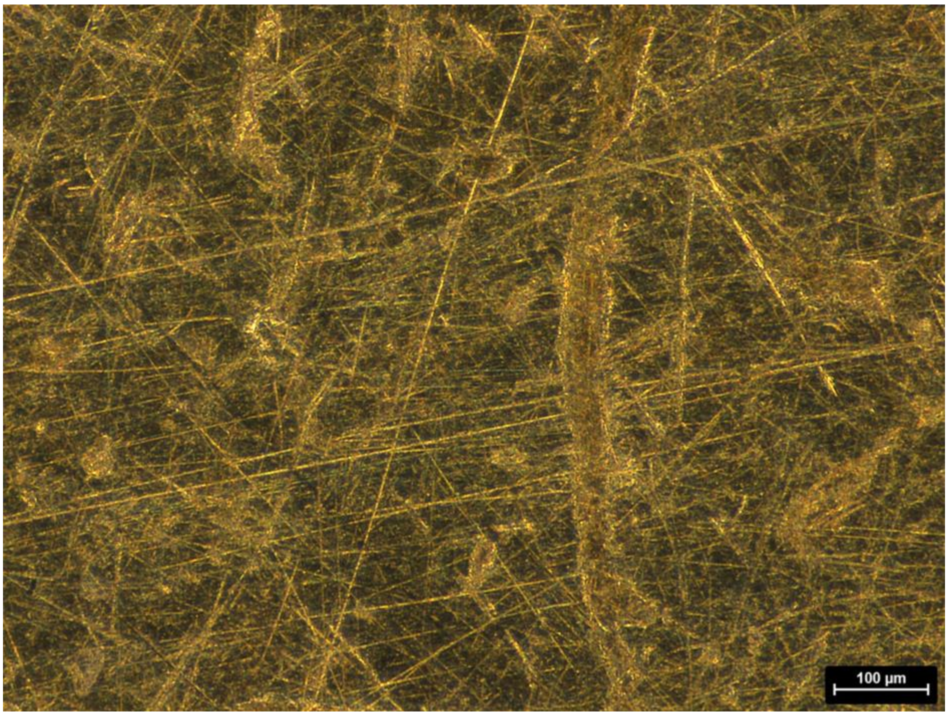

Figure S.1. 18 Reverse, flat field, in behind goddess's shoulder

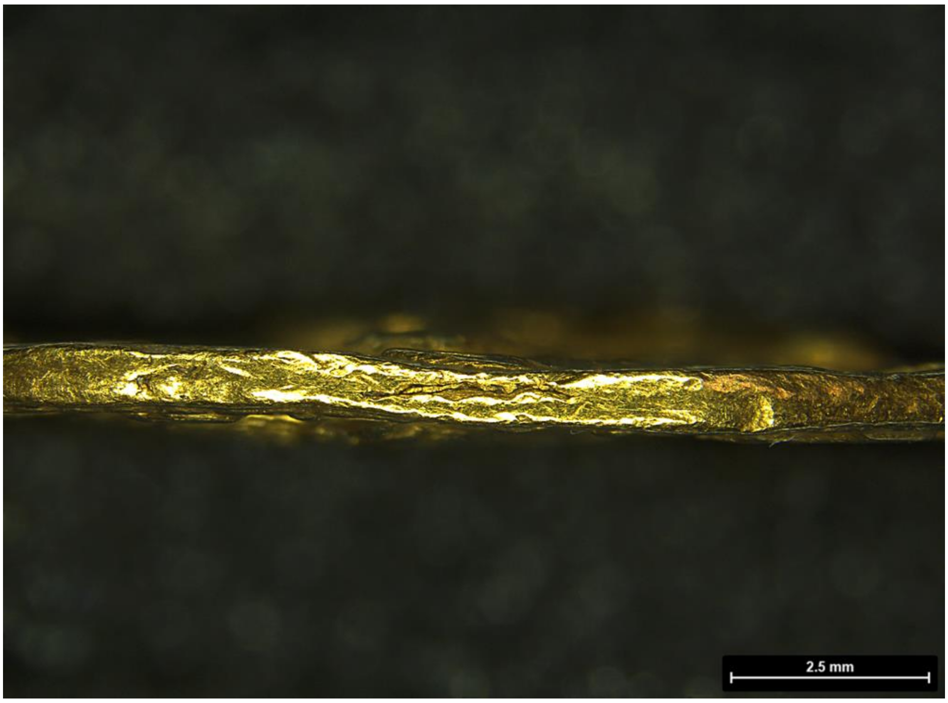

Figure S.1. 19 Edge, first image

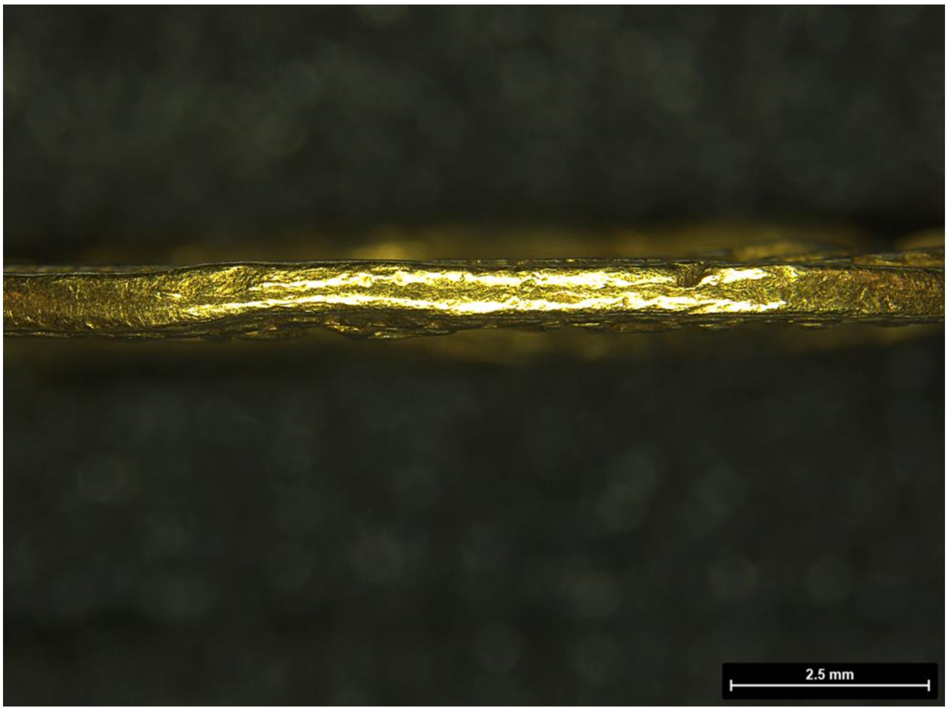

Figure S.1. 20 Edge, second image

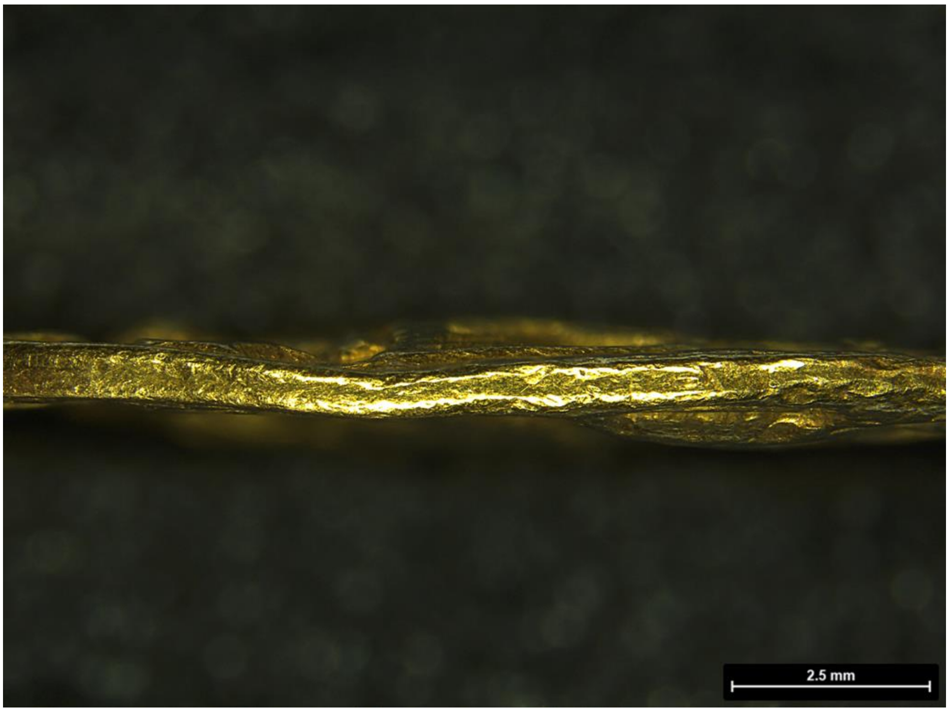

Figure S.1. 21 Edge, third image

S.1.2.3 Additional images

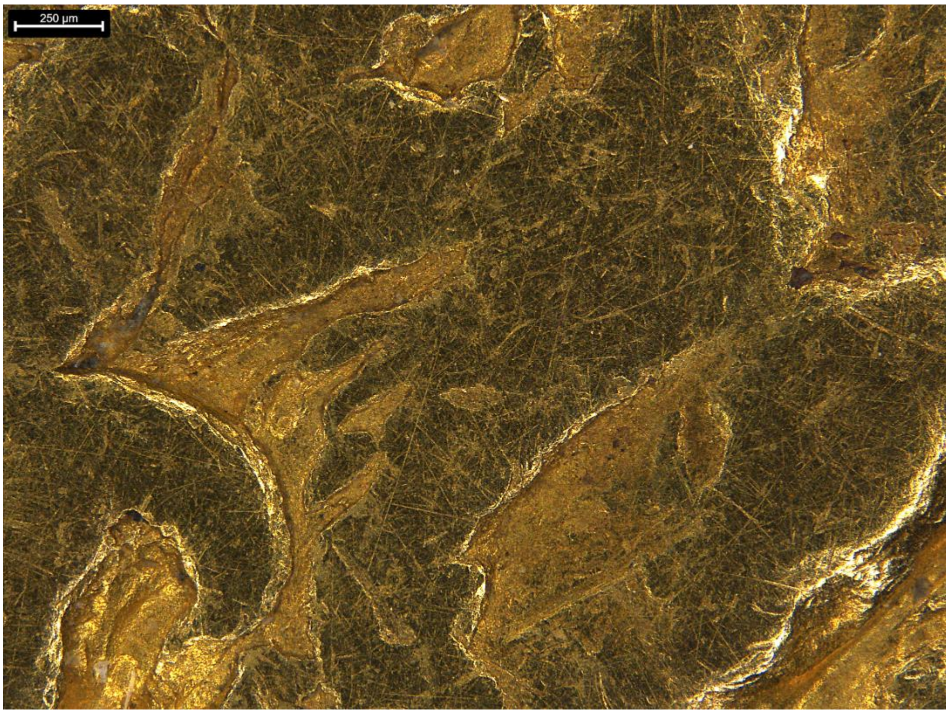

Figure S.1. 22 Detail of reverse showing scratches and fractures

S.1.3 Coin GLAHM:29596 (Questionable Gordian III medallion / binio)

S.1.3.1 Whole coin photographs

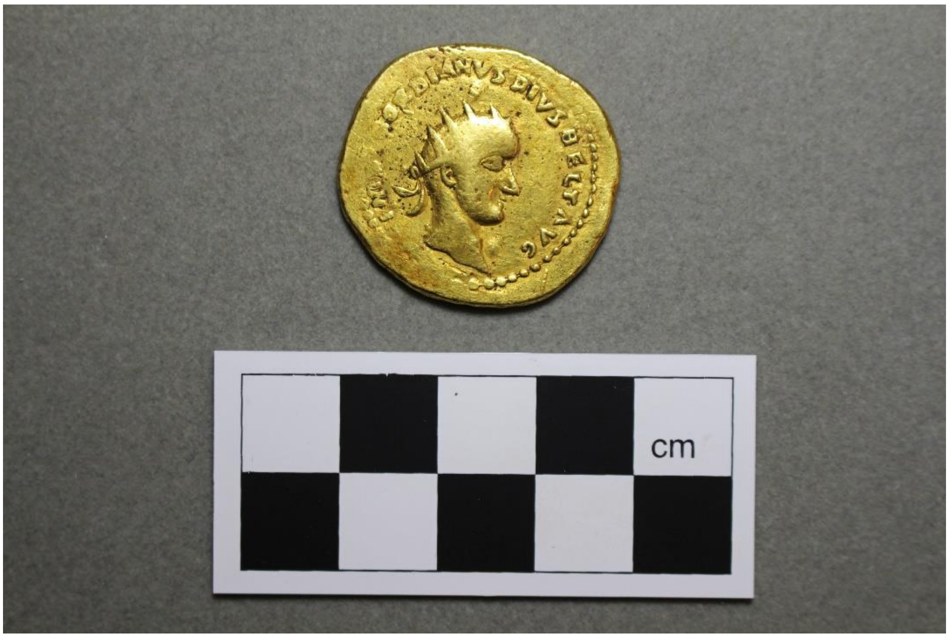

Figure S.1. 23 Obverse of Coin GLAHM:29596

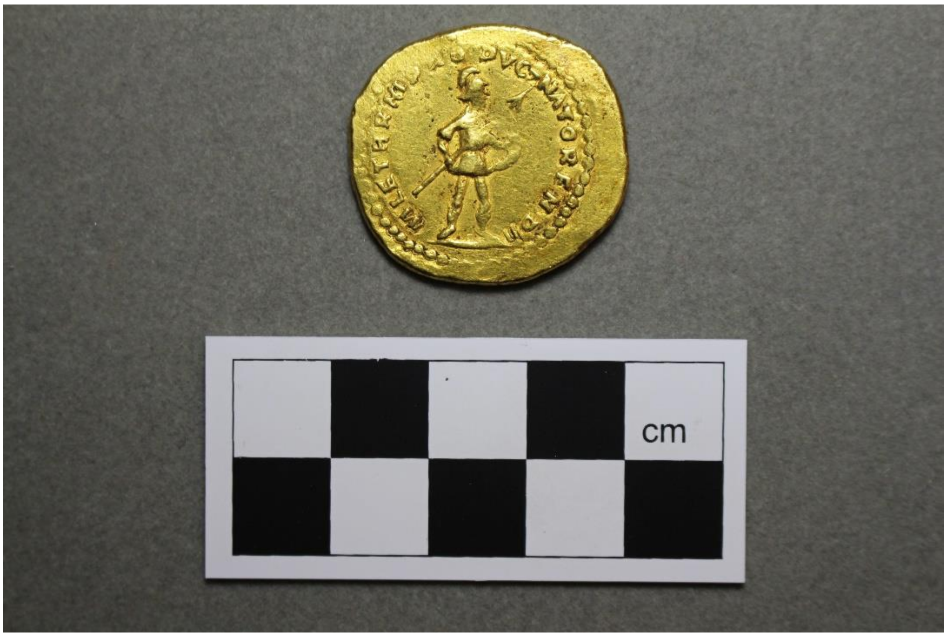

Figure S.1. 24 Reverse of Coin GLAHM:29596

S.1.3.2 Standardised images

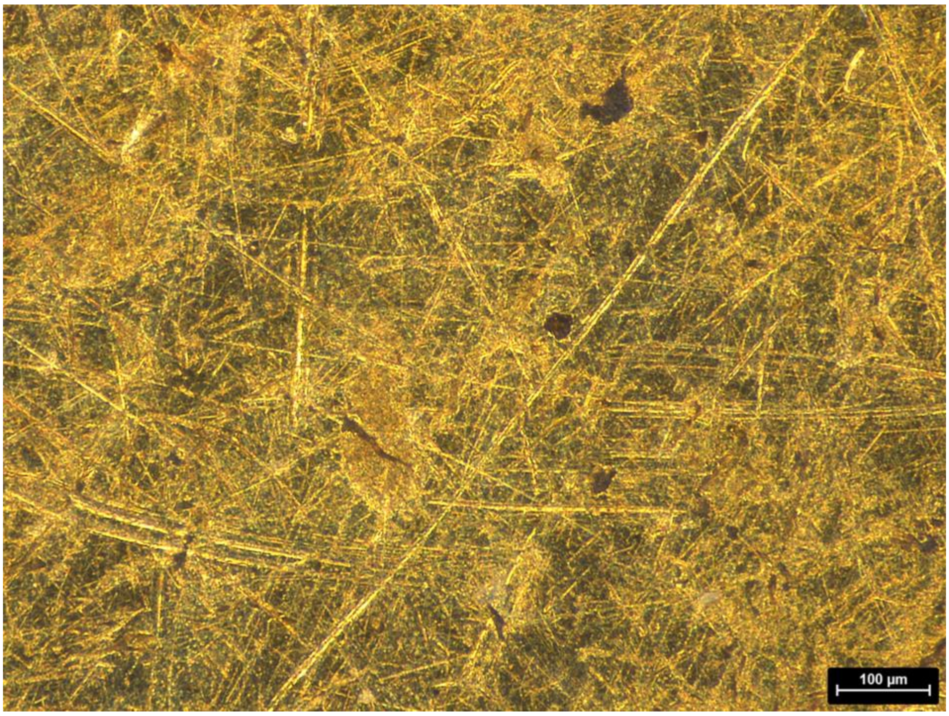

Figure S.1. 25 Obverse, exposed area, to eft of emperor's eye

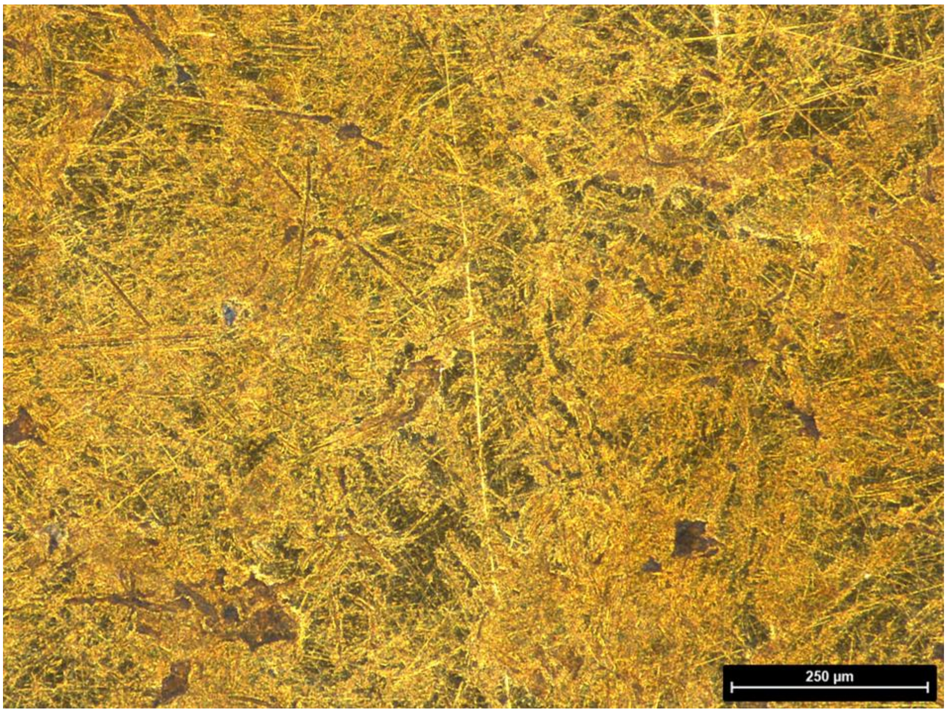

Figure S.1. 26 Obverse, flat field, to right of emperor's nose

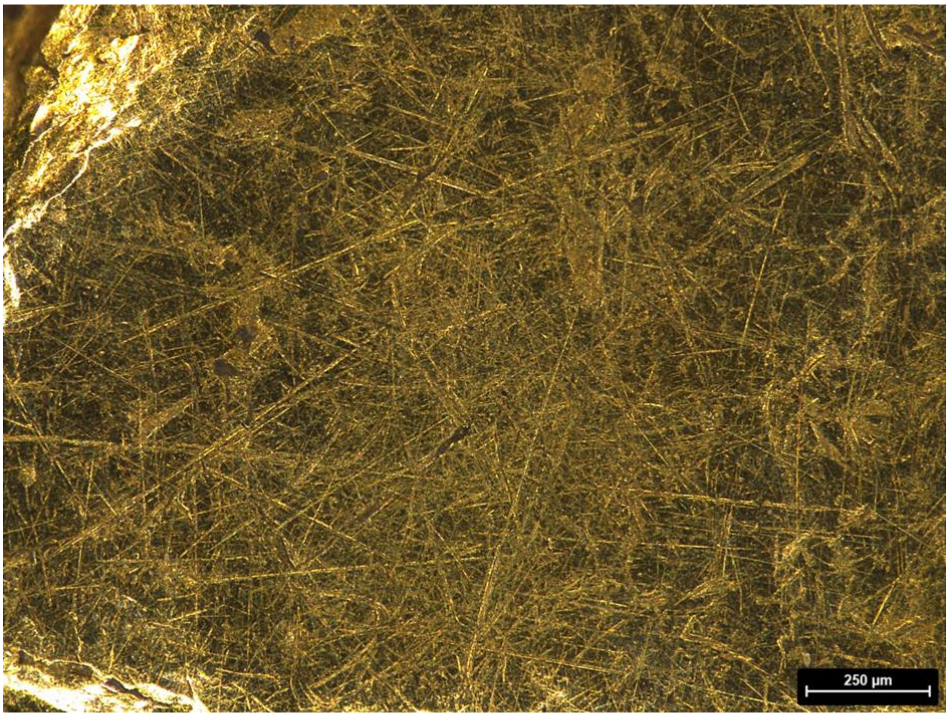

Figure S.1. 27 Reverse, exposed area, on tunic of Mars

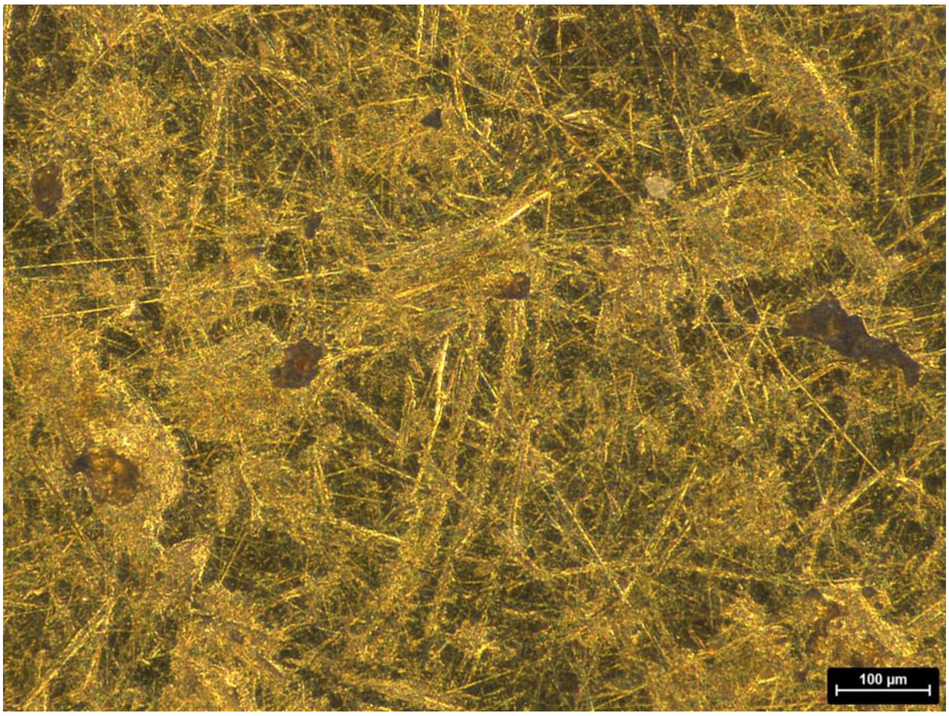

Figure S.1. 28 Reverse, flat field, in front of Mars's forehead

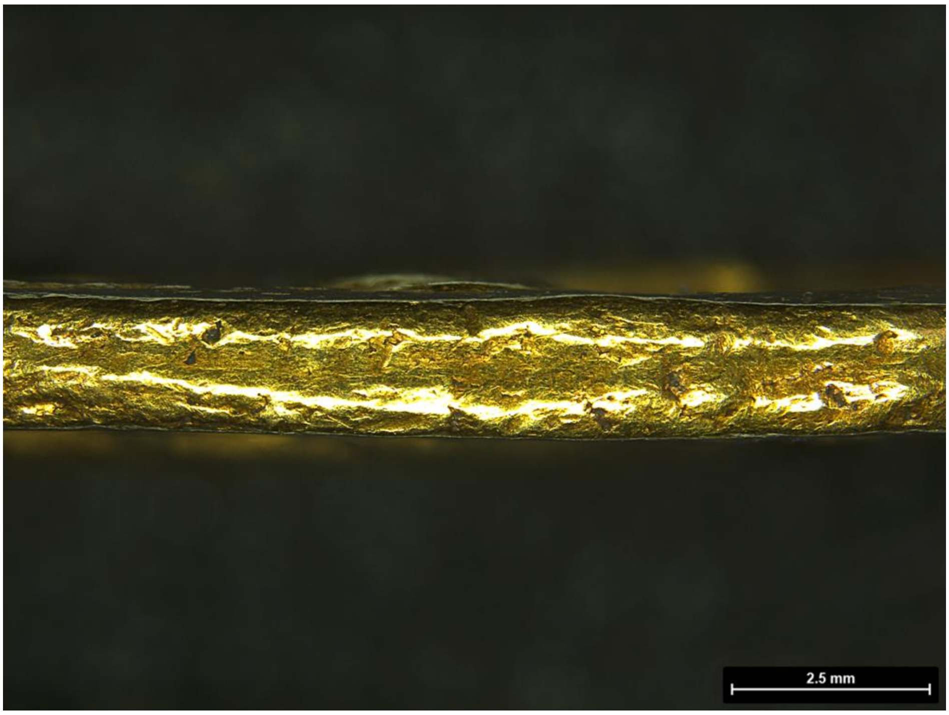

Figure S.1. 29 Edge, first image

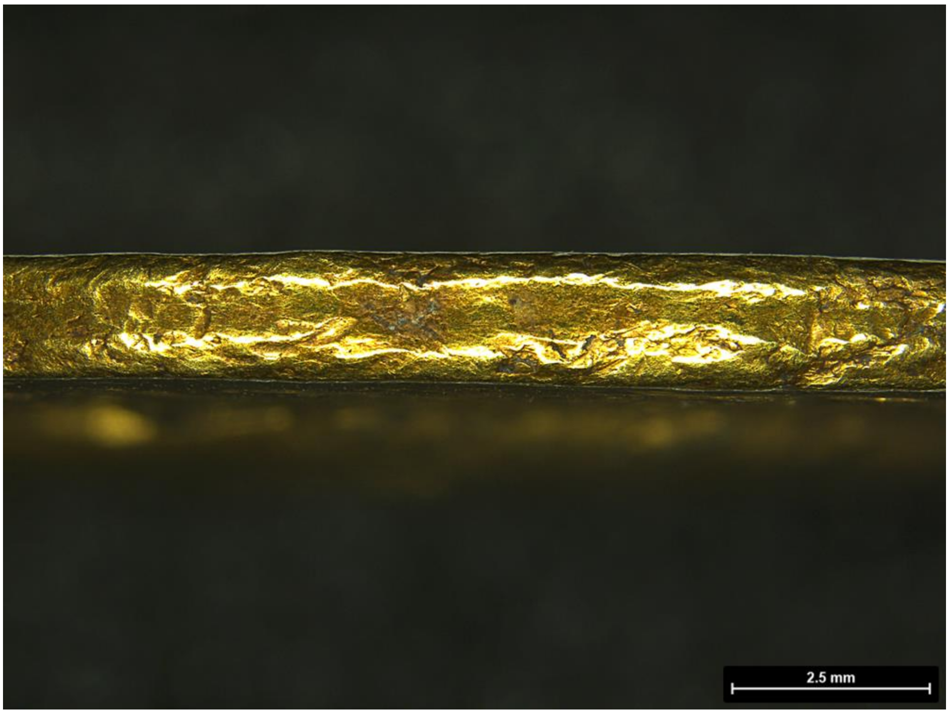

Figure S.1. 30 Edge, second image

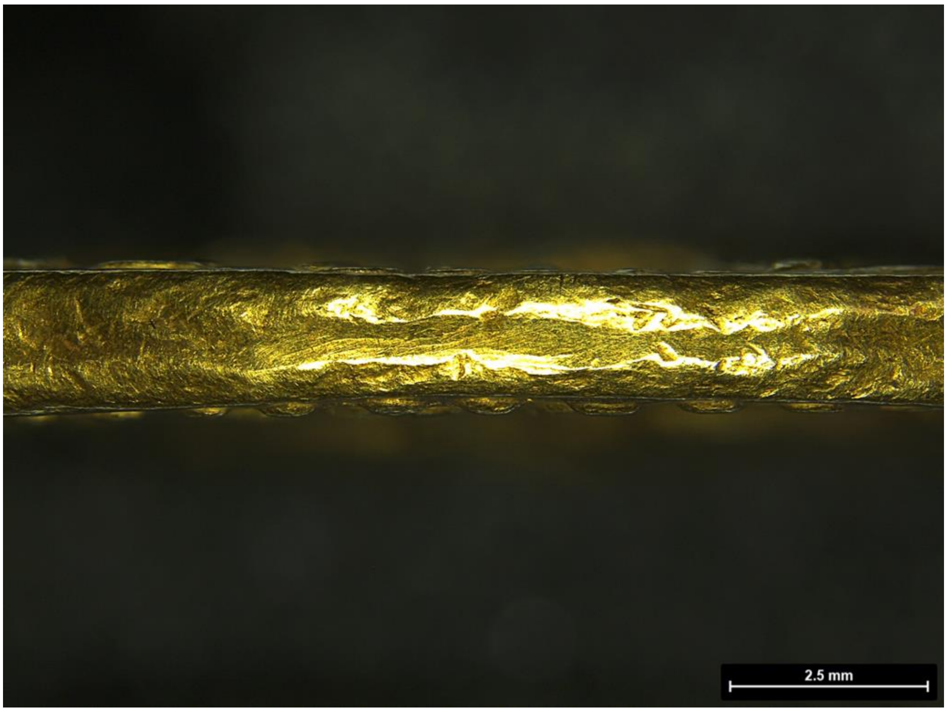

Figure S.1. 31 Edge, third image

1.3.3 Additional images

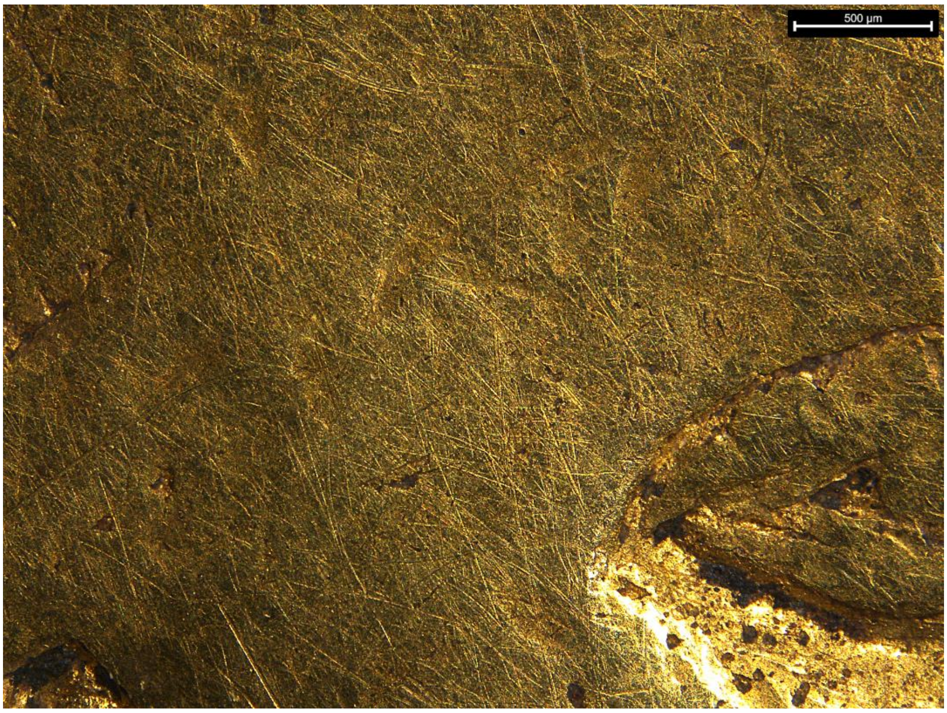

Figure S.1. 32 Detail of scratches and earthen deposits in angled light

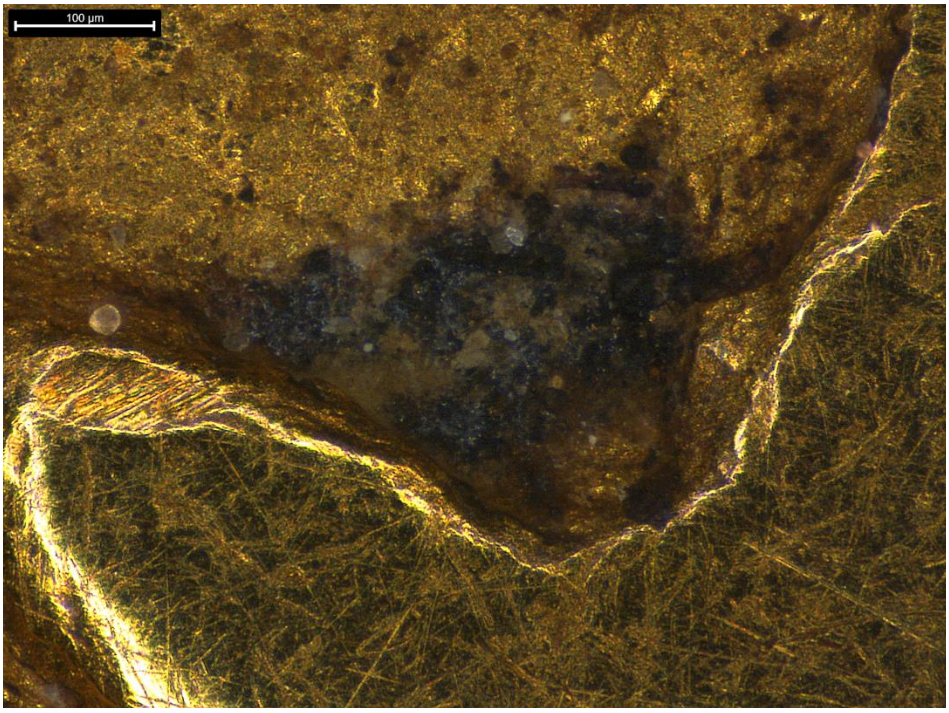

Figure S.1. 33 Detail of earthen deposit beside letter 'S' on obverse

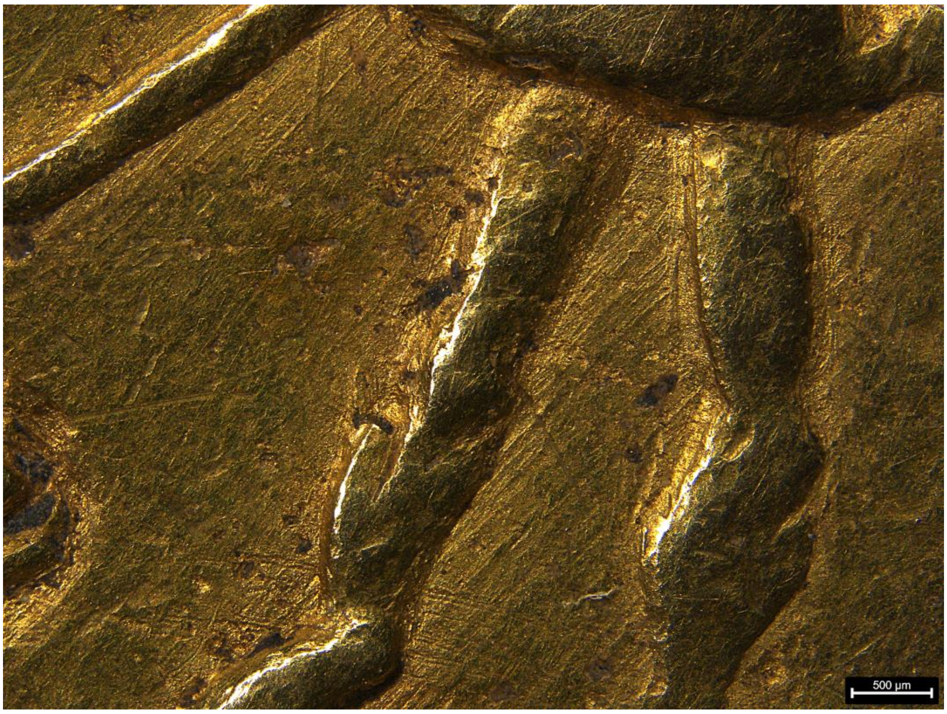

Figure S.1. 34 Detail of grooves in protected area of reverse field

S.1.4 Coin GLAHM:29820 (Questionable Philip I medallion)

S.1.4.1 Whole coin photographs

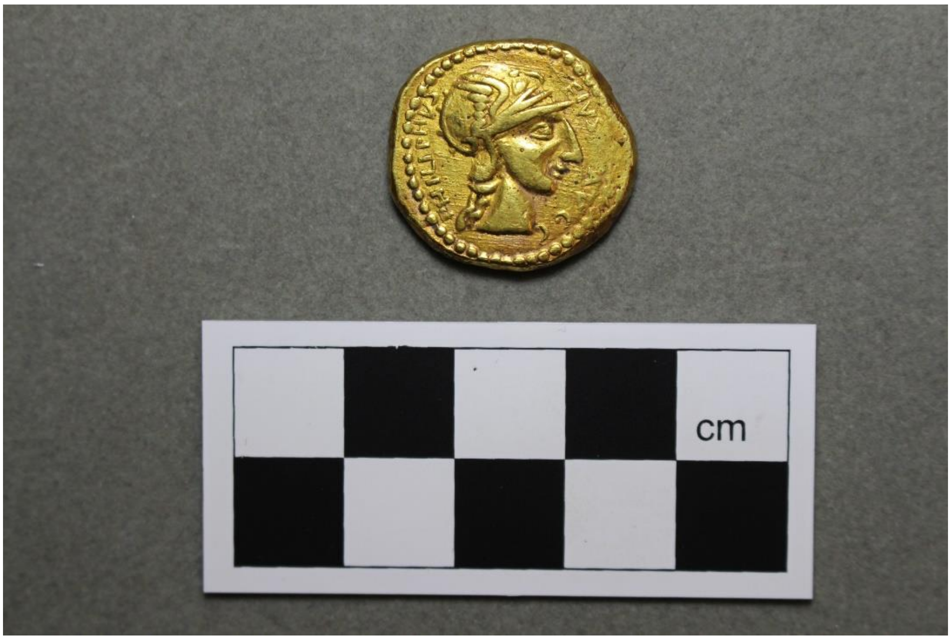

Figure S.1. 35 Obverse of Coin GLAHM:29820

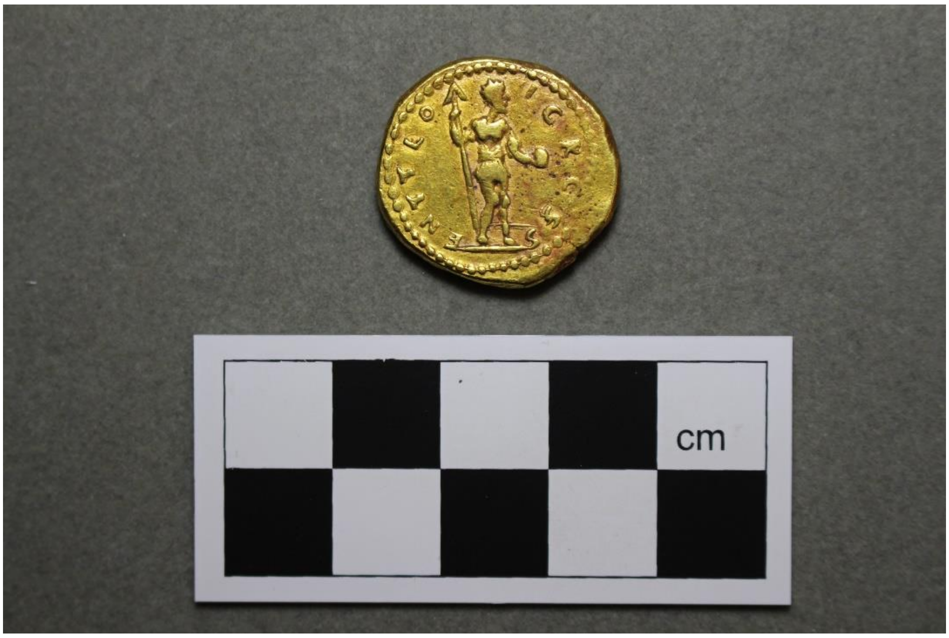

Figure S.1. 36 Reverse of Coin GLAHM:29820

S.1.4.2 Standardised images

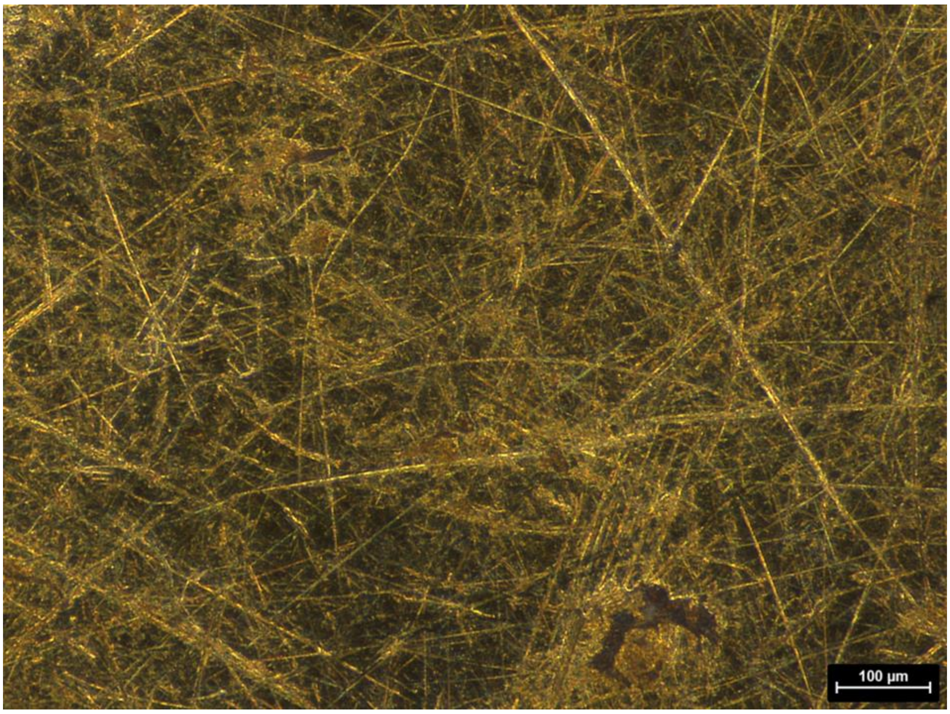

Figure S.1. 37 Obverse, exposed area, cheekbone of Roma

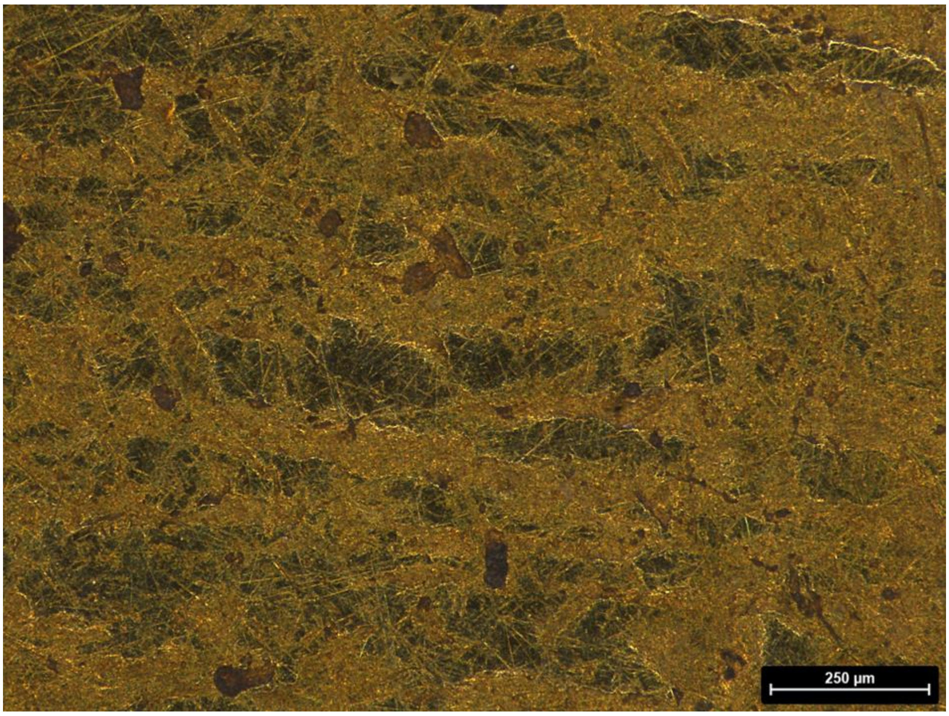

Figure S.1. 38 Obverse, flat field, to the right of the letter 'H'

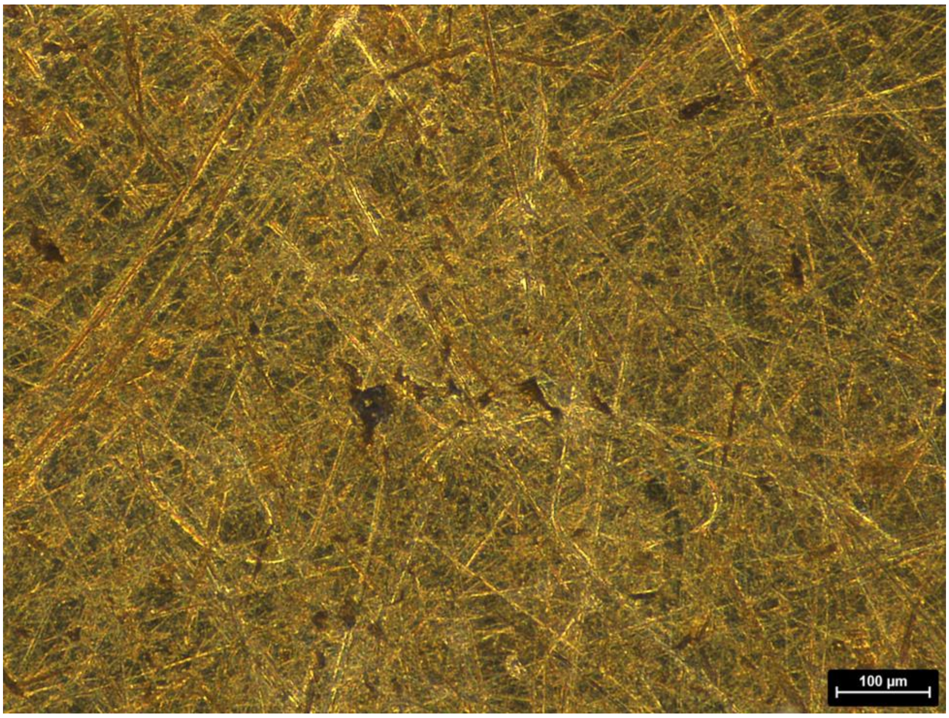

Figure S.1. 39 Reverse, exposed area, emperor's tunic

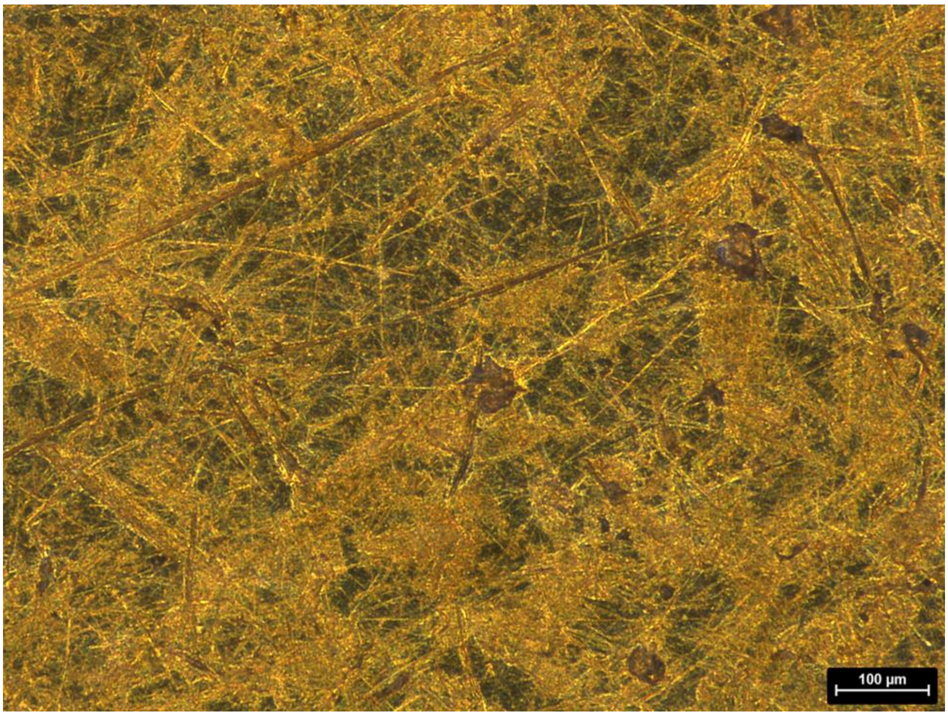

Figure S.1. 40 Reverse, flat field, to left of spear and emperor's waist

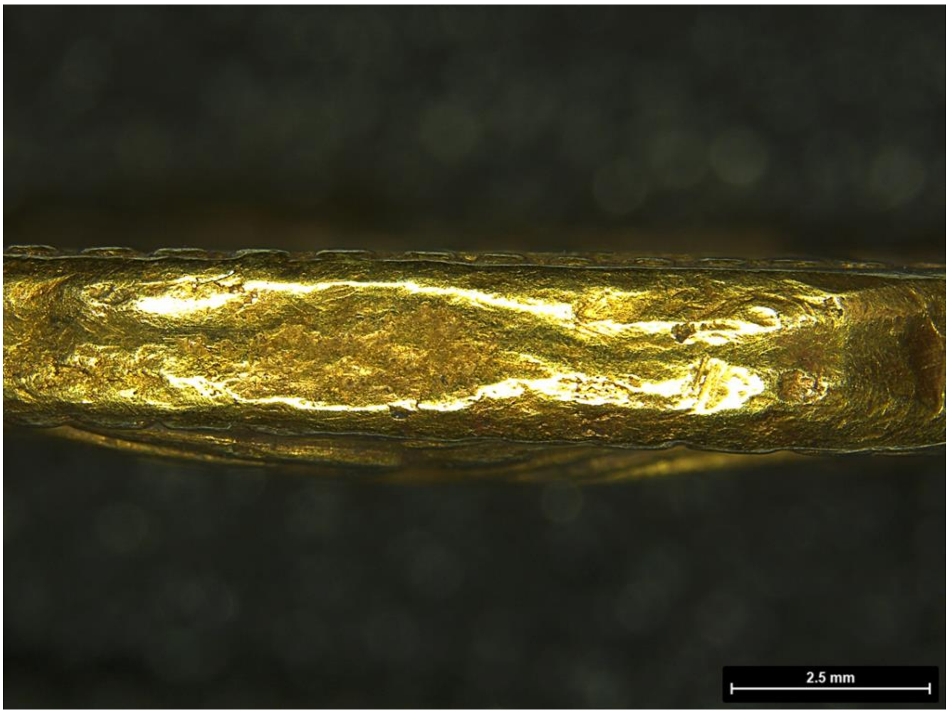

Figure S.1. 41 Edge, first image

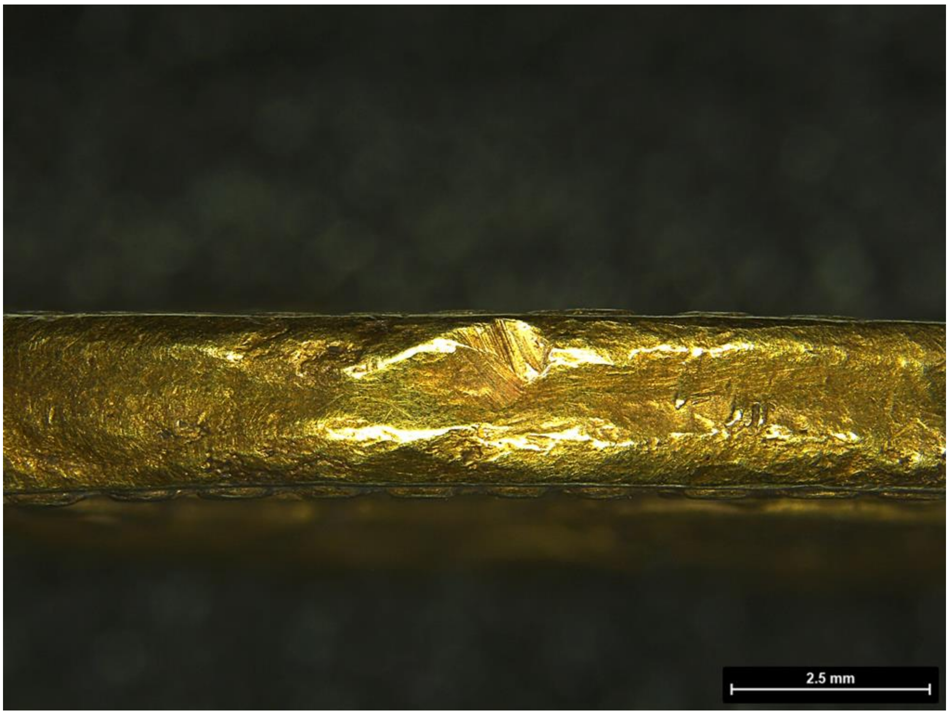

Figure S.1. 42 Edge, second image

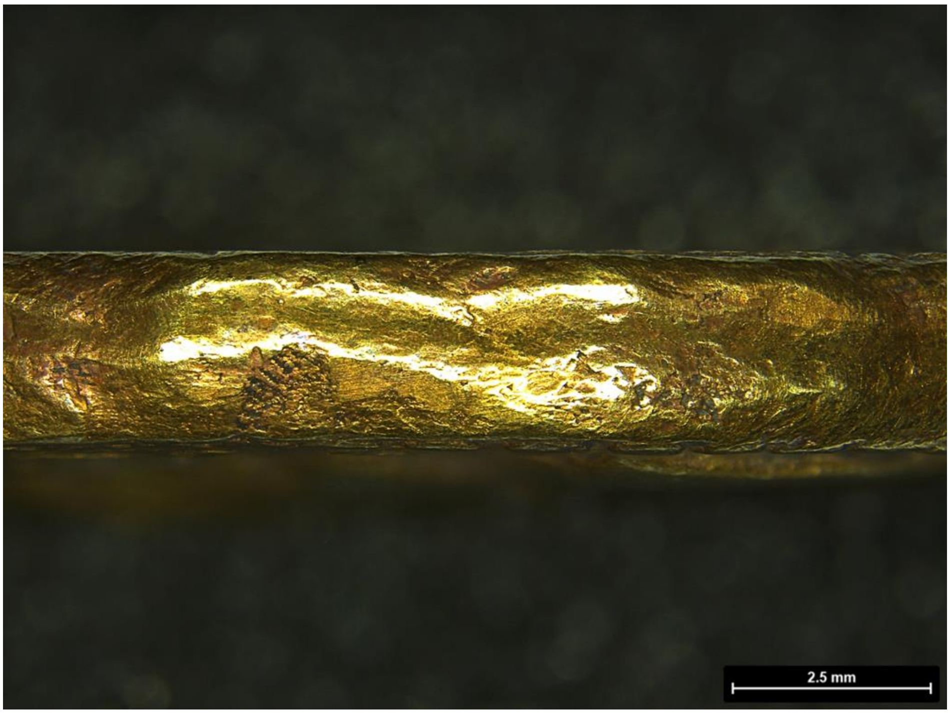

Figure S.1. 43 Edge, third image

S.1.4.3 Additional images

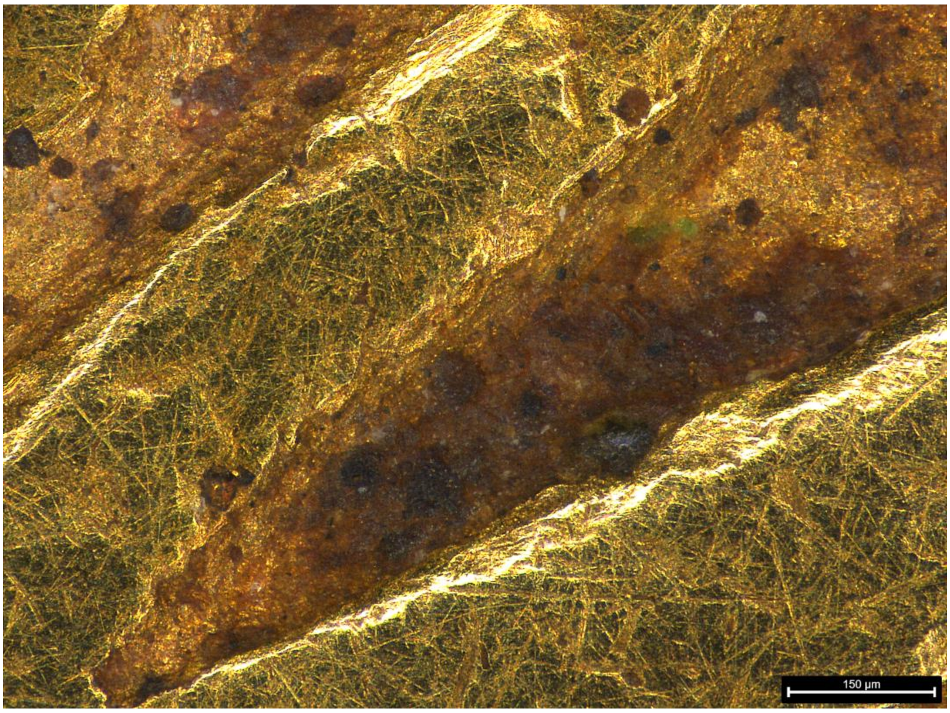

Figure S.1. 44 Detail of obverse showing earthen deposits in helmet

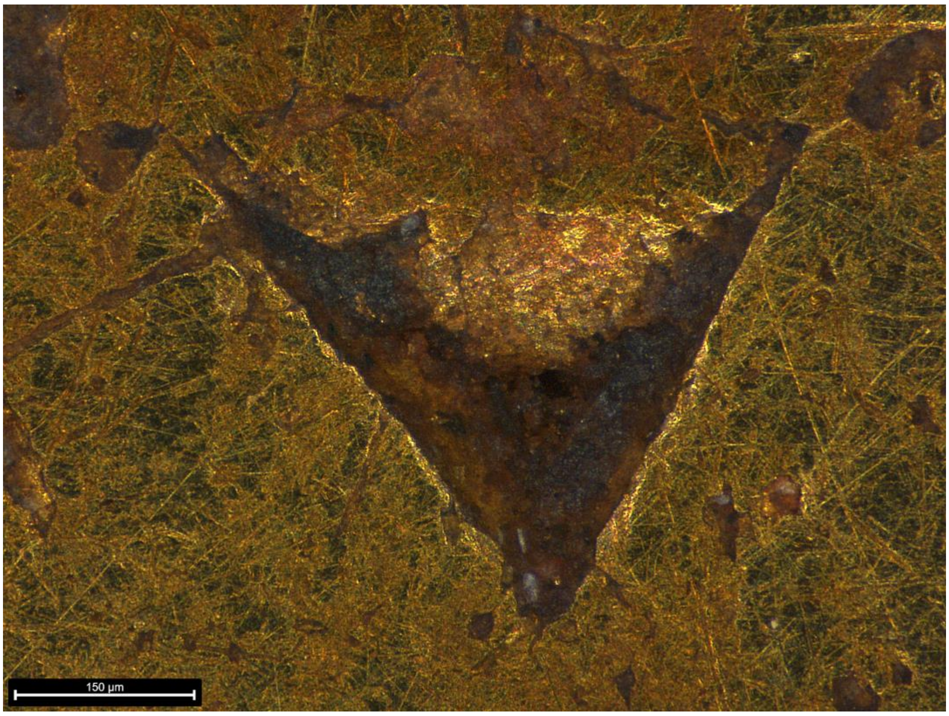

Figure S.1. 45 Detail of obverse showing earthen deposit in 'V'-shape gouge

S.1.5 Coin GLAHM:29821 (Questionable Philip I medallion)

S.1.5.1 Whole coin photographs

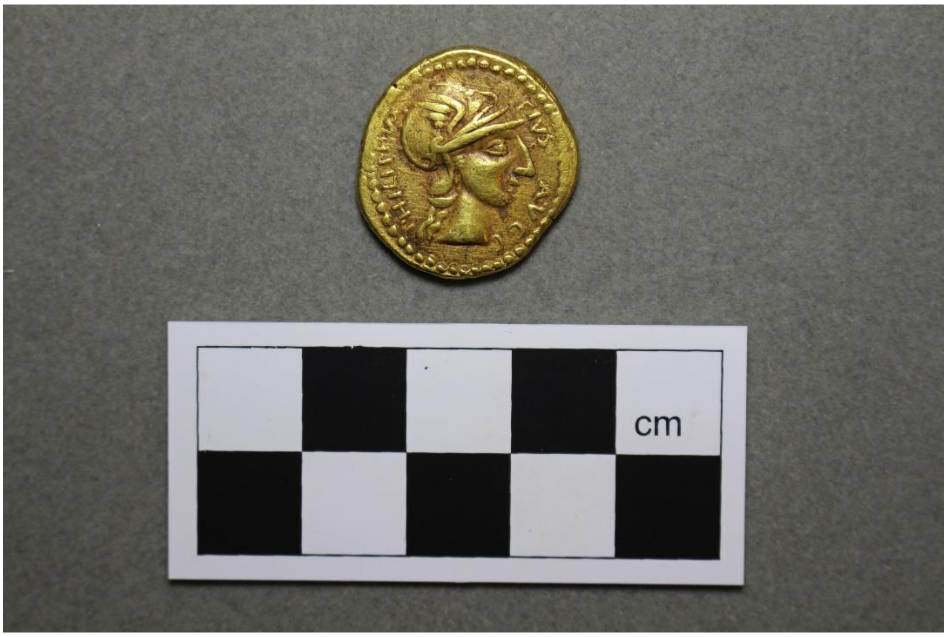

Figure S.1. 46 Obverse of Coin GLAHM:29821

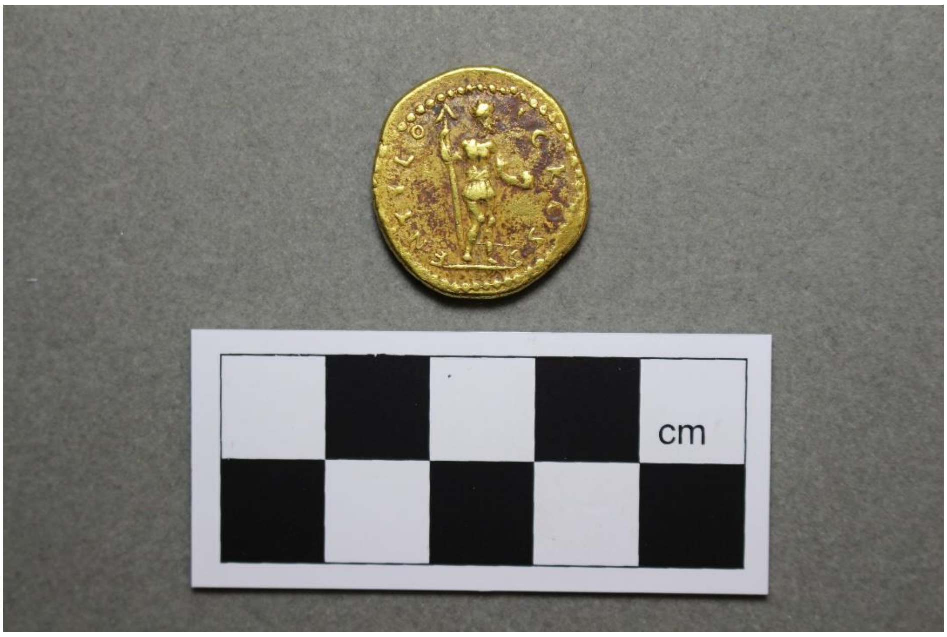

Figure S.1. 47 Reverse of Coin GLAHM:29821

S.1.5.2 Standardised images

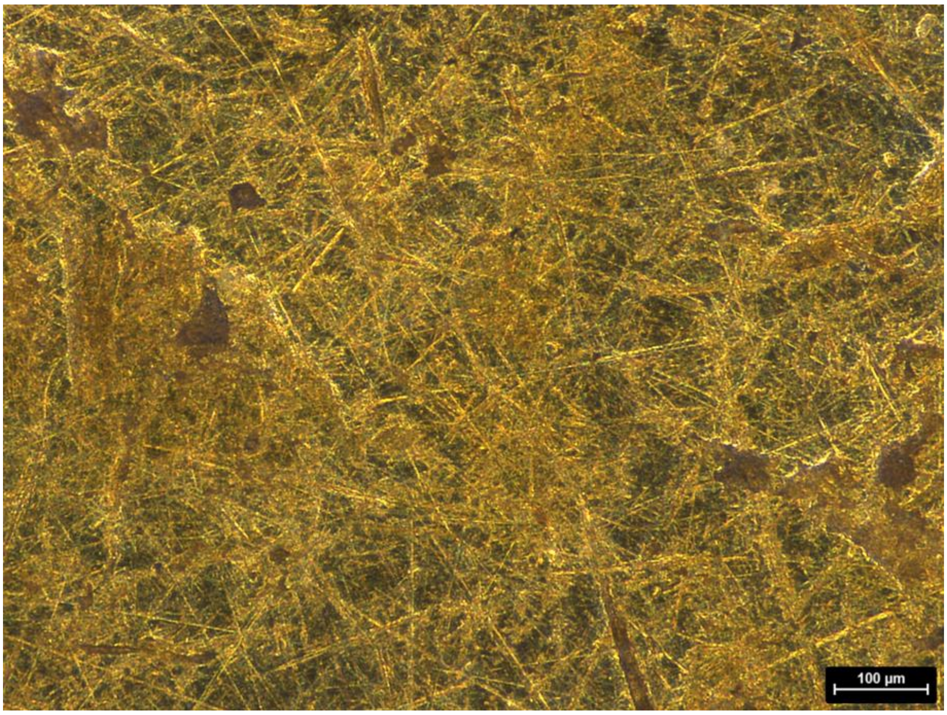

Figure S.1. 48 Obverse, exposed area, emperor's cheek

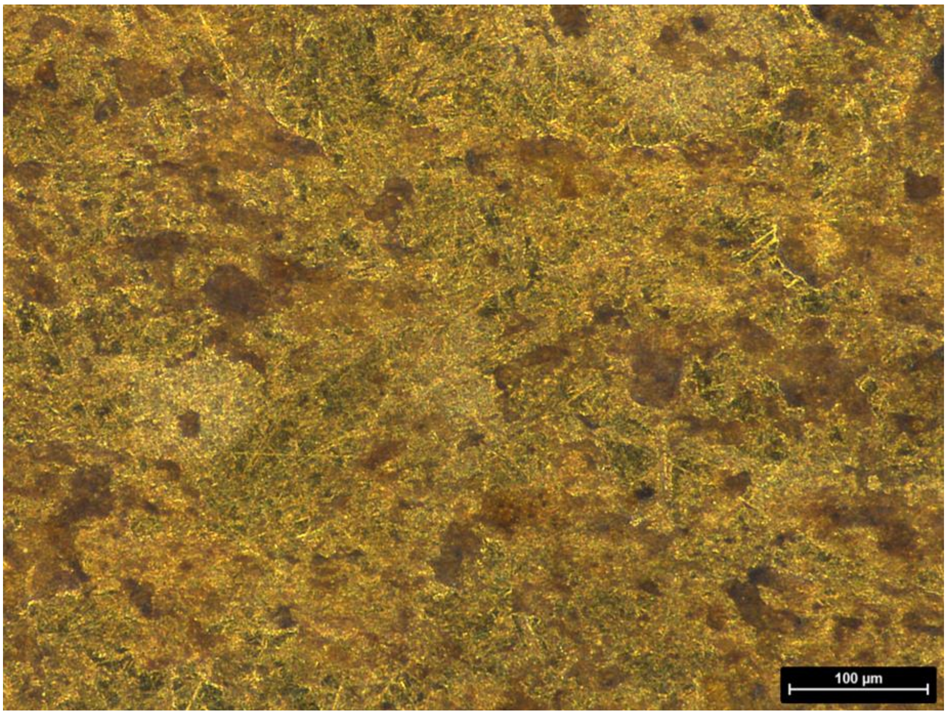

Figure S.1. 49 Obverse, flat field, to left of emperor's head

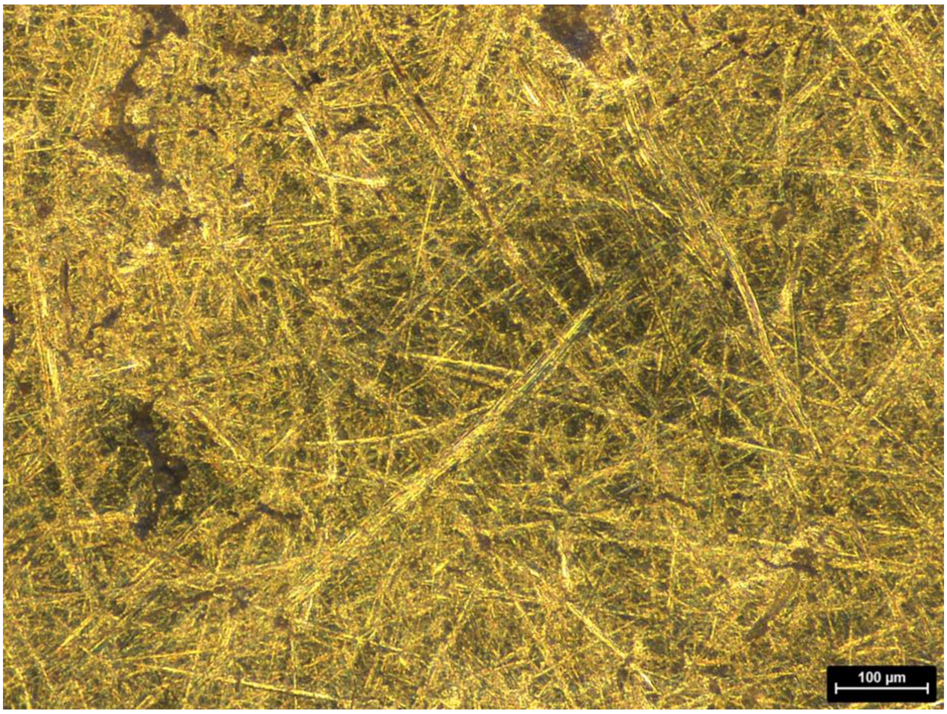

Figure S.1. 50 Reverse, exposed area, emperor's tunic

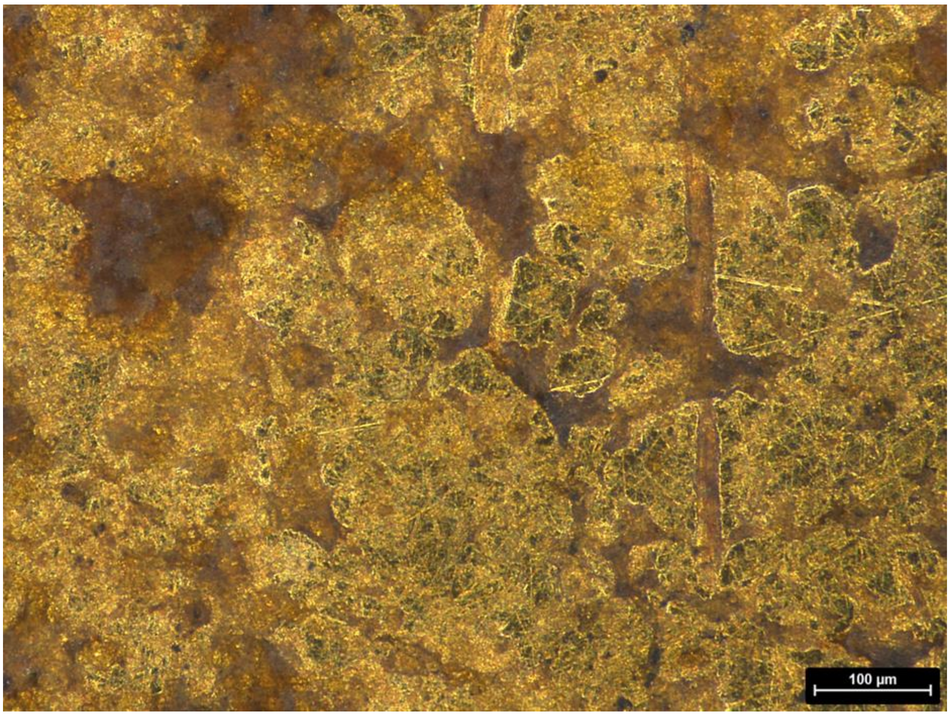

Figure S.1. 51 Reverse, flat field, to right of emperor's tunic

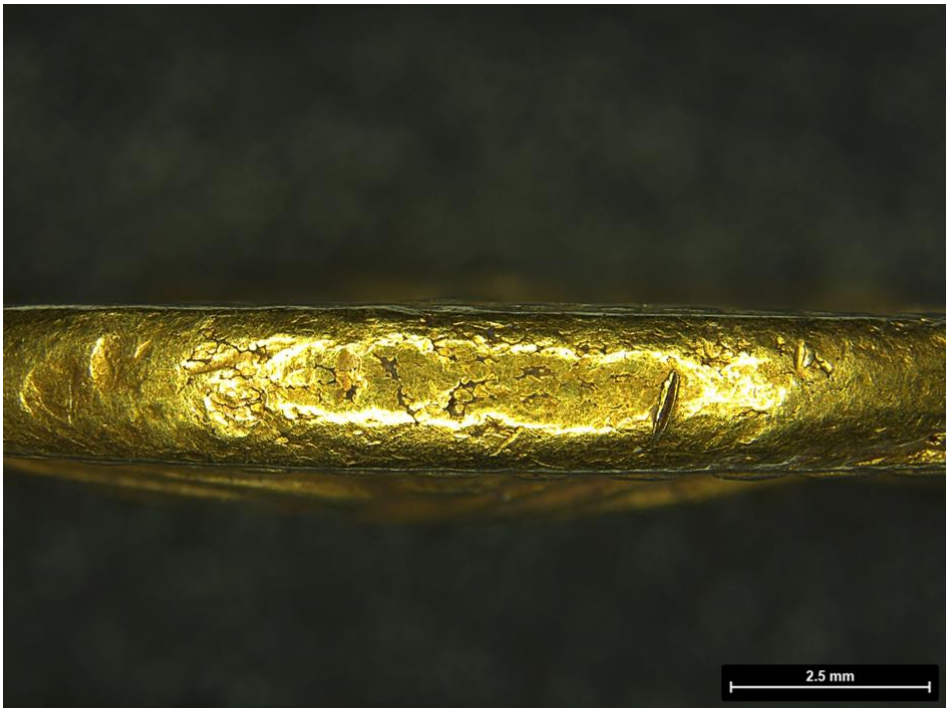

Figure S.1. 52 Edge, first image

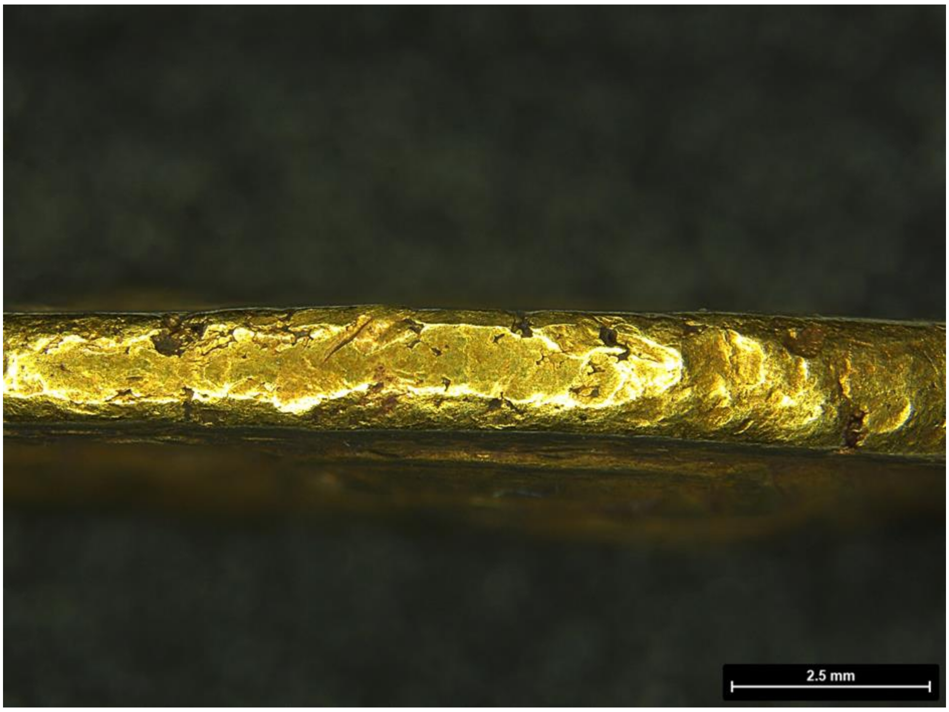

Figure S.1. 53 Edge, second image

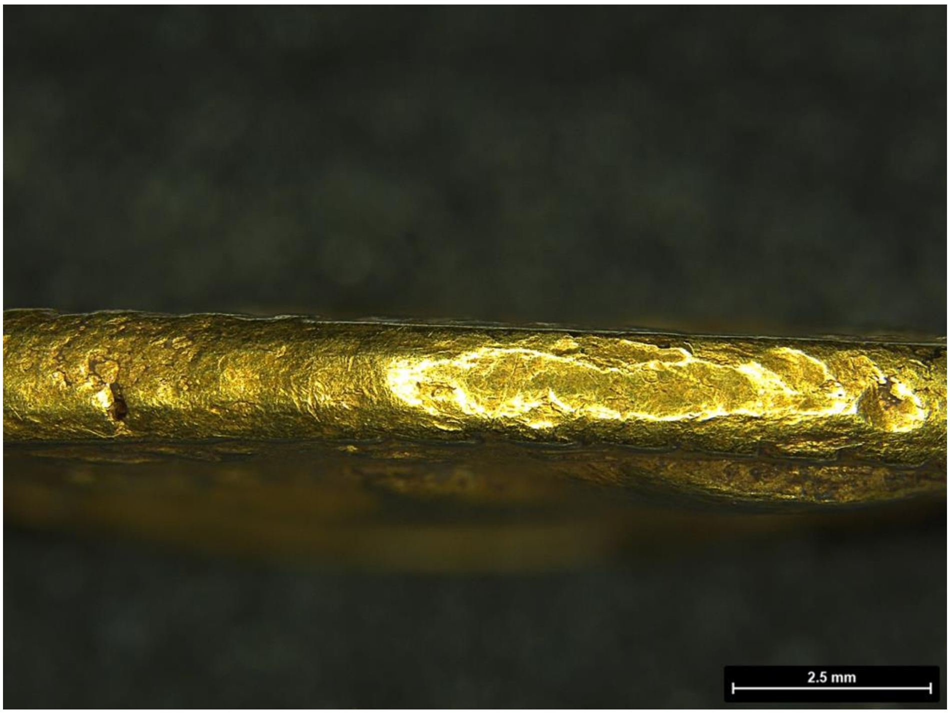

Figure S.1. 54 Edge, third image

1.5.3 Additional images

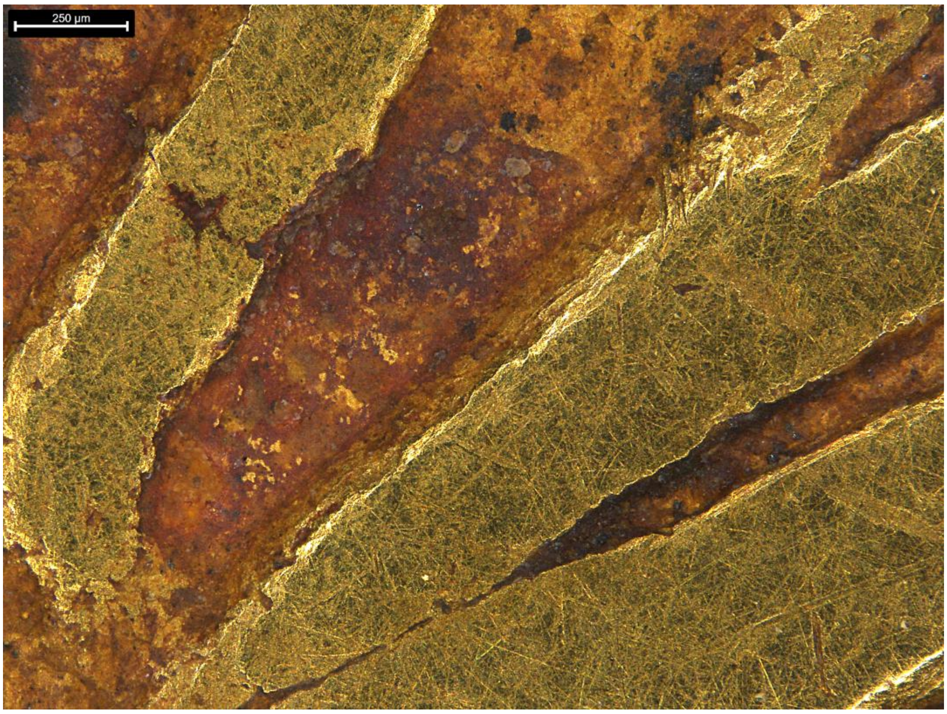

Figure S.1. 55 Detail of obverse showing earthen deposits in helmet

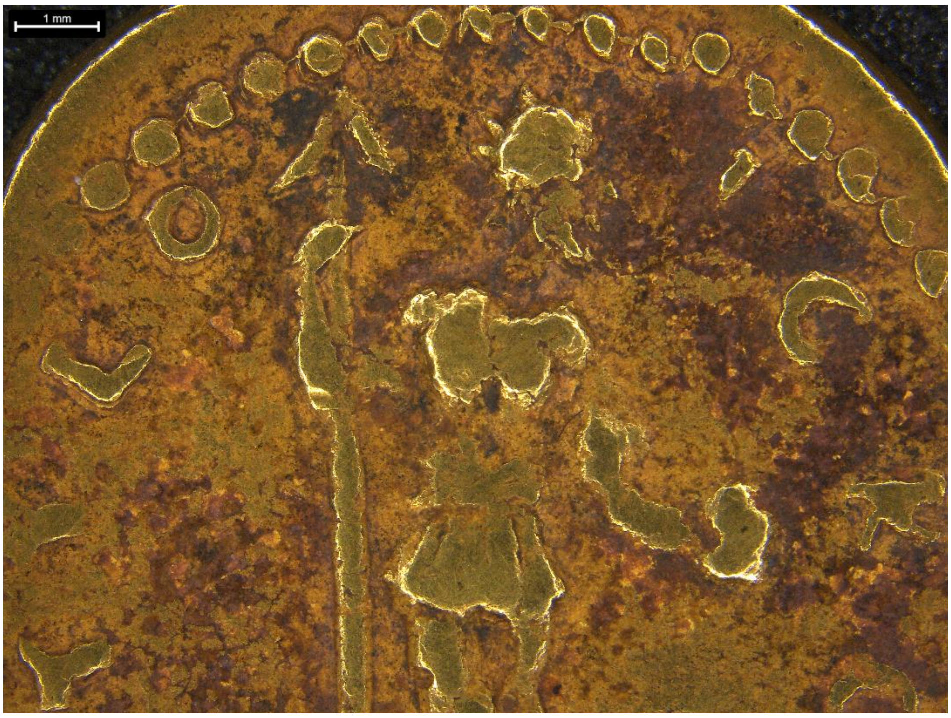

Figure S.1. 56 Detail of reverse side showing reddish areas

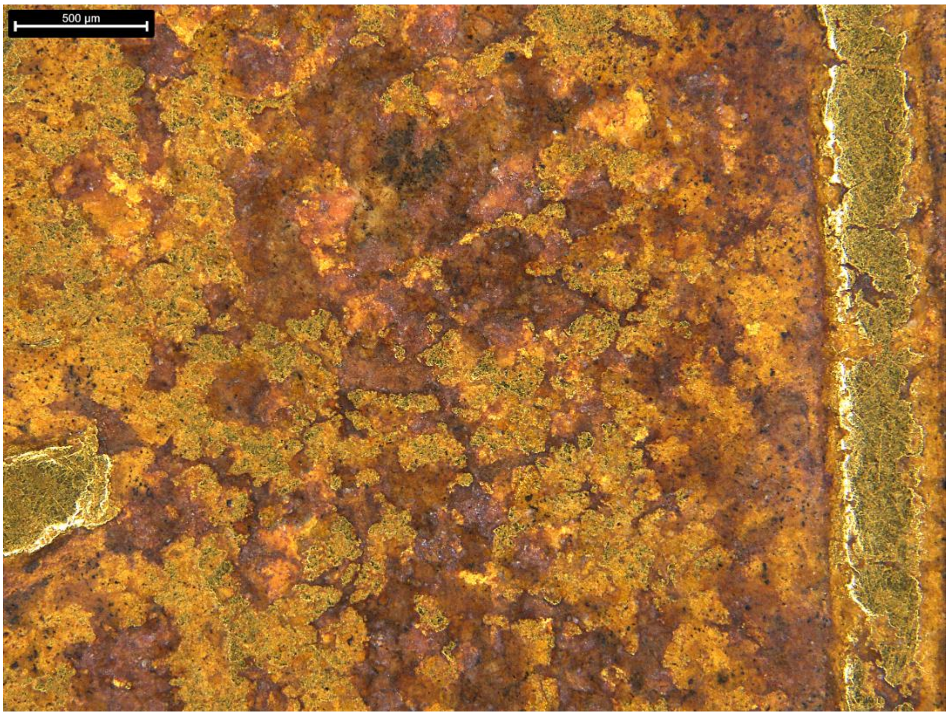

Figure S.1. 57. Detail of reverse side showing cracking of surface and reddish deposit

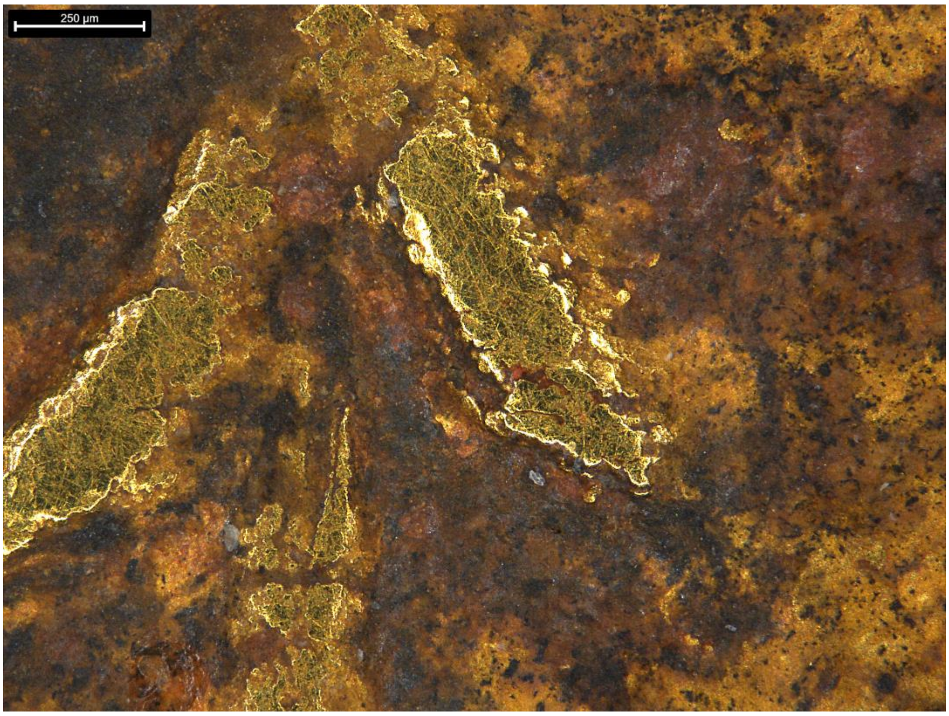

Figure S.1. 58 Detail of reverse side showing reddish areas and earthen deposits

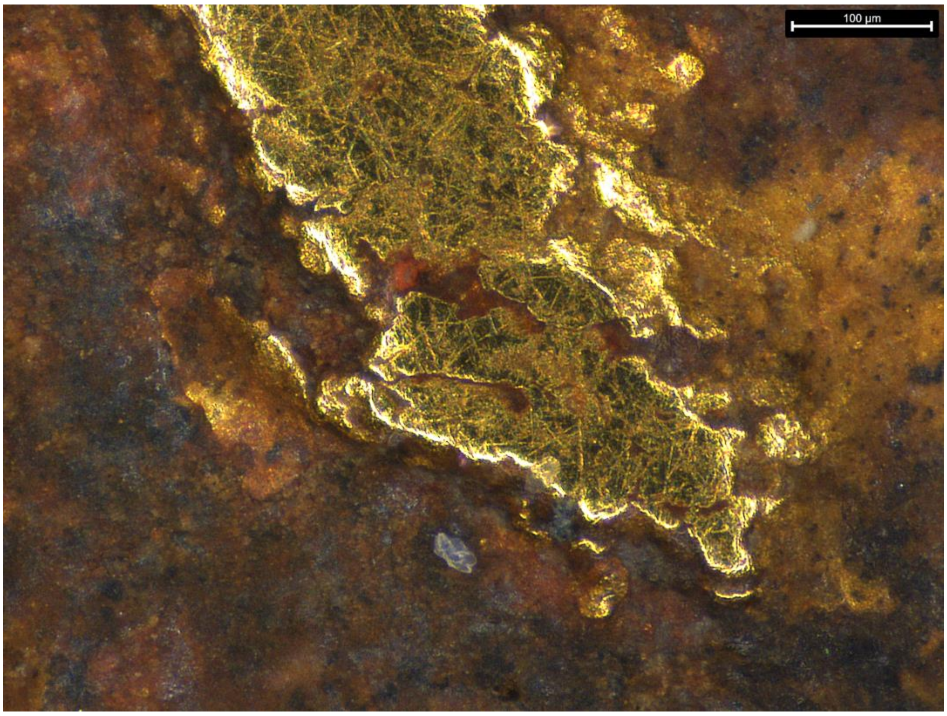

Figure S.1. 59 Detail of reverse side showing incomplete casting

S.1.6 Coin GLAHM:40333 (Questionable Sponsian medallion)

S.1.5.1 Whole coin photographs

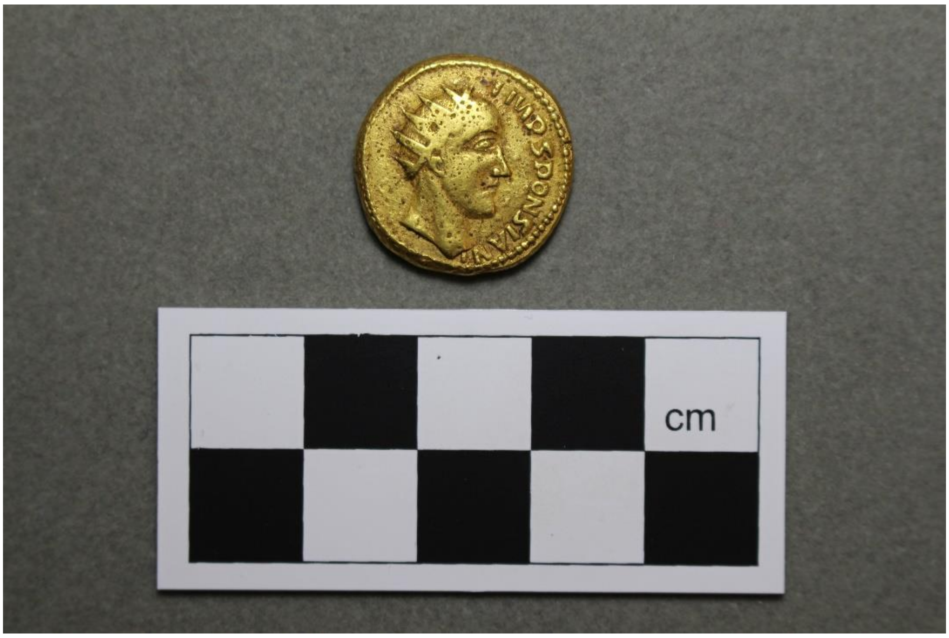

Figure S.1. 60 Obverse of Coin GLAHM:40333

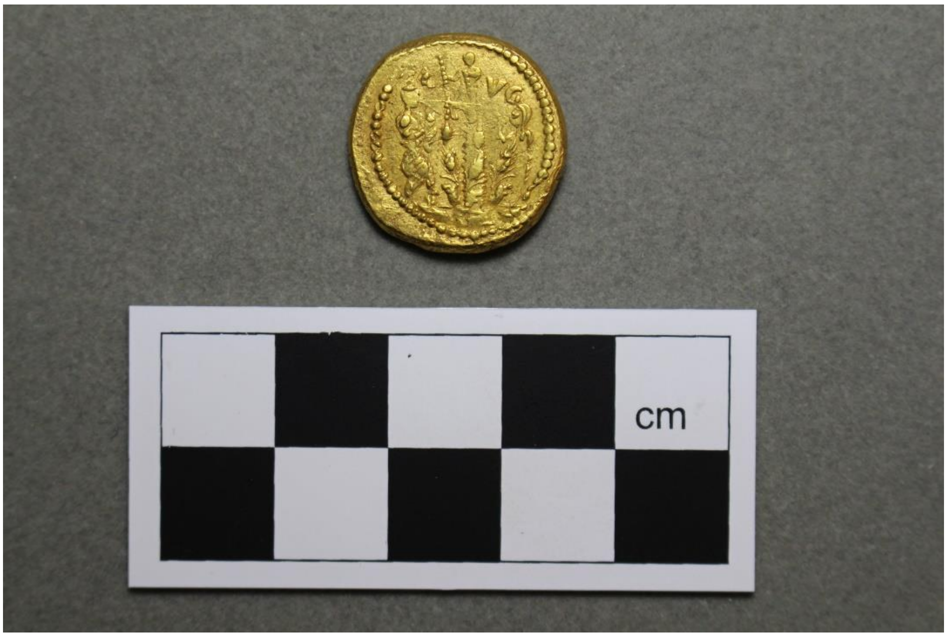

Figure S.1. 61 Reverse of Coin GLAHM:40333

1.6.2 Standardised images

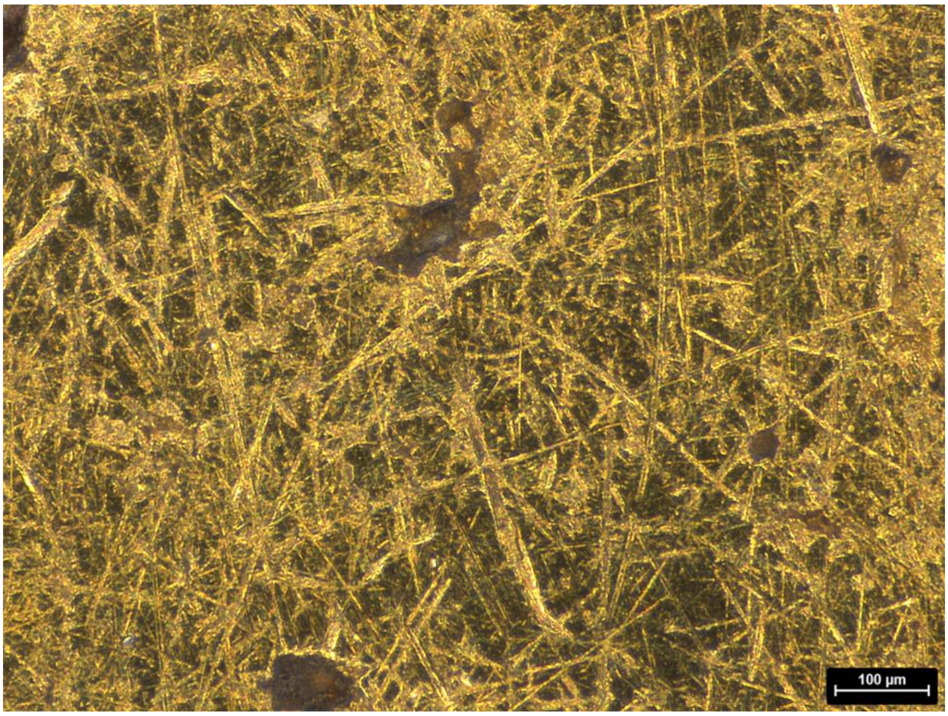

Figure S.1. 62 Obverse, exposed area, emperor's head to right of ear

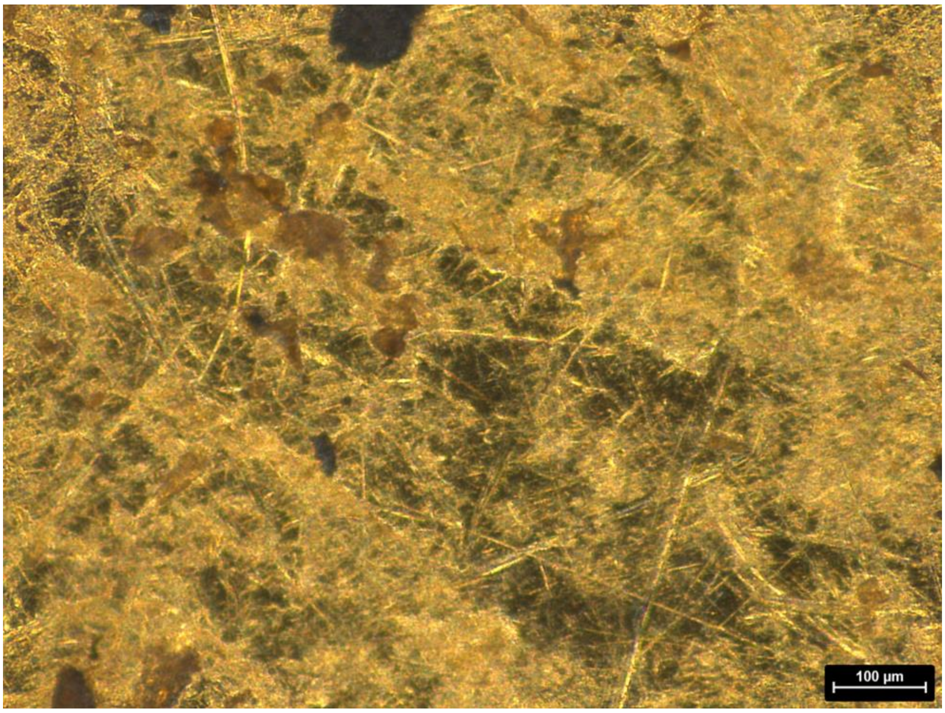

Figure S.1. 63 Obverse, flat field, near lowest ray of emperor's crown

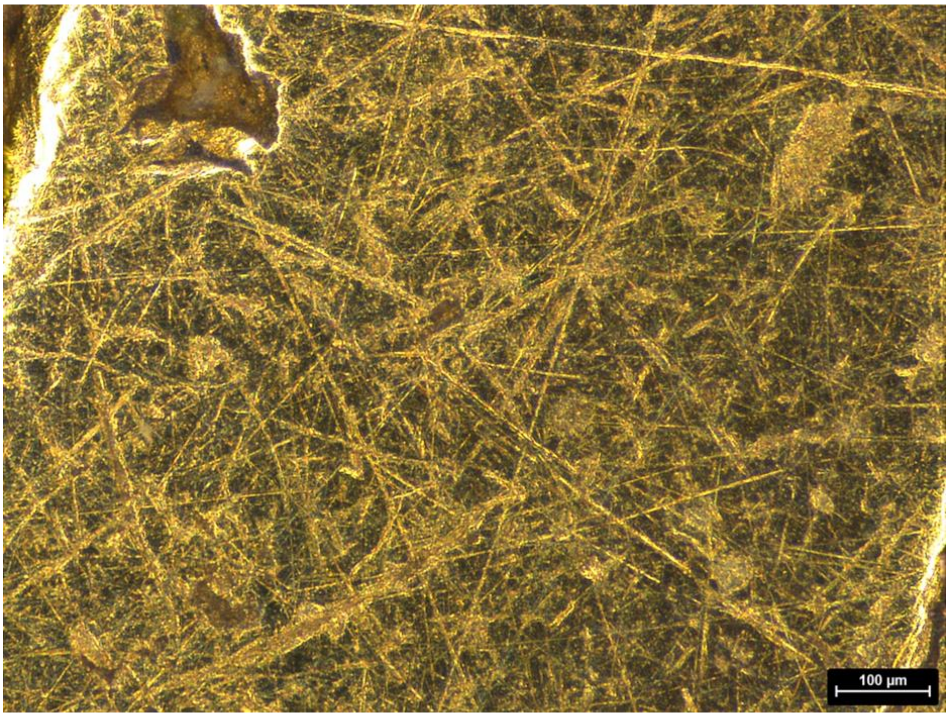

Figure S.1. 64 Reverse, exposed area, lower right of wheat ear

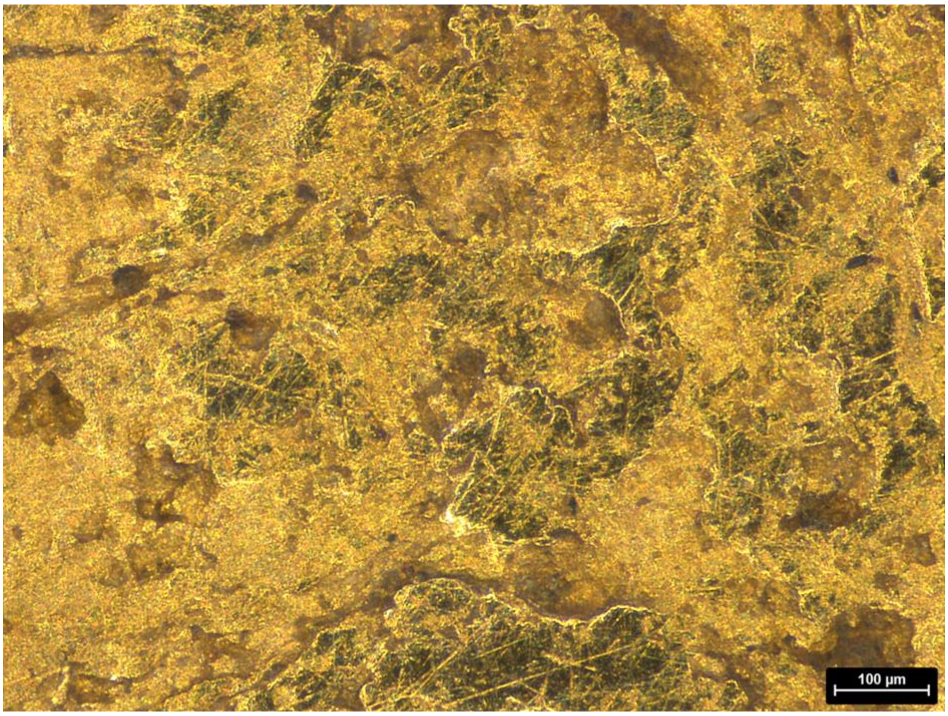

Figure S.1. 65 Reverse, flat field, to left of waist of standing figure

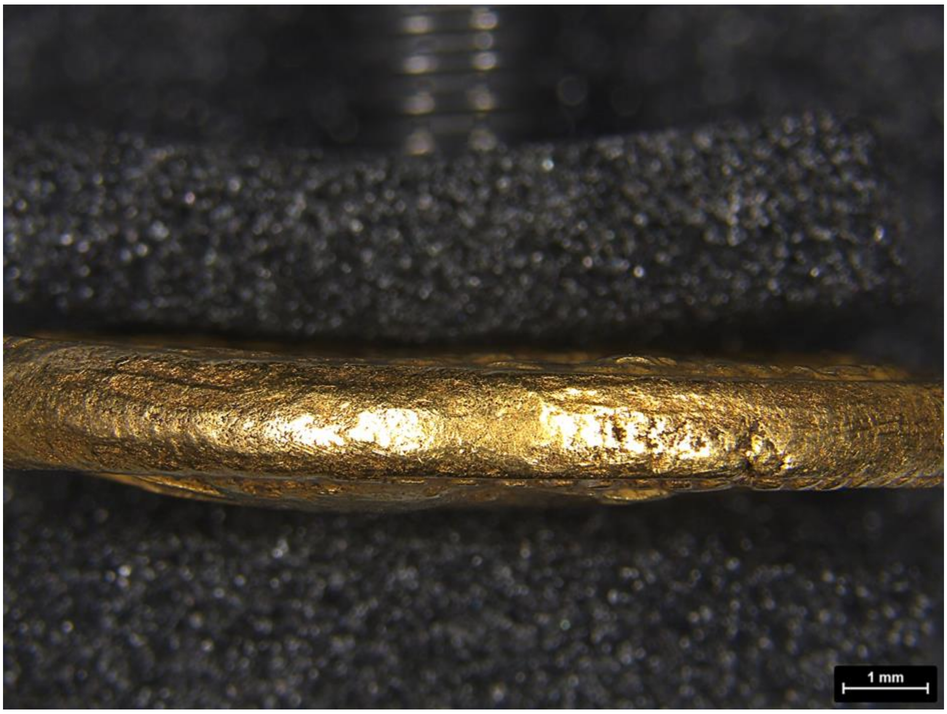

Figure S.1. 66 Edge, first image

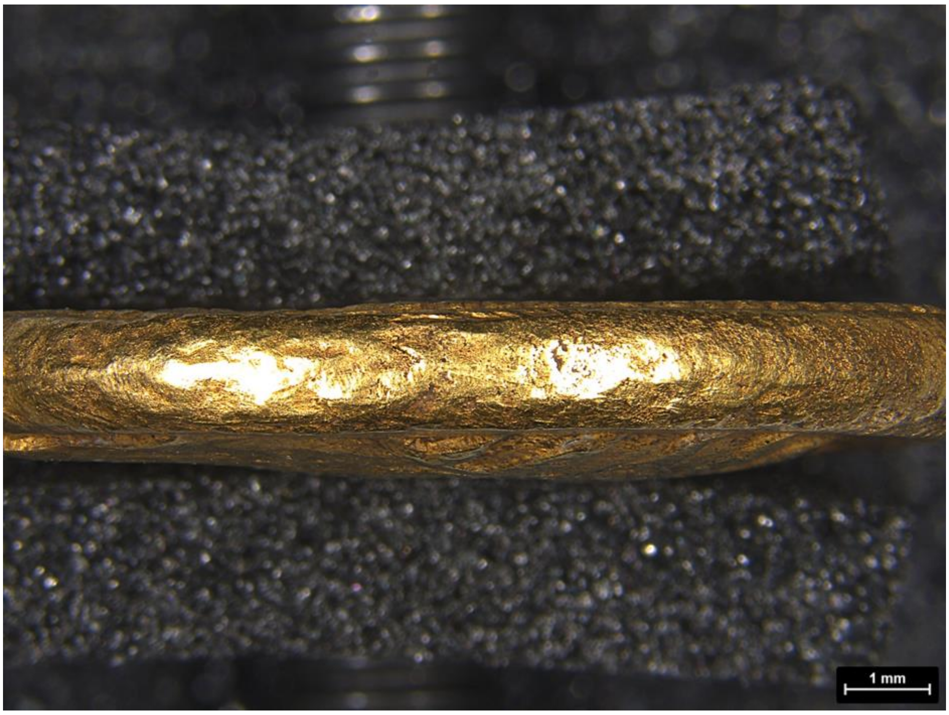

Figure S.1. 67 Edge, second image

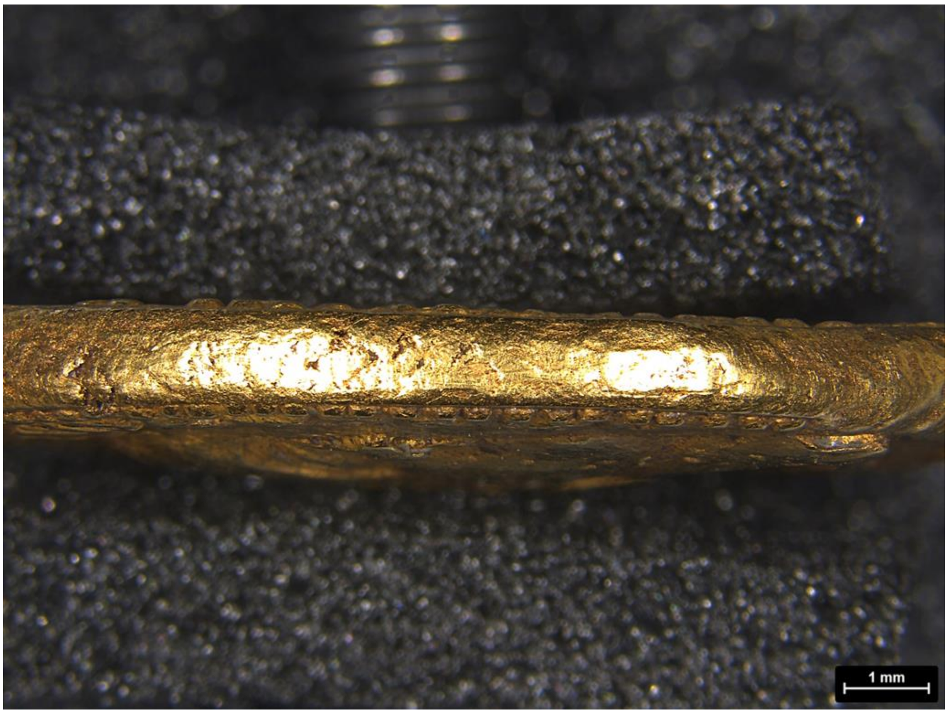

Figure S.1. 68 Edge, third image

S.1.6.3 Additional images

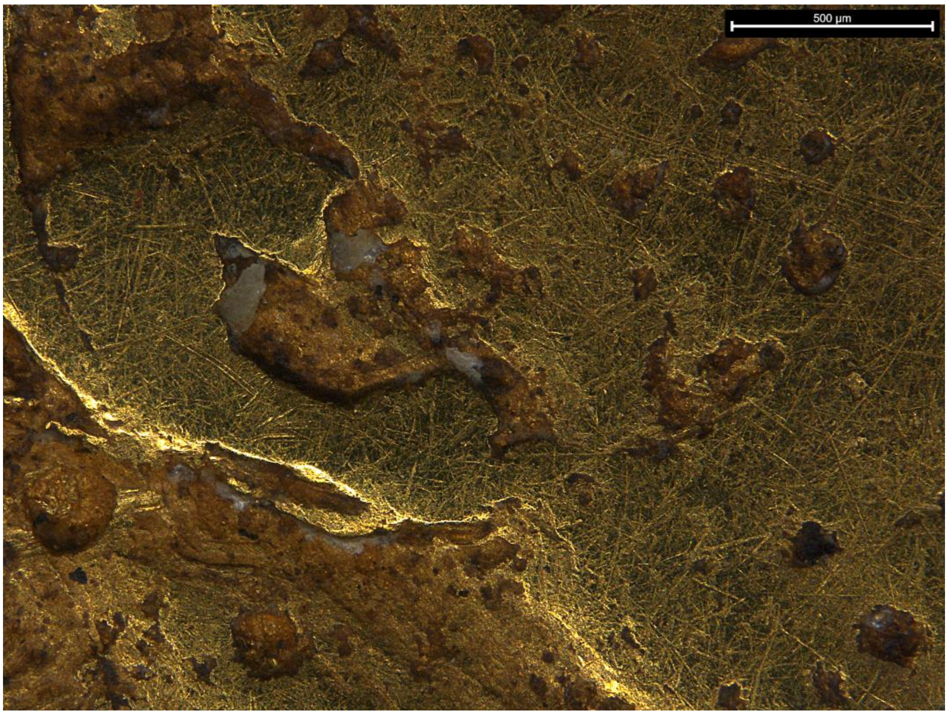

Figure S.1. 69 Detail of obverse showing scratches and wax

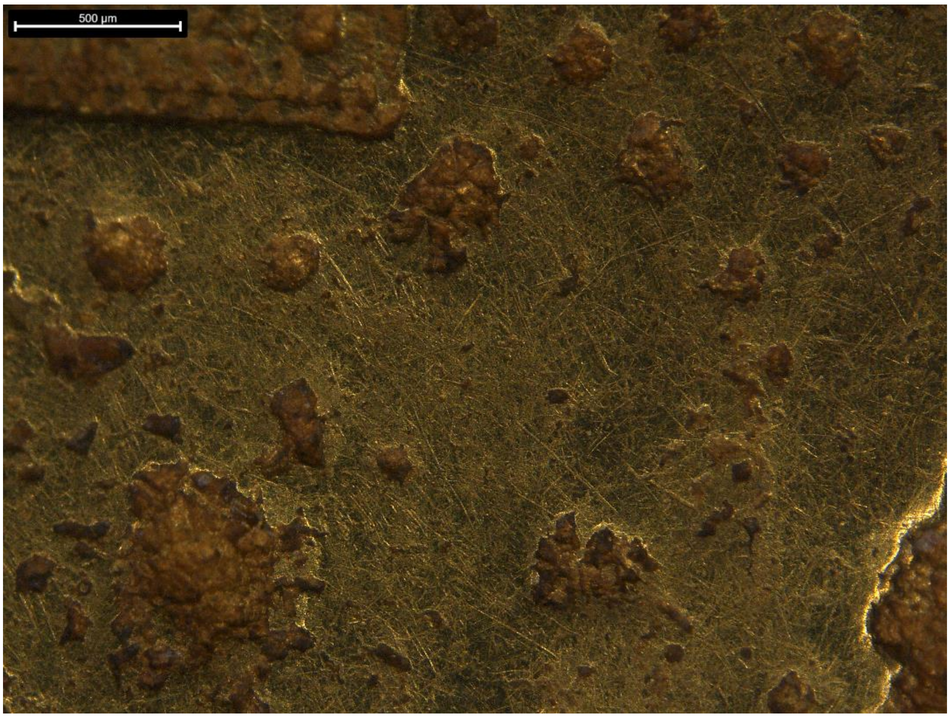

Figure S.1. 70 Detail of obverse showing scratches under first directional illumination

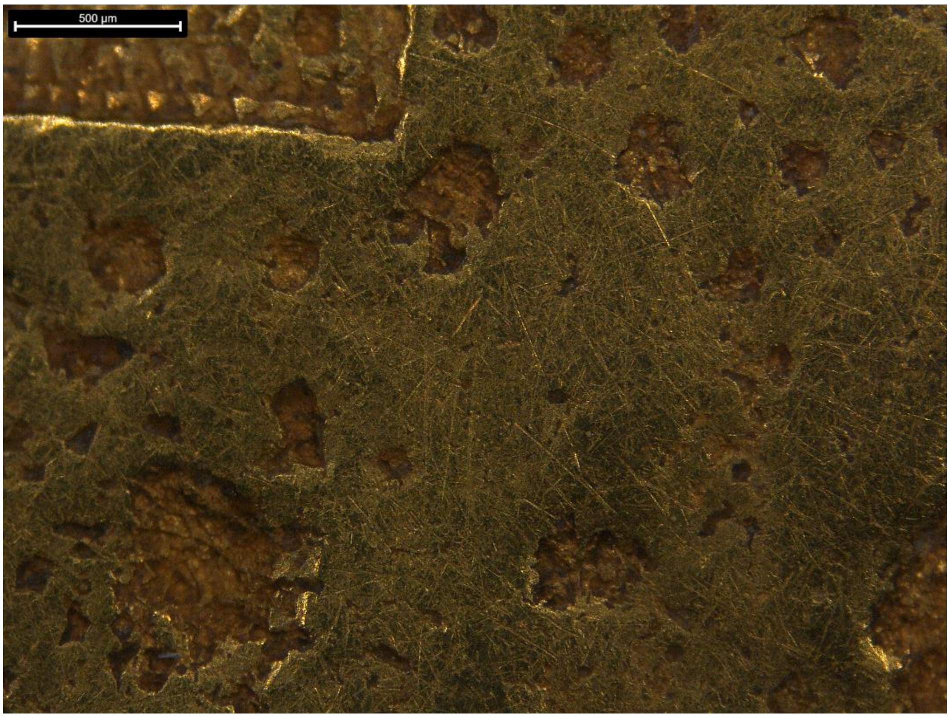

Figure S.1. 71 Detail of obverse showing scratches under second directional illumination

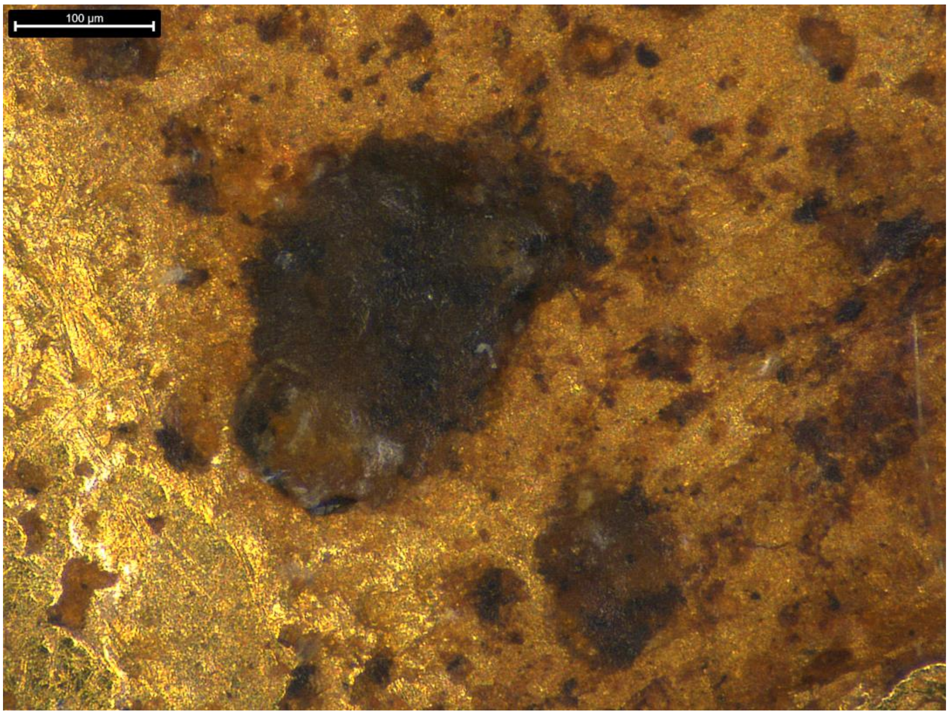

Figure S.1. 72 Detail of earthen deposit on obverse

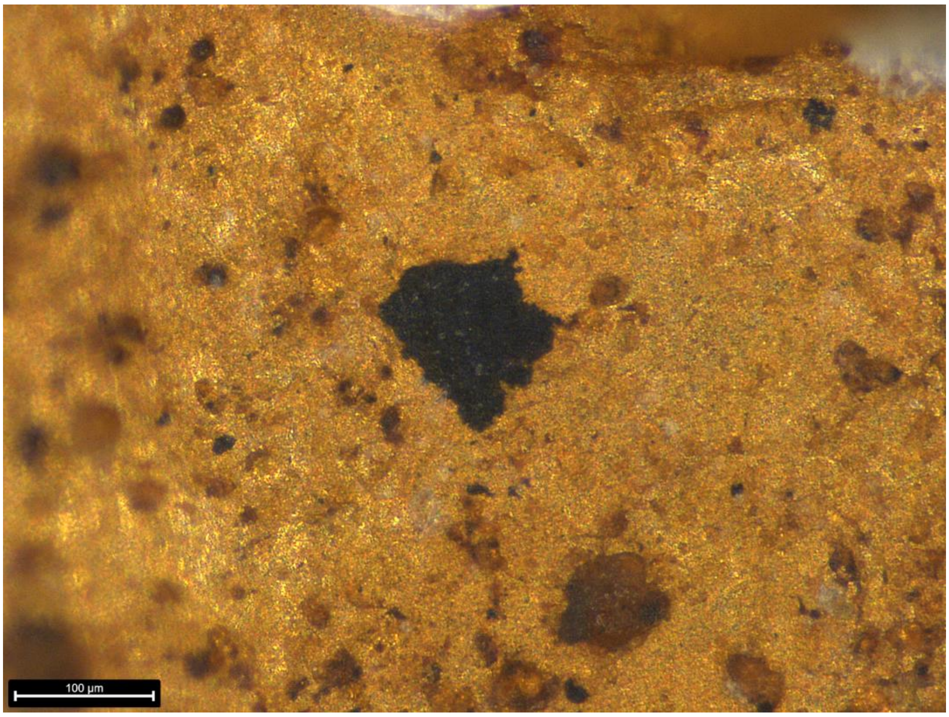

Figure S.1. 73 Detail of earthen deposit on obverse

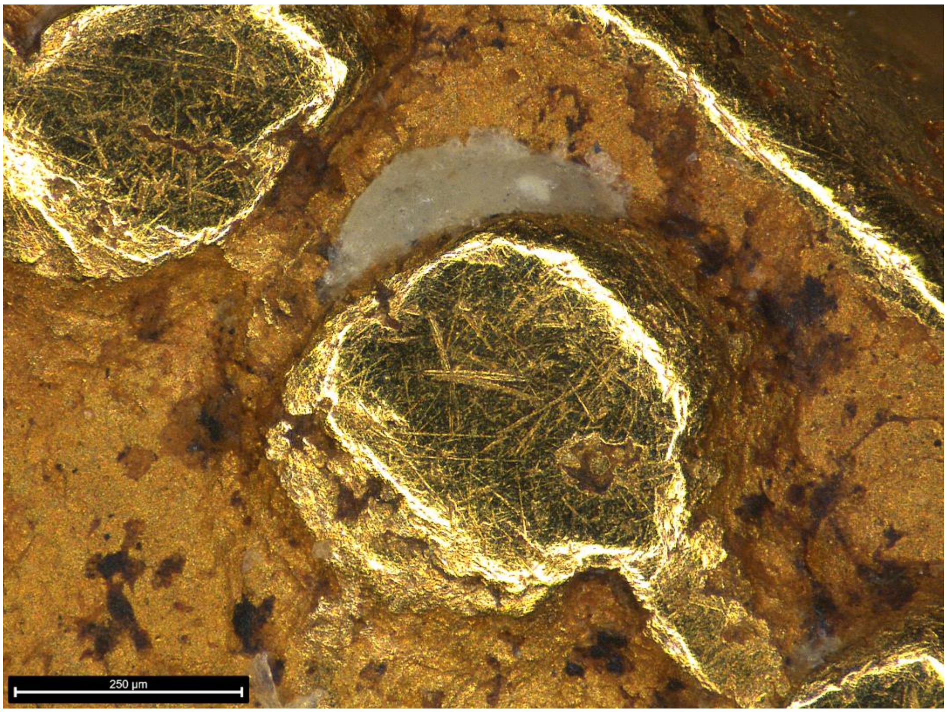

Figure S.1. 74 Detail of beading, wax and earthen deposits on obverse

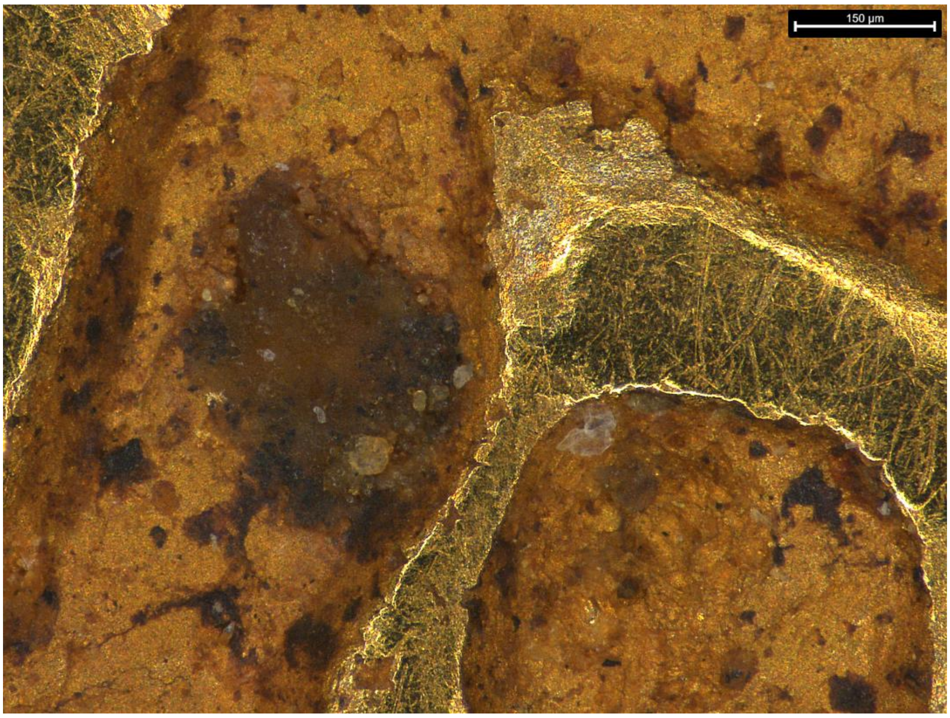

Figure S.1. 75 Detail of lettering, fractures and earthen deposits on obverse

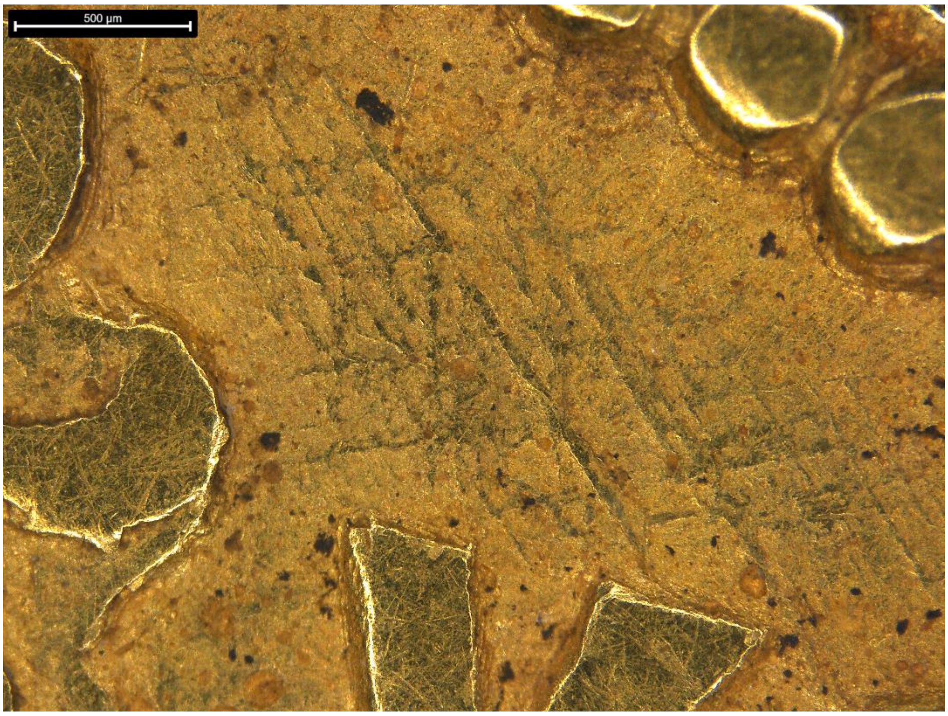

Figure S.1. 76 Detail of flat field in reverse

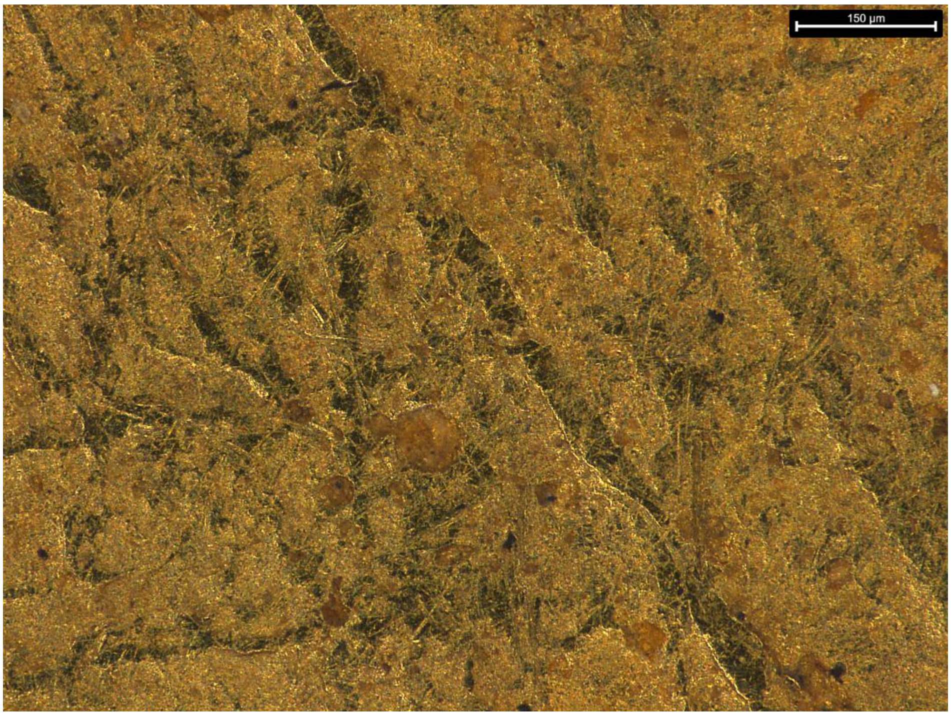

Figure S.1. 77 Detail of lines in flat field on reverse
