# Supplementary material for: Authenticating coins of the ‘Roman emperor’ Sponsian
Source: PLoS One. 2022 Nov 23;17(11):e0274285. doi: 10.1371/journal.pone.0274285 (PMC9683583; doi:10.1371/journal.pone.0274285)
Supplement: S2 File — (PDF) [file pone.0274285.s002.pdf]

Supporting Information 2

Ultraviolet (UV) imaging

Contents

S.2.1 Coin GLAHM:29540 (Genuine Gordian III aureus) 2

Figure S.2. 1 Obverse of Coin GLAHM:29540 2

Figure S.2. 2 Reverse of Coin GLAHM:29540 2

S.2.2 Coin GLAHM:29697 (Genuine Philip I aureus) 3

Figure S.1. 13 Obverse of Coin GLAHM:29697 3

Figure S.1. 14 Reverse of Coin GLAHM:29697 3

S.2.3 Coin GLAHM:29596 (Questionable Gordian III medallion / binio) 4

Figure S.1. 23 Obverse of Coin GLAHM:29596 4

Figure S.1. 24 Reverse of Coin GLAHM:29596 4

S.1.4 Coin GLAHM:29820 (Questionable Philip I medallion) 5

Figure S.1. 35 Obverse of Coin GLAHM:29820 5

Figure S.1. 36 Reverse of Coin GLAHM:29820 5

S.1.5 Coin GLAHM:29821 (Questionable Philip I medallion) 6

Figure S.1. 46 Obverse of Coin GLAHM:29821 6

Figure S.1. 47 Reverse of Coin GLAHM:29821 6

S.1.6 Coin GLAHM:40333 (Questionable Sponsian medallion) 7

Figure S.1. 60 Obverse of Coin GLAHM:40333 7

Figure S.1. 61 Reverse of Coin GLAHM:40333 7

S.2.1 Coin GLAHM:29540 (Genuine Gordian III aureus)

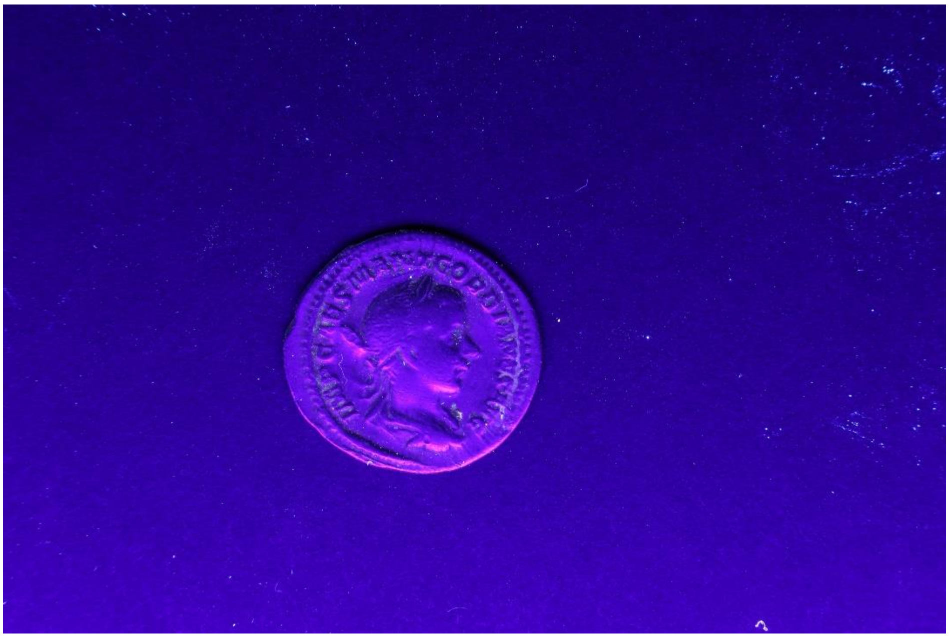

Figure S.2. 1 Obverse of Coin GLAHM:29540

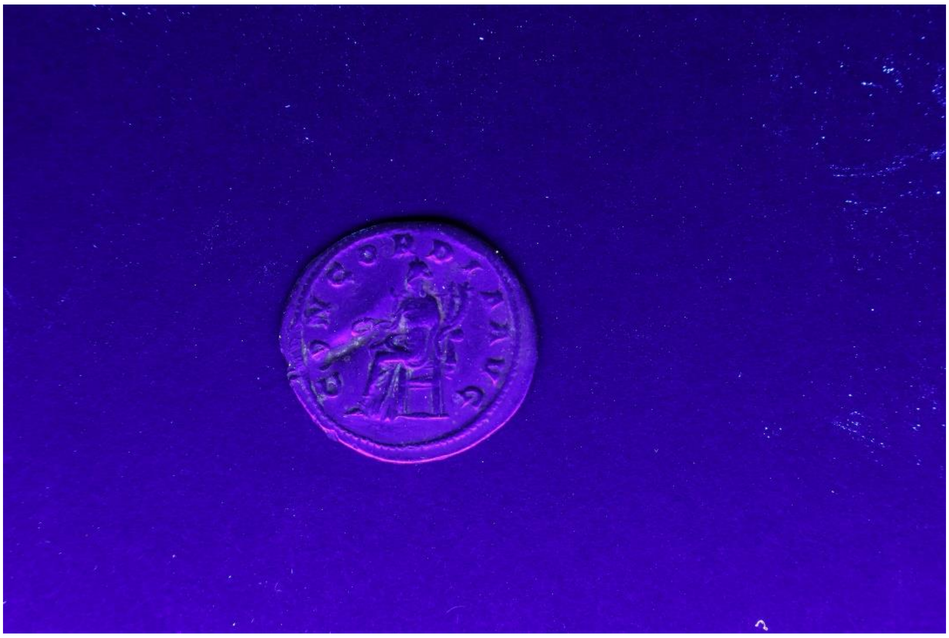

Figure S.2. 2 Reverse of Coin GLAHM:29540

S.2.2 Coin GLAHM:29697 (Genuine Philip I aureus)

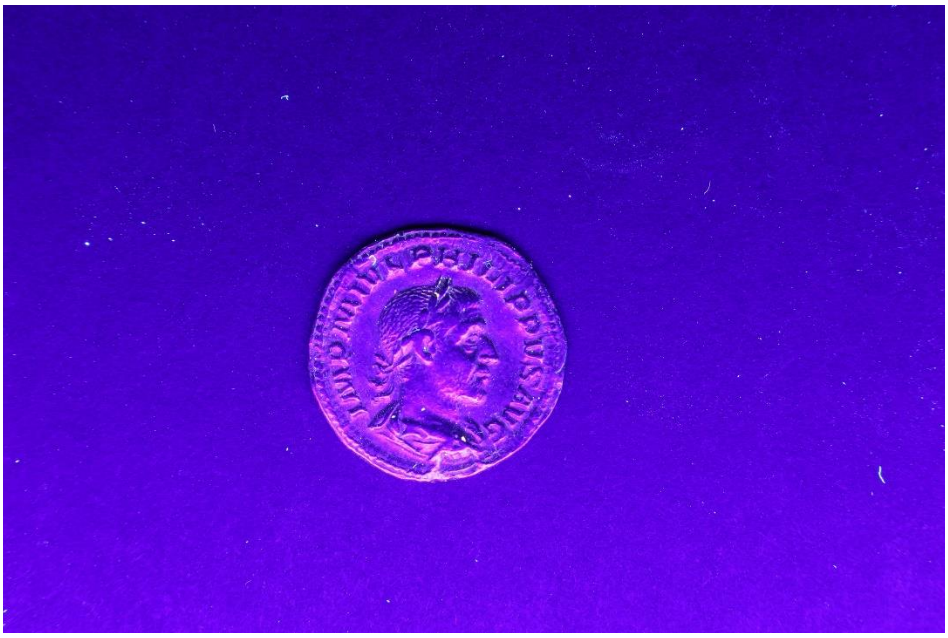

Figure S.1. 3 Obverse of Coin GLAHM:29697

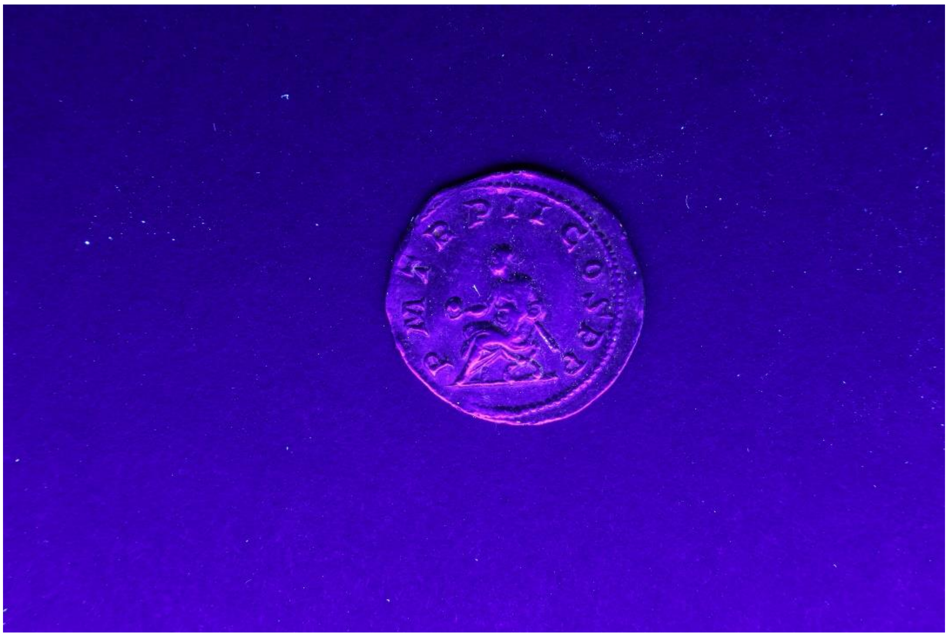

Figure S.1. 4 Reverse of Coin GLAHM:29697

S.2.3 Coin GLAHM:29596 (Questionable Gordian III medallion / binio)

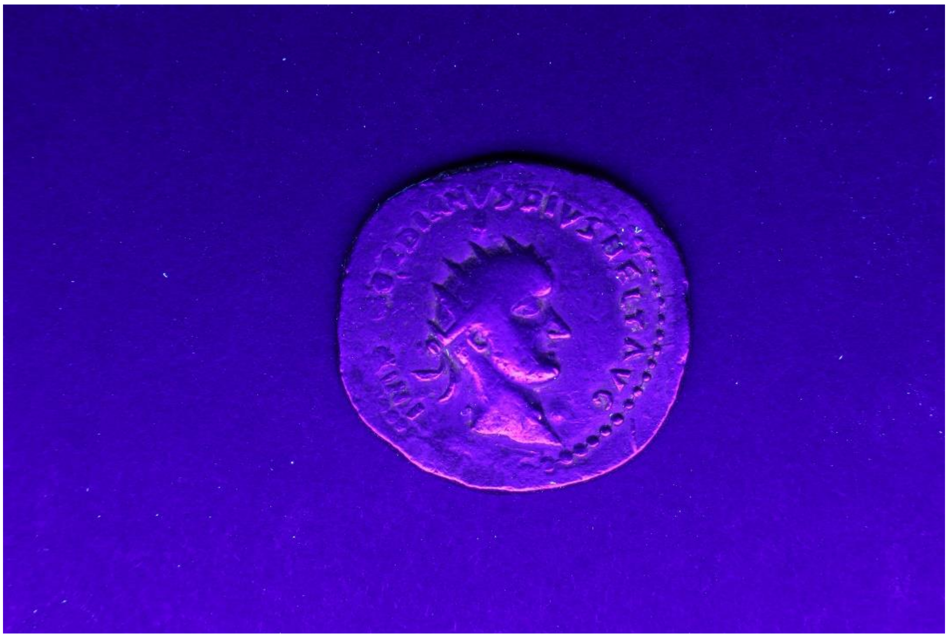

Figure S.1. 5 Obverse of Coin GLAHM:29596

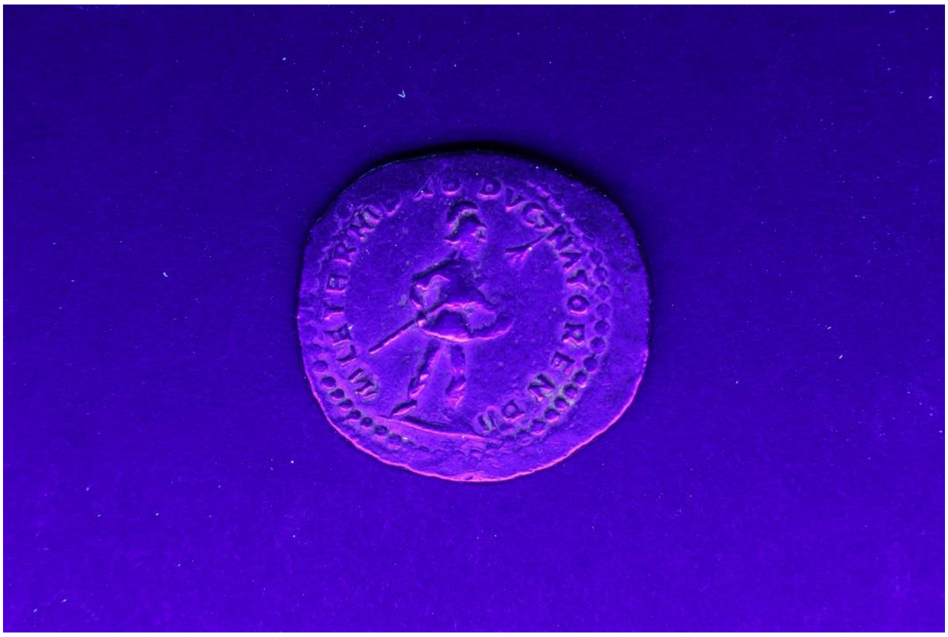

Figure S.1. 6 Reverse of Coin GLAHM:29596

S.2.4 Coin GLAHM:29820 (Questionable Philip I medallion)

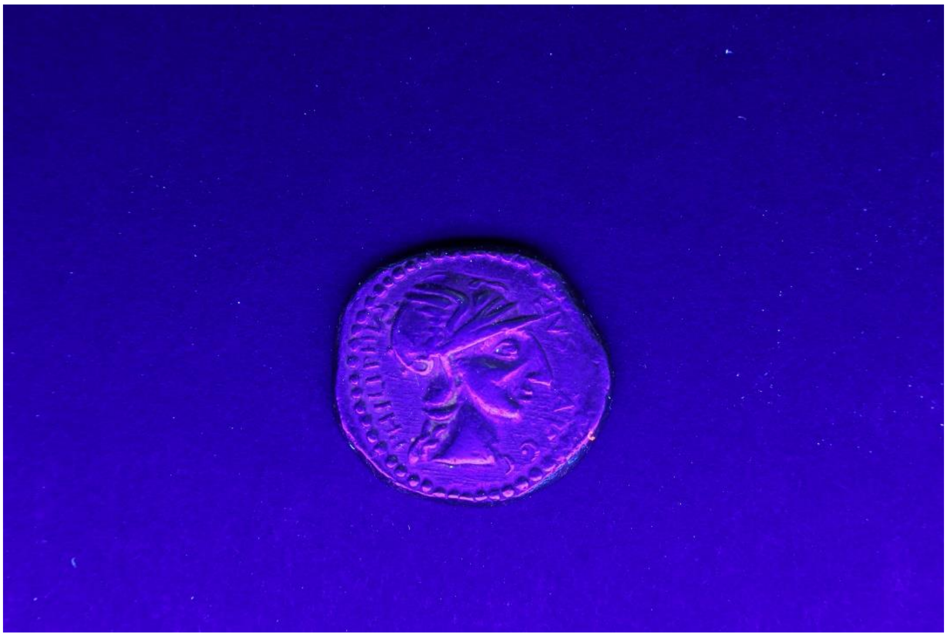

Figure S.1. 7 Obverse of Coin GLAHM:29820

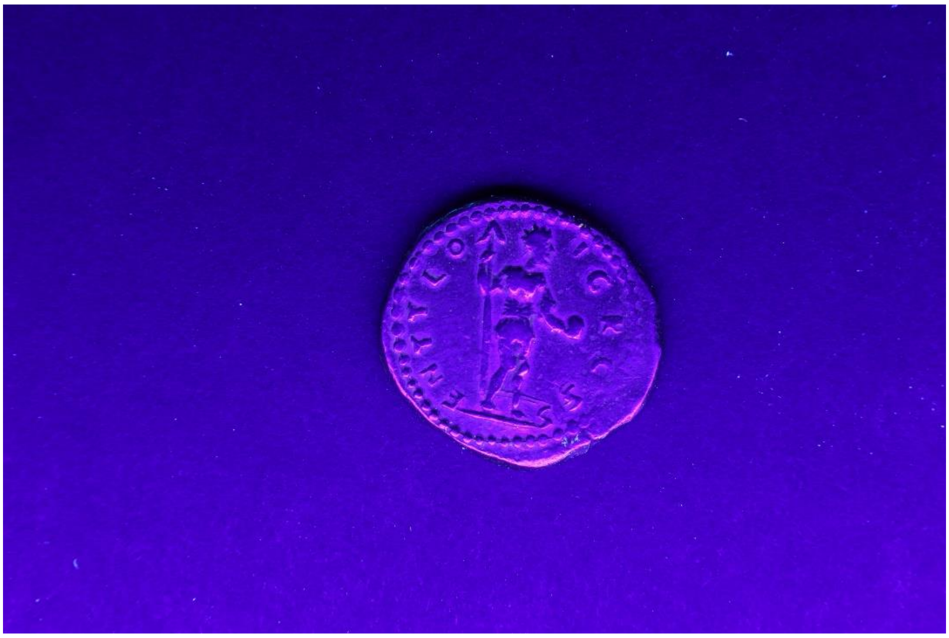

Figure S.1. 8 Reverse of Coin GLAHM:29820

S.2.5 Coin GLAHM:29821 (Questionable Philip I medallion)

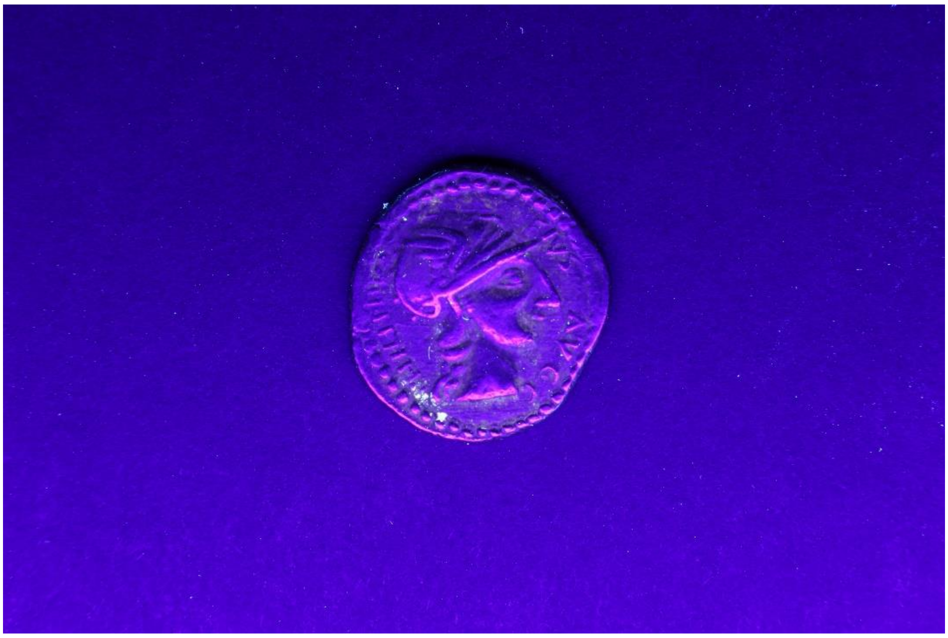

Figure S.1. 9 Obverse of Coin GLAHM:29821

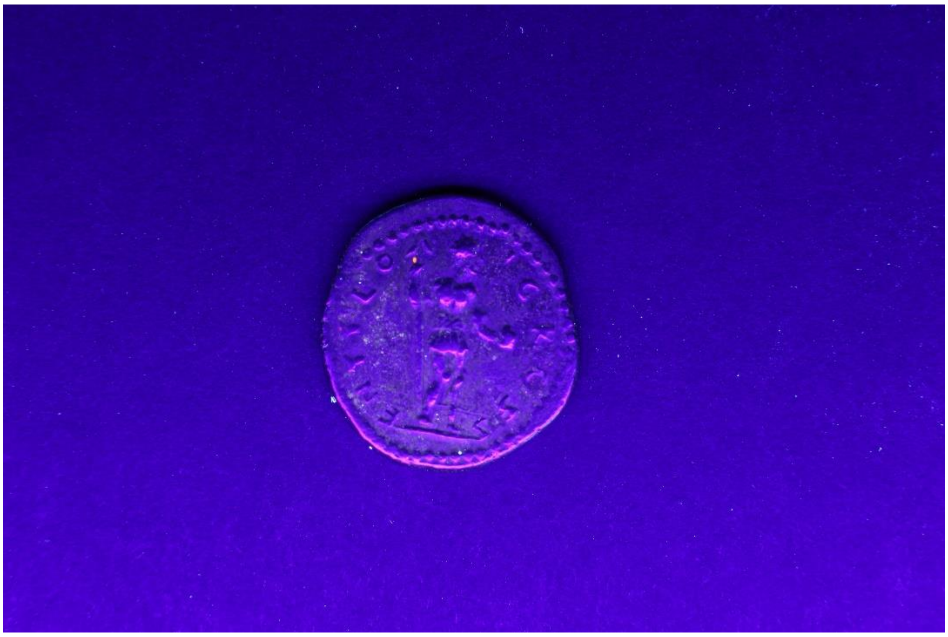

Figure S.1. 10 Reverse of Coin GLAHM:29821

S.2.6 Coin GLAHM:40333 (Questionable Sponsian medallion)

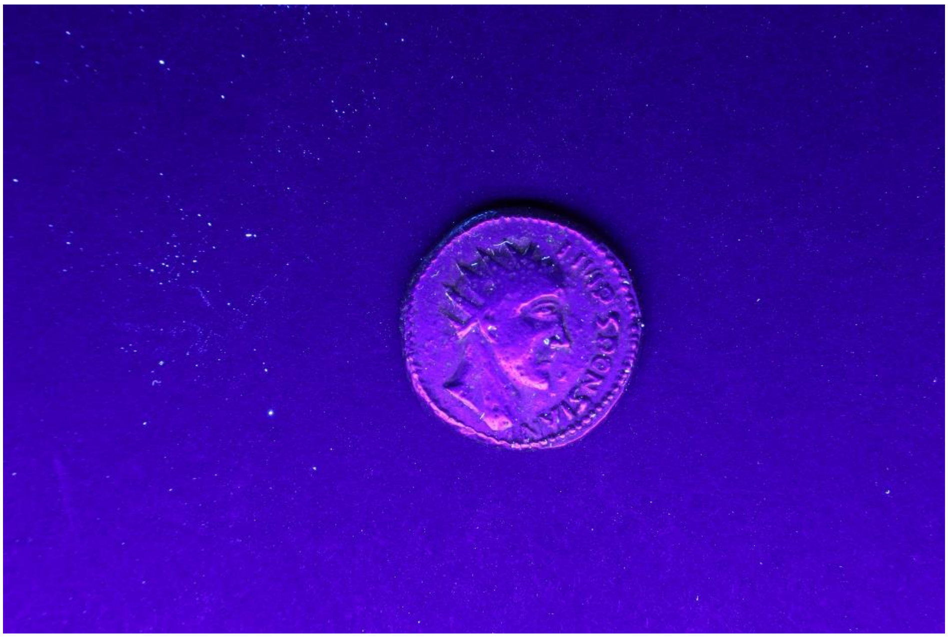

Figure S.1. 11 Obverse of Coin GLAHM:40333

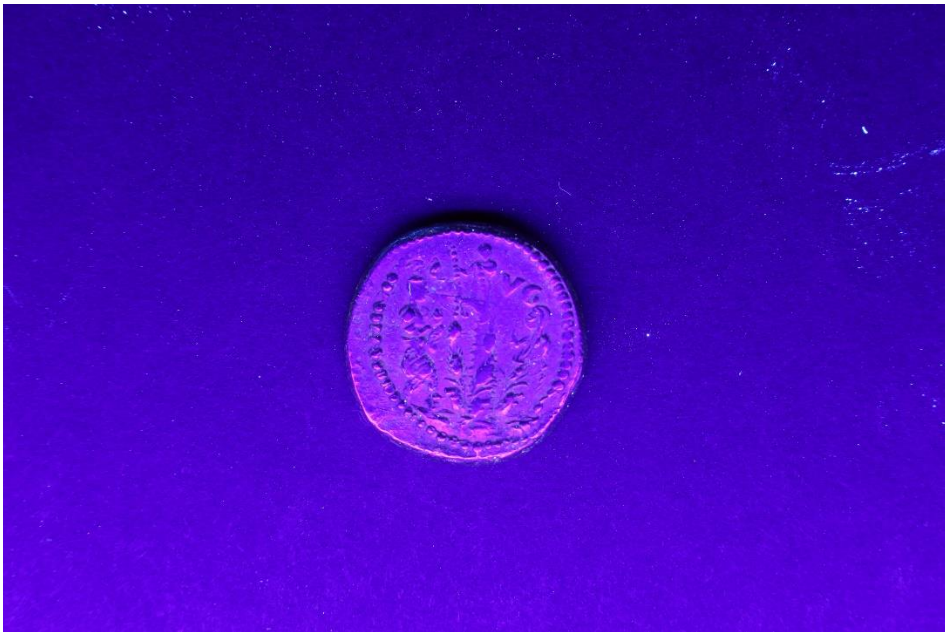

Figure S.1. 12 Reverse of Coin GLAHM:40333
