# Supplementary material for: Authenticating coins of the ‘Roman emperor’ Sponsian
Source: PLoS One. 2022 Nov 23;17(11):e0274285. doi: 10.1371/journal.pone.0274285 (PMC9683583; doi:10.1371/journal.pone.0274285)
Supplement: S3 File — (PDF) [file pone.0274285.s003.pdf]

Supporting Information 3: Scanning electron microscopy (SEM) and Energy Dispersive X-ray (EDX) spectra

Contents

S.3.1 Coin GLAHM:29540 (Genuine Gordian III aureus)....6

S.3.1.1 Gallery of surface images....6

Figure S.3. 1 Key to images....6

Figure S.3. 2 Image of area of emperor’s head at 500 x magnification....6

Figure S.3. 3 Image of area of emperor’s head at 1000 x magnification....7

Figure S.3. 4 Image of area of emperor’s head at 1000 x magnification....7

Figure S.3. 5 Image of area of emperor’s head at 5000 x magnification....8

Figure S.3. 6 Second image of exposed surface at 5000 x magnification....8

Figure S.3. 7 Image of exposed surface at 20000 x magnification....9

S.3.1.2 Metal composition....10

Figure S.3. 8 Area of coin surface showing sites of individual analyses of metal composition....10

Figure S.3. 9 Spectrum 1 on Coin GLAHM:29540....11

Figure S.3. 10 Spectrum 2 on Coin GLAHM:29540....11

Figure S.3. 11 Spectrum 3 on Coin GLAHM:29540....12

Figure S.3. 12 Spectrum 4 on Coin GLAHM:29540....12

Figure S.3. 13 Spectrum 5 on Coin GLAHM:29540....13

Figure S.3. 14 Spectrum 6 on Coin GLAHM:29540....13

Figure S.3. 15 Spectrum 7 on Coin GLAHM:29540....14

Table S.3. 1 Summary of results for metal composition of GLAHM:29540....14

S.3.1.3 Small superficial patches....15

Figure S.3. 16 Area of Area of coin surface from the area of the emperor’s head showing dark patches....15

Figure S.3. 17 Sites of individual analyses....15

Figure S.3. 18 Spectrum 17 on Coin GLAHM:29540....16

Figure S.3. 19 Spectrum 18 on Coin GLAHM:29540....16

Figure S.3. 20 Spectrum 19 on Coin GLAHM:29540....17

Figure S.3. 21 Spectrum 20 on Coin GLAHM:29540....17

S.3.1.4 Larger earthen deposits....18

Figure S.3. 22 LM image of the area within the ‘O’ of the obverse legend....18

Figure S.3. 23 Area of coin surface showing area within the 'O' of the obverse legend where Spectra 21 and 22 were collected (corresponding to the upper part of Figure S13) 19
Figure S.3. 24 Spectrum 21 on Coin GLAHM:29540 19
Figure S.3. 25 Spectrum 22 on Coin GLAHM:29540 20
S.3.2 Coin GLAHM:29697 (Genuine Philip I aureus) 21
S.3.2.1 Gallery of surface images 21
Figure S.3. 26 Area of emperor's head at 500x magnification 21
Figure S.3. 27 Area of emperor's head at 1000x magnification 21
Figure S.3. 28 Area of emperor's head at 5000x magnification 22
Figure S.3. 29 Area of emperor's head at 10000x magnification 22
S.3.3 Coin GLAHM:29596 (Questionable Gordian III medallion) 23
S.3.3.1 Gallery of surface images 23
Figure S.3. 30 Area of emperor's head at 500 x magnification 23
Figure S.3. 31 Area of emperor's head at 1000 x magnification 23
Figure S.3. 32 Area of emperor's head at 5000 x magnification 24
Figure S.3. 33 Area of emperor's head at 10000 x magnification 24
S.3.3.2 Metal composition 25
Figure S.3. 34 Exposed area of the emperor's head showing the location of Spectra 42-47 25
Figure S.3. 35 Exposed area of the emperor's head showing the location of Spectra 48-54 26
Figure S.3. 36 Spectrum 45 on Coin GLAHM:29596 26
Figure S.3. 37 Spectrum 46 on Coin GLAHM:29596 27
Figure S.3. 38 Spectrum 47 on Coin GLAHM:29596 27
Figure S.3. 39 Spectrum 51 on Coin GLAHM:29596 27
Figure S.3. 40 Spectrum 52 on Coin GLAHM:29596 28
Figure S.3. 41 Spectrum 53 on Coin GLAHM:29596 28
Figure S.3. 42 Spectrum 54 on Coin GLAHM:29596 29
Table S. 3.2 Analyses of metal on Coin GLAHM:29596 29
S.3.3.3 Small superficial patches 30
Figure S.3. 43 Spectrum 42 on Coin GLAHM:29596 30
Figure S.3. 44 Spectrum 43 on Coin GLAHM:29596 31
Figure S.3. 45 Spectrum 48 on Coin GLAHM:29596 31
Figure S.3. 46 Spectrum 49 on Coin GLAHM:29596 32
Figure S.3. 47 Spectrum 50 on Coin GLAHM:29596 32
S.3.3.4 Larger earthen deposits 33

Figure S.3. 48 LM photograph of the area investigated 33
Figure S.3. 49 Figure Area of the obverse letter ‘S’ and its earthen deposits 34
Figure S.3. 50 Area of coin surface showing the beside the ‘S’ of the obverse legend 34
Figure S.3. 51 Spectrum 23 on Coin GLAHM:29596.. 35
Figure S.3. 52 Spectrum 24 on Coin GLAHM:29596.. 35
Figure S.3. 53 First investigated area of mineralization within the letter ‘S’........................ 36
Figure S.3. 54 High magnification image of the area to the left of Spectrum 24 in Figure S.3.33.. 36
Figure S.3. 55 Spectrum 28 on Coin GLAHM:29596.. 37
Figure S.3. 56 Spectrum 25 on Coin GLAHM:29596.. 38
Figure S.3. 57 Spectrum 26 on Coin GLAHM:29596.. 38
Figure S.3. 58 Spectrum 27 on Coin GLAHM:29596.. 39
Figure S.3. 59 Spectrum 32 on Coin GLAHM:29596.. 39
Figure S.3. 60 Spectrum 33 on Coin GLAHM:29596.. 40
Figure S.3. 61 Spectrum 34 on Coin GLAHM:29596.. 40
Figure S.3. 62 Spectrum 35 on Coin GLAHM:29596.. 41
Figure S.3. 63 Spectrum 36 on Coin GLAHM:29596.. 41
Figure S.3. 64 Spectrum 29 on Coin GLAHM:29596.. 42
Figure S.3. 65 Spectrum 30 on Coin GLAHM:29596.. 43
Figure S.3. 66 Spectrum 31 on Coin GLAHM:29596.. 43
Figure S.3. 67 Second investigated area of mineralization within the letter ‘S’ 44
Figure S.3. 68 High magnification image of the area to the top right of Spectrum 24 in Figure S33.. 44
Figure S.3. 69 Spectrum 41 on Coin GLAHM:29596.. 45
Figure S.3. 70 Spectrum 39 on Coin GLAHM:29596.. 46
Figure S.3. 71 Spectrum 37 on Coin GLAHM:29596.. 46
Figure S.3. 72 Spectrum 38 on Coin GLAHM:29596.. 47
Figure S.3. 73 Spectrum 40 on Coin GLAHM:29596.. 47
S.3.4 Coin GLAHM:29820 (Questionable Philip I medallion).. 49
S.3.4.1 Gallery of surface images 49
Figure S.3. 74 Area of Roma’s head at 1000x magnification 49
Figure S.3. 75 Area of Roma’s head at 5000x magnification 49
Figure S.3. 76 Area of Roma’s head at 10000x magnification 50
S.3.4.1 Metal composition 51
Figure S.3. 77 Area of analyses on the worn area of Coin GLAHM:29820........................... 51
Figure S.3. 78 Spectrum 82 on Coin GLAHM:29820.. 52

Figure S.3. 79 Spectrum 83 on Coin GLAHM:29820 52
Figure S.3. 80 Spectrum 84 on Coin GLAHM:29820 52
Table S.3. 3 Analyses of metal on Coin GLAHM:29596 53
S.3.4.2 Superficial deposits 53
Figure S.3. 81 Spectrum 80 on Coin GLAHM:29820 53
Figure S.3. 82 Spectrum 81 on Coin GLAHM:29820 54
S.3.5 Coin GLAHM:29821 (Questionable Philip I medallion) 55
S.3.5.1 Gallery of images 55
Figure S.3. 83 Area of Roma’s head at 500x magnification 55
Figure S.3. 84 Area of Roma’s head at 1000x magnification 55
Figure S.3. 85 Area of Roma’s head at 5000x magnification 56
Figure S.3. 86 Second area of Roma’s head at 5000x magnification 56
Figure S.3. 87 Area of Roma’s head at 10000x magnification 57
S.3.6 Coin GLAHM:40333 (Questionable Sponsian medallion) 58
S.3.6.1 Gallery of images 58
Figure S.3. 88 Key to images 58
Figure S.3. 89 Area of emperor’s head at 500x magnification 58
Figure S.3. 90 Area of emperor’s head at 1000x magnification 59
Figure S.3. 91 Area of emperor’s head at 5000x magnification 59
Figure S.3. 92 Area of emperor’s head at 10000x magnification 60
S.3.6.2 Metal composition 61
Figure S.3. 93 First area of analyses on worn upper surface of Coin GLAHM:40333 61
Figure S.3. 94 Second area of analyses on worn upper surface of Coin GLAHM:40333 61
Figure S.3. 95 Spectrum 64 on Coin GLAHM:40333 62
Figure S.3. 96 Spectrum 65 on Coin GLAHM:40333 62
Figure S.3. 97 Spectrum 76 on Coin GLAHM:40333 63
Figure S.3. 98 Spectrum 77 on Coin GLAHM:40333 63
Figure S.3. 99 Spectrum 78 on Coin GLAHM:40333 64
Figure S.3. 100 Spectrum 79 on Coin GLAHM:40333 64
Table S.3. 4 Metal composition of the six spectra obtained from Coin GLAHM:40333 65
S.3.6.3 Small superficial patches 65
Figure S.3. 101 Spectrum 61 on Coin GLAHM:40333 66
Figure S.3. 102 Spectrum 62 on Coin GLAHM:40333 66
Figure S.3. 103 Spectrum 63 on Coin GLAHM:40333 66
Figure S.3. 104 Spectrum 73 on Coin GLAHM:40333 67

Figure S.3. 105 Spectrum 74 on Coin GLAHM:40333.. 67
Figure S.3. 106 Spectrum 75 on Coin GLAHM:40333.. 68
S.3.6.4 Larger earthen deposits.. 69
Figure S.3. 107 Area of analyses of possible earthen deposits on Coin GLAHM:40333 69
Figure S.3. 108 Areas of Spectra 55 to 60 on Coin GLAHM:40333 70
Figure S.3. 109 Spectrum 55 on Coin GLAHM:40333.. 70
Figure S.3. 110 Spectrum 56 on Coin GLAHM:40333.. 71
Figure S.3. 111 Spectrum 57 on Coin GLAHM:40333.. 71
Figure S.3. 112 Spectrum 58 on Coin GLAHM:40333.. 72
Figure S.3. 113 Spectrum 59 on Coin GLAHM:40333.. 72
Figure S.3. 114 Spectrum 60 on Coin GLAHM:40333.. 73
Figure S.3. 115 Area analysed on Coin GLAHM:40333 74
Figure S.3. 116 Spectrum 67 on Coin GLAHM:40333.. 74
Figure S.3. 117 Spectrum 68 on Coin GLAHM:40333.. 75
Figure S.3. 118 Spectrum 69 on Coin GLAHM:40333.. 75
Figure S.3. 119 Spectrum 70 on Coin GLAHM:40333.. 76
Figure S.3. 120 Area analysed on Coin GLAHM:40333 77
Figure S.3. 121 Spectrum 71 on Coin GLAHM:40333.. 77
Figure S.3. 122 Spectrum 72 on Coin GLAHM:40333.. 78

S.3.1 Coin GLAHM:29540 (Genuine Gordian III aureus)

S.3.1.1 Gallery of surface images

We took a series of images of the coin surface at increasing magnification. These images show wear scratches and cementation spots on an undoubtedly genuine coin.

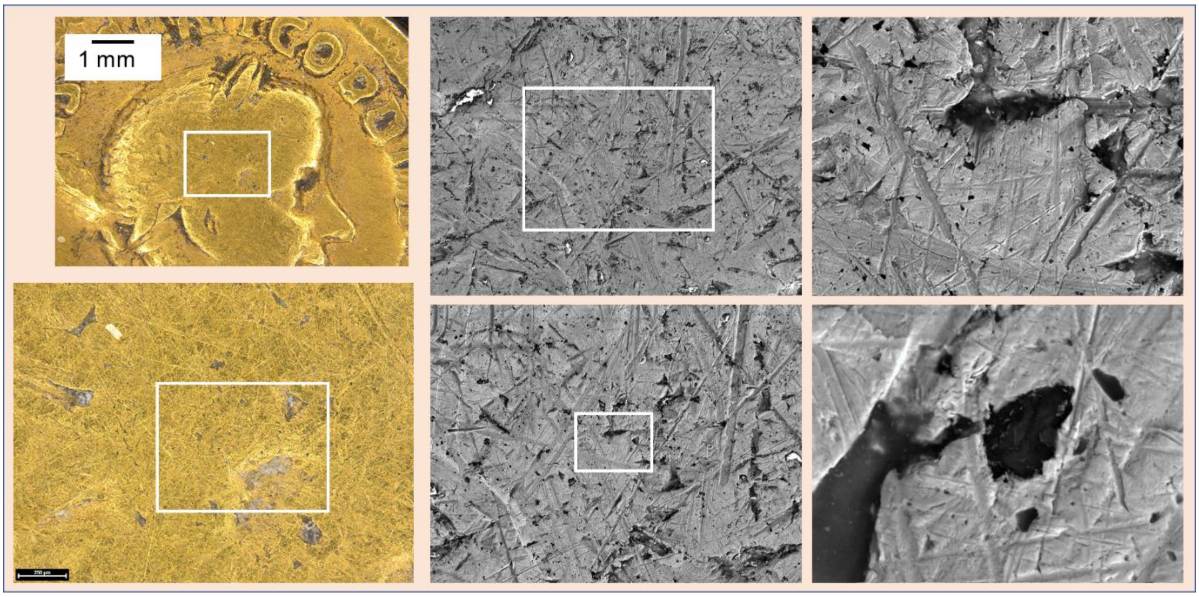

Figure S.3. 1 Key to images

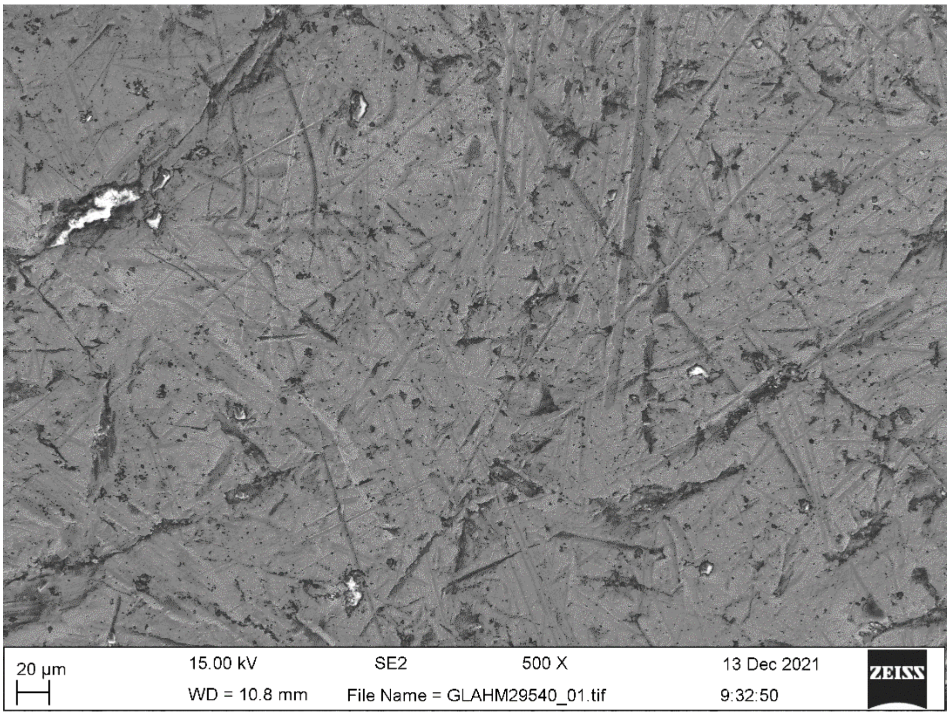

Figure S.3. 2 Image of area of emperor's head at 500 x magnification

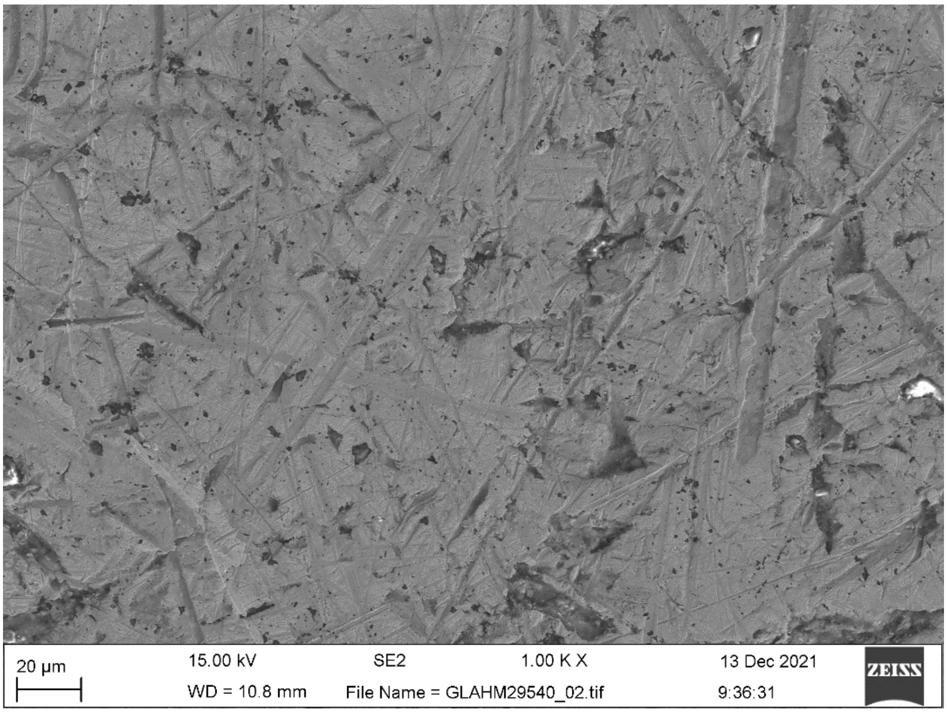

Figure S.3. 3 Image of area of emperor’s head at 1000 x magnification

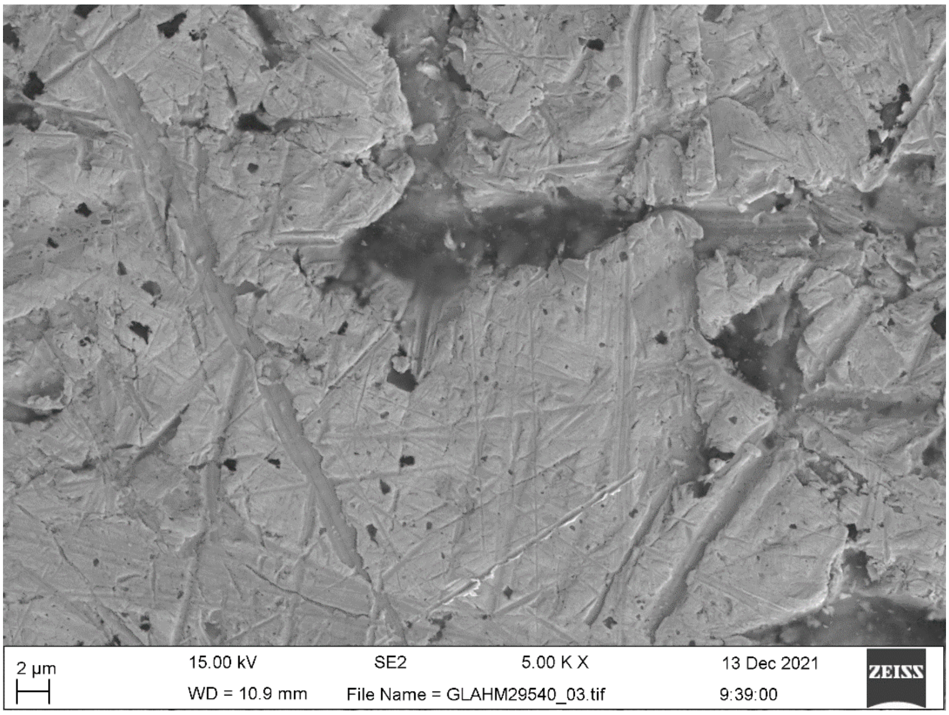

Figure S.3. 4 Image of area of emperor’s head at 1000 x magnification

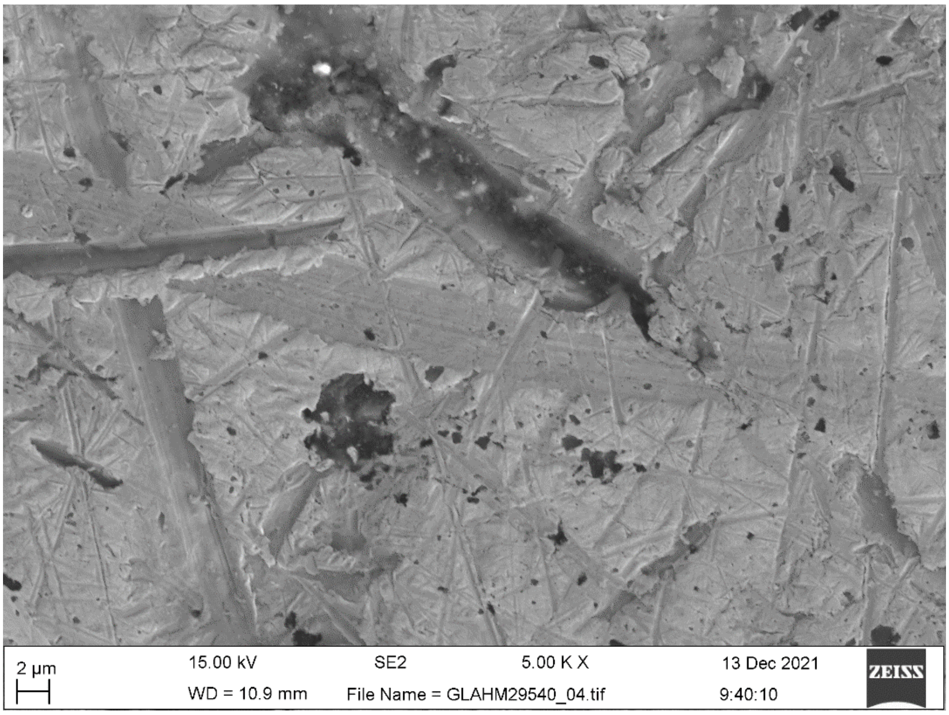

Figure S.3. 5 Image of area of emperor's head at 5000 x magnification

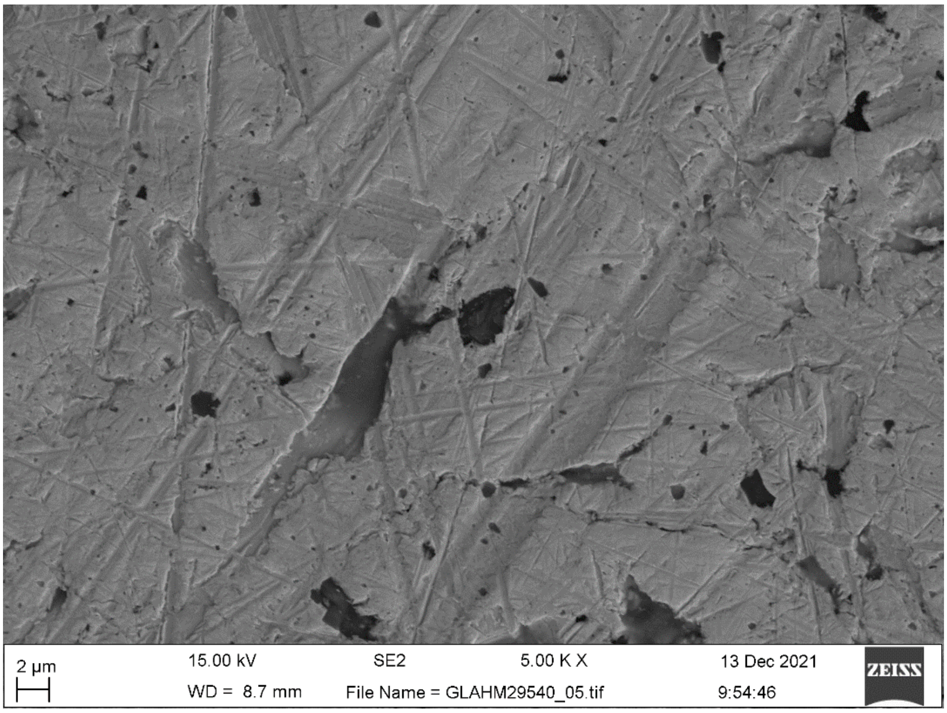

Figure S.3. 6 Second image of exposed surface at 5000 x magnification

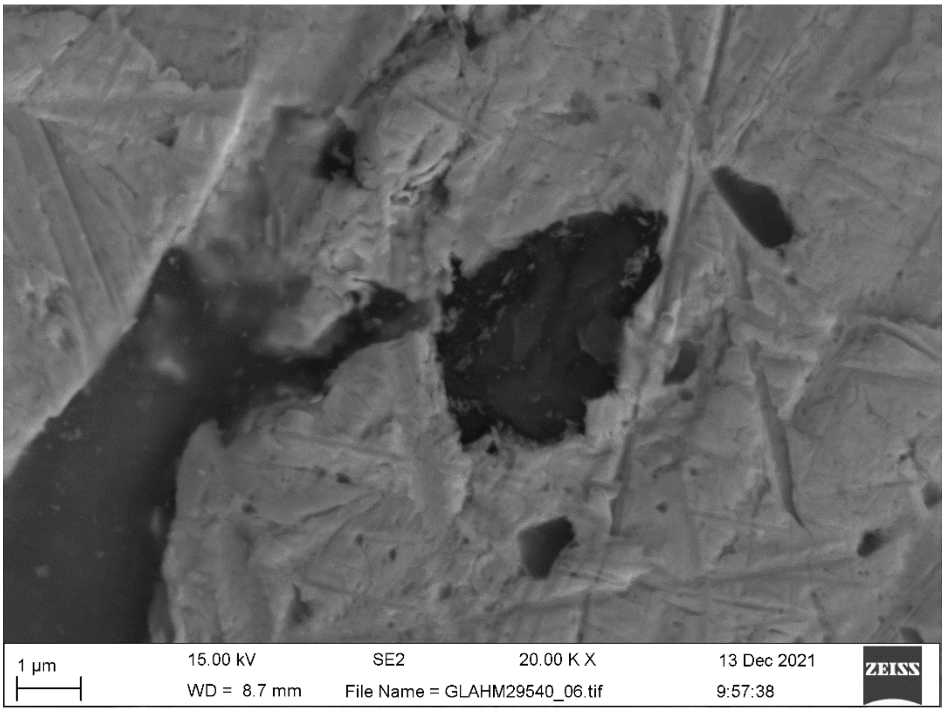

Figure S.3. 7 Image of exposed surface at 20000 x magnification

S.3.1.2 Metal composition

We took seven spectra from an area of the exposed and worn upper surface in the area of the emperor’s head (Figure S.3.1).

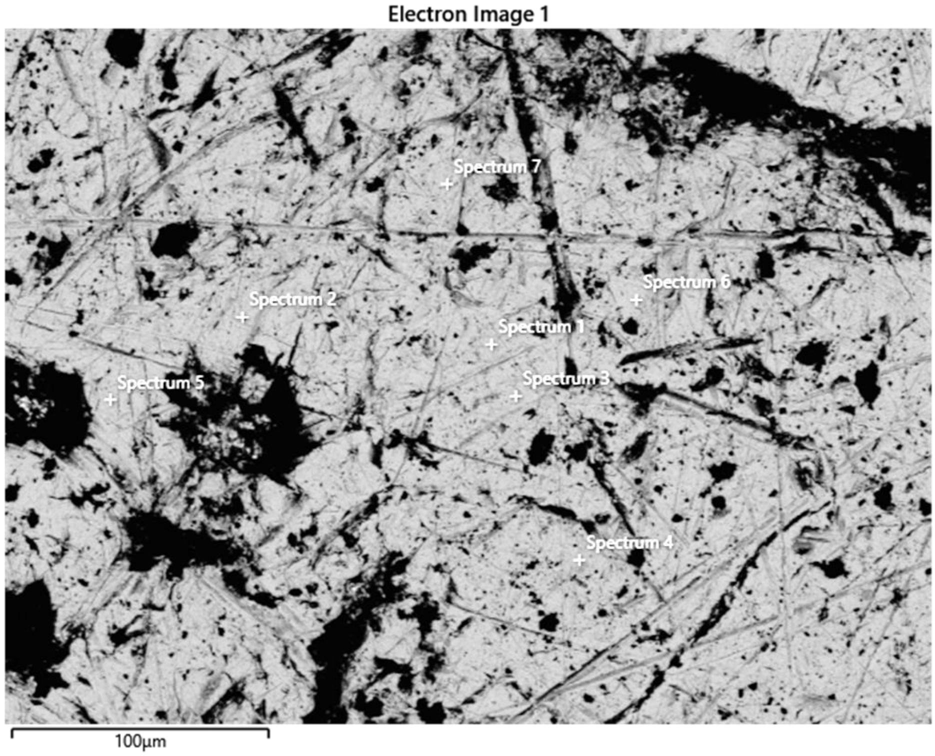

Figure S.3. 8 Area of coin surface showing sites of individual analyses of metal composition

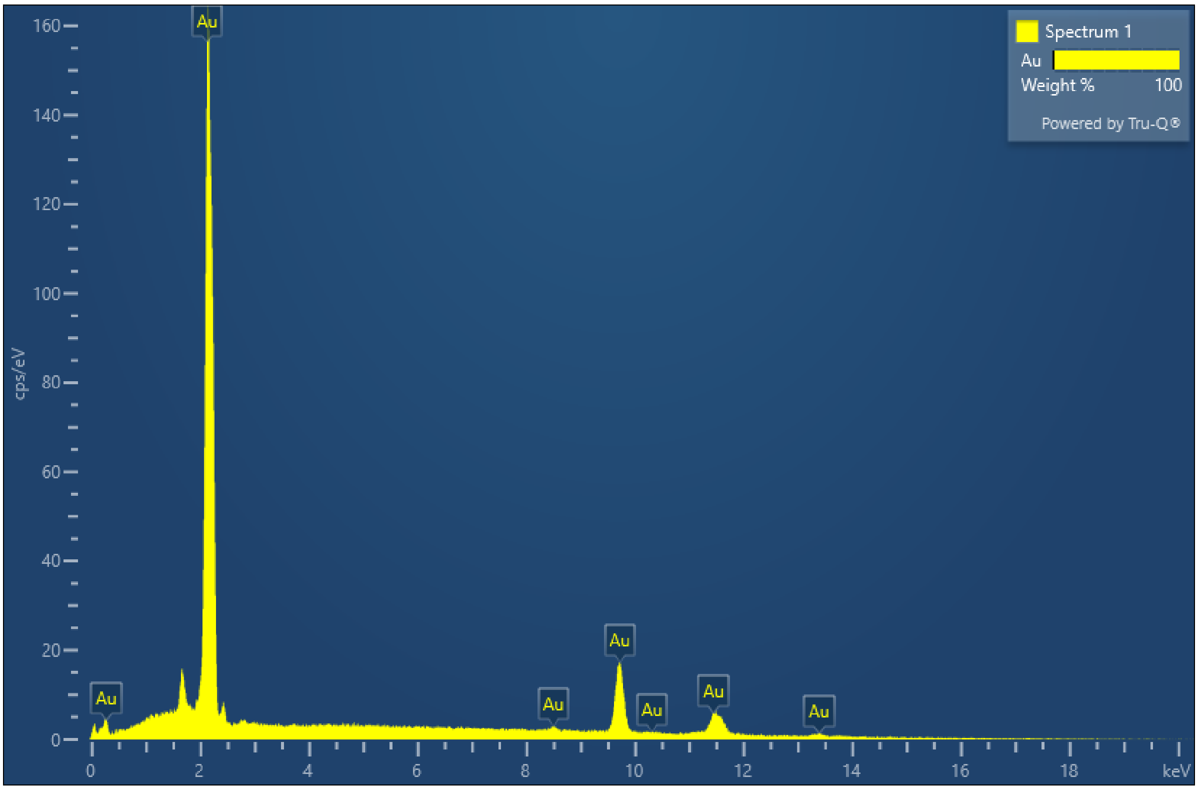

Figure S.3. 9 Spectrum 1 on Coin GLAHM:29540

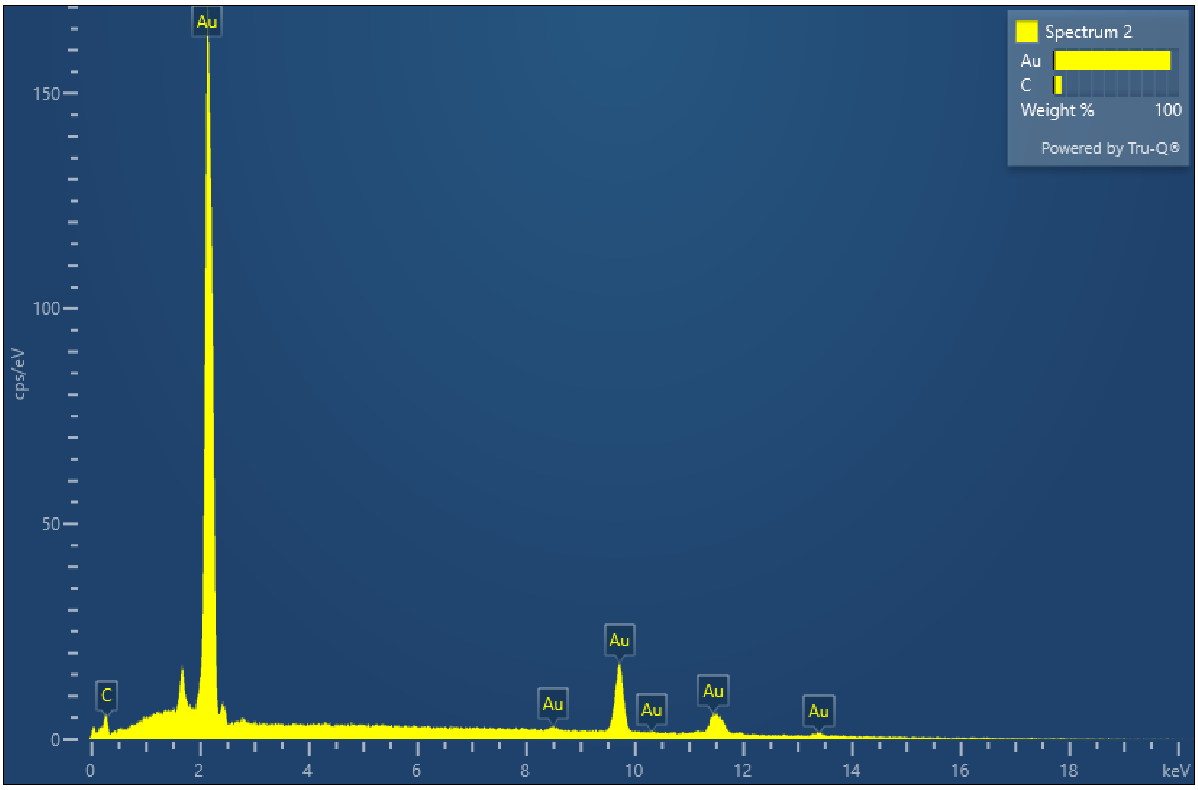

Figure S.3. 10 Spectrum 2 on Coin GLAHM:29540

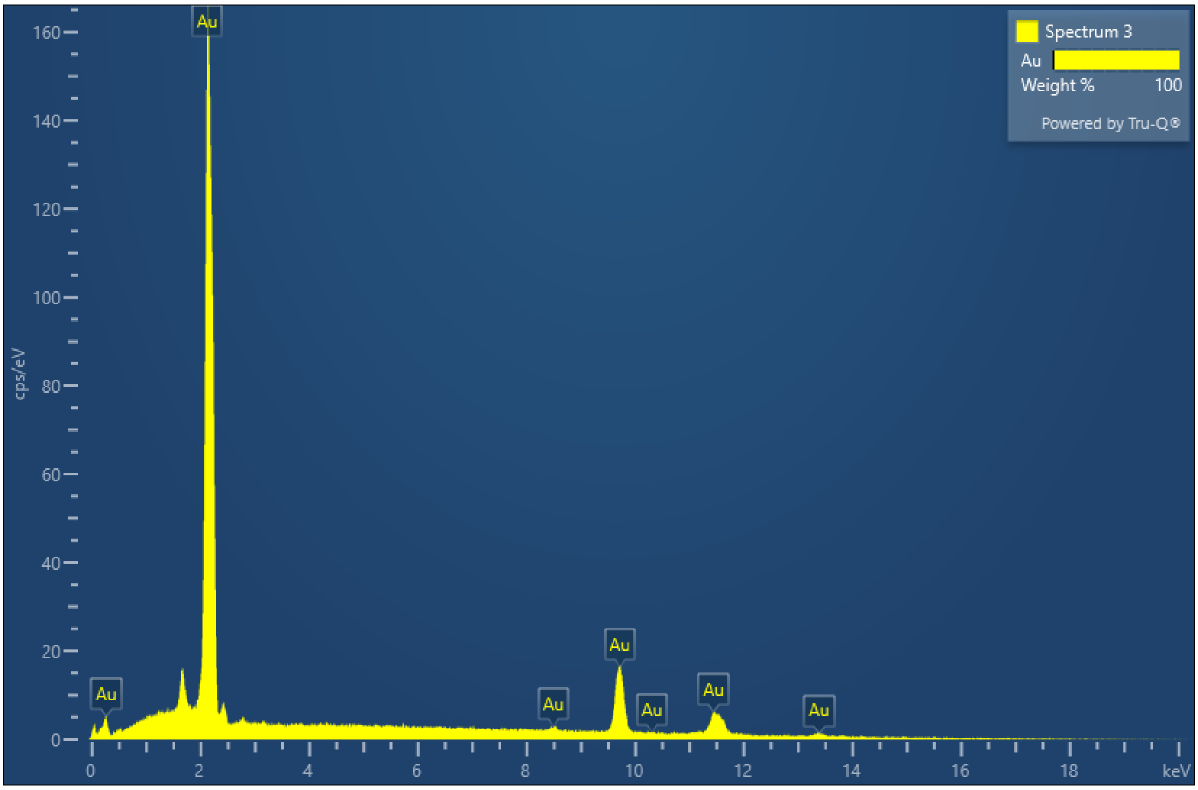

Figure S.3. 11 Spectrum 3 on Coin GLAHM:29540

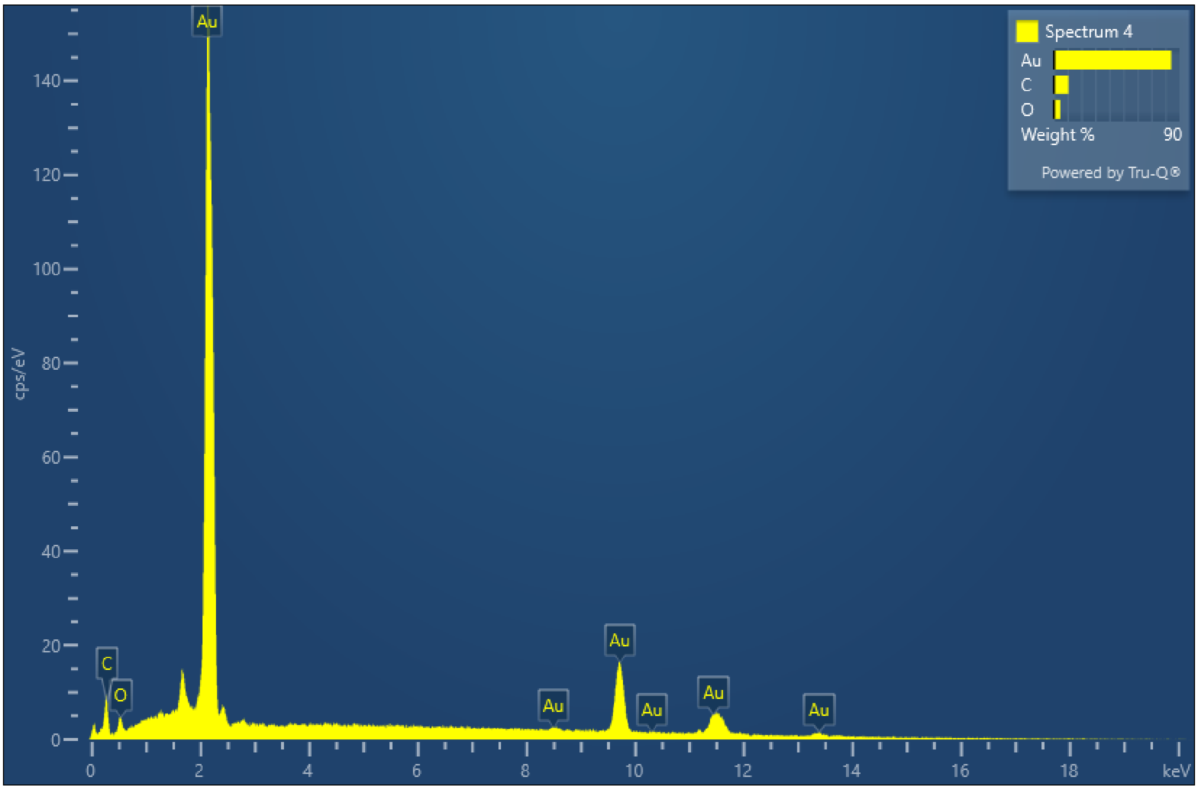

Figure S.3. 12 Spectrum 4 on Coin GLAHM:29540

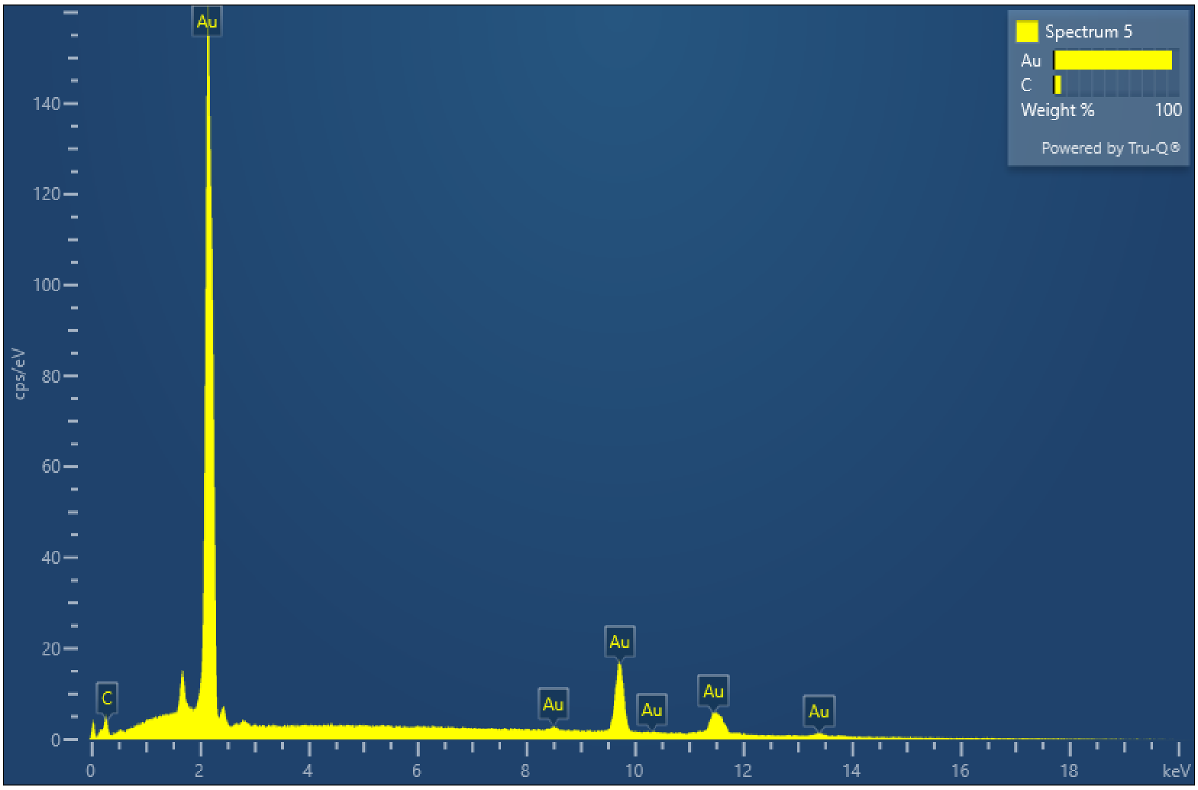

Figure S.3. 13 Spectrum 5 on Coin GLAHM:29540

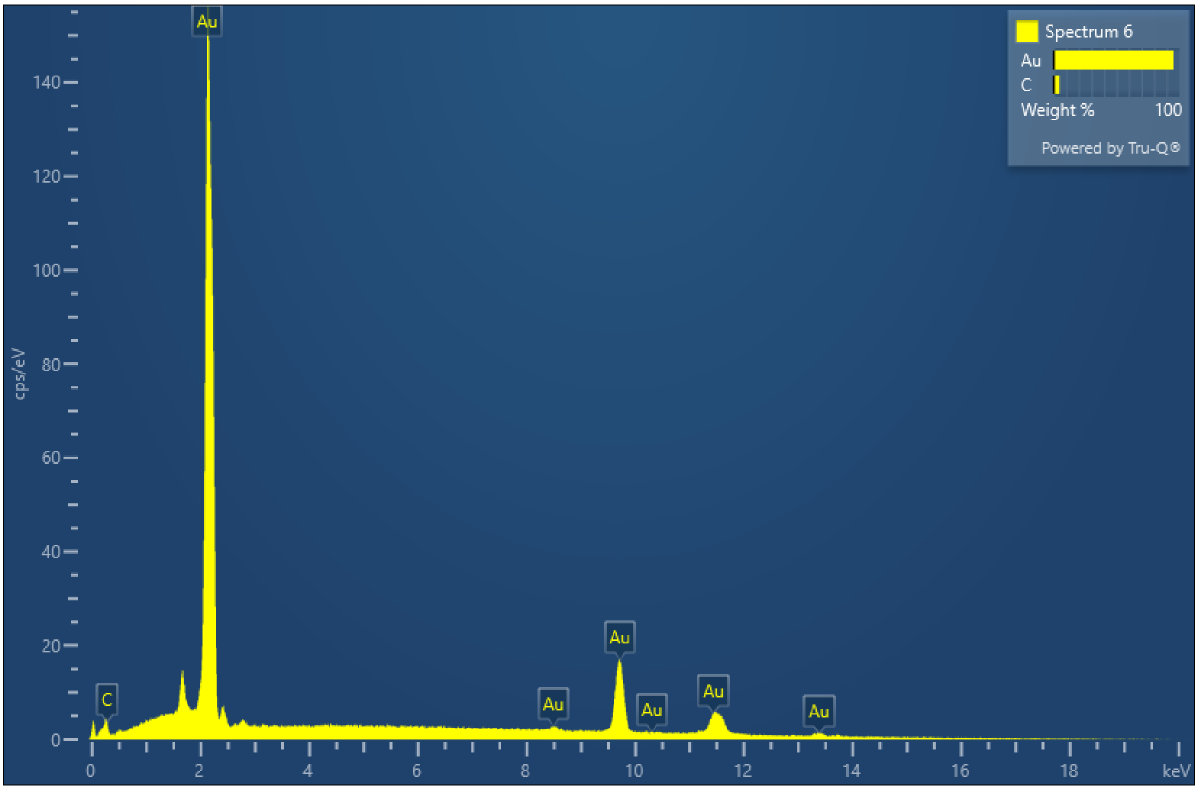

Figure S.3. 14 Spectrum 6 on Coin GLAHM:29540

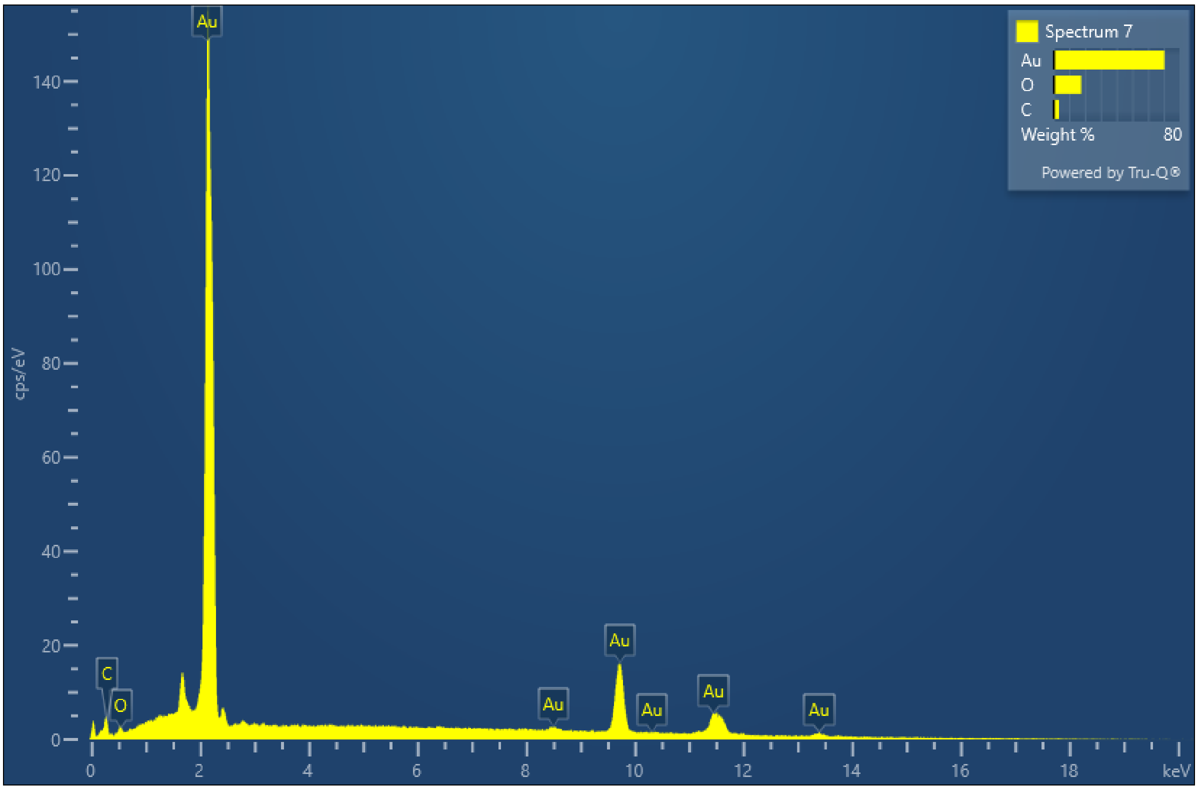

Figure S.3. 15 Spectrum 7 on Coin GLAHM:29540

Discussion: All spectra reveal multiple peaks for gold. No silver or copper was detected.

**GLAHM 29540**			
Spectrum Label	Au	Ag	Cu
Spectrum 1	100		
Spectrum 2	100		
Spectrum 3	100		
Spectrum 4	100		
Spectrum 5	100		
Spectrum 6	100		
Spectrum 7	100		
average	***100.00***		
standard dev P	***0.00***		
standard dev S	***0.00***		

Table S.3. 1 Summary of results for metal composition of GLAHM:29540

S.3.1.3 Small superficial patches

Analyses were made of four dark patches of similar appearance in SEM. Patches appear as this superficial deposits 3-8 µm in diameter overlying wear scratches (Spectra 17, 18, 20) or as infilling a crater ~10 µm in diameter.

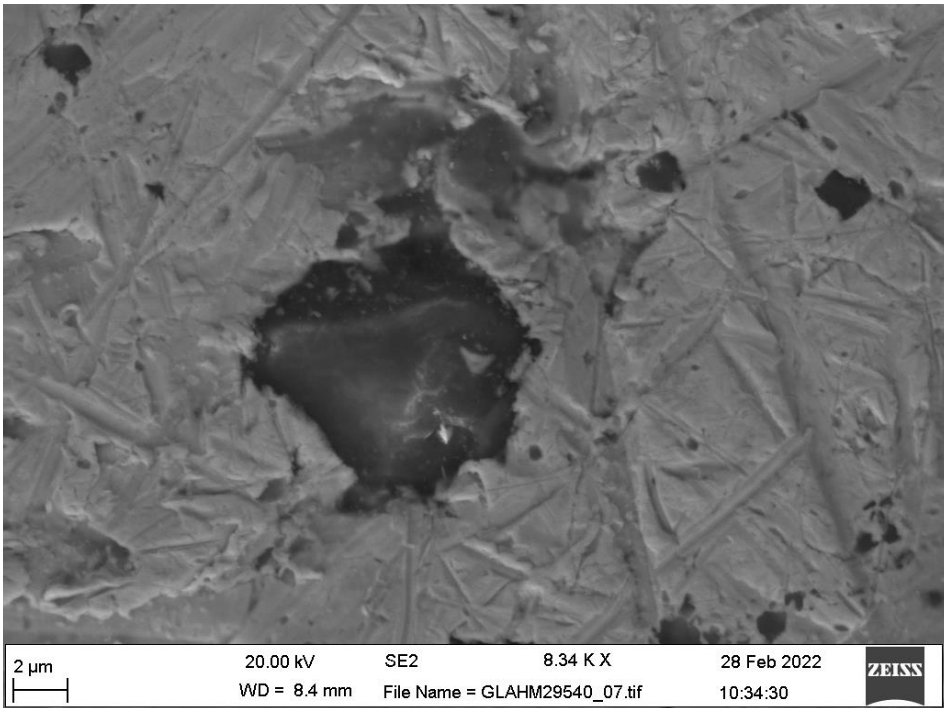

Figure S.3. 16 Area of Area of coin surface from the area of the emperor's head showing dark patches

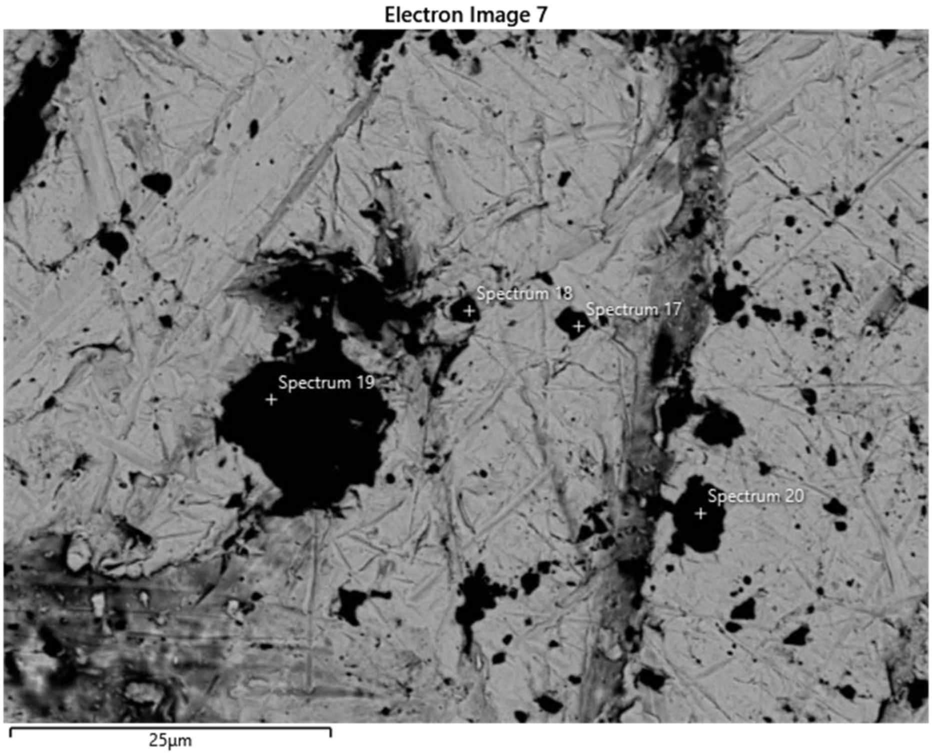

Figure S.3. 17 Sites of individual analyses

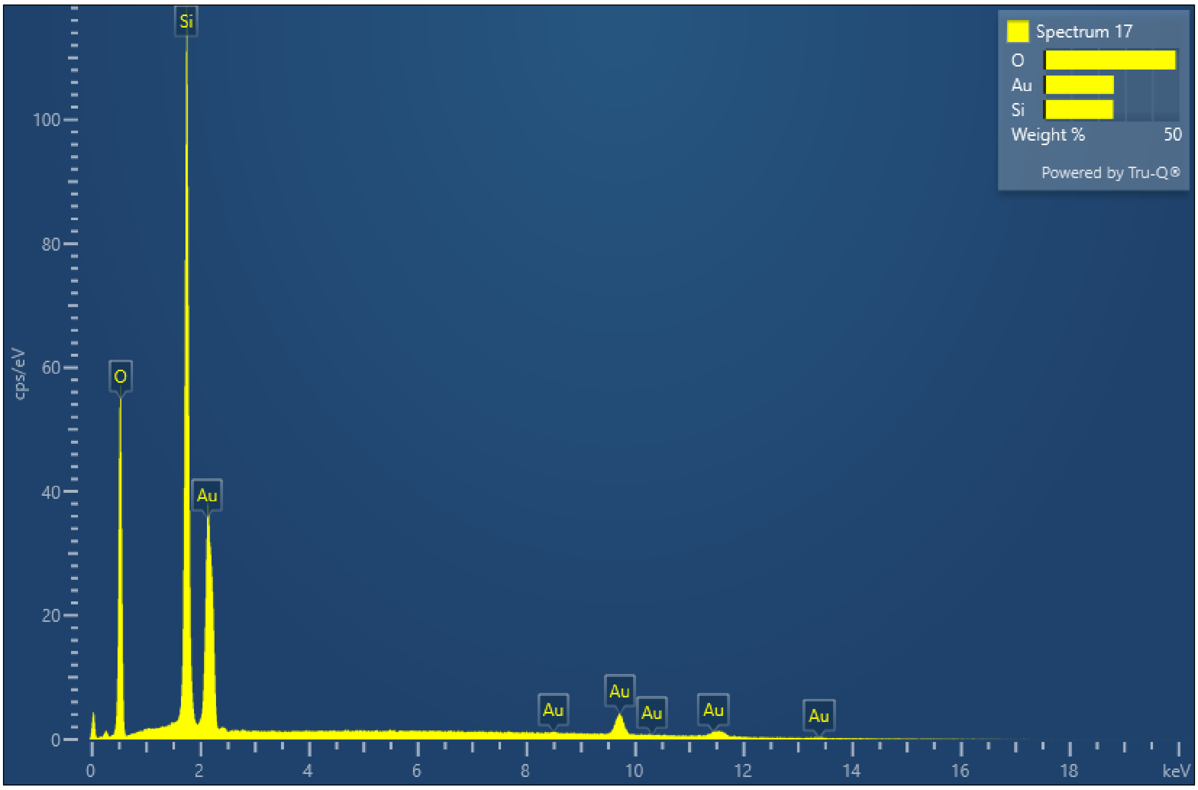

Figure S.3. 18 Spectrum 17 on Coin GLAHM:29540

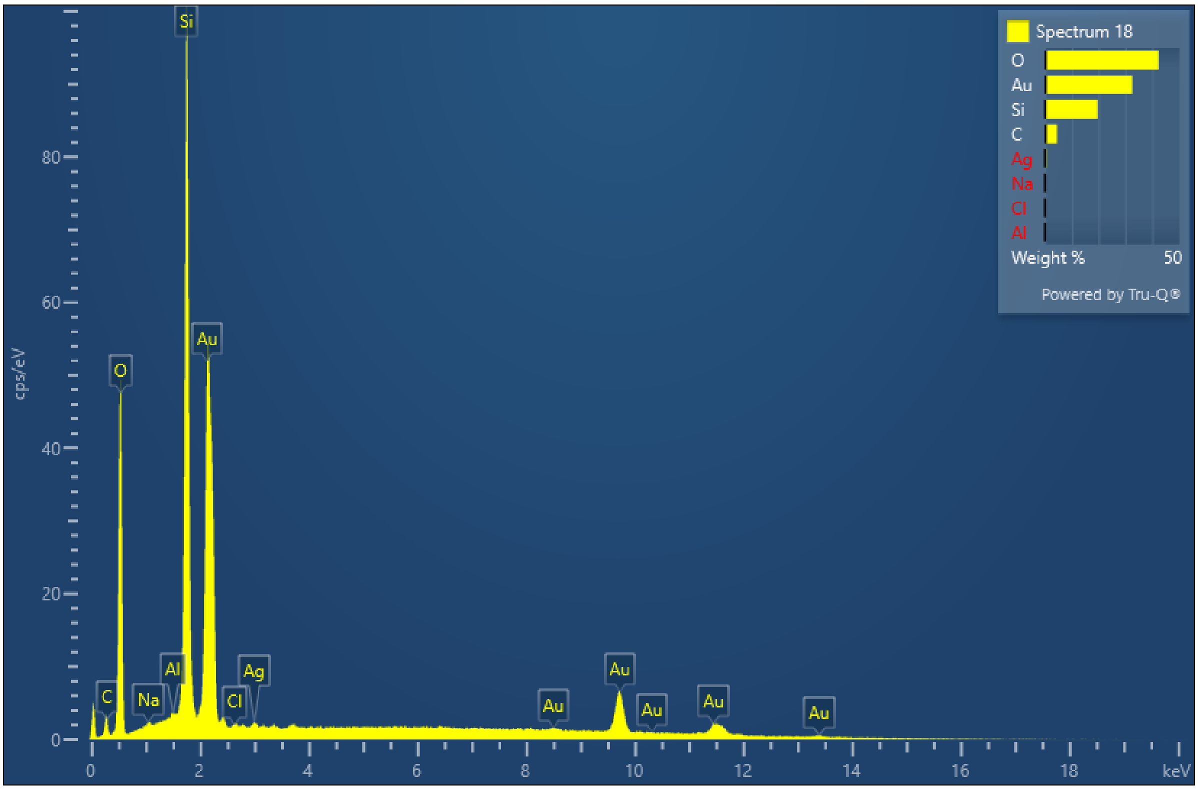

Figure S.3. 19 Spectrum 18 on Coin GLAHM:29540

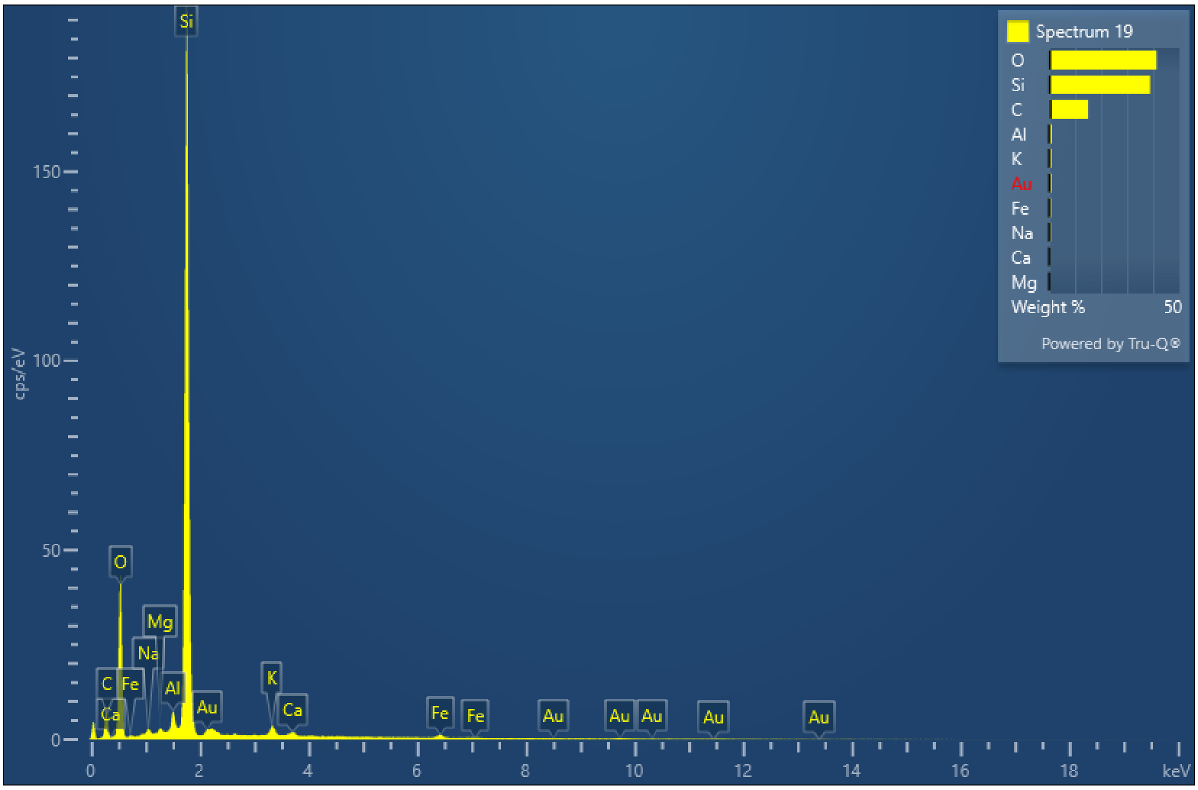

Figure S.3. 20 Spectrum 19 on Coin GLAHM:29540

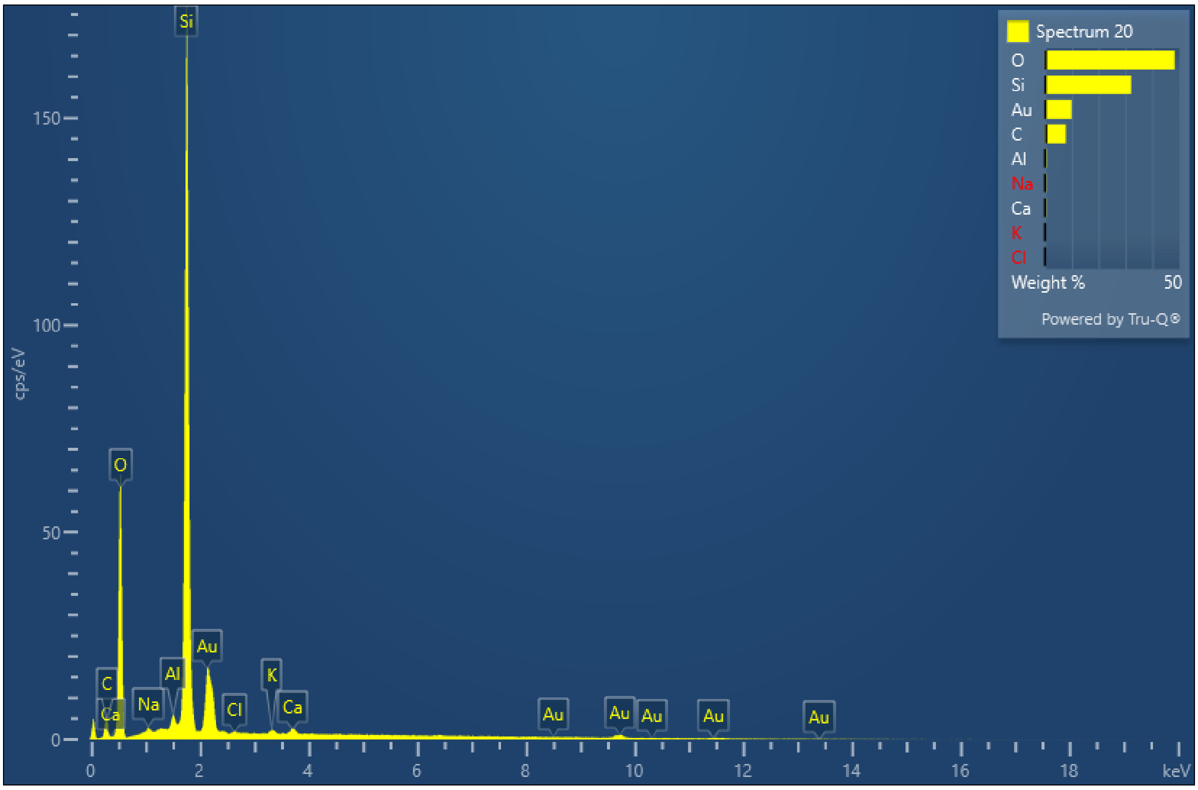

Figure S.3. 21 Spectrum 20 on Coin GLAHM:29540

Discussion: Spectrum 17 revealed the presence of Si and O with additional Au peaks from the underlying coin. Spectra 18, 19 and 20 were also dominated by Si and O. Minor peaks of

C, Al, K, Fe, Ca and Mg suggest small amounts of other substances, presumably included silicate minerals. The patches have no obvious crystalline morphology and are interpreted as amorphous opaline silica cementation spots.

S.3.1.4 Larger earthen deposits

Spectra 21 and 22 were taken within the earthen deposit nestling within the letter ‘O’ of the obverse legend (Figure S14 and S15). Note that Spectrum 21 was taken in the position that a significant patch of white matter prominent in Figure S14 had apparently detached prior to analysis revealing mostly bare metal underneath.

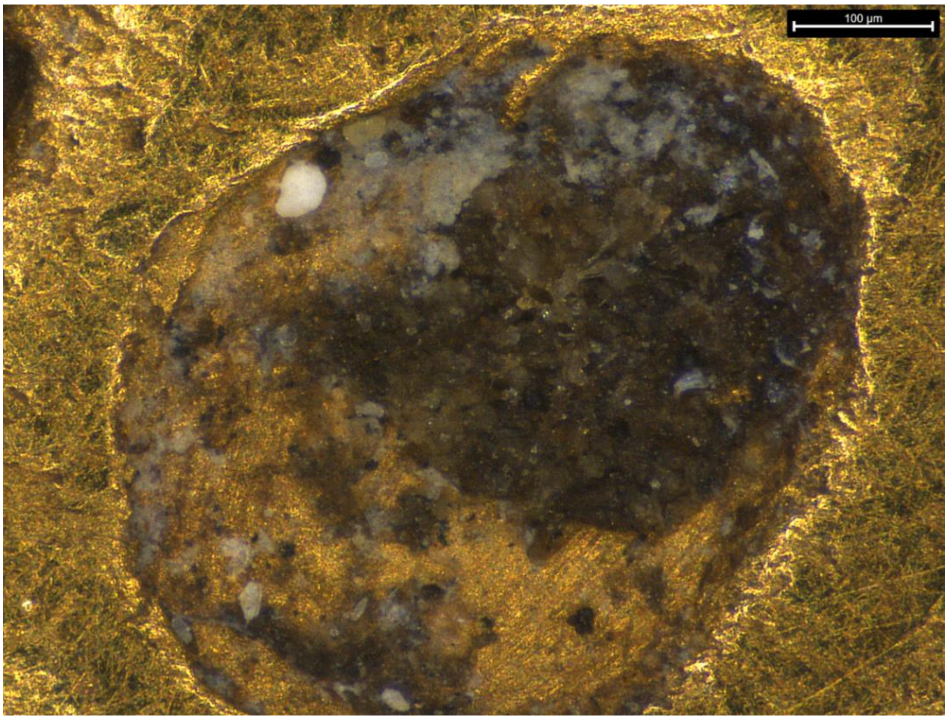

Figure S.3. 22 LM image of the area within the ‘O’ of the obverse legend

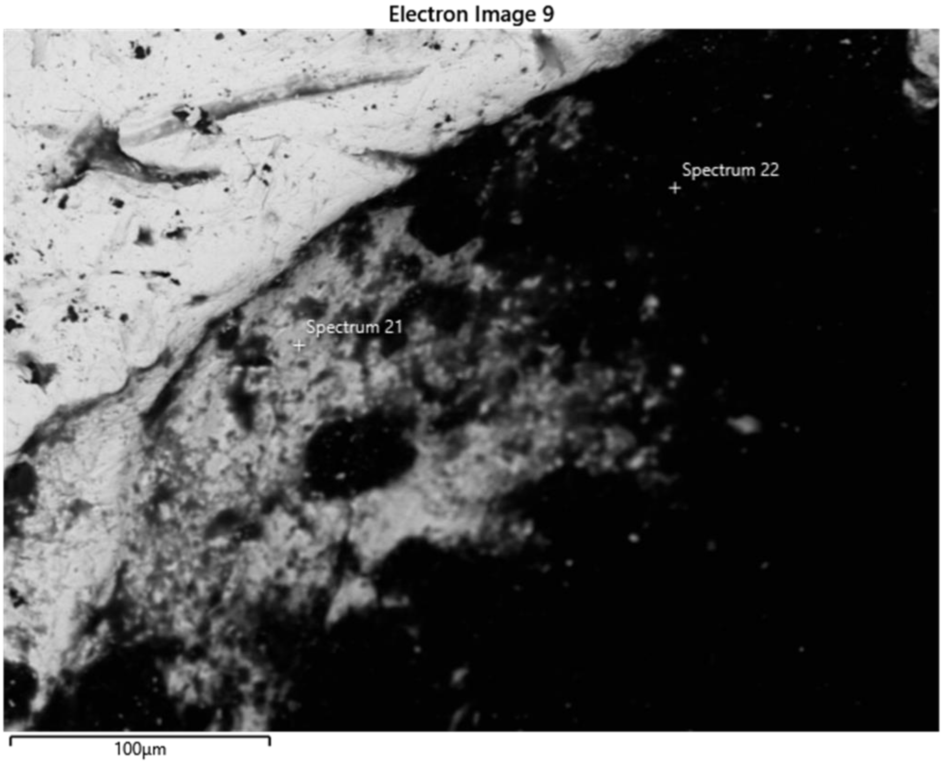

Figure S.3. 23 Area of coin surface showing area within the 'O' of the obverse legend where Spectra 21 and 22 were collected (corresponding to the upper part of Figure S13)

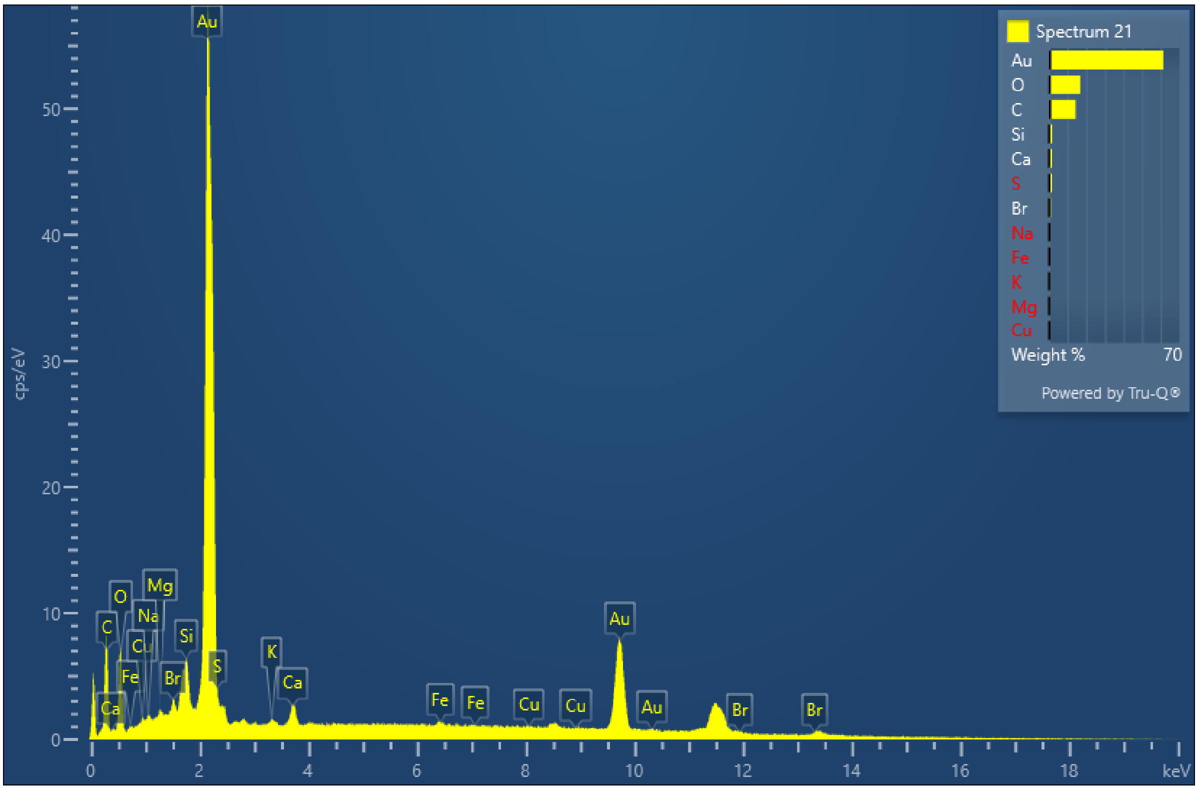

Figure S.3. 24 Spectrum 21 on Coin GLAHM:29540

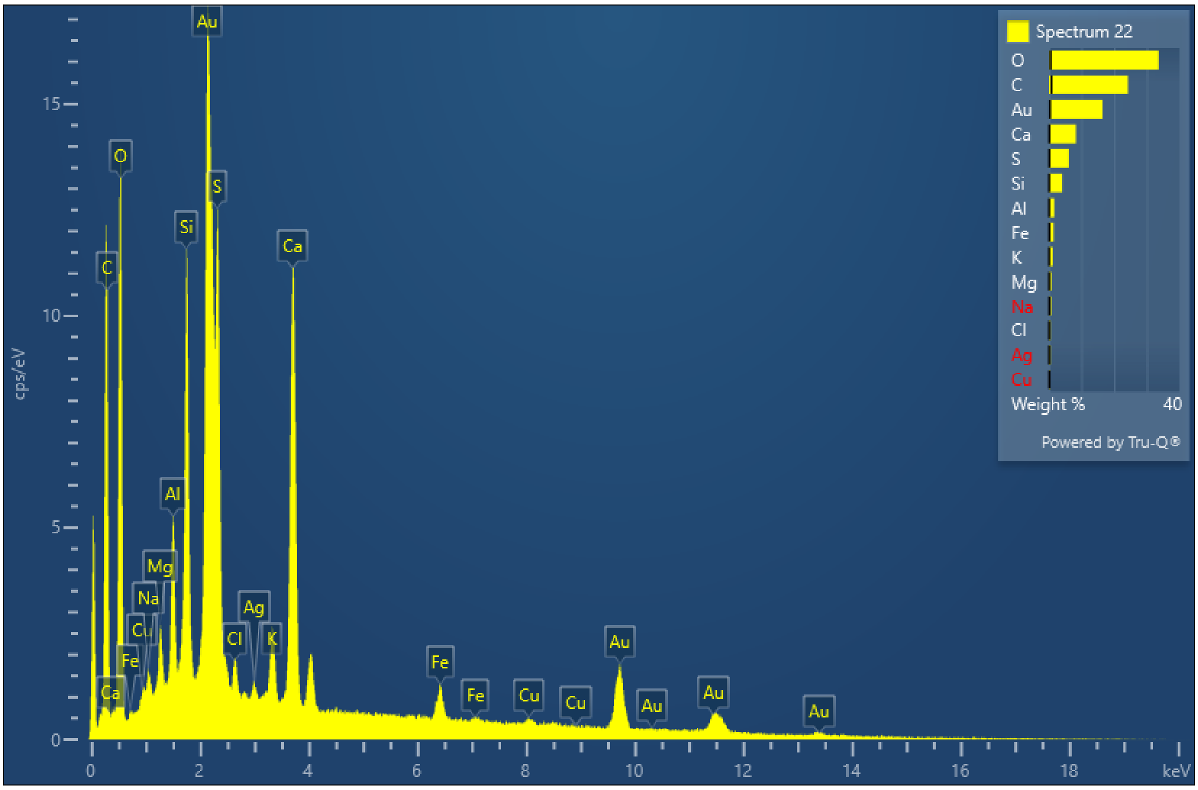

Figure S.3. 25 Spectrum 22 on Coin GLAHM:29540

Discussion: Spectrum 21 reveals mainly Au with other elements that are better observed in Spectrum 22. The C peak is interpreted as unreliable. Significant other peaks are Si, O, Ca, S and various cations. In combination with the LM and SEM imaging, and consistent with the r-FTIR results, the spectrum is interpreted as indicating gypsum ($CaSO_4.2H_2O$), silicate minerals and possibly calcite ($CaCO_3$).

S.3.2 Coin GLAHM:29697 (Genuine Philip I aureus)

S.3.2.1 Gallery of surface images

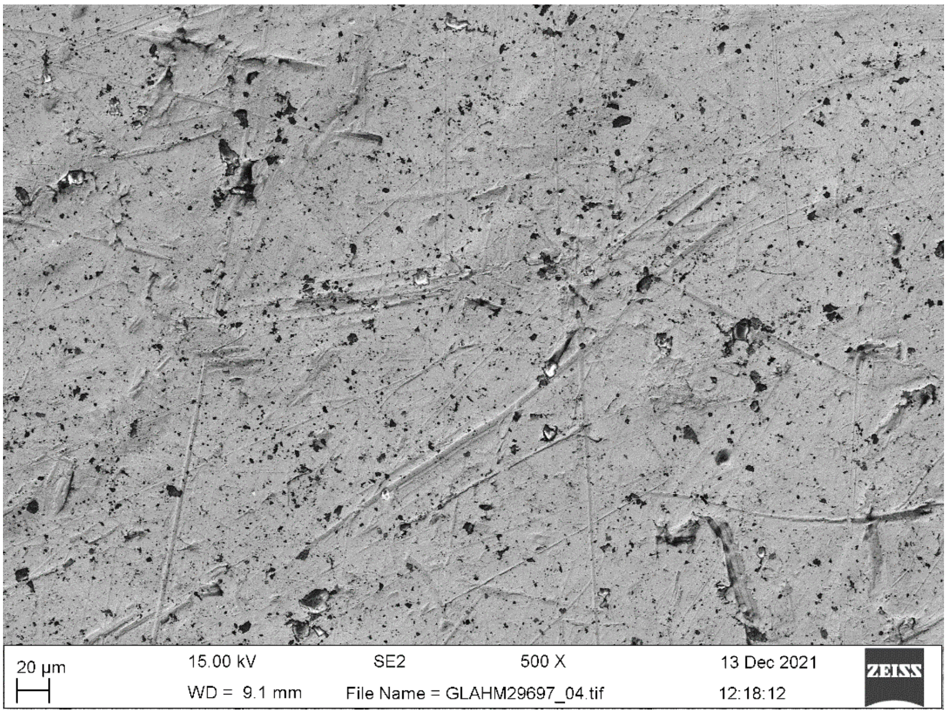

Figure S.3. 26 Area of emperor's head at 500x magnification

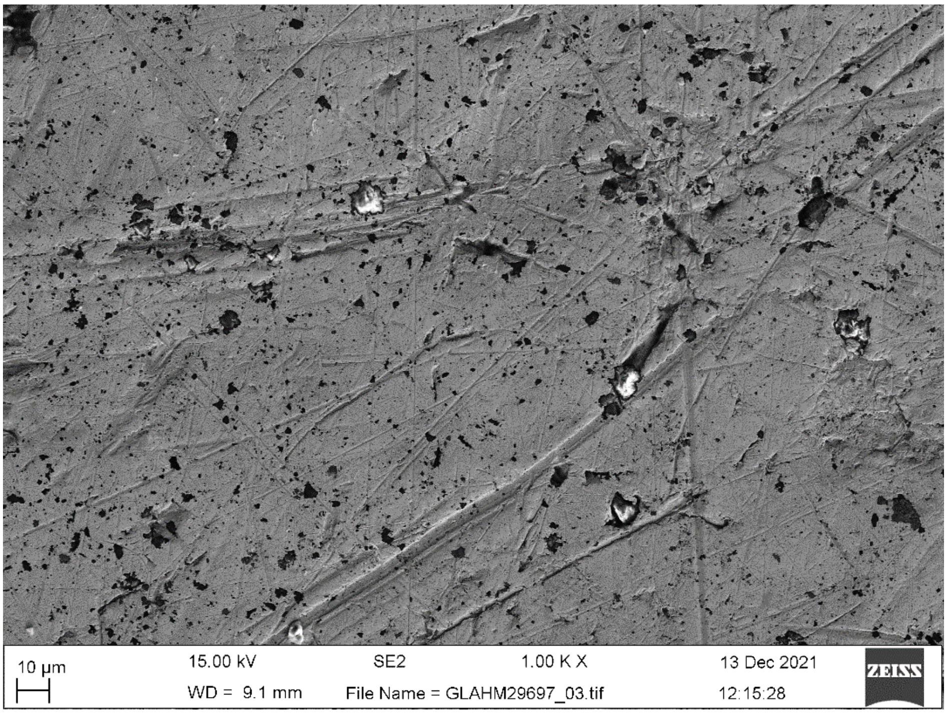

Figure S.3. 27 Area of emperor's head at 1000x magnification

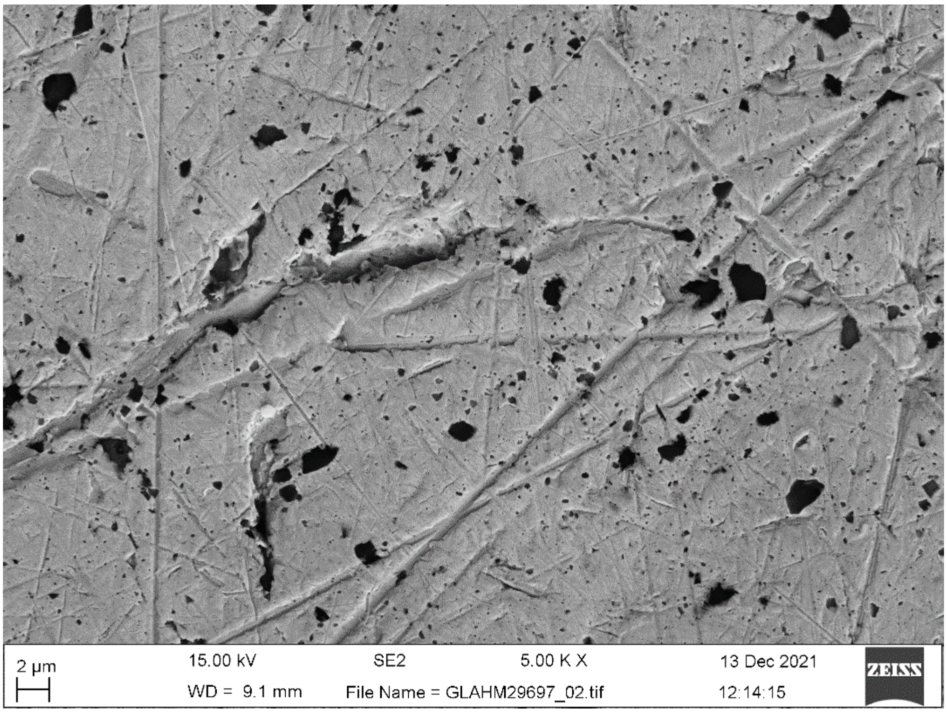

Figure S.3. 28 Area of emperor's head at 5000x magnification

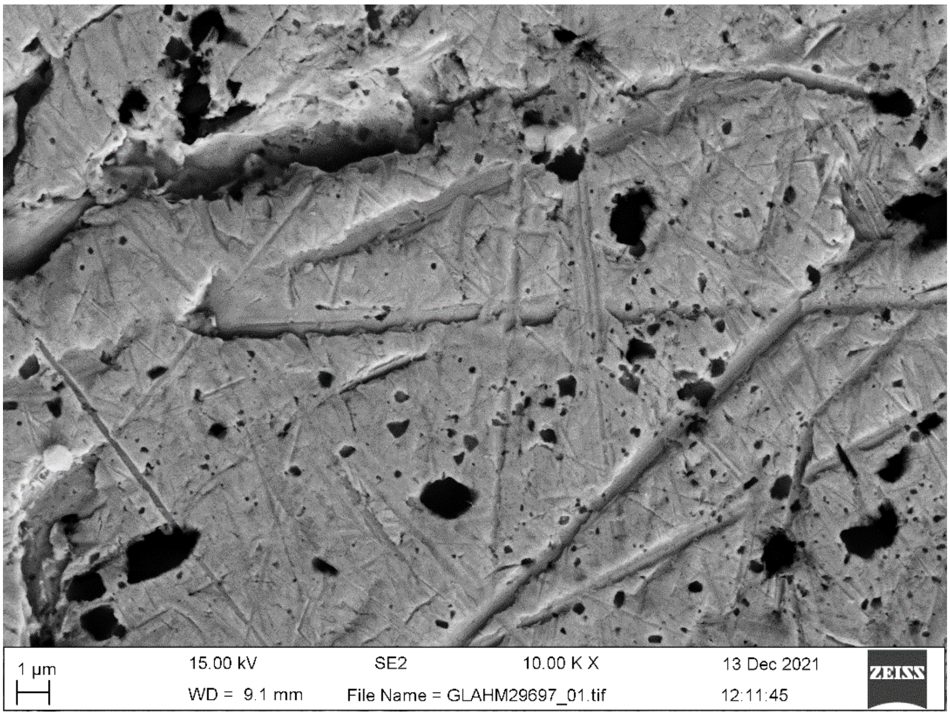

Figure S.3. 29 Area of emperor's head at 10000x magnification

S.3.3 Coin GLAHM:29596 (Questionable Gordian III medallion)

S.3.3.1 Gallery of surface images

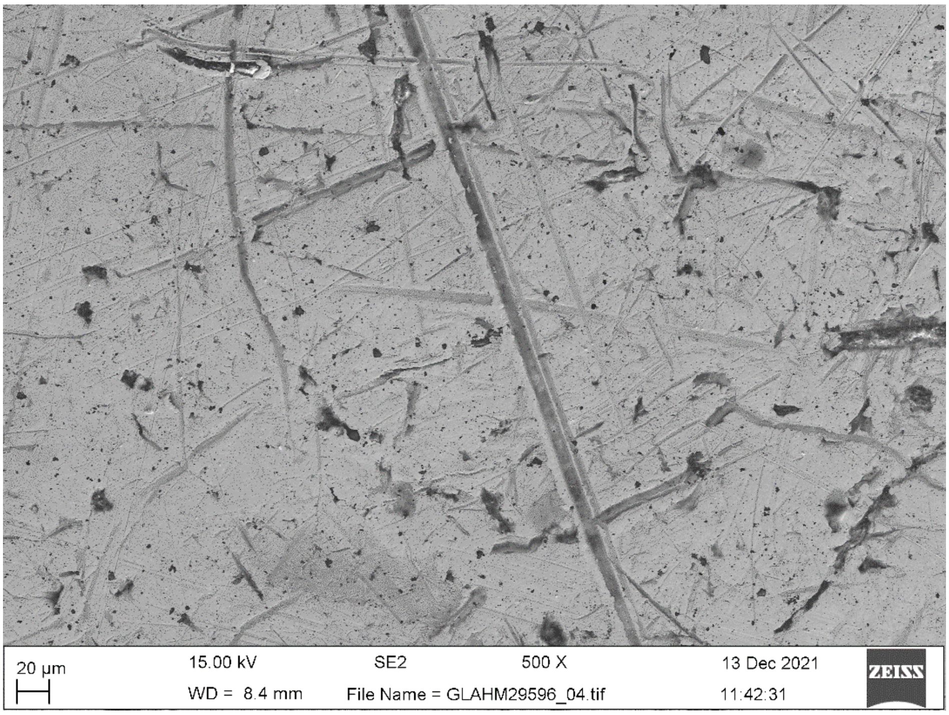

Figure S.3. 30 Area of emperor's head at 500 x magnification

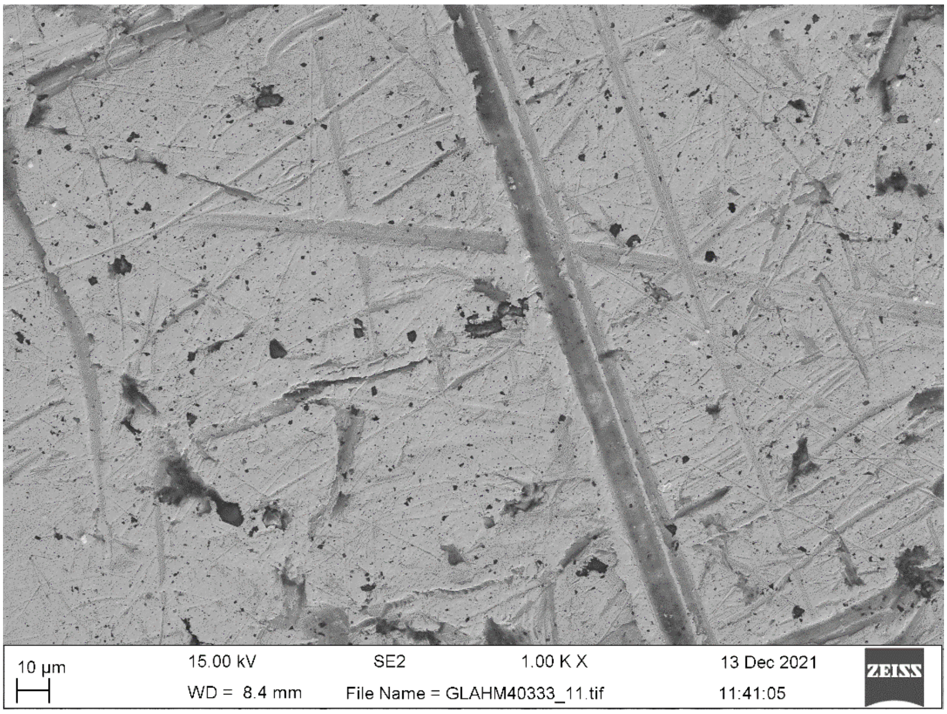

Figure S.3. 31 Area of emperor's head at 1000 x magnification

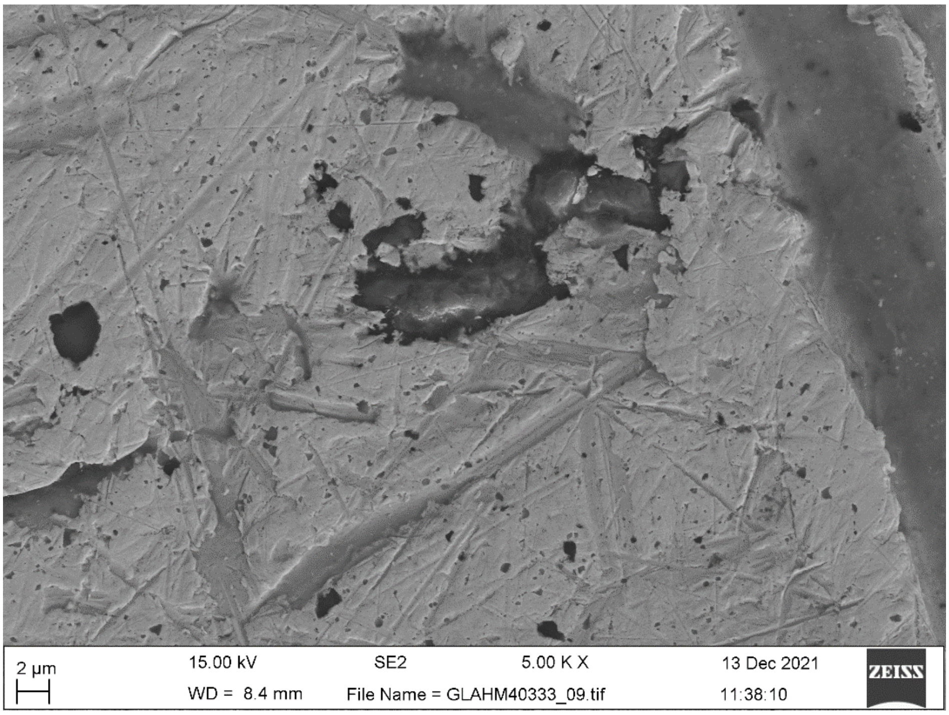

Figure S.3. 32 Area of emperor's head at 5000 x magnification

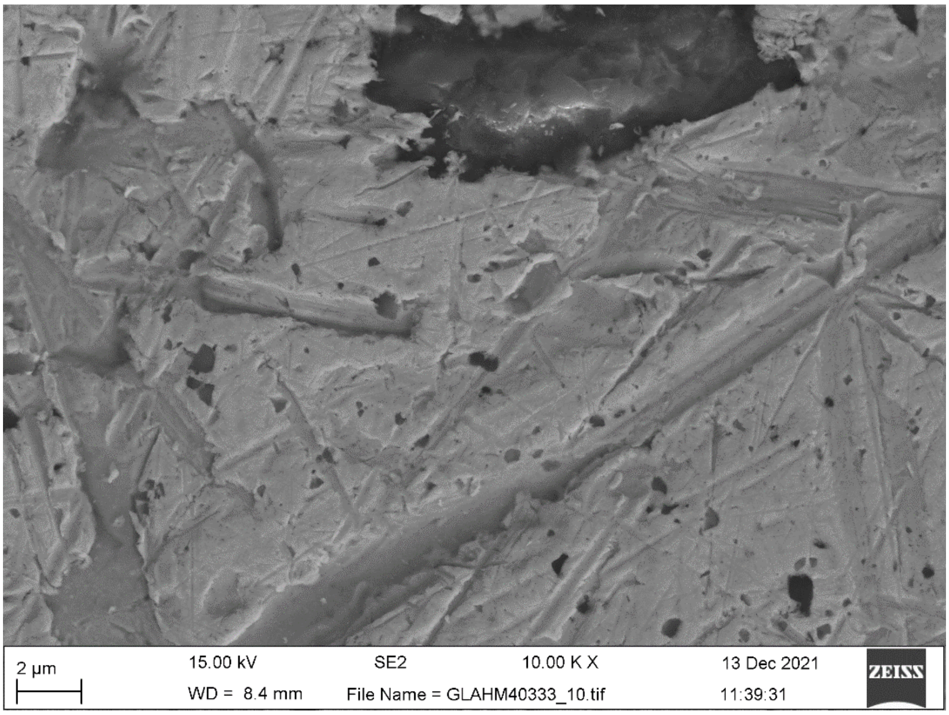

Figure S.3. 33 Area of emperor's head at 10000 x magnification

S.3.3.2 Metal composition

Analysis of two exposed and worn areas of the emperor's head (Figure S.3.18 and S.3.19) allowed us to establish the composition of the metal with Spectra 45, 46 and 47, 51, 52, 53 and 54. Other spots on the same images are discussed in the following section.

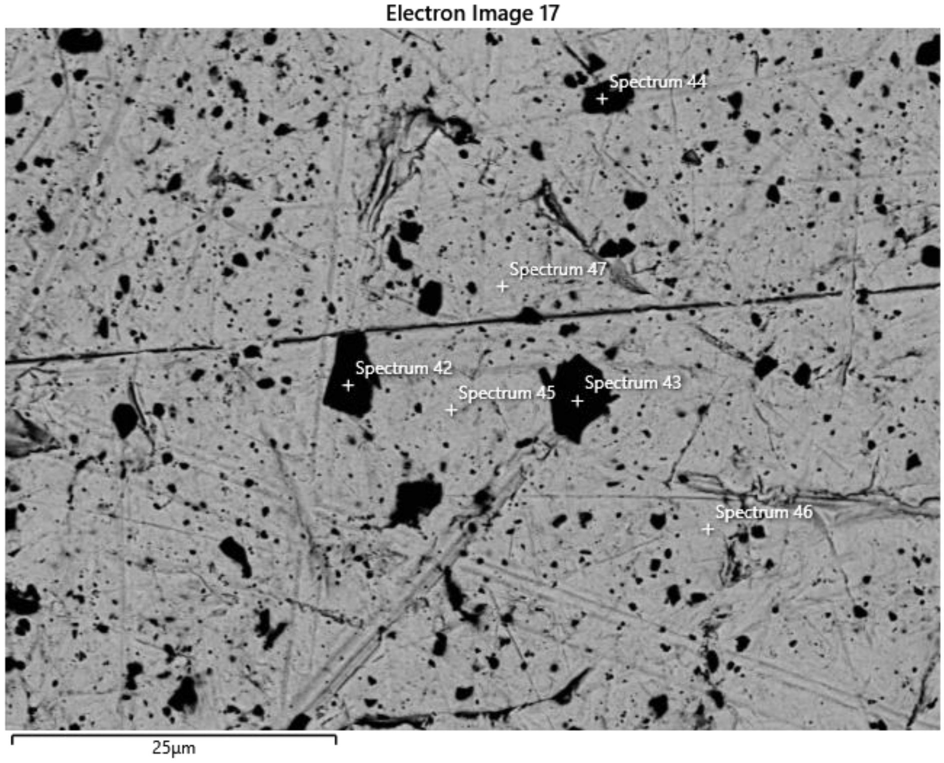

Figure S.3. 34 Exposed area of the emperor's head showing the location of Spectra 42-47

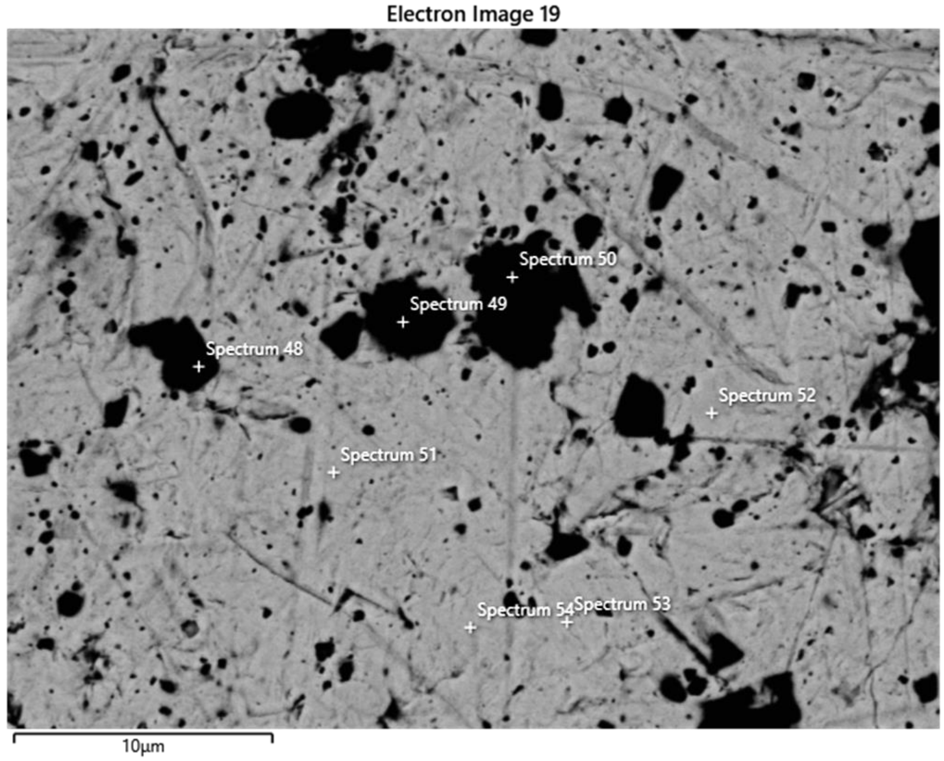

Figure S.3. 35 Exposed area of the emperor's head showing the location of Spectra 48-54

The spectra from metal analyses in these two areas are presented below:

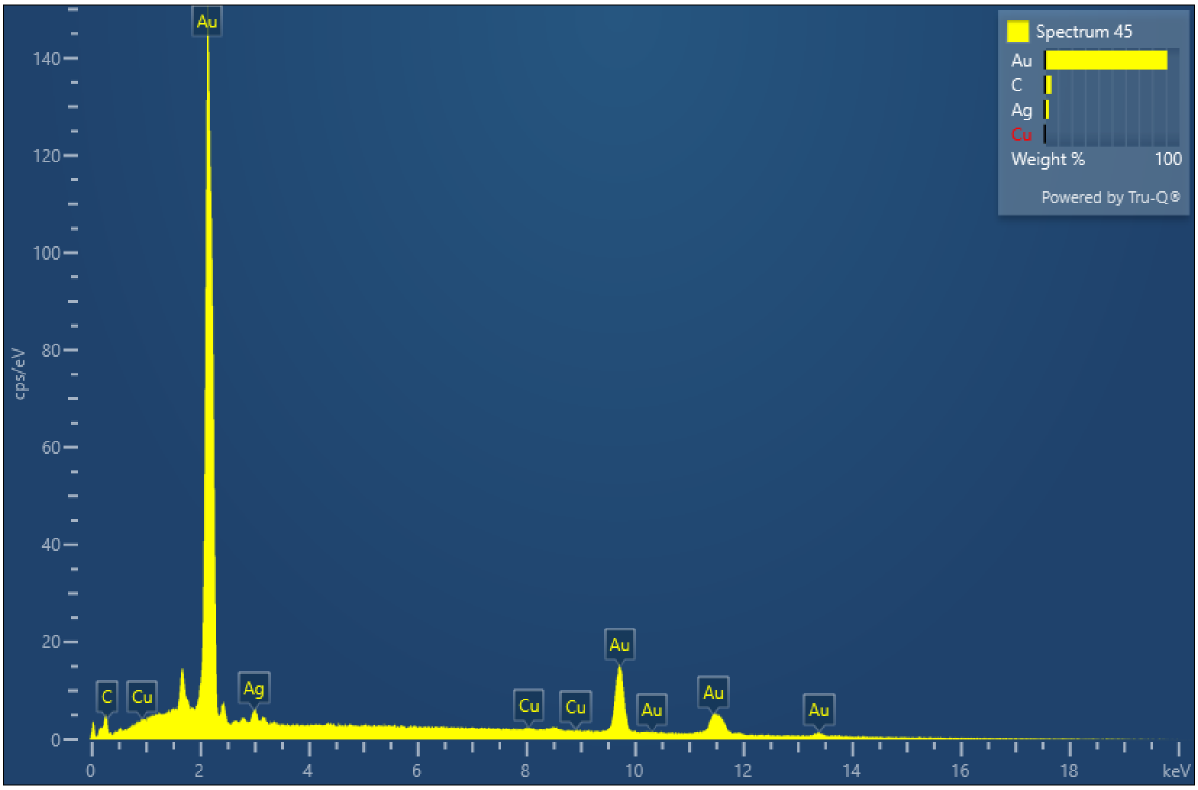

Figure S.3. 36 Spectrum 45 on Coin GLAHM:29596

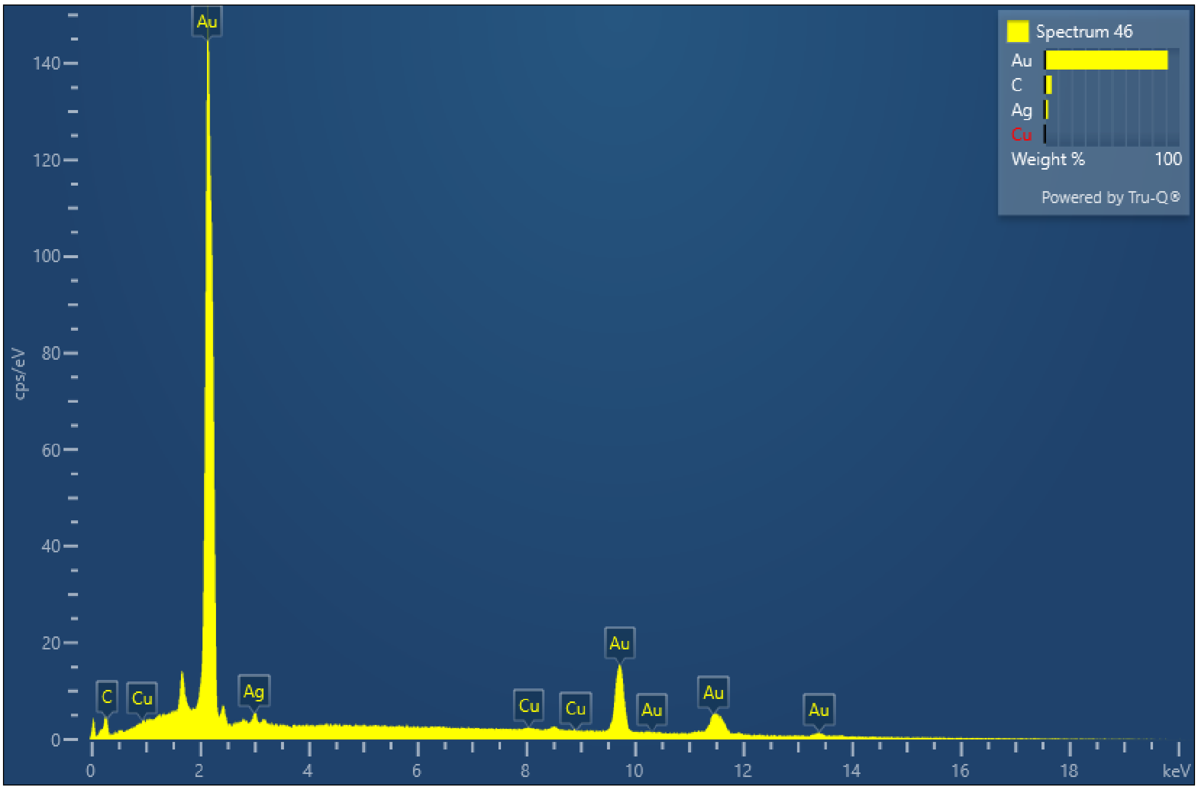

Figure S.3. 37 Spectrum 46 on Coin GLAHM:29596

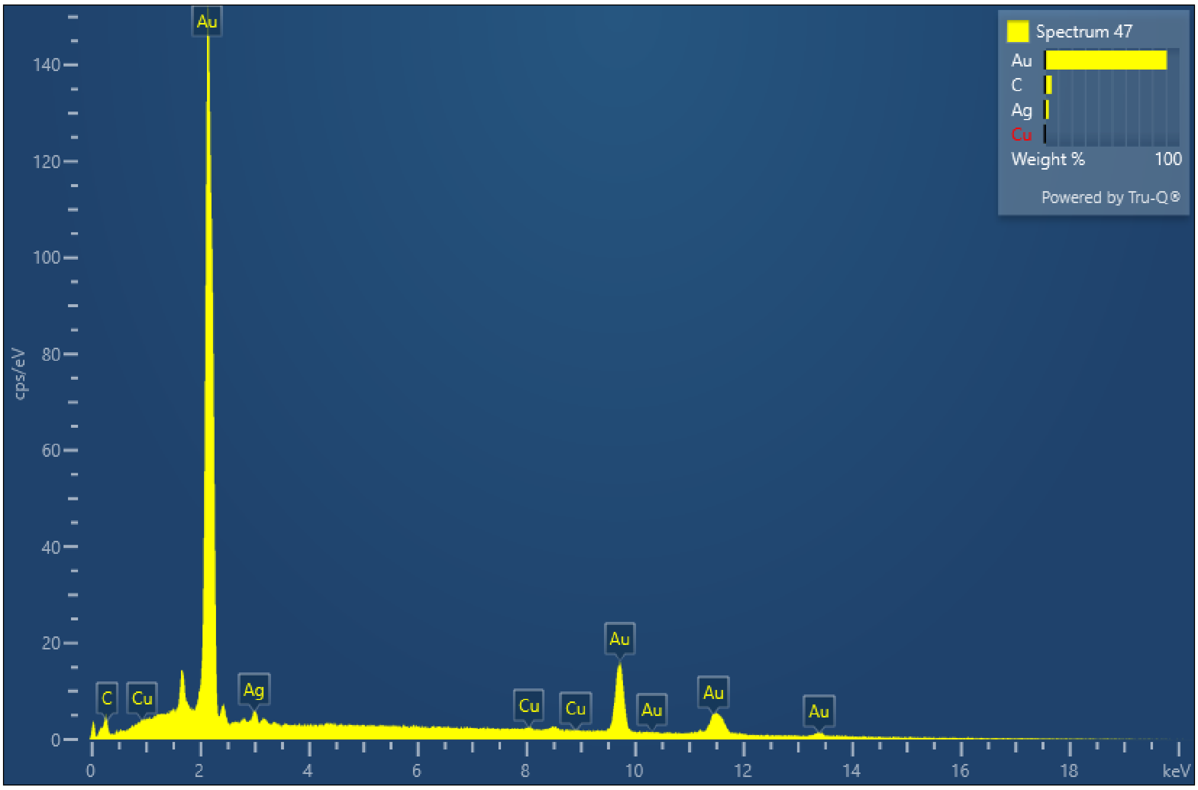

Figure S.3. 38 Spectrum 47 on Coin GLAHM:29596

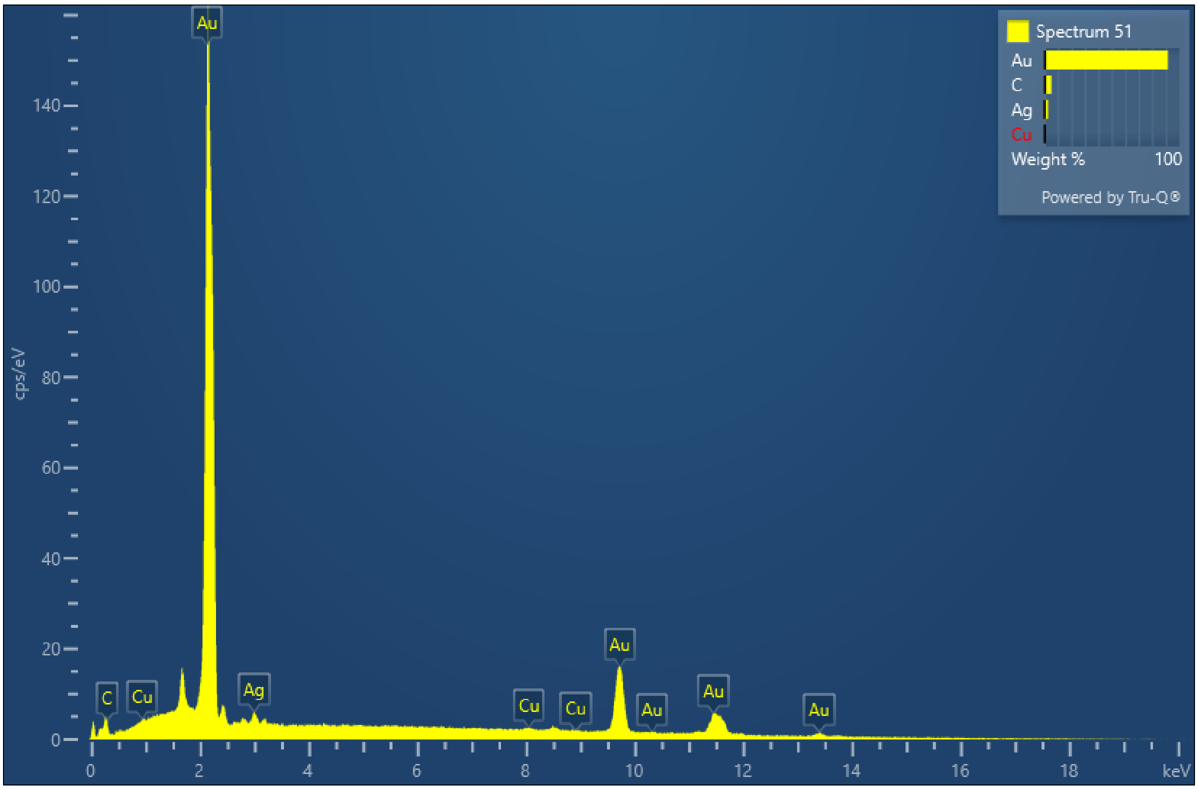

Figure S.3. 39 Spectrum 51 on Coin GLAHM:29596

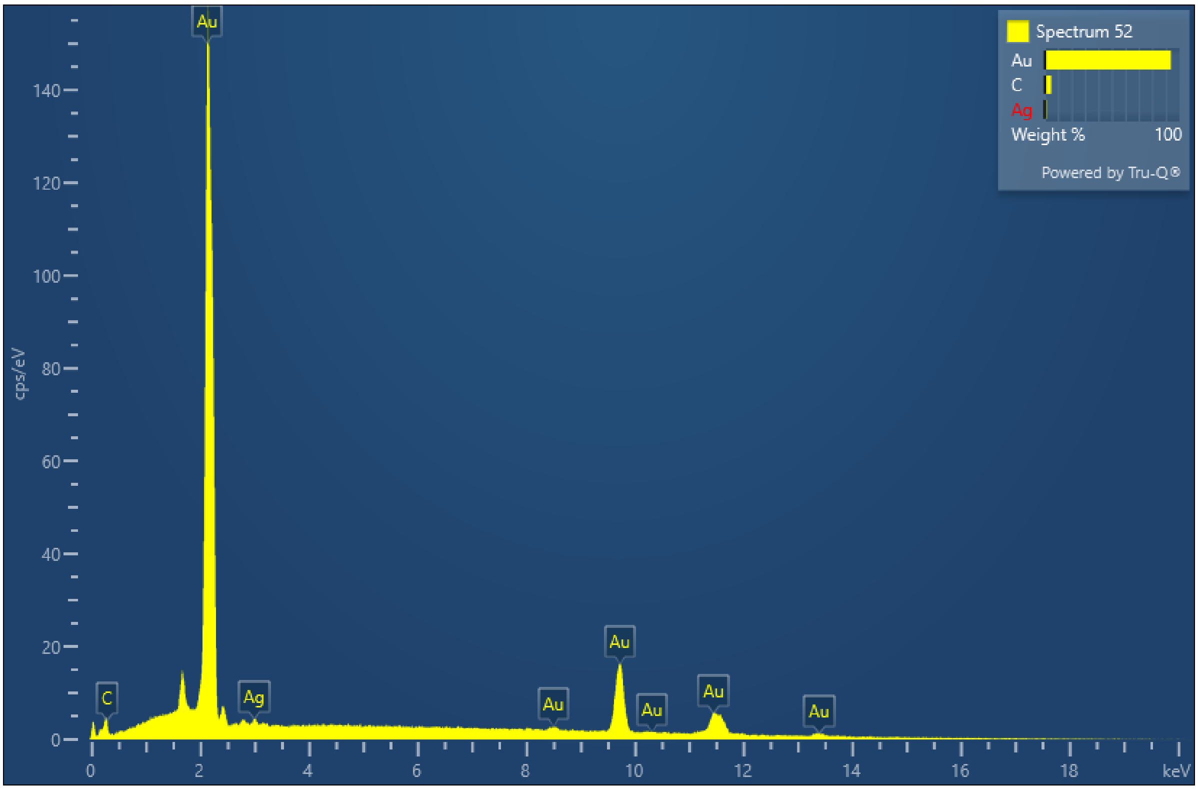

Figure S.3. 40 Spectrum 52 on Coin GLAHM:29596

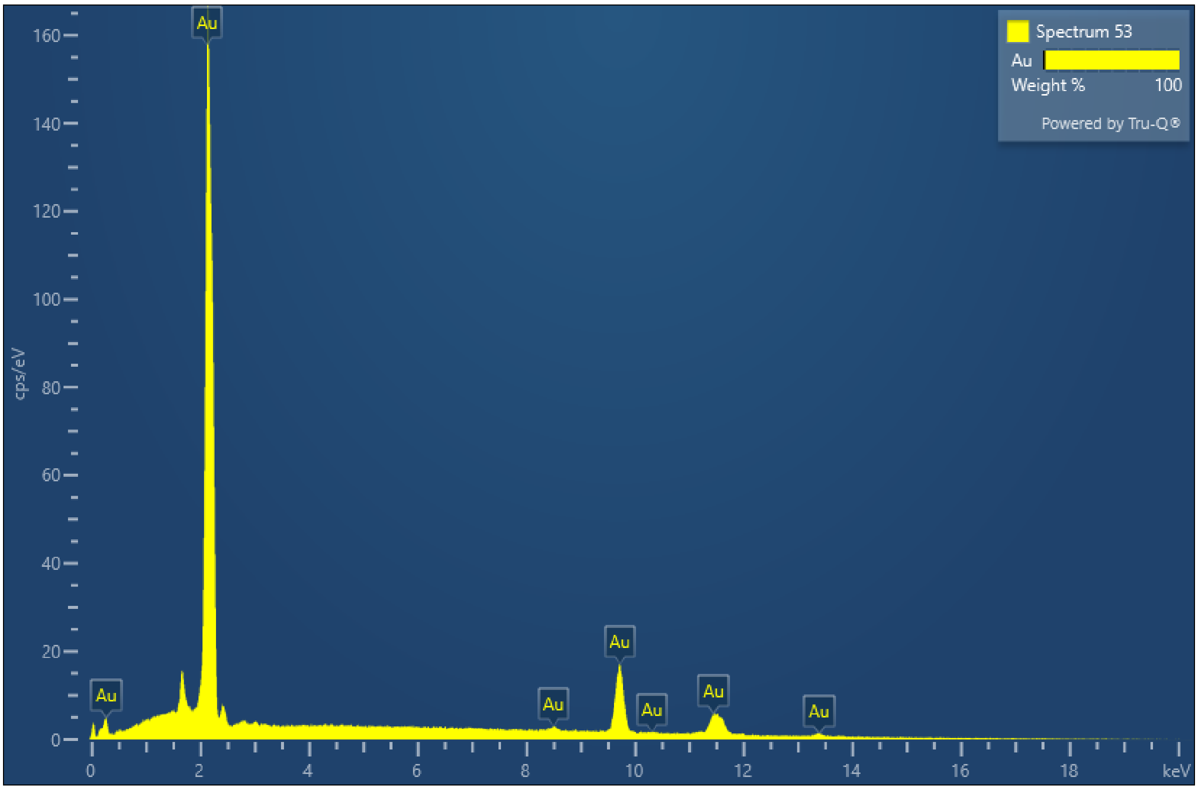

Figure S.3. 41 Spectrum 53 on Coin GLAHM:29596

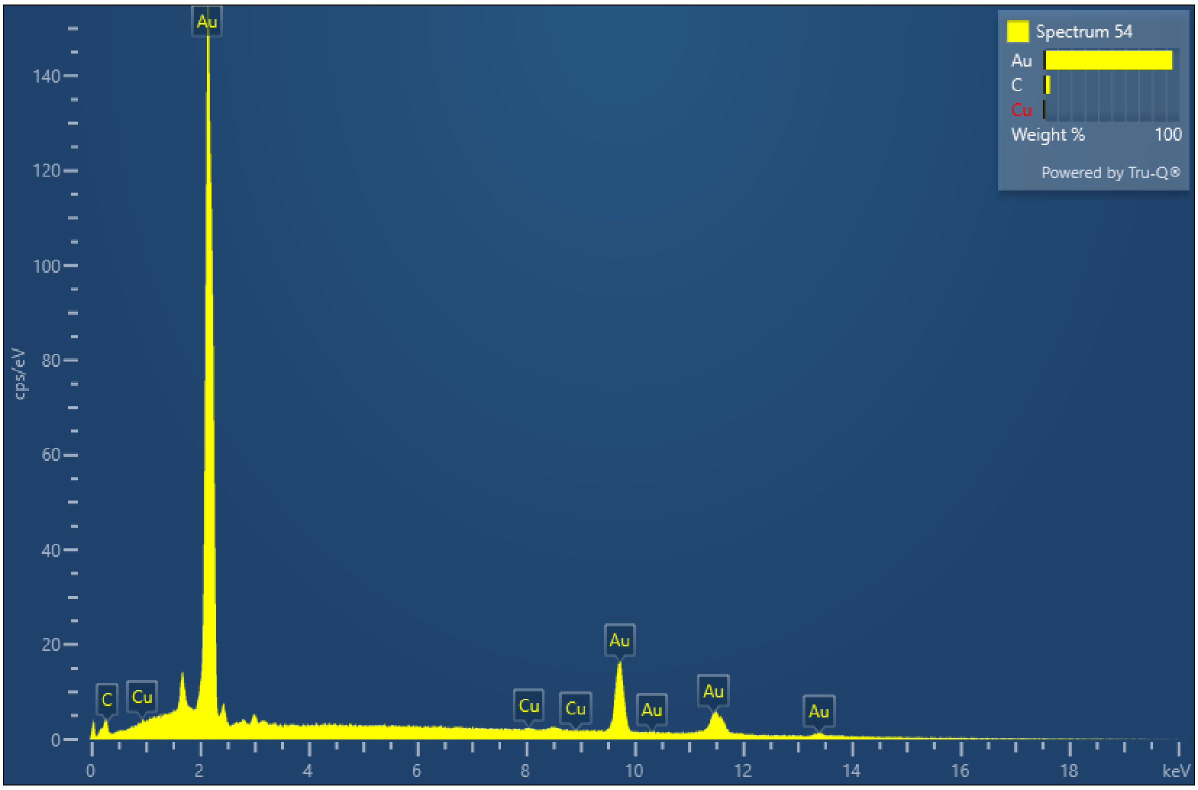

Figure S.3. 42 Spectrum 54 on Coin GLAHM:29596

Discussion: These six spectra are similar with multiple peaks associated with Au and subsidiary peaks related to Ag and Cu. The compositions of the six spectra are given in Table S2.

**GLAHM 29596**			
Spectrum Label	Au	Ag	Cu
Spectrum 45	95.62	3.8	0.59
Spectrum 46	96.3	3.06	0.64
Spectrum 47	95.51	3.62	0.87
Spectrum 51	96.15	3.1	0.75
Spectrum 52	98.34	1.66	0
Spectrum 54	96.55	2.7	0.75
average	***96.41***	***2.99***	***0.60***
standard dev P	***0.94***	***0.70***	***0.28***
standard dev S	1.03	0.76	0.31

Table S. 3.2 Analyses of metal on Coin GLAHM:29596

S.3.3.3 Small superficial patches

Spectra were obtained from five small irregular dark patches in two areas (see Figures S3.18 and S.3.19) that clearly overlie wear scratches. They are very similar in appearance to the cementation spots on the genuine Gordian aureus discussed in Section 3.1.2. The results are as follows:

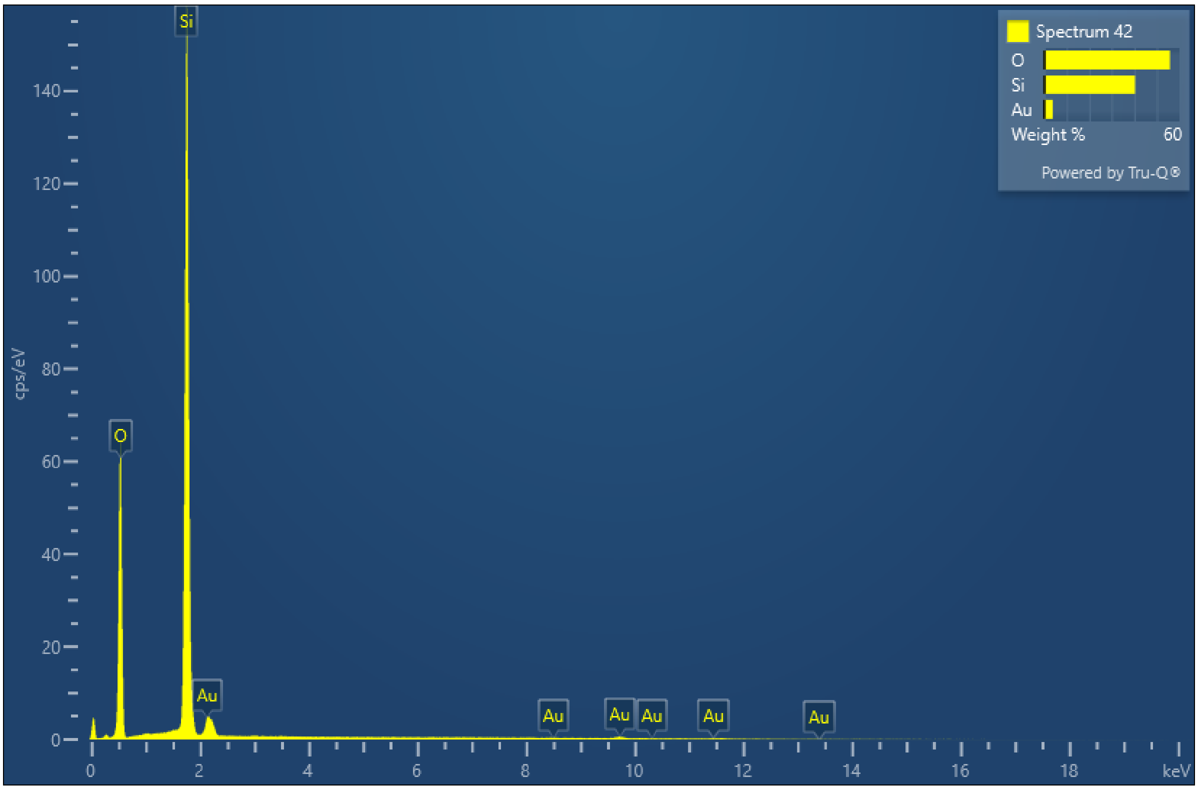

Figure S.3. 43 Spectrum 42 on Coin GLAHM:29596

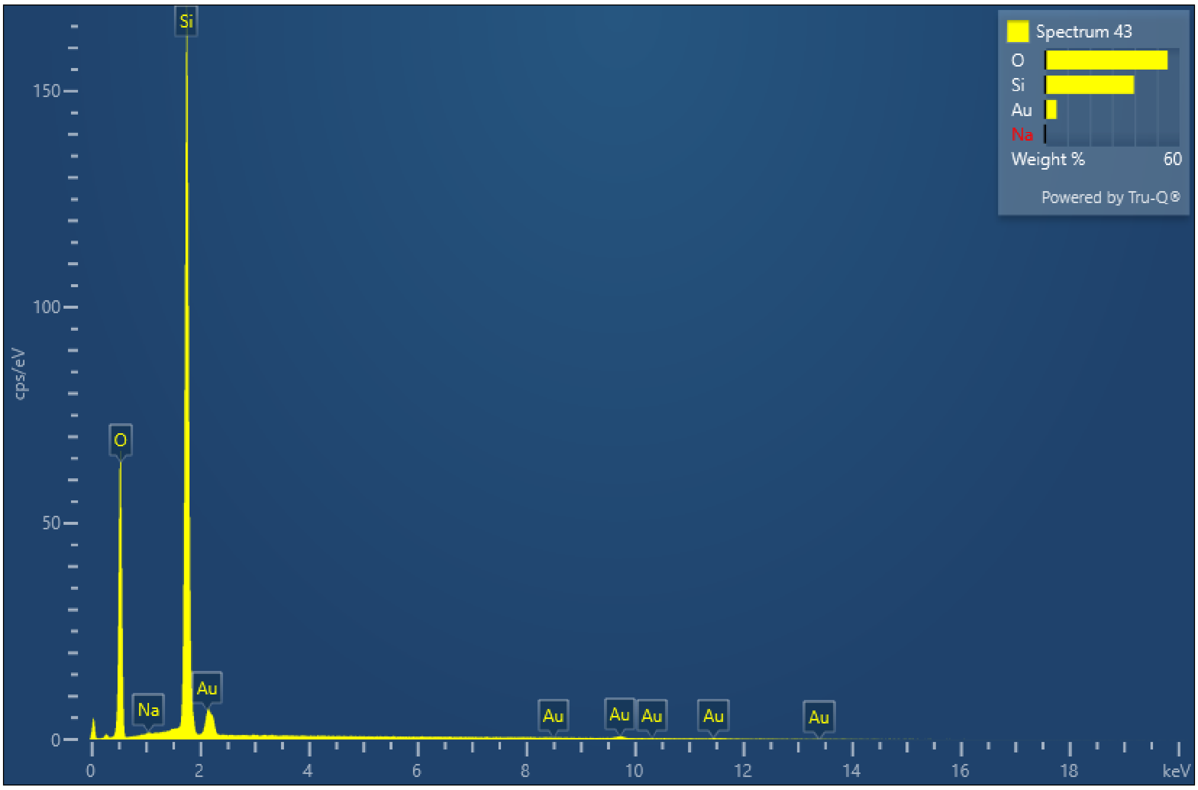

Figure S.3. 44 Spectrum 43 on Coin GLAHM:29596

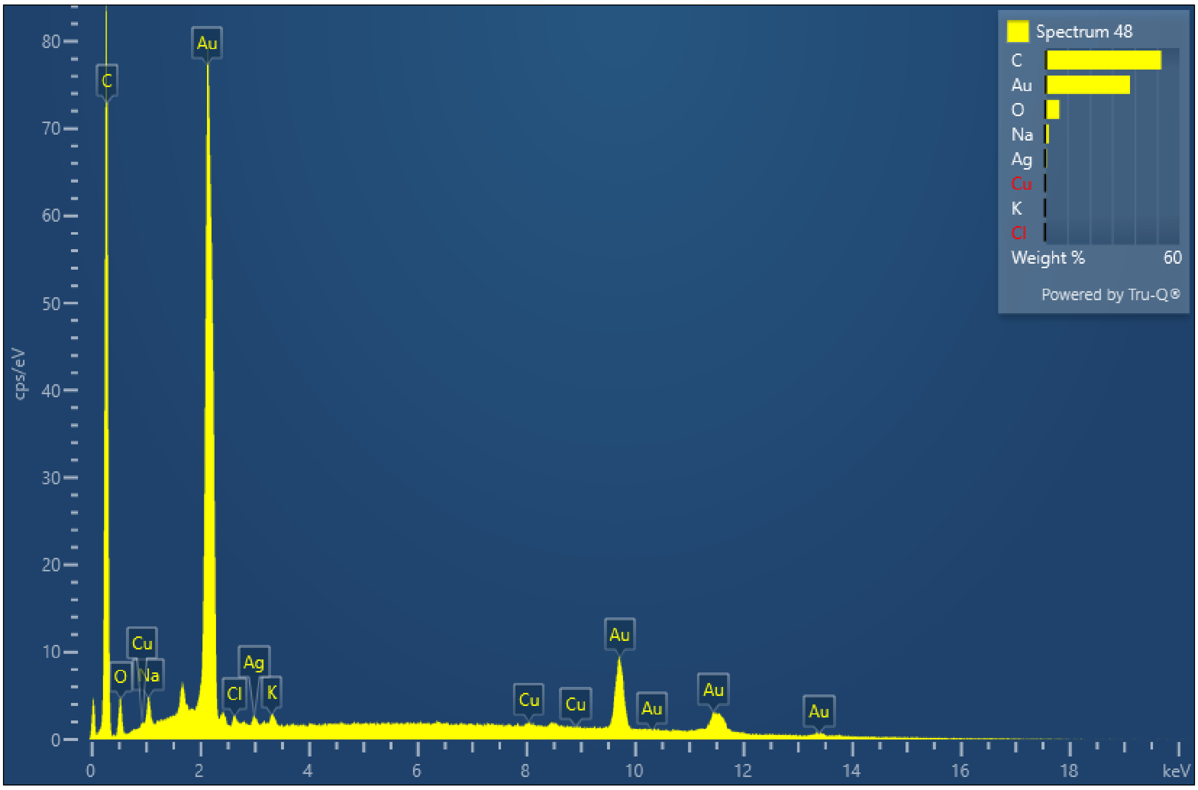

Figure S.3. 45 Spectrum 48 on Coin GLAHM:29596

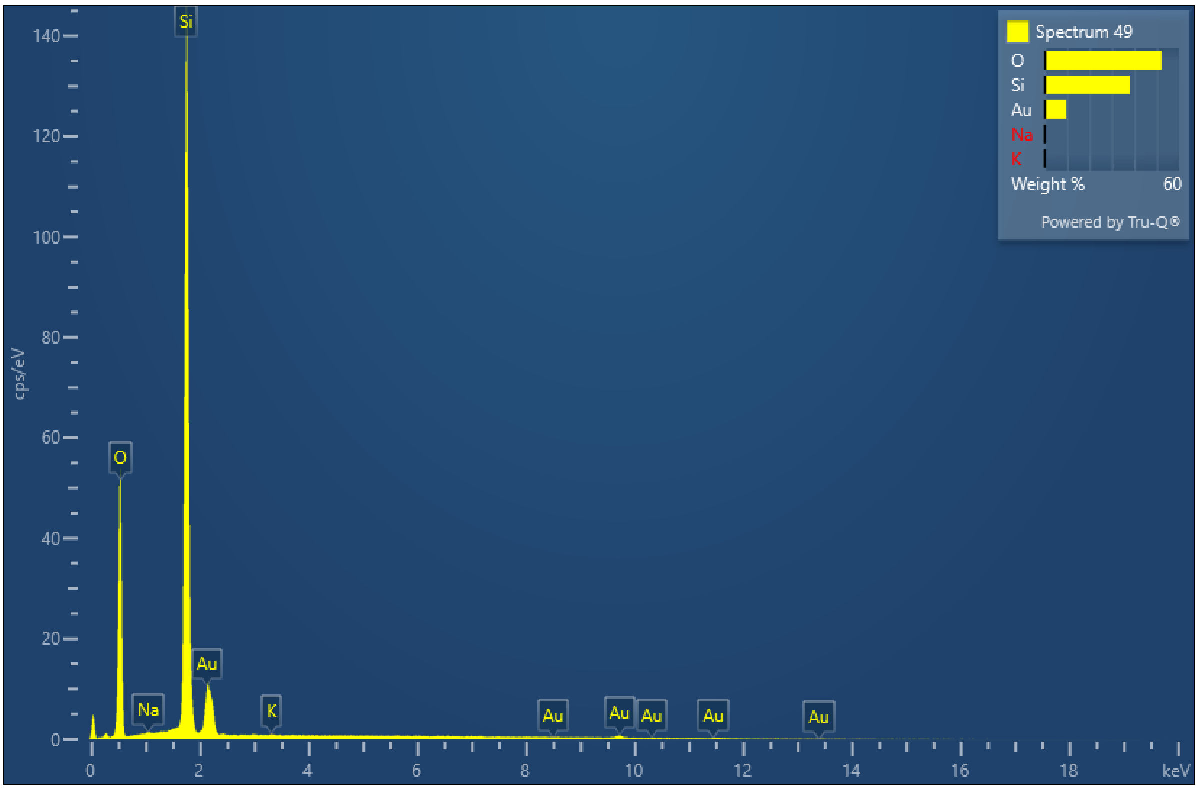

Figure S.3. 46 Spectrum 49 on Coin GLAHM:29596

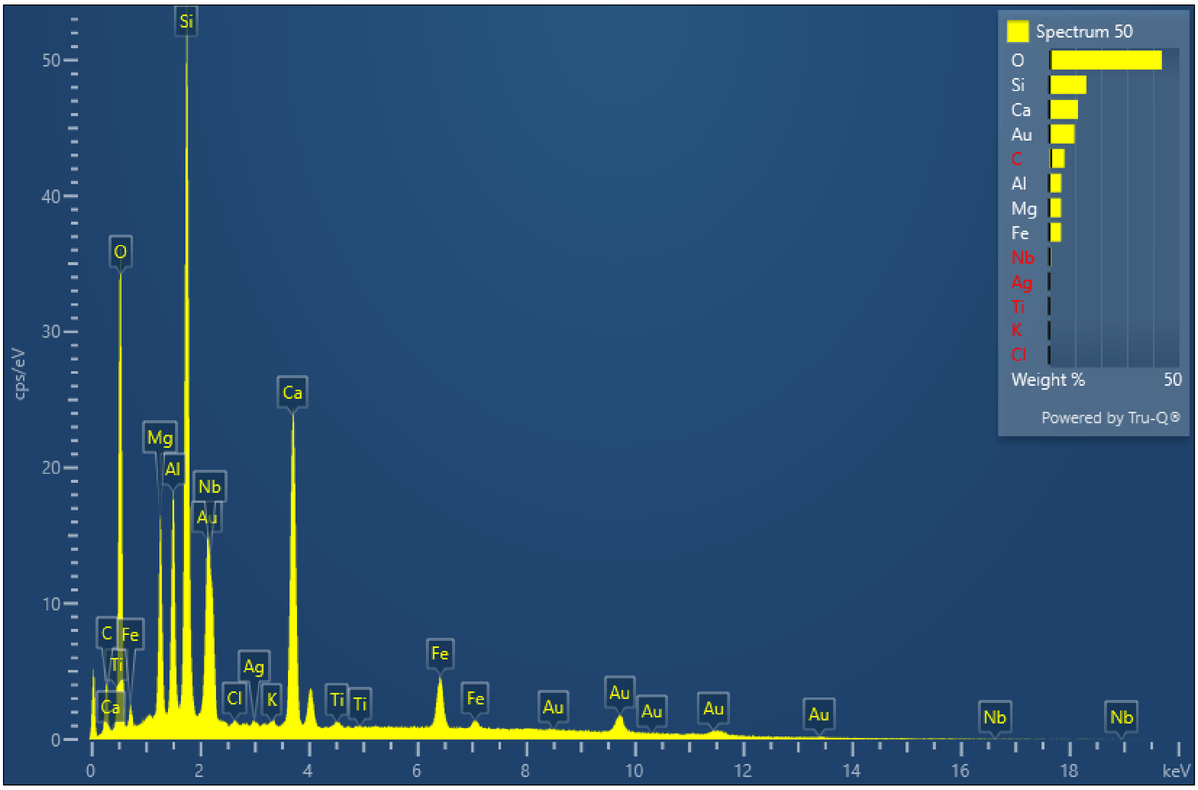

Figure S.3. 47 Spectrum 50 on Coin GLAHM:29596

Discussion: Aside from Au and Ag from the underlying coin, Spectra 42, 43 and 49 are dominated by peaks associated with Si and O, suggesting amorphous silica cement. Spectrum 50, the largest of the superficial patches, is more complex, showing significant peaks associated with Ca, Al, Mg and Fe. Spectrum 44 surprised us by being dominated by peaks associated with the underlying metal, presumably because the observed deposit was exceptionally thin at the point of analysis. In general these results indicate widespread nucleation of an amorphous silica cement, with the larger patches likely entraining other materials.

S.3.3.4 Larger earthen deposits

Observations were also made on a large area of apparent earthen deposit beside the letter 'S' of the obverse legend (Figures S32 and S33).

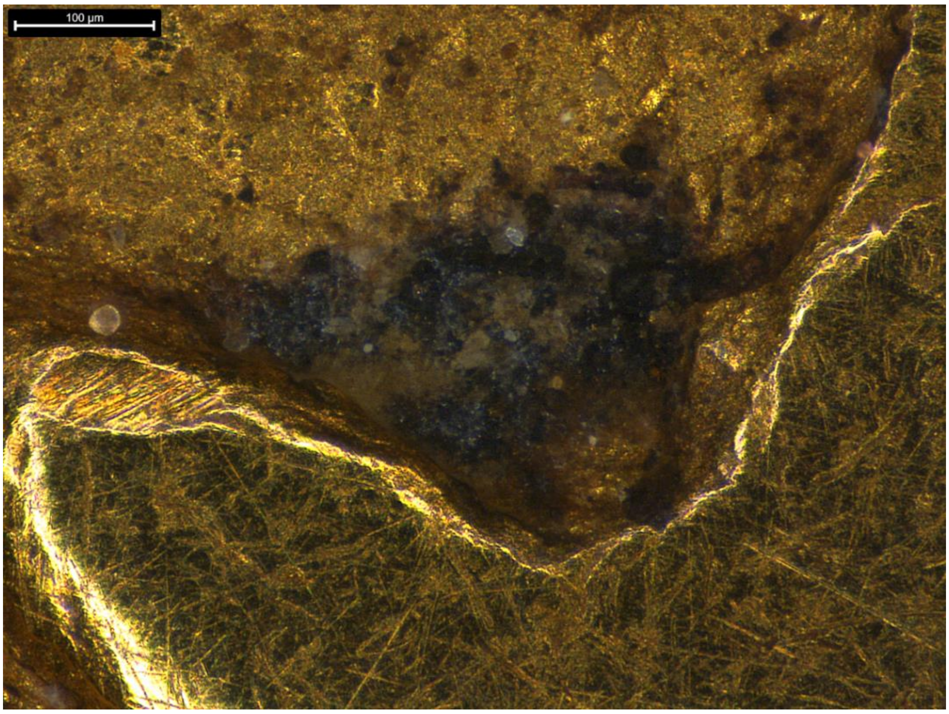

Figure S.3. 48 LM photograph of the area investigated

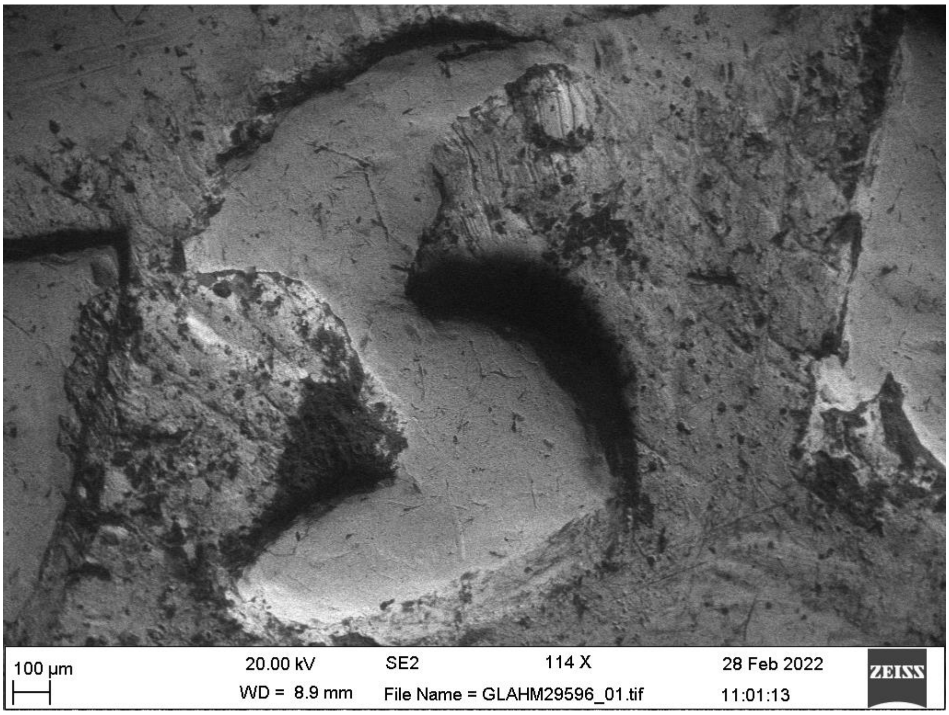

Figure S.3. 49 Figure Area of the obverse letter 'S' and its earthen deposits

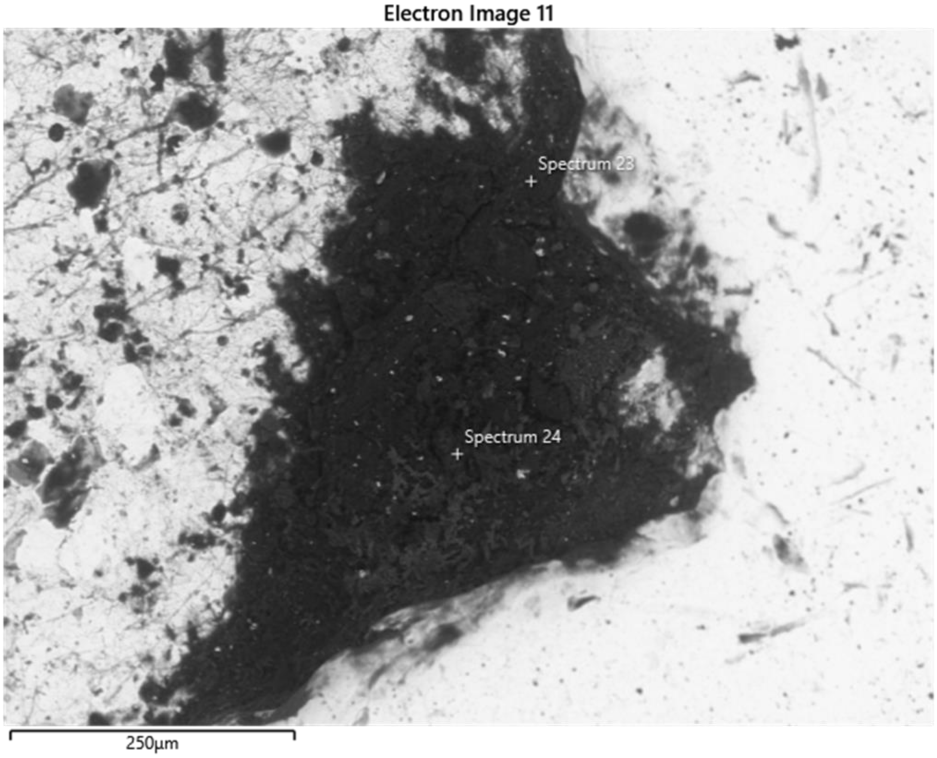

Figure S.3. 50 Area of coin surface showing the beside the 'S' of the obverse legend

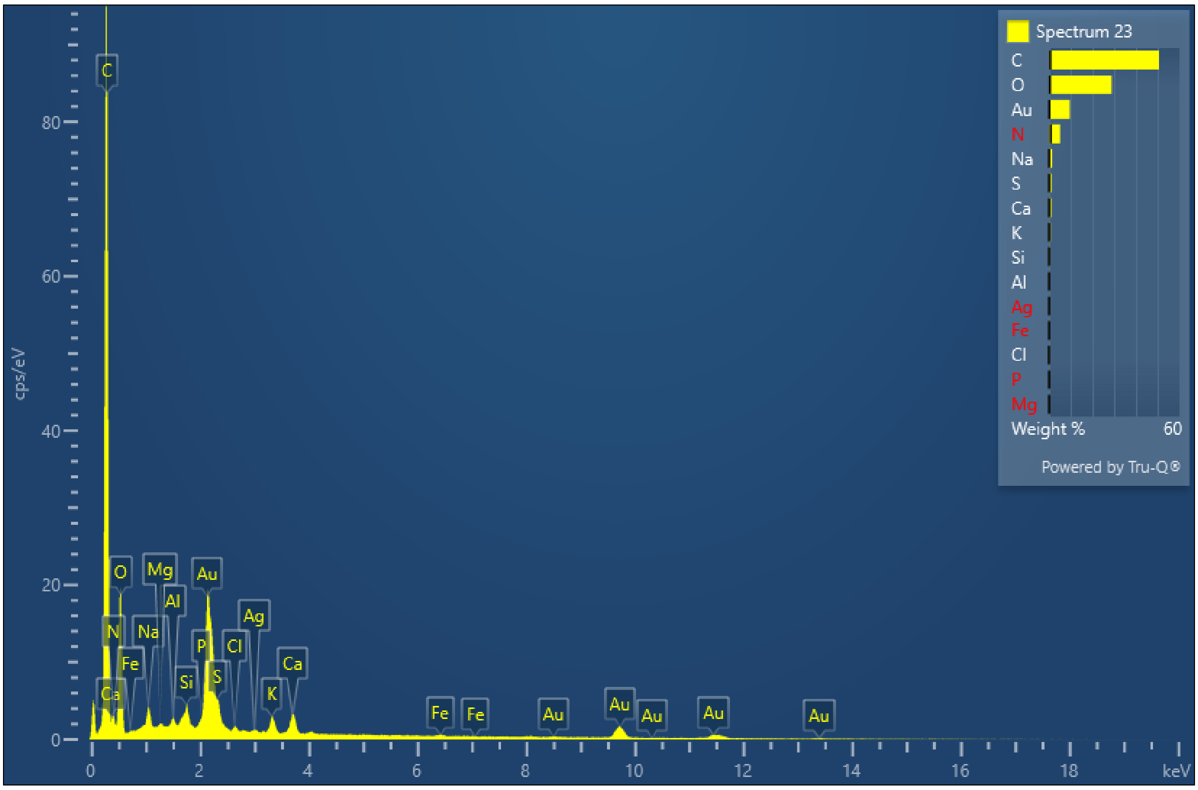

Figure S.3. 51 Spectrum 23 on Coin GLAHM:29596

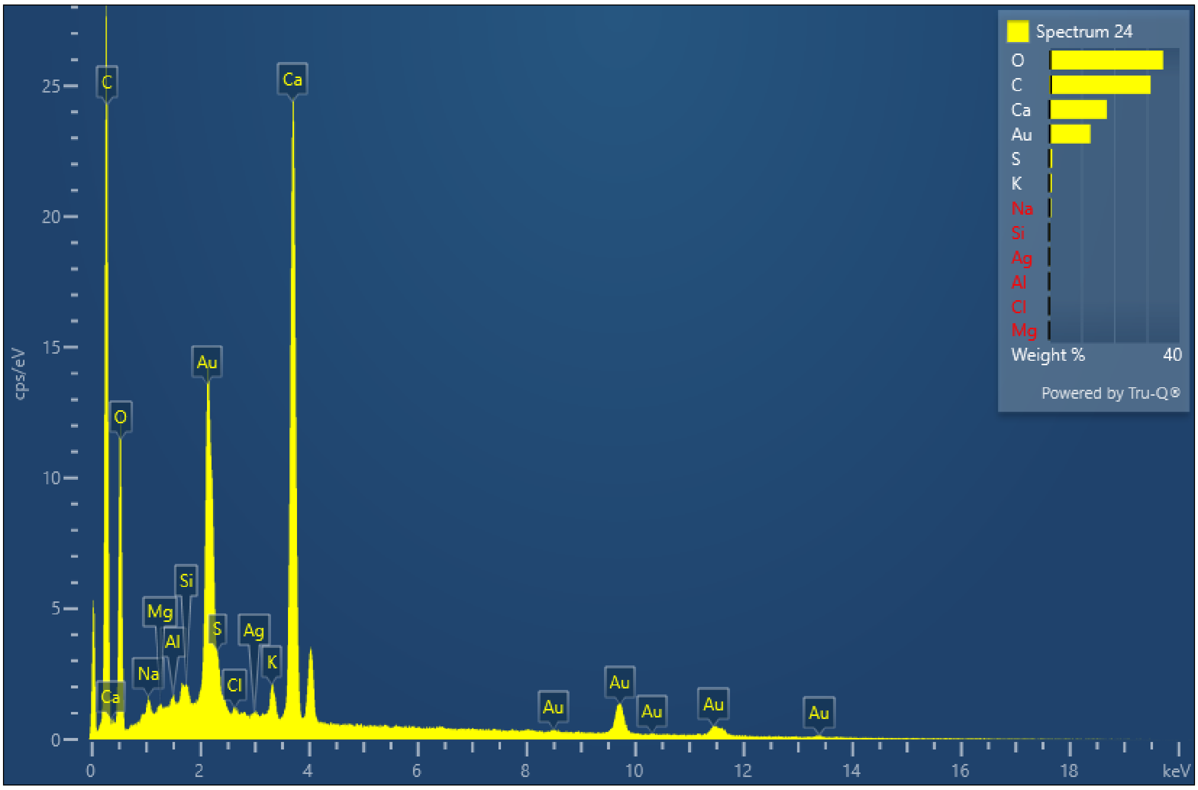

Figure S.3. 52 Spectrum 24 on Coin GLAHM:29596

Discussion: Both spectra are quite complex with prominent Ca and O peaks as well as Au from the coin. Subsidiary peaks correspond to S, K, Na and other cations.

Higher magnification of the same area revealed what appears to be light-coloured bladed crystals of ~10-15 µm length in rosette and fan-shaped arrangements (Spectra 25, 26, 27, 32, 33, 34, 35, 36) with scattered very bright patches around (Spectrum 28), all overlying darker material (Spectra 29, 30, 31).

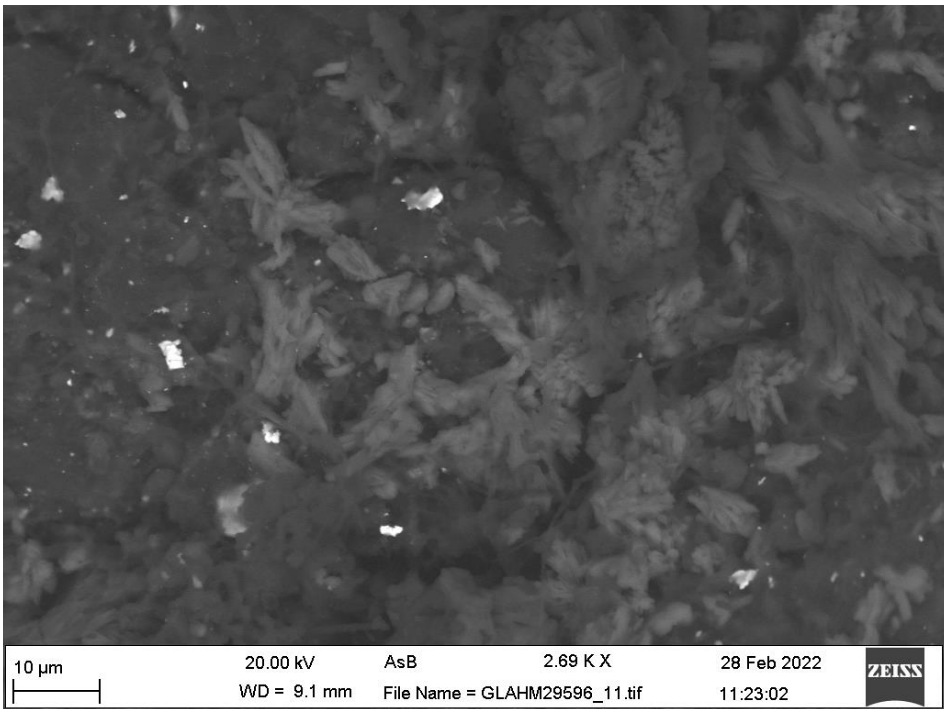

Figure S.3. 53 First investigated area of mineralization within the letter 'S'

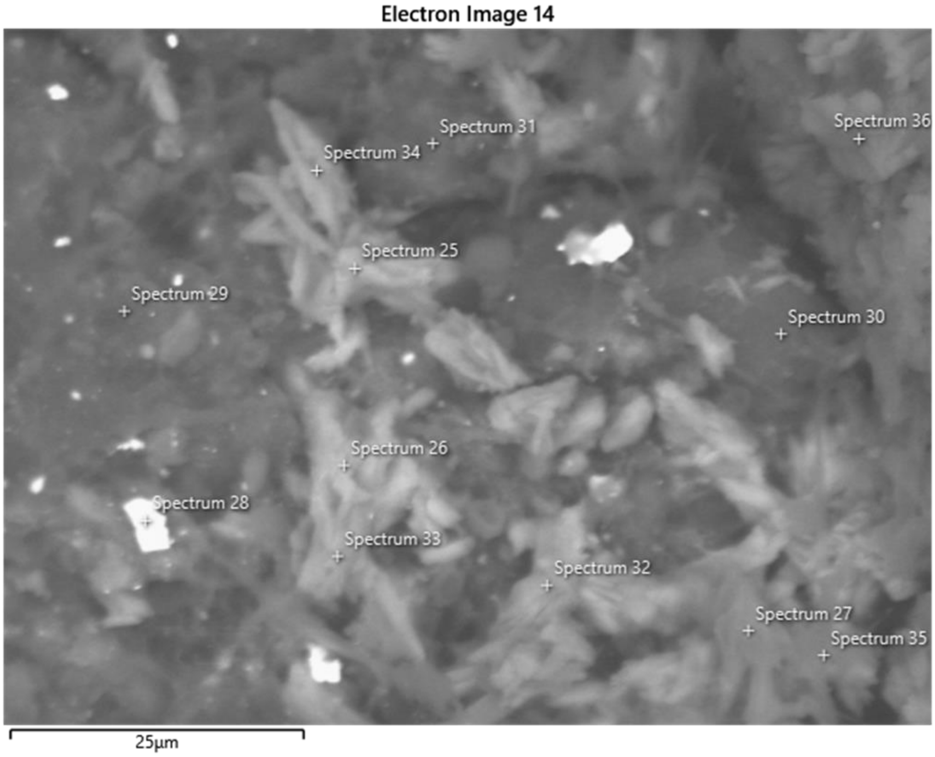

Figure S.3. 54 High magnification image of the area to the left of Spectrum 24 in Figure S.3.33

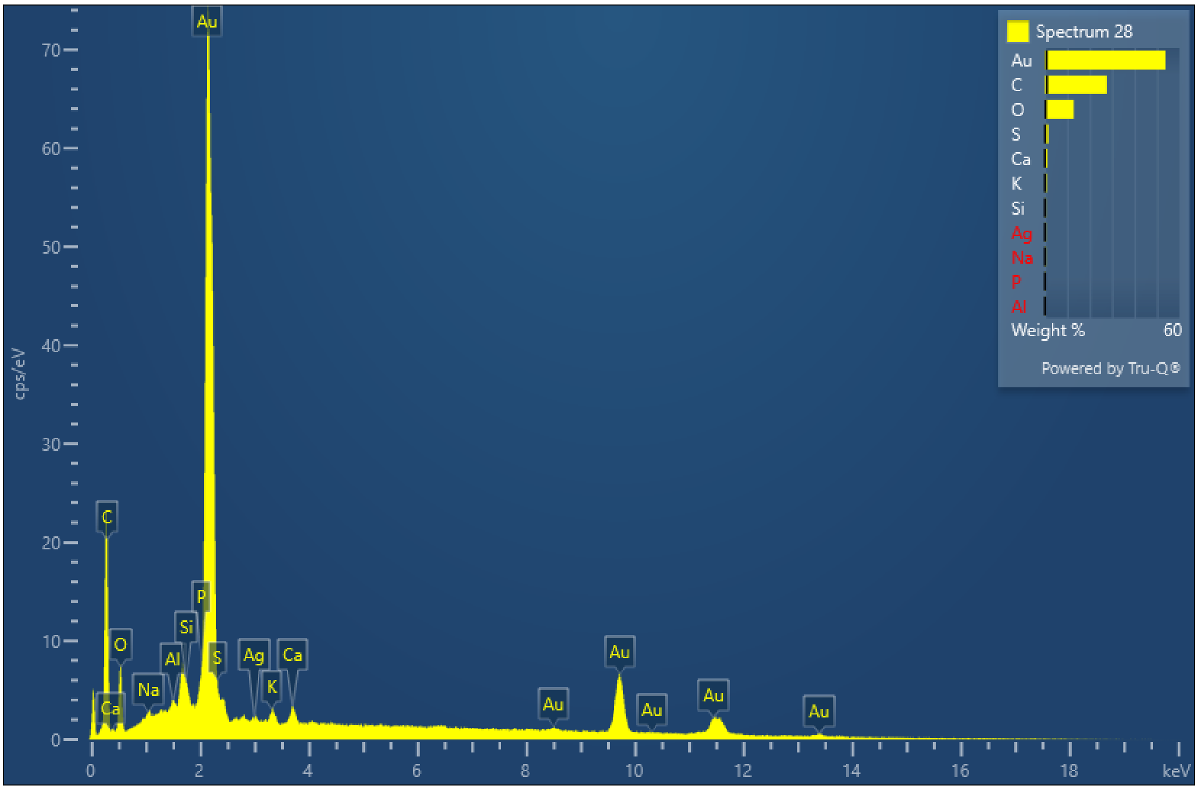

Figure S.3. 55 Spectrum 28 on Coin GLAHM:29596

Discussion: Spectrum 28 was dominated by Au (Figure S37) and is interpreted as a fragment of the coin surface that has spalled off and become associated with the other material.

The eight spectra taken from upper areas of bladed mineralization are given in Figures S38 to S45 below.

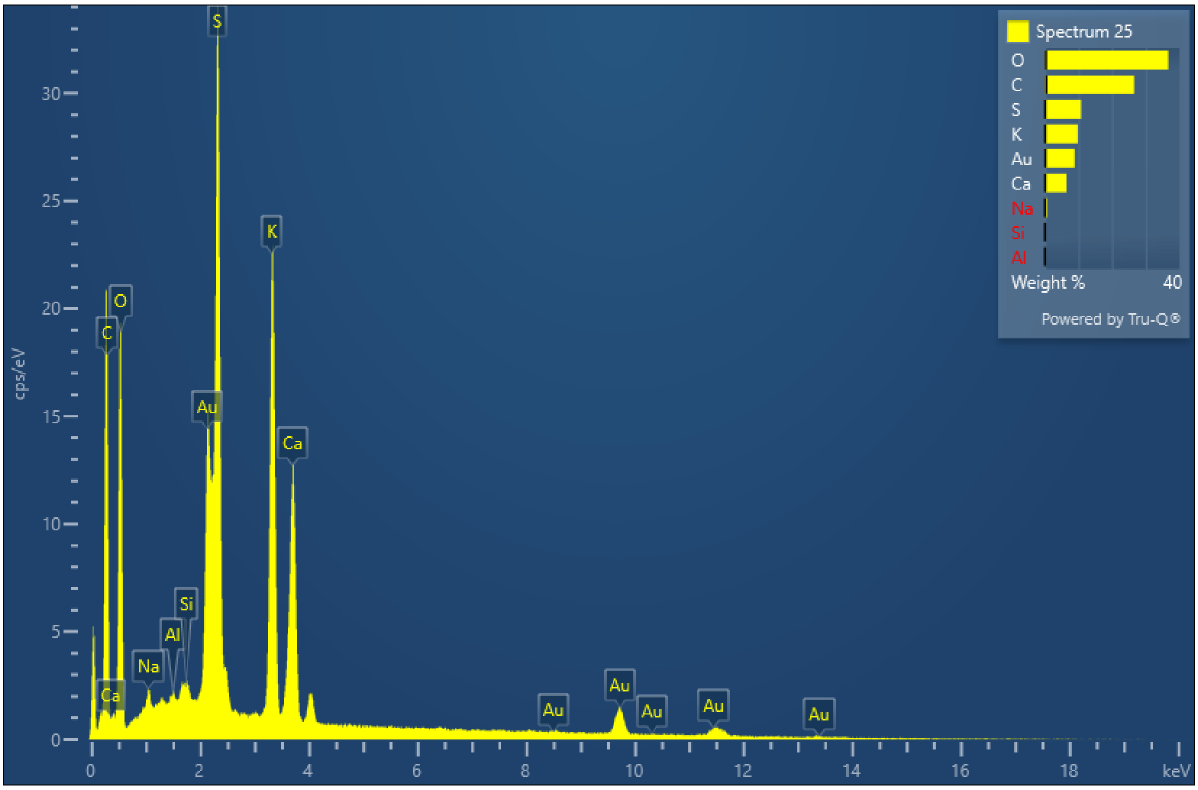

Figure S.3. 56 Spectrum 25 on Coin GLAHM:29596

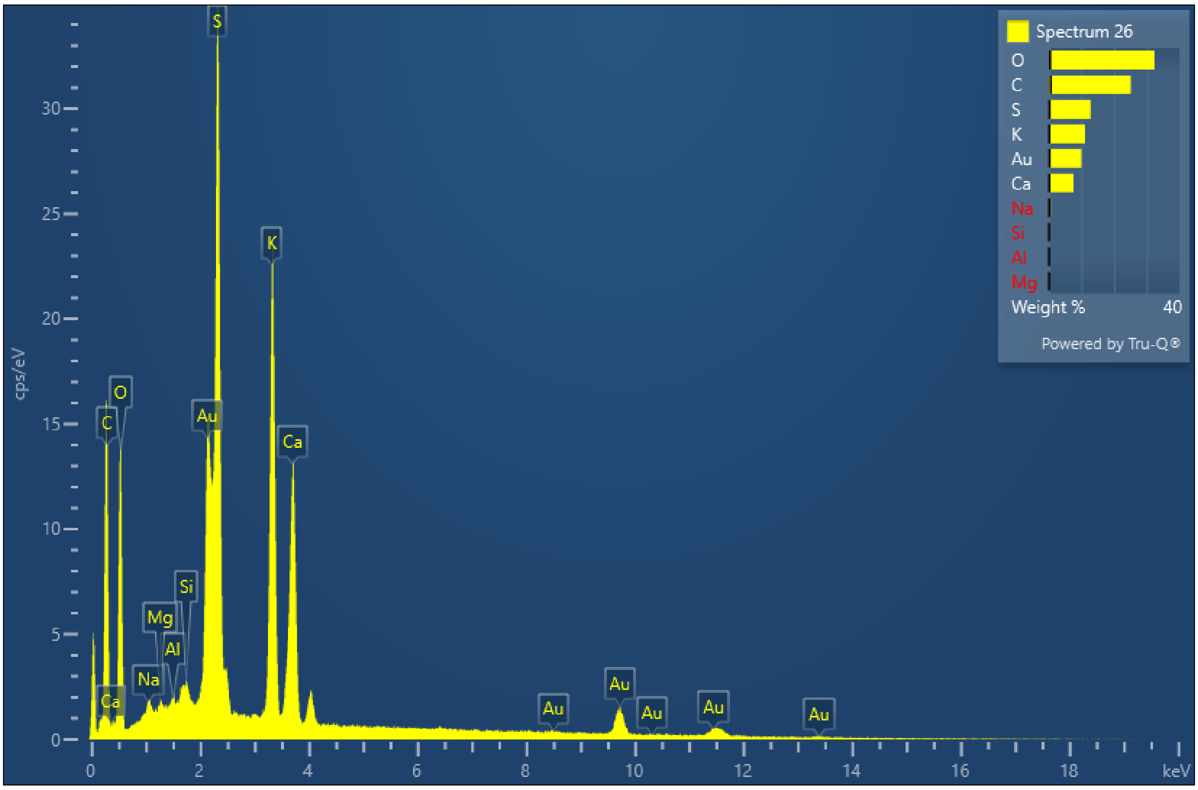

Figure S.3. 57 Spectrum 26 on Coin GLAHM:29596

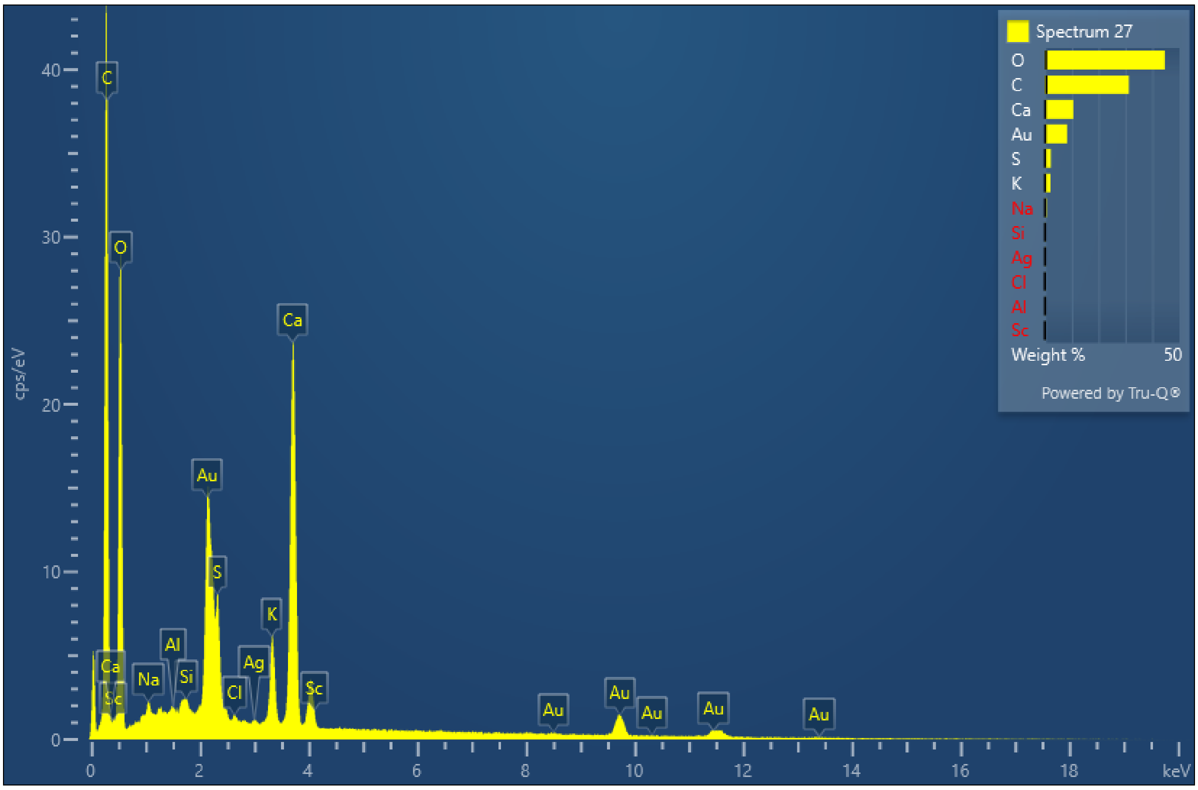

Figure S.3. 58 Spectrum 27 on Coin GLAHM:29596

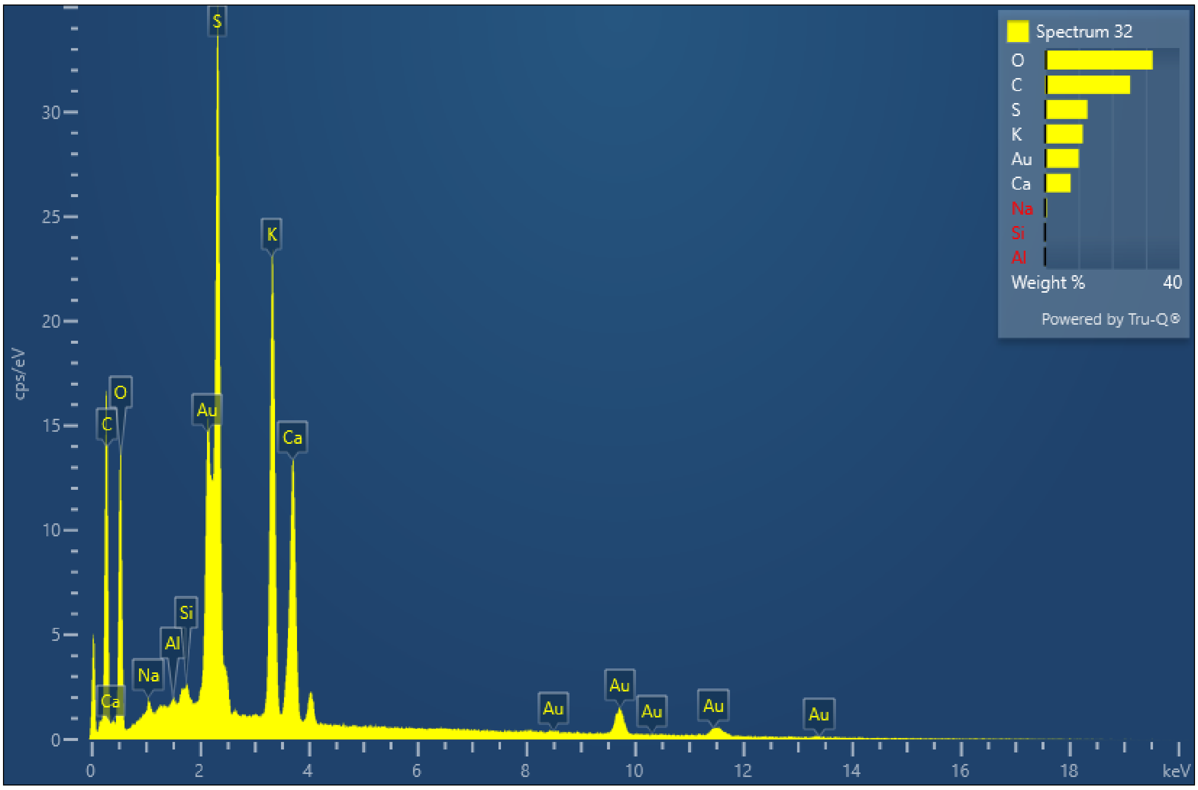

Figure S.3. 59 Spectrum 32 on Coin GLAHM:29596

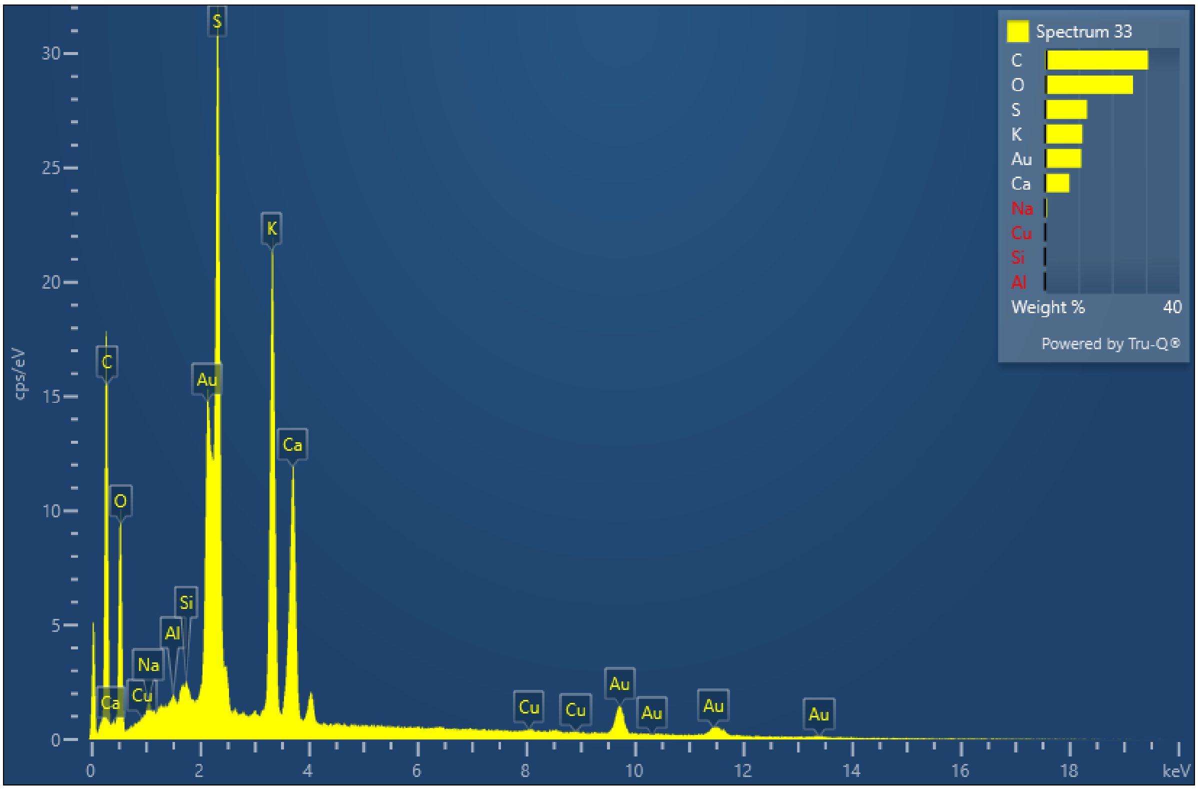

Figure S.3. 60 Spectrum 33 on Coin GLAHM:29596

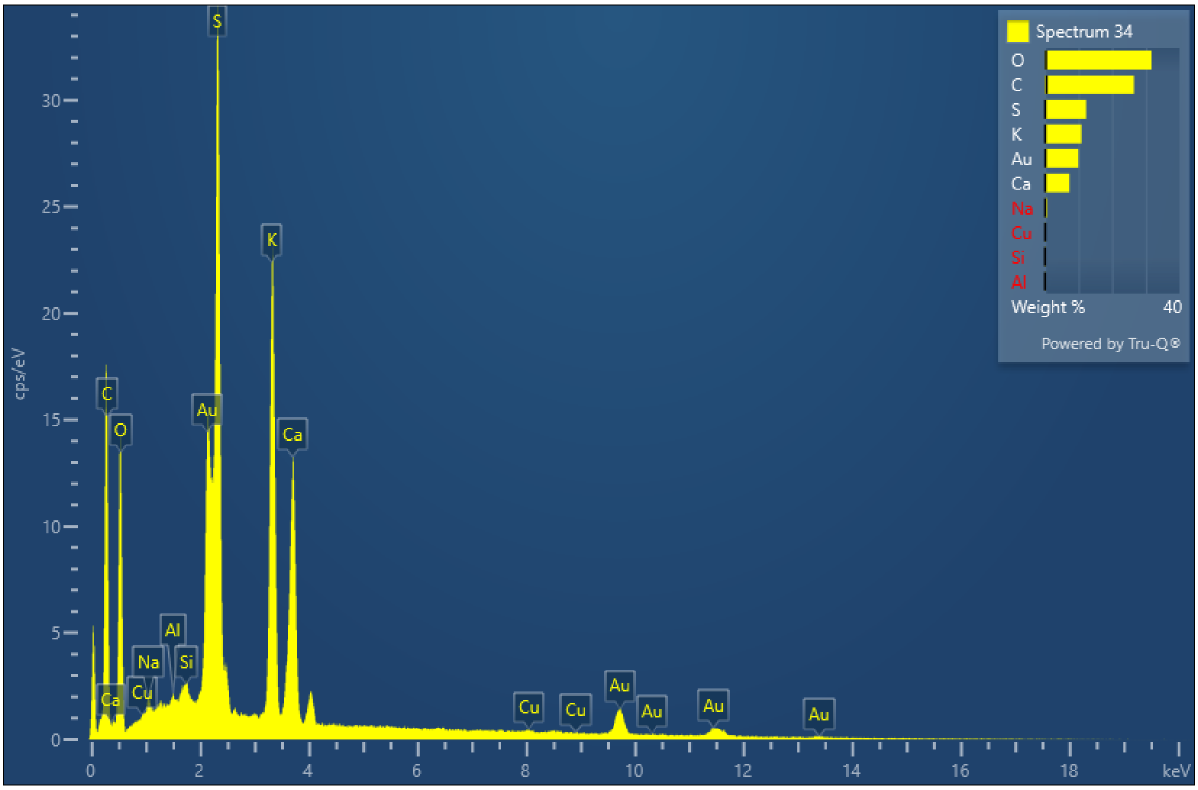

Figure S.3. 61 Spectrum 34 on Coin GLAHM:29596

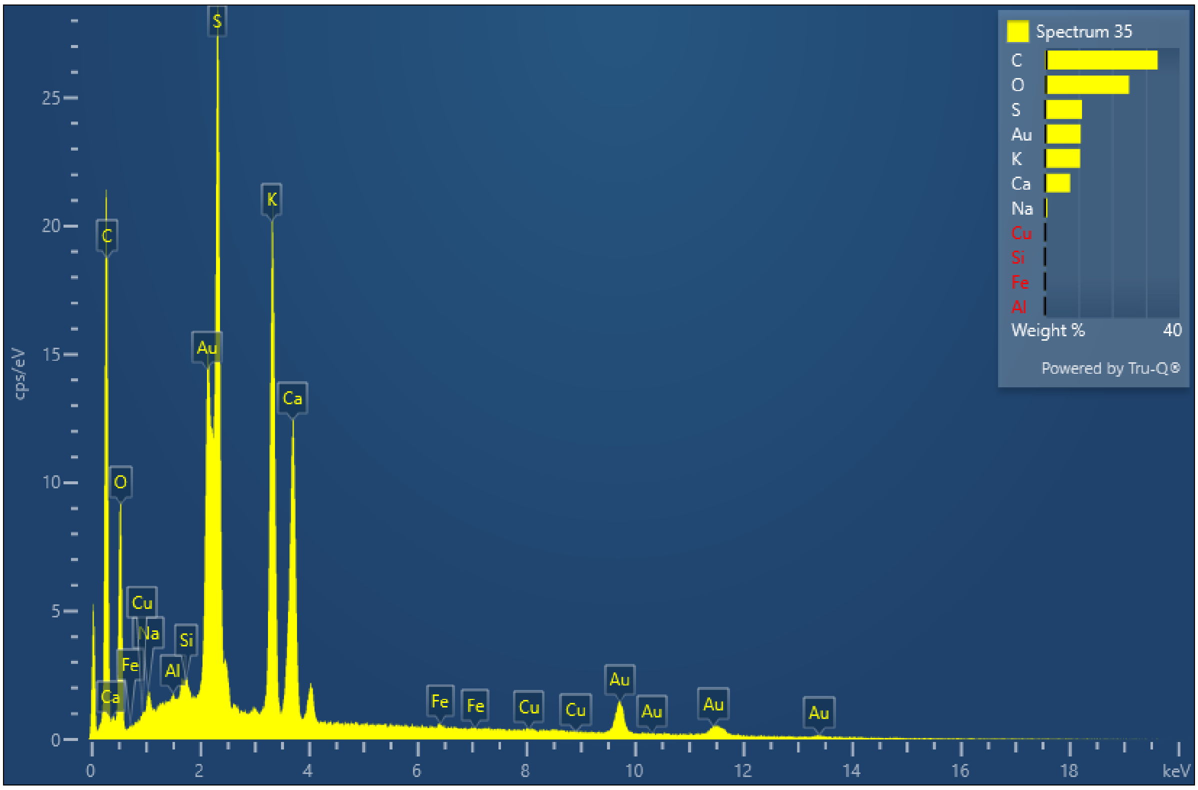

Figure S.3. 62 Spectrum 35 on Coin GLAHM:29596

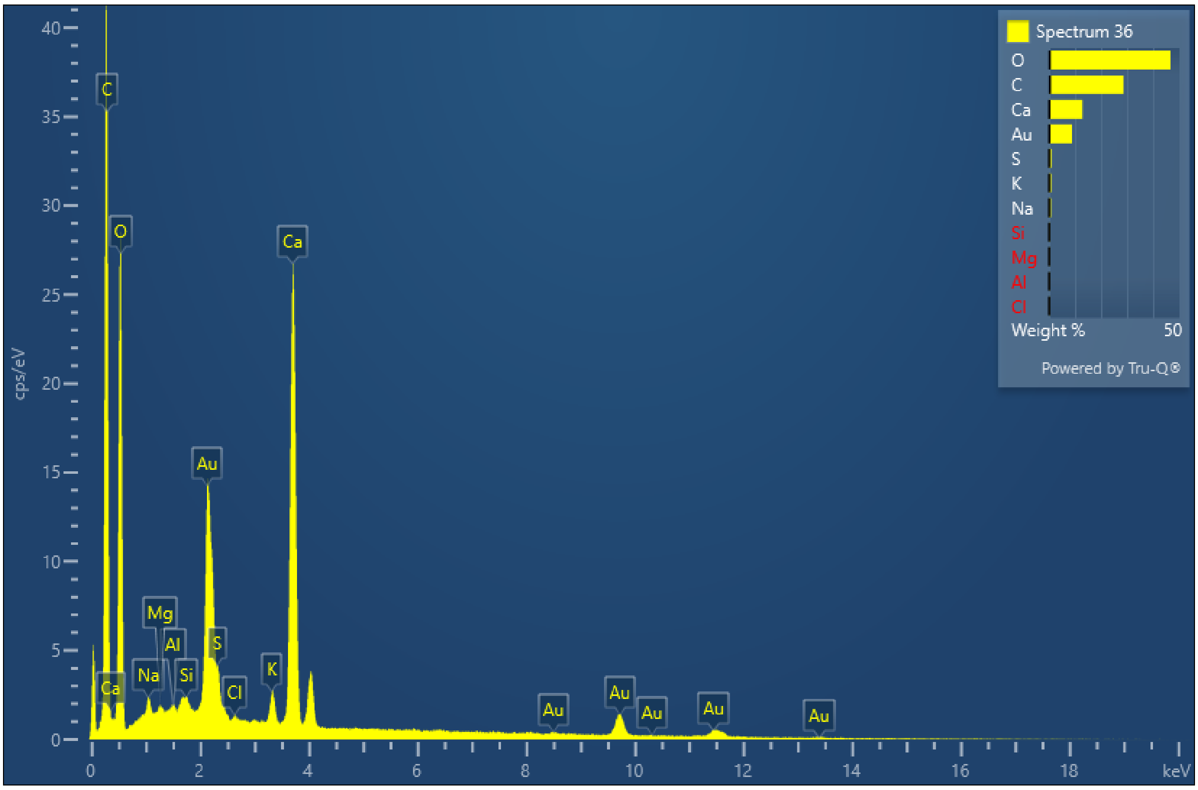

Figure S.3. 63 Spectrum 36 on Coin GLAHM:29596

Discussion: Of these analyses, Spectra 25, 26, 32, 34, and 35 are very similar showing prominent peaks associated with O, S, K, and Ca. The habit and composition indicate the

potassic calcium sulphate salt syngenite ($K_2Ca(SO_4)_2 \cdot H_2O$). This is an unusual mineral which is related to gypsum and can be formed by the transformation of gypsum with a potassic reagent [1]. Spectra 26 and 36 show much lower K peaks and are dominated by Ca, C, O and S, consistent with calcite ($CaCO_3$) and gypsum ($CaSO_4.2H_2O$).

The next set of spectra (29, 30, 31) were taken from spots on the amorphous darker, underlying material (see Figure S.3.38):

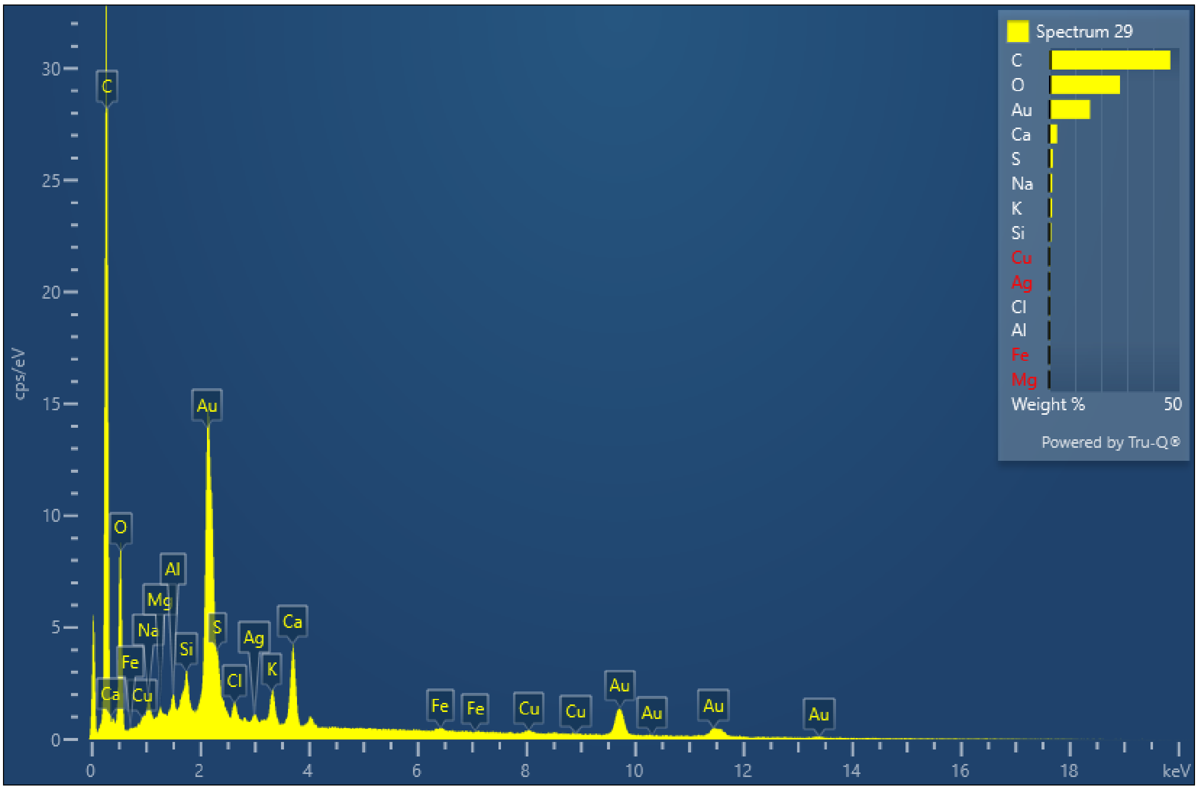

Figure S.3. 64 Spectrum 29 on Coin GLAHM:29596

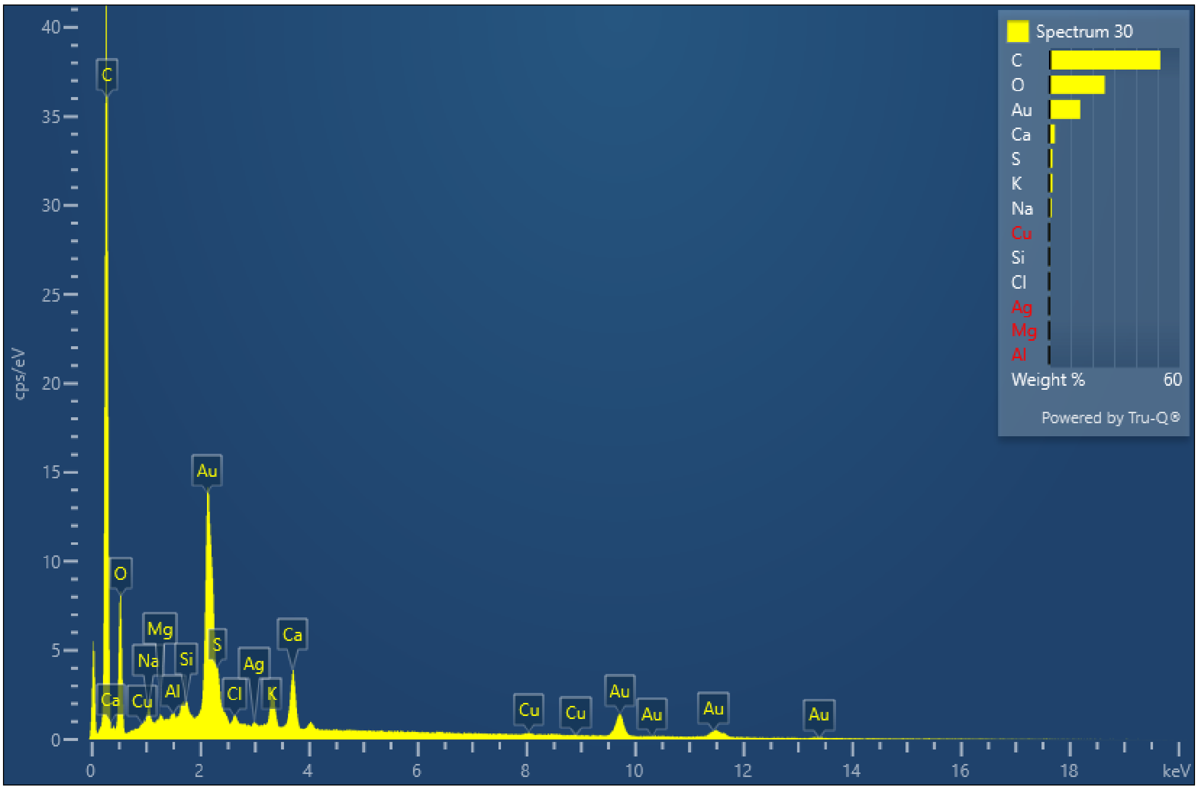

Figure S.3. 65 Spectrum 30 on Coin GLAHM:29596

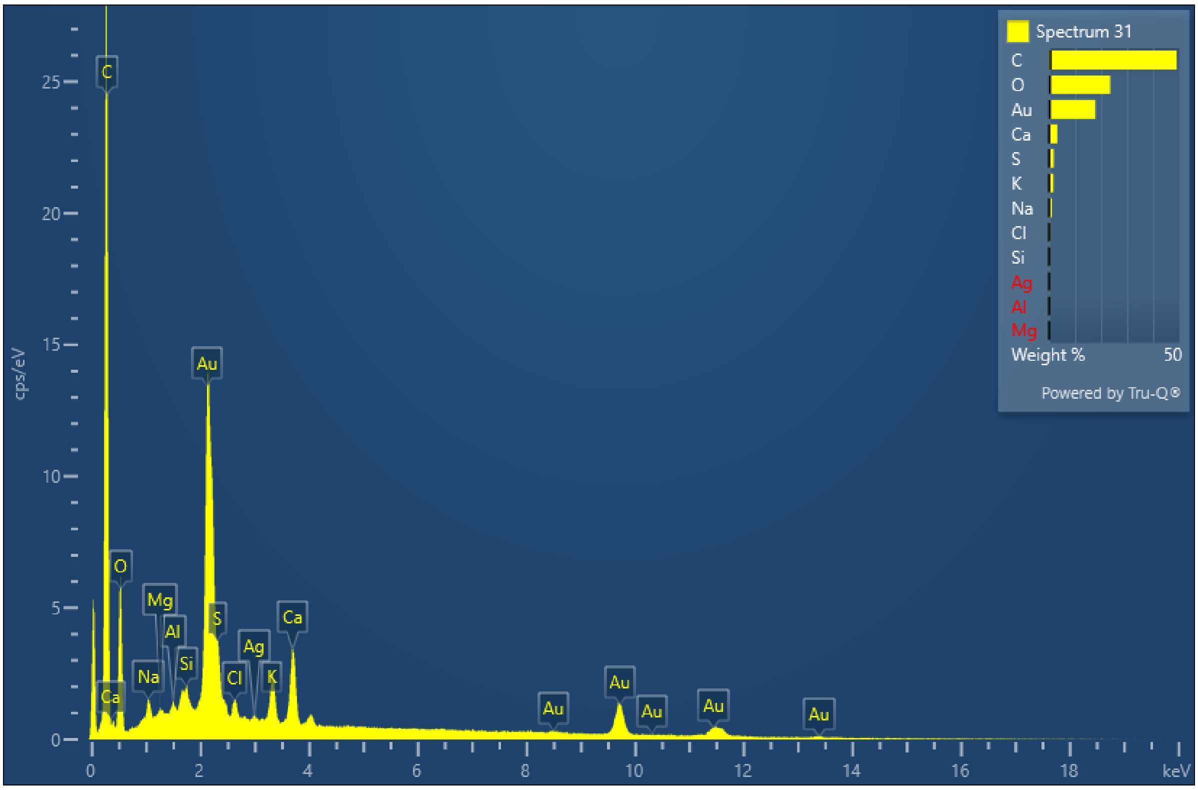

Figure S.3. 66 Spectrum 31 on Coin GLAHM:29596

Discussion: The above three spectra are extremely similar. Signals from Au and Ag are interpreted as derived from the underling metal. Prominent peaks are O, Ca, S, K, Na and Si.

The material appears to be some form of complex cement, possibly with feldspathic, opaline and/or carbonate origin. To further investigate these possibilities another nearby area was selected for analysis (Figure S33).

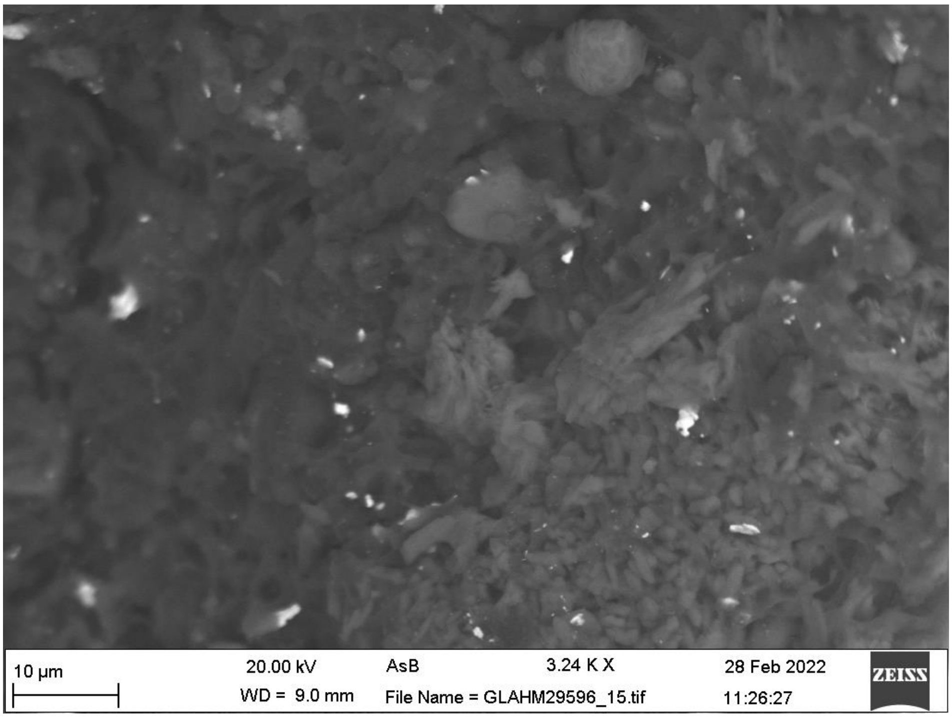

Figure S.3. 67 Second investigated area of mineralization within the letter 'S'

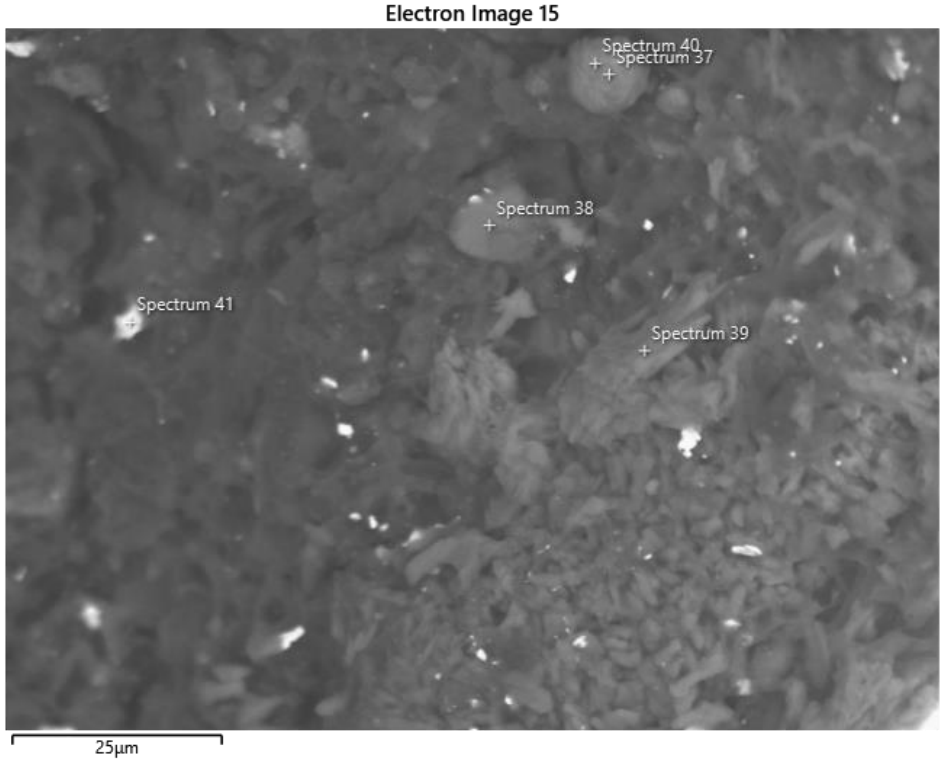

Figure S.3. 68 High magnification image of the area to the top right of Spectrum 24 in Figure S33

One spectrum (41) was taken from a bright flake in the image, another (39) an area dominated by a cluster of bladed crystals, and three spectra (37, 38, 40) from roundish agglomerates of similar brightness under SEM, as shown in order:

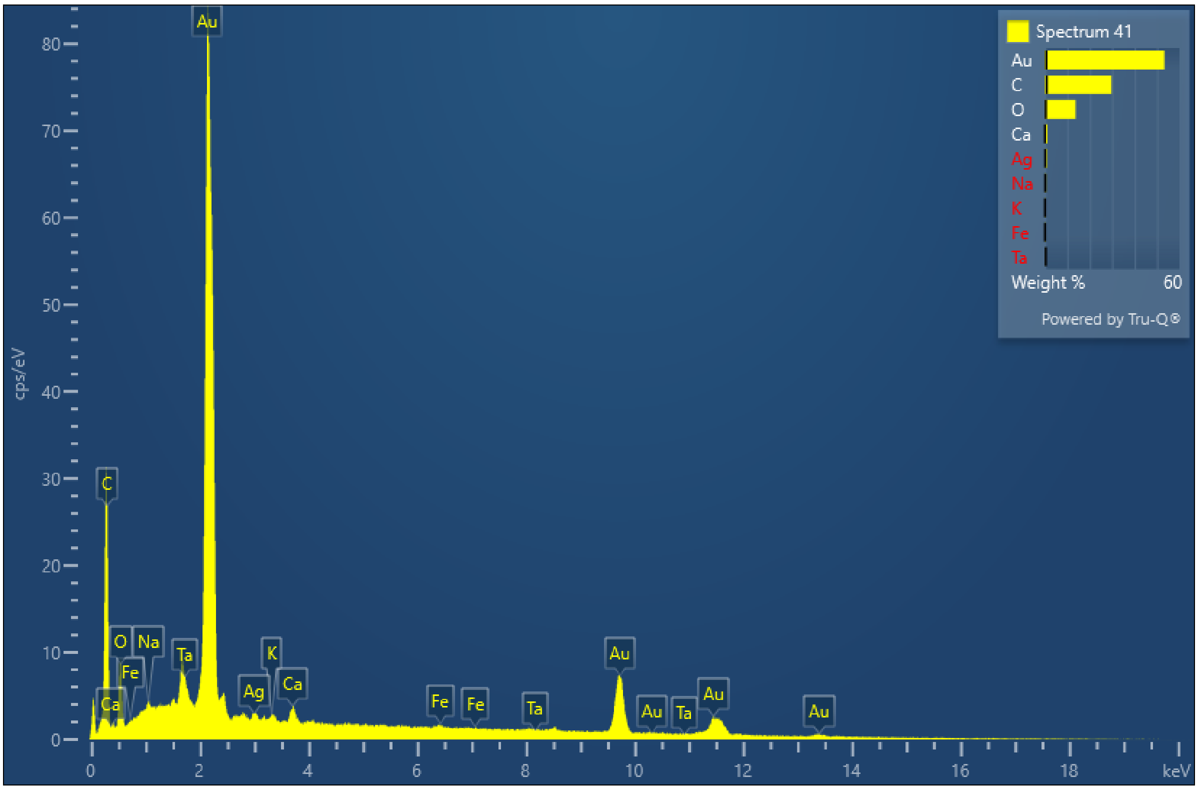

Figure S.3. 69 Spectrum 41 on Coin GLAHM:29596

Discussion: This spectrum is similar to Spectrum 28 and appears to be a metallic flake from the coin surface.

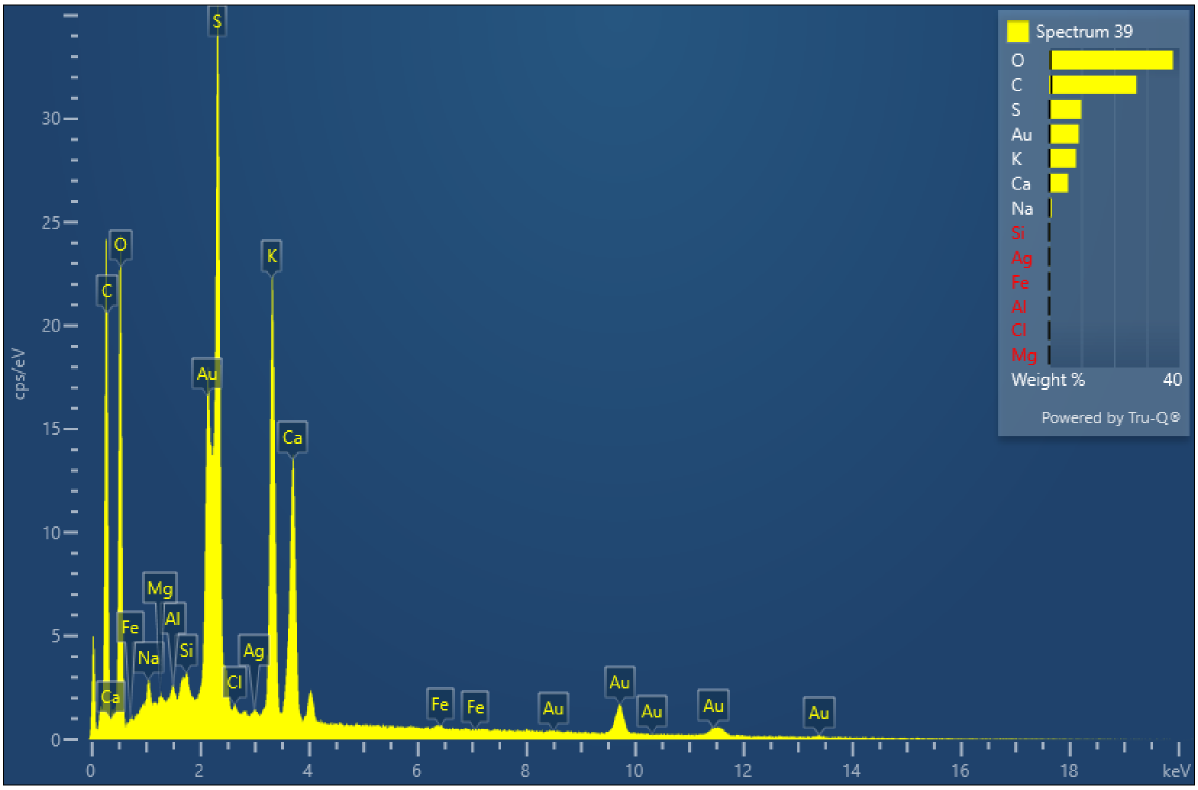

Figure S.3. 70 Spectrum 39 on Coin GLAHM:29596

Discussion: This spectrum is similar to Spectra 25, 26, 32, 34, and 35 and is interpreted as representing a syngenite crystal.

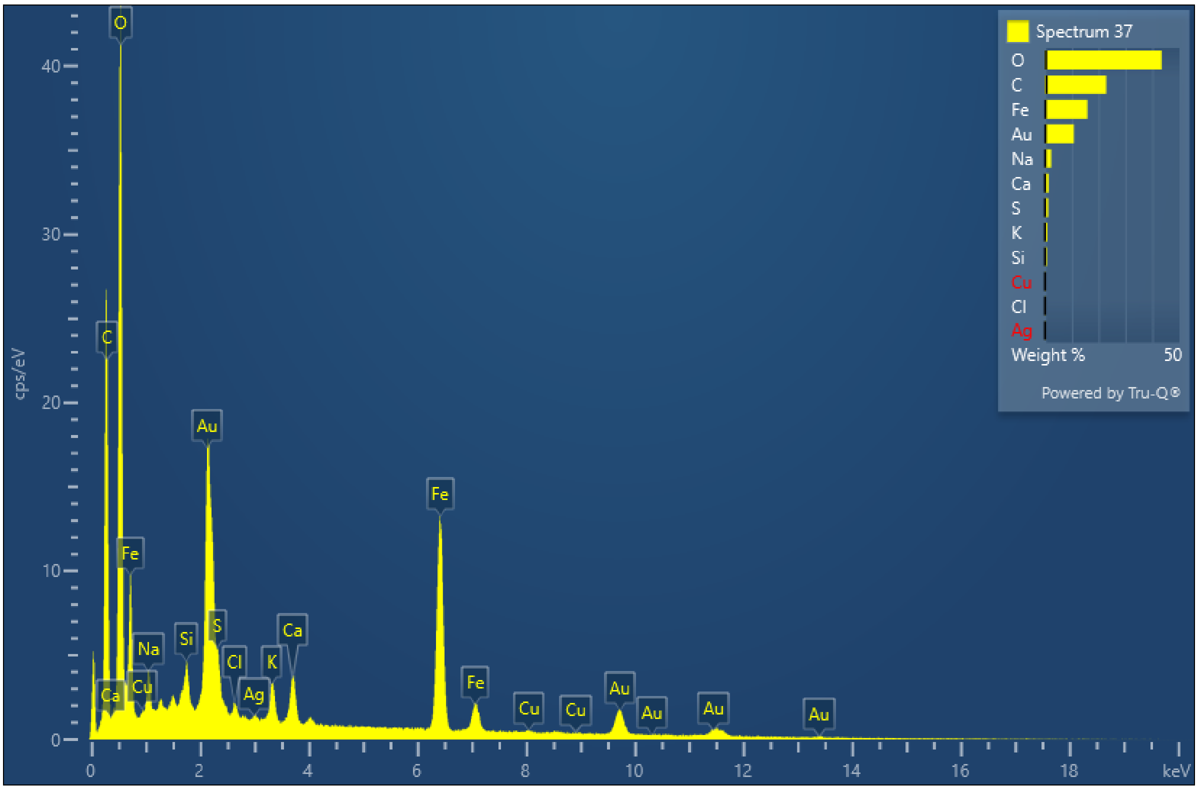

Figure S.3. 71 Spectrum 37 on Coin GLAHM:29596

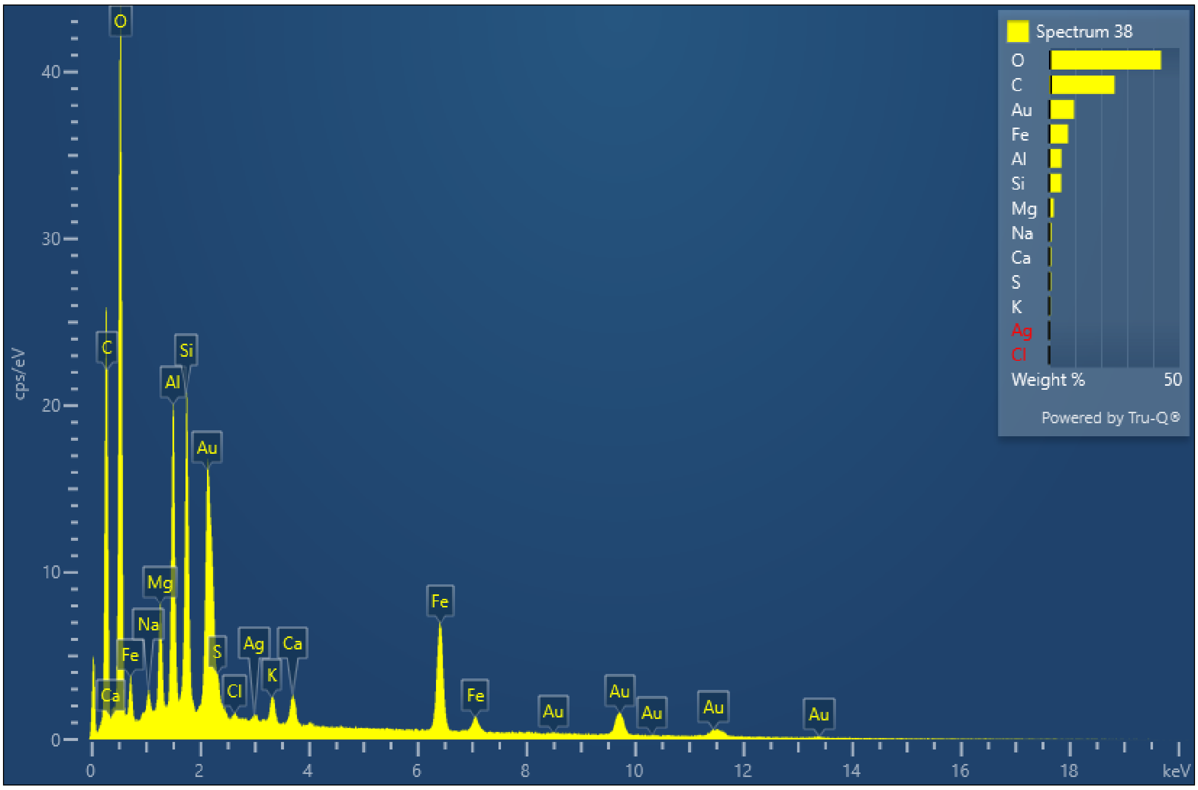

Figure S.3. 72 Spectrum 38 on Coin GLAHM:29596

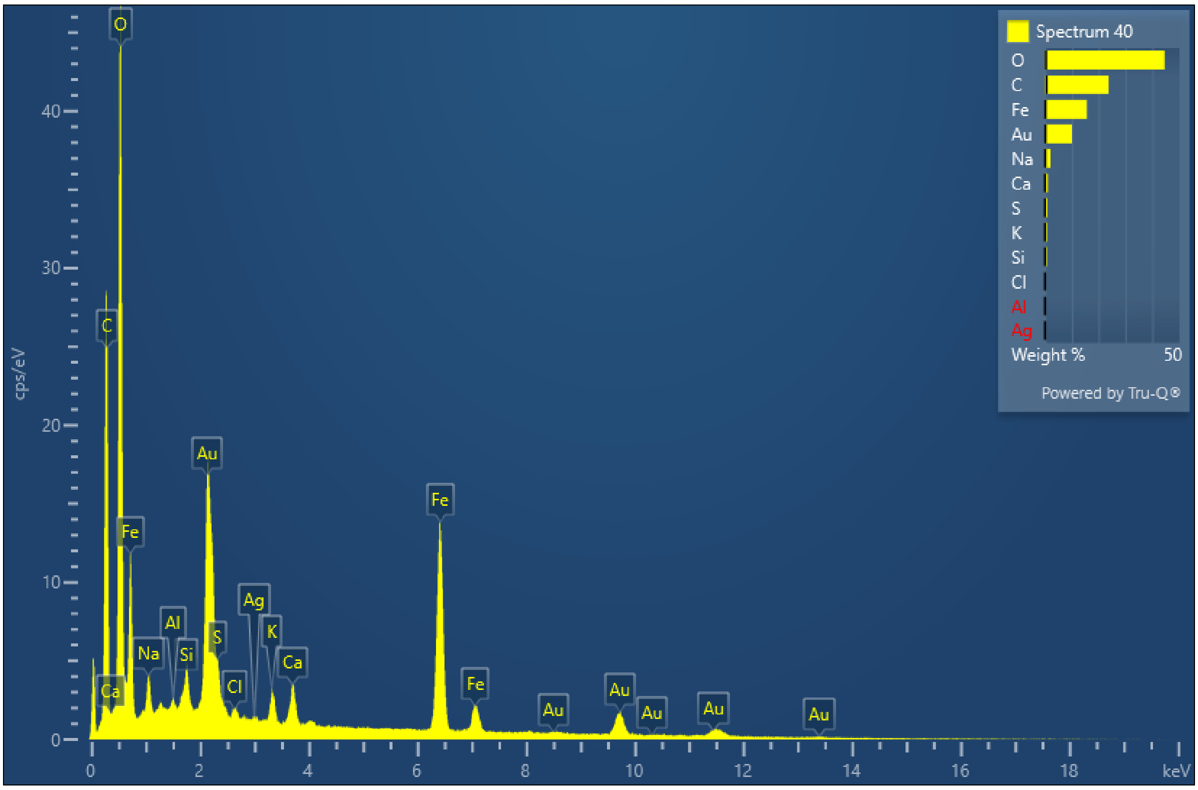

Figure S.3. 73 Spectrum 40 on Coin GLAHM:29596

Discussion: This group of three spectra are similar to one another and are characterised by prominent peaks associated with Fe, C and O and, in the case of Spectrum 38, Al. The main mineral phase may be siderite ($FeCO_3$).

S.3.4 Coin GLAHM:29820 (Questionable Philip I medallion)

S.3.4.1 Gallery of surface images

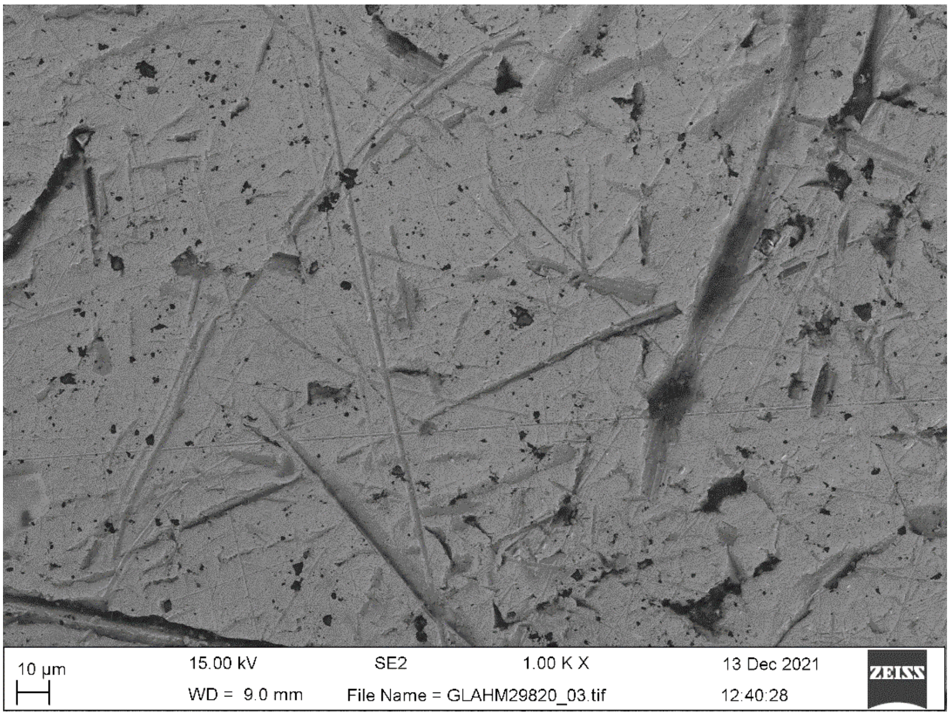

Figure S.3. 74 Area of Roma's head at 1000x magnification

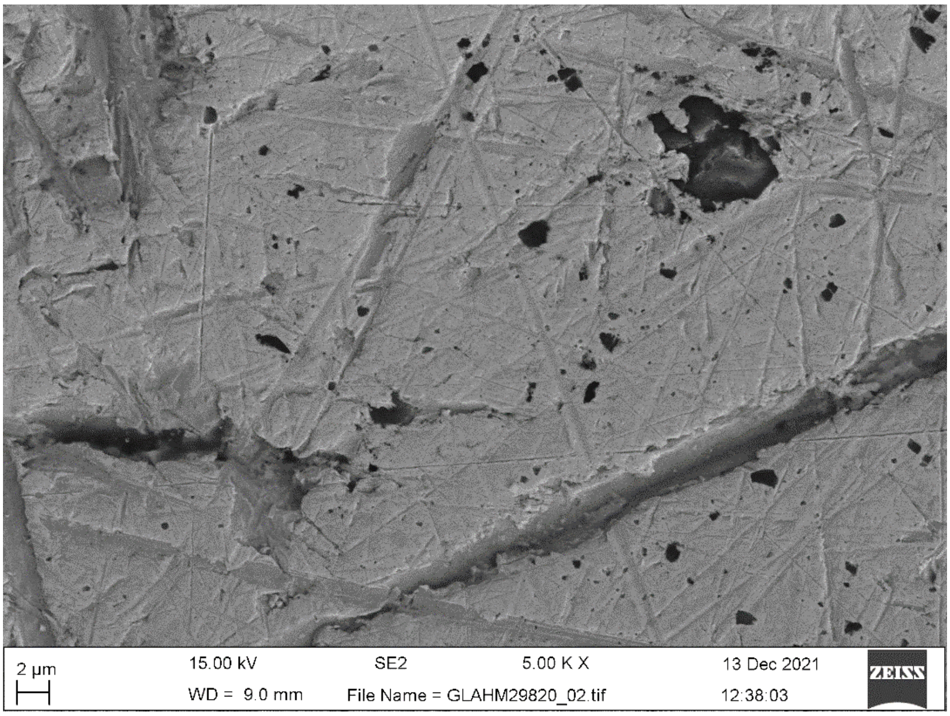

Figure S.3. 75 Area of Roma's head at 5000x magnification

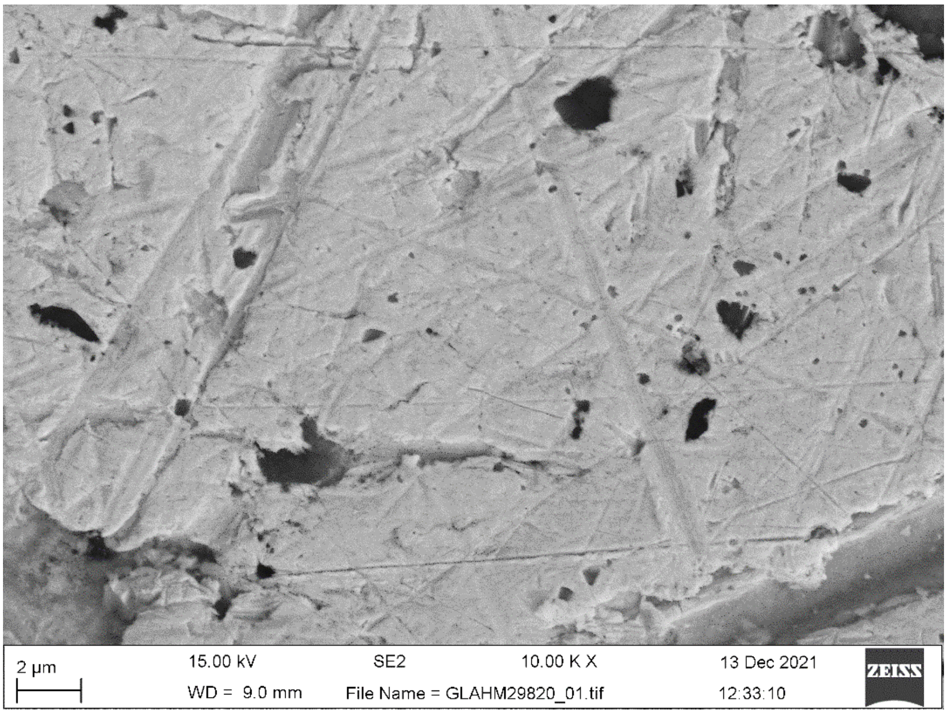

Figure S.3. 76 Area of Roma's head at 10000x magnification

S.3.4.1 Metal composition

Three spectra (82-84) were obtained from a worn and exposed part of the head of Roma on the obverse side as follows:

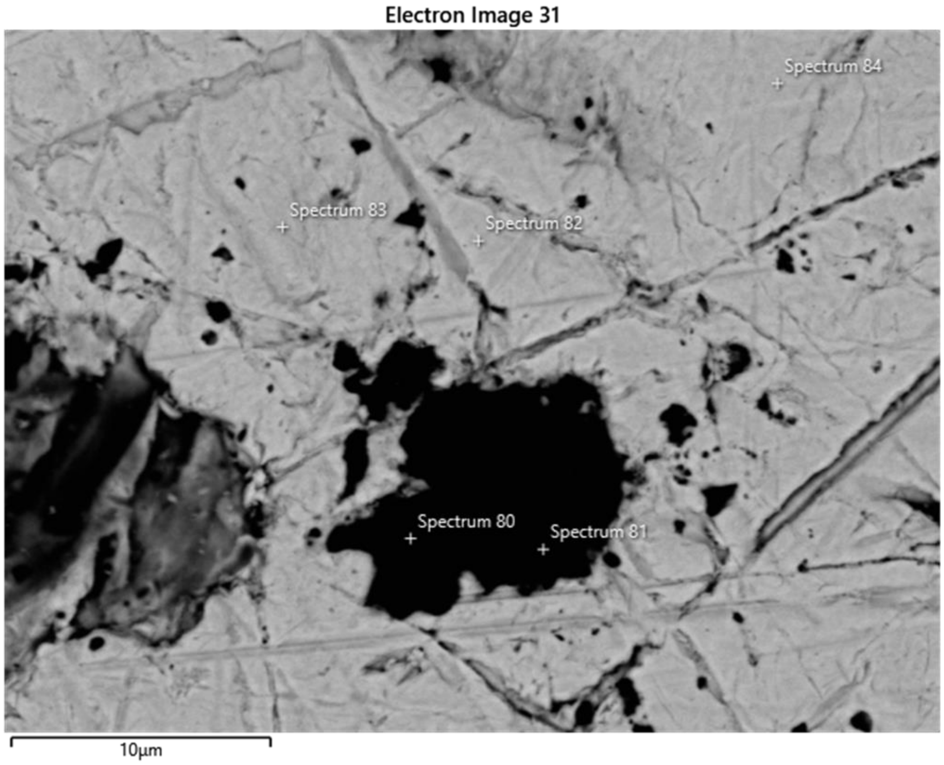

Figure S.3. 77 Area of analyses on the worn area of Coin GLAHM:29820

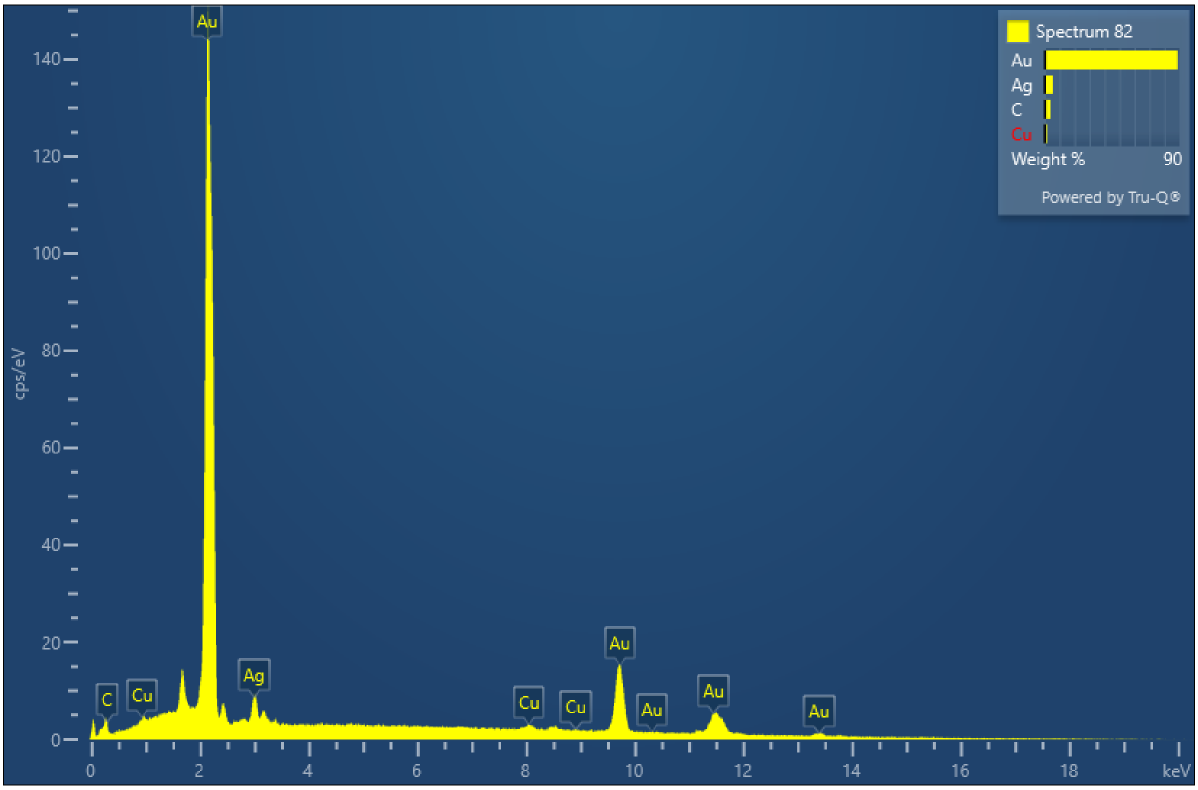

Figure S.3. 78 Spectrum 82 on Coin GLAHM:29820

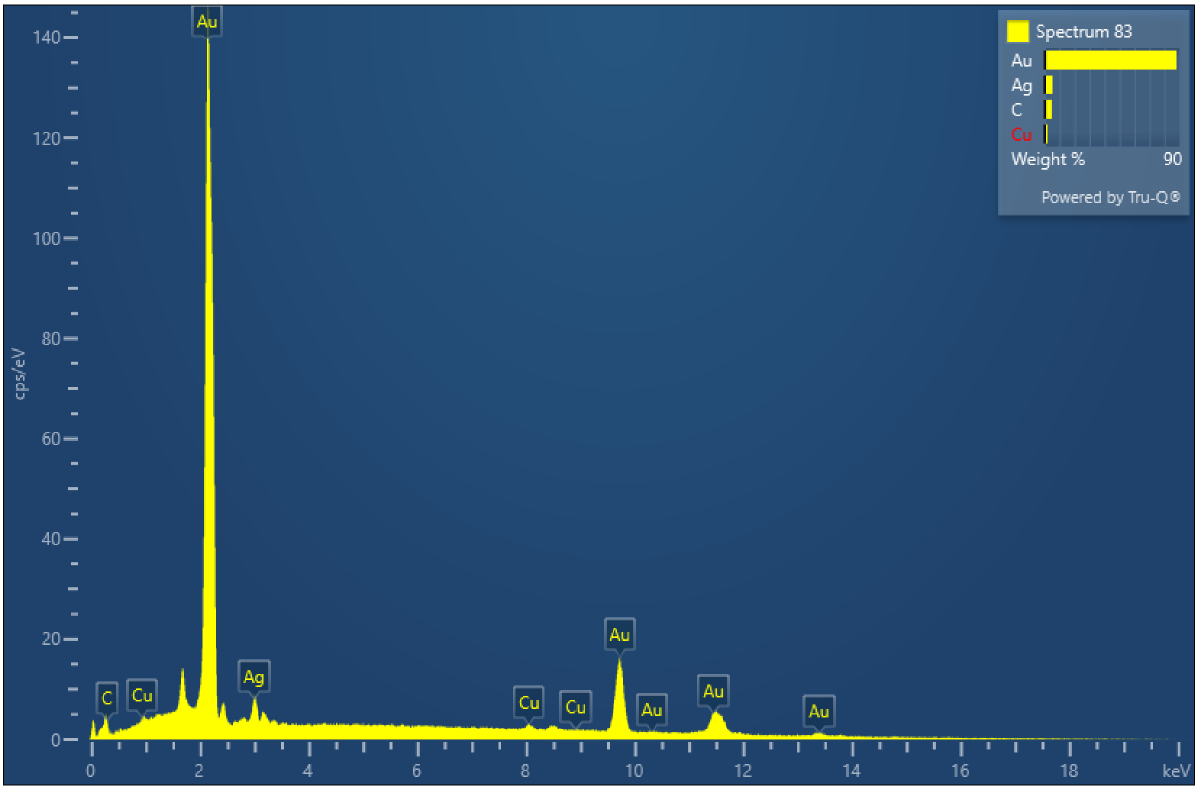

Figure S.3. 79 Spectrum 83 on Coin GLAHM:29820

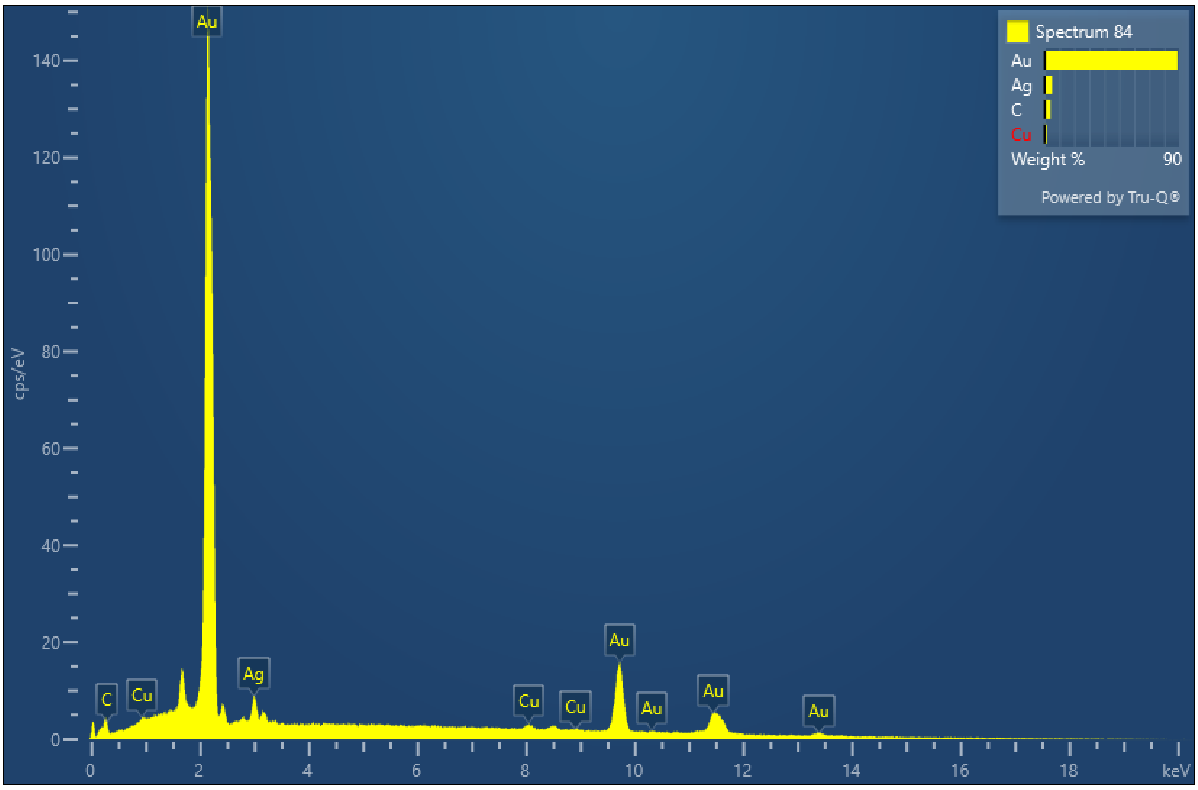

Figure S.3. 80 Spectrum 84 on Coin GLAHM:29820

Discussion: These three spectra are similar to one another and indicate a composition of approximately 92% gold with 6.5% silver and 1.5% copper (Table S3).

GLAHM 29820			
Spectrum Label	Au	Ag	Cu
Spectrum 82	91.84	6.85	1.31
Spectrum 83	91.98	6.47	1.55
Spectrum 84	92.28	6.3	1.43
average	*92.03*	*6.54*	*1.43*
standard dev P	*0.18*	*0.23*	*0.10*
standard dev S	0.22	0.28	0.12

Table S.3. 3 Analyses of metal on Coin GLAHM:29596

S.3.4.2 Superficial deposits

Spectra 80 and 81 were taken from a superficial patch of material with a dark appearance under SEM (see Figure S.3.58), as follows:

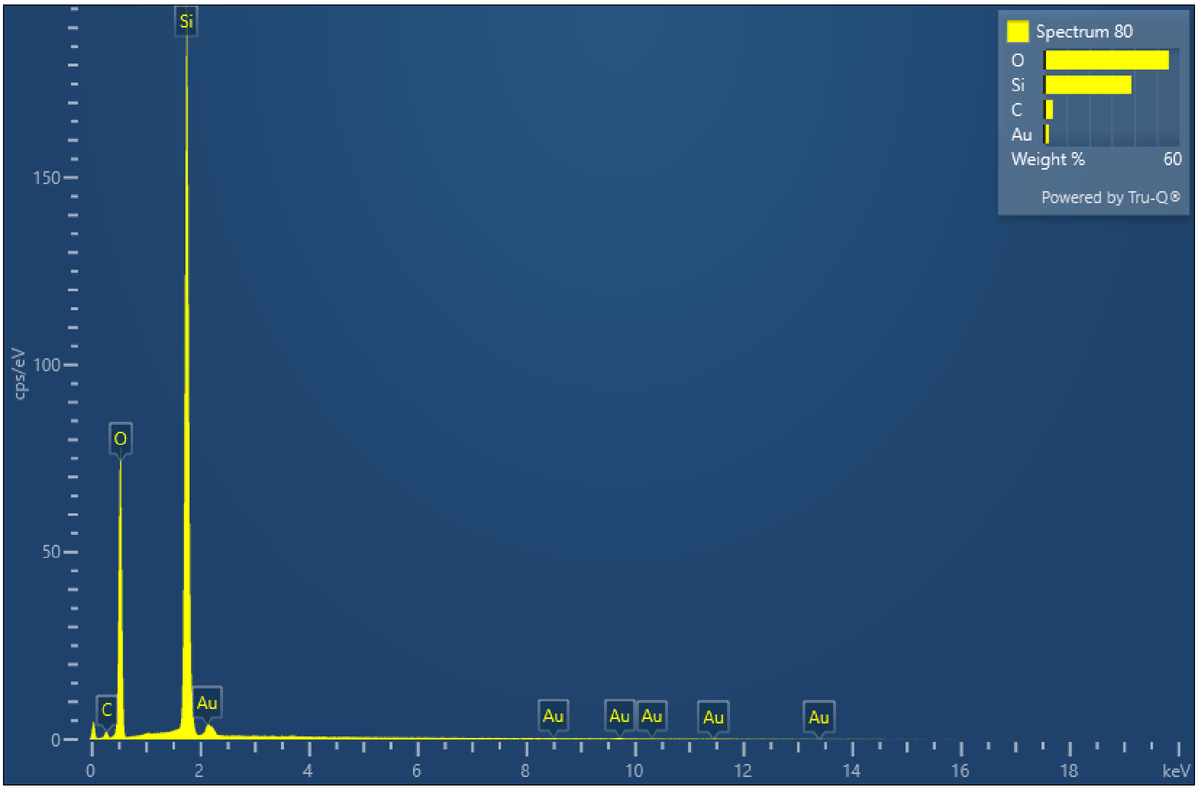

Figure S.3. 81 Spectrum 80 on Coin GLAHM:29820

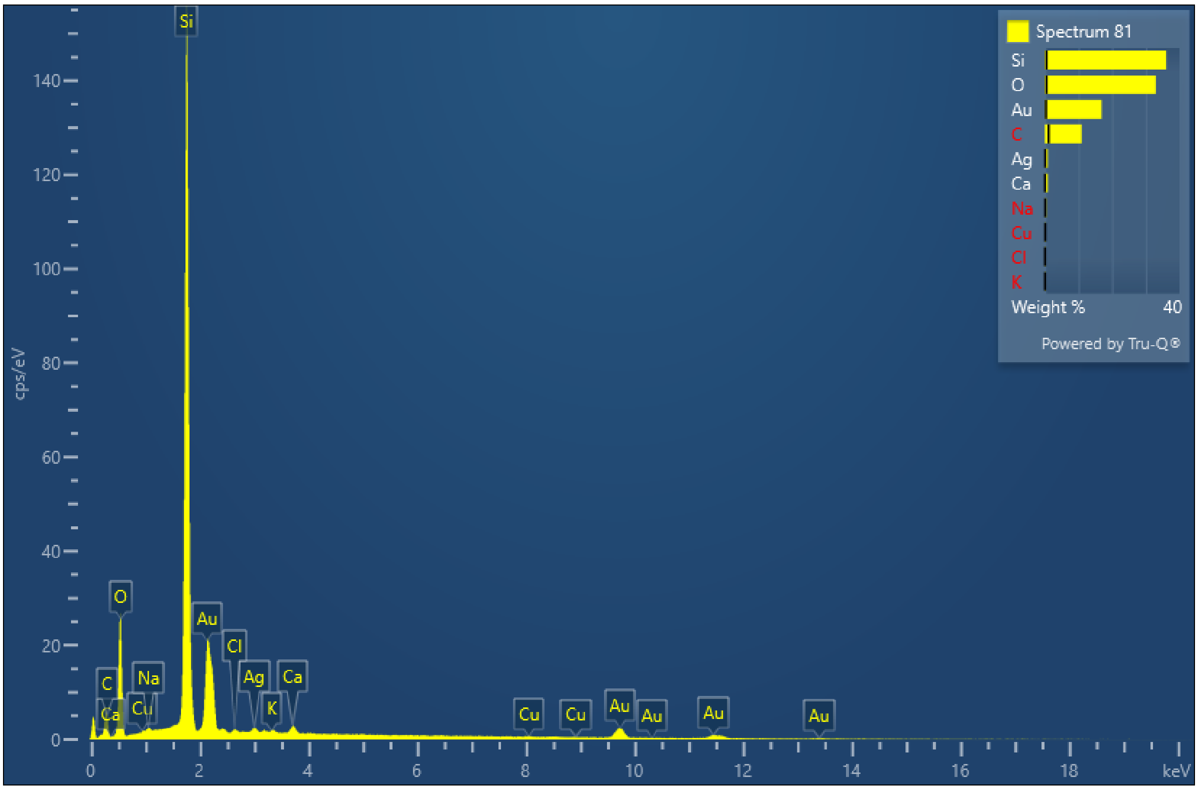

Figure S.3. 82 Spectrum 81 on Coin GLAHM:29820

Discussion: These two spectra are dominated by peaks associated with Si and O and are interpreted as amorphous silica cement, with traces of Au from the underlying coin. Spectrum 81 also includes a minor but significant Ca component.

S.3.5 Coin GLAHM:29821 (Questionable Philip I medallion)

S.3.5.1 Gallery of images

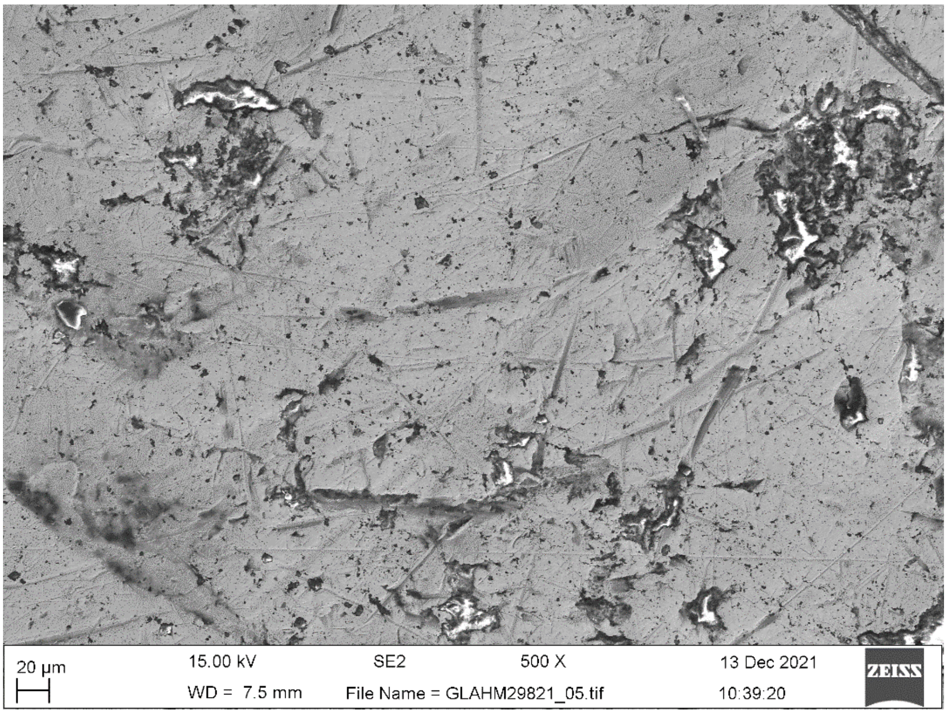

Figure S.3. 83 Area of Roma's head at 500x magnification

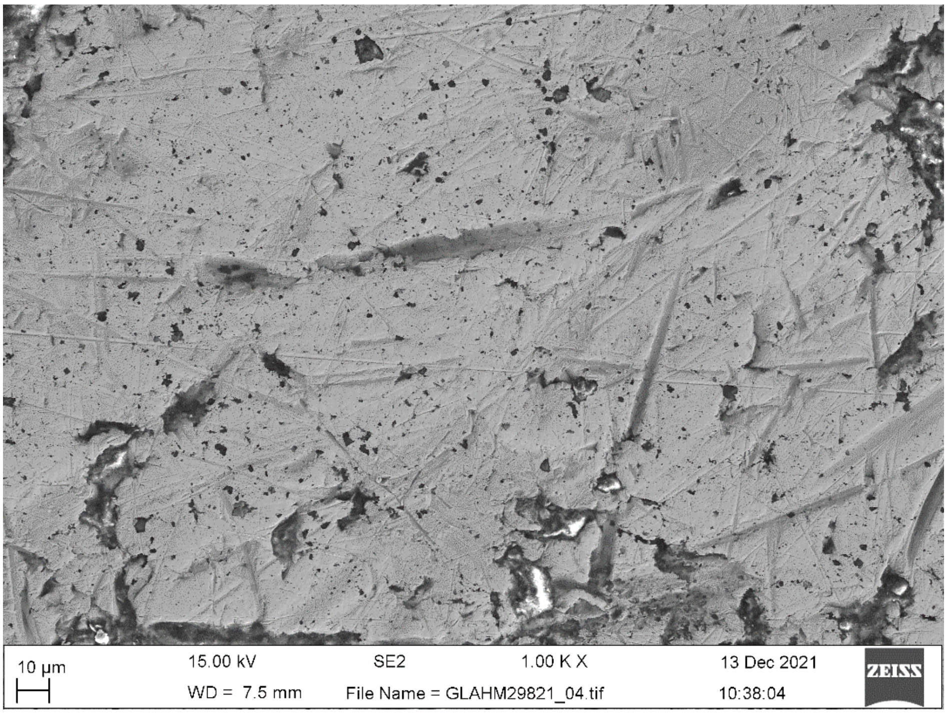

Figure S.3. 84 Area of Roma's head at 1000x magnification

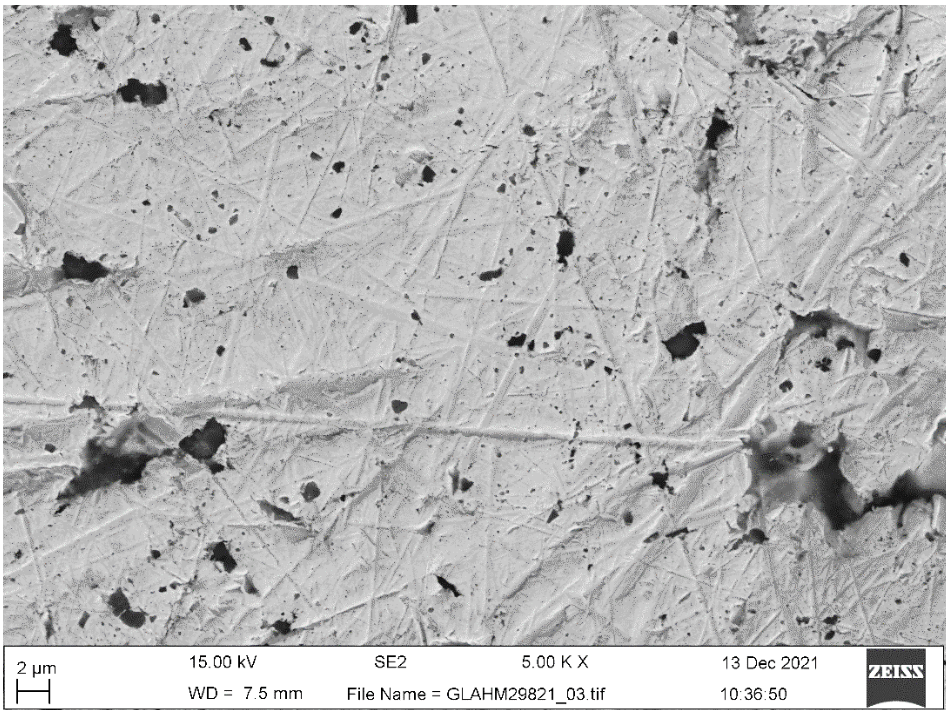

Figure S.3. 85 Area of Roma's head at 5000x magnification

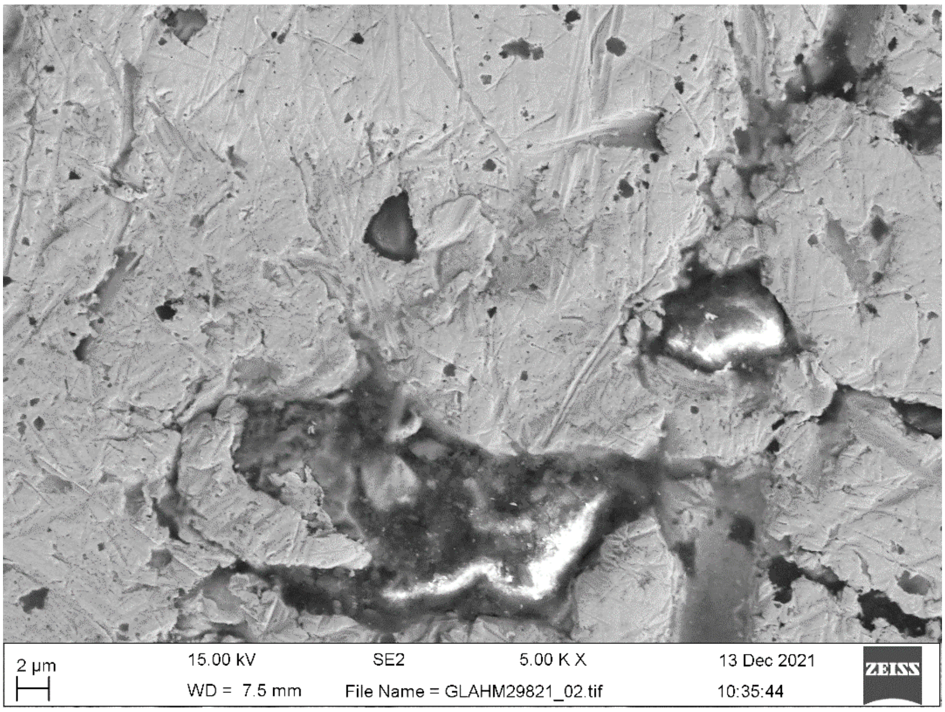

Figure S.3. 86 Second area of Roma's head at 5000x magnification

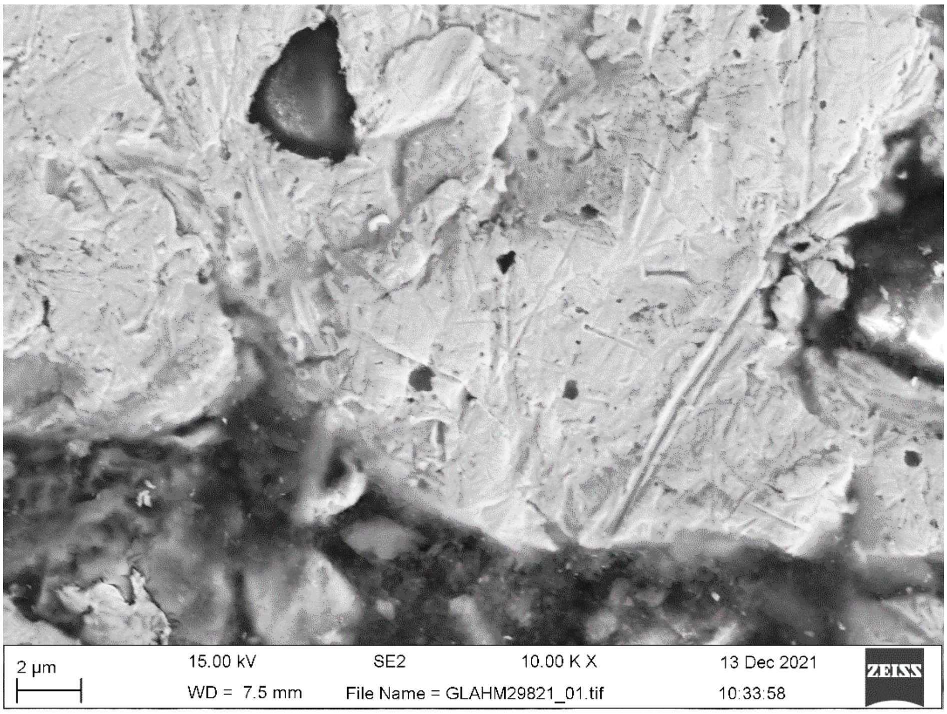

Figure S.3. 87 Area of Roma's head at 10000x magnification

S.3.6 Coin GLAHM:40333 (Questionable Sponsian medallion)

S.3.6.1 Gallery of images

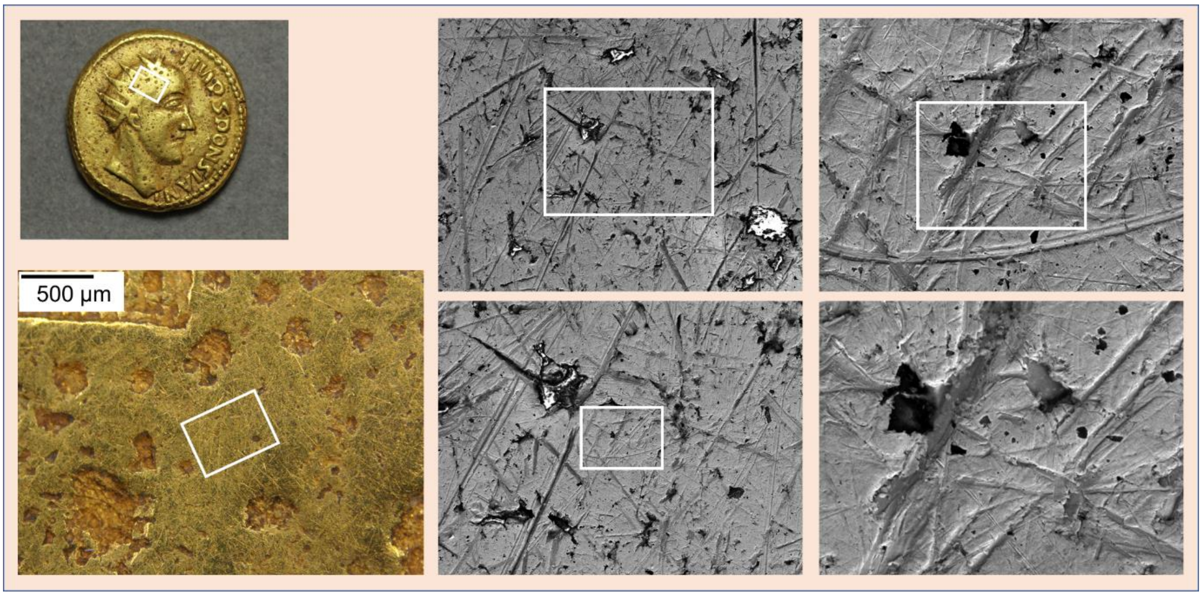

Figure S.3. 88 Key to images

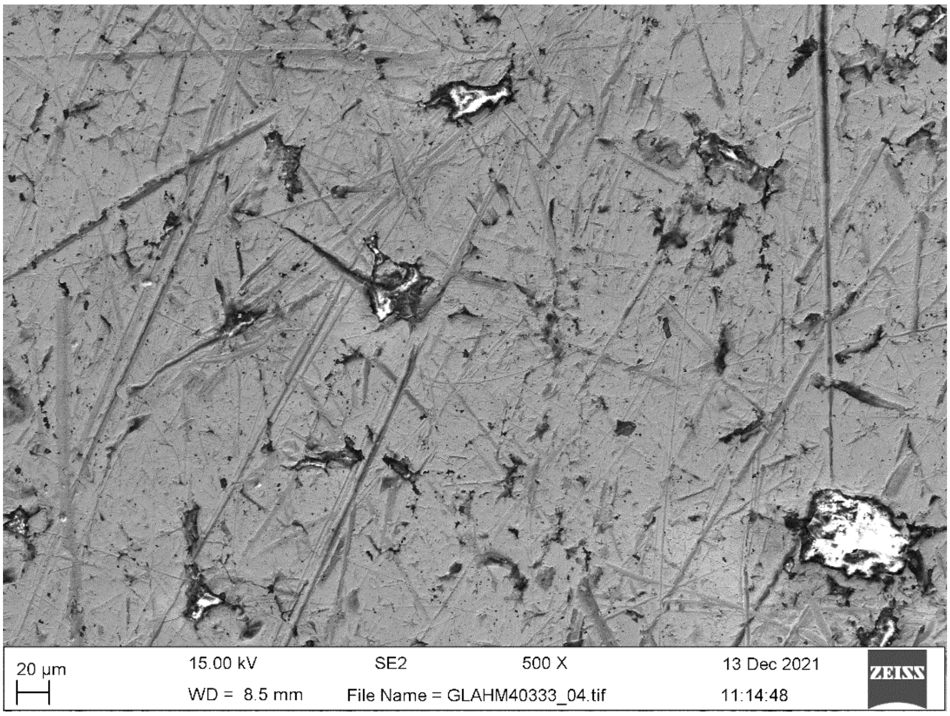

Figure S.3. 89 Area of emperor's head at 500x magnification

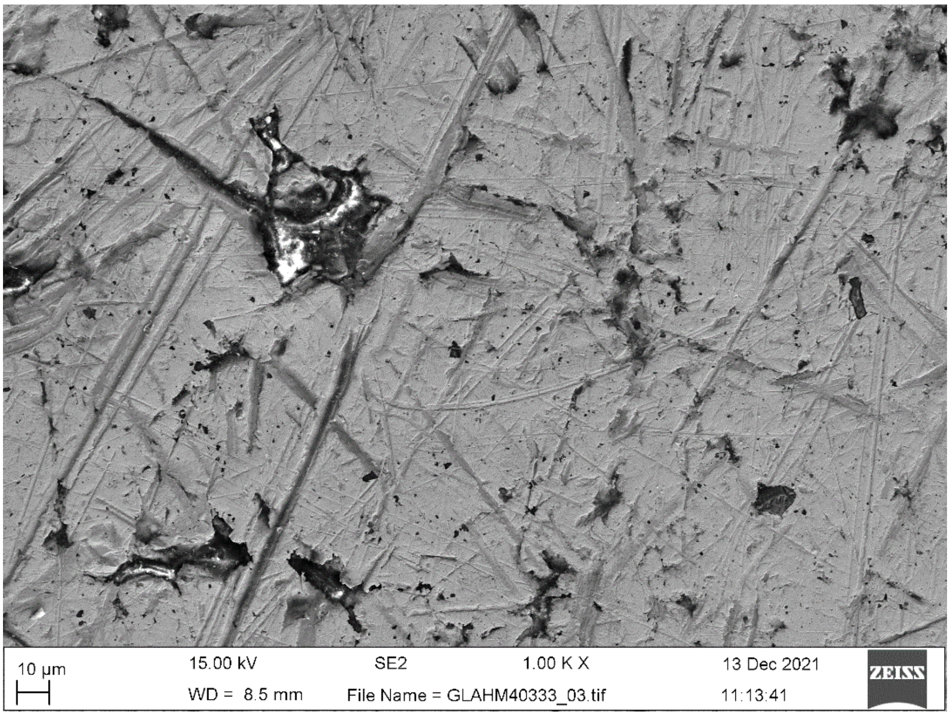

Figure S.3. 90 Area of emperor's head at 1000x magnification

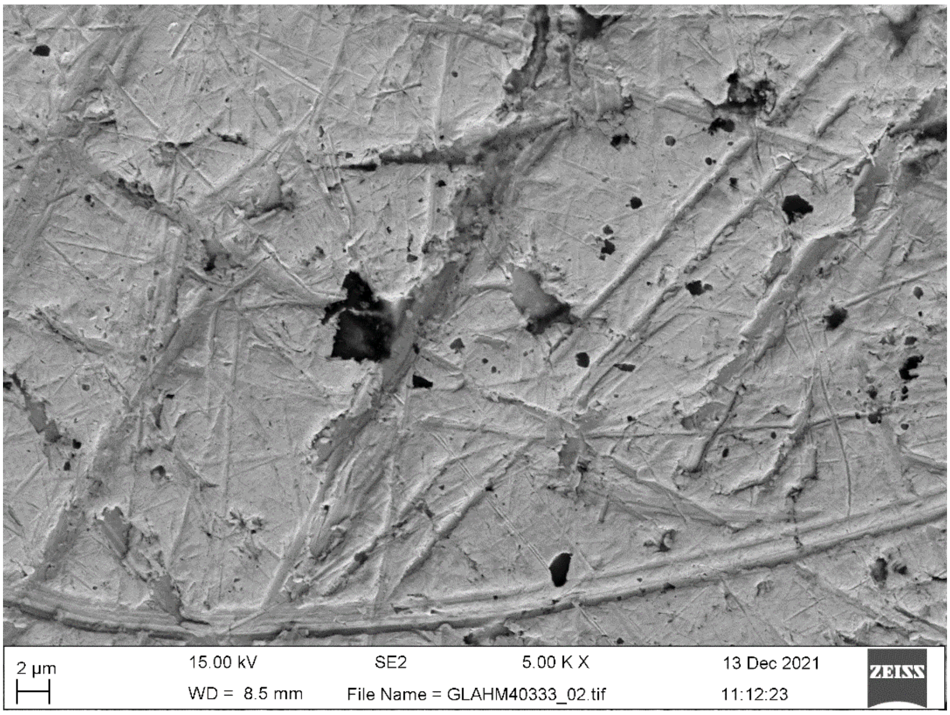

Figure S.3. 91 Area of emperor's head at 5000x magnification

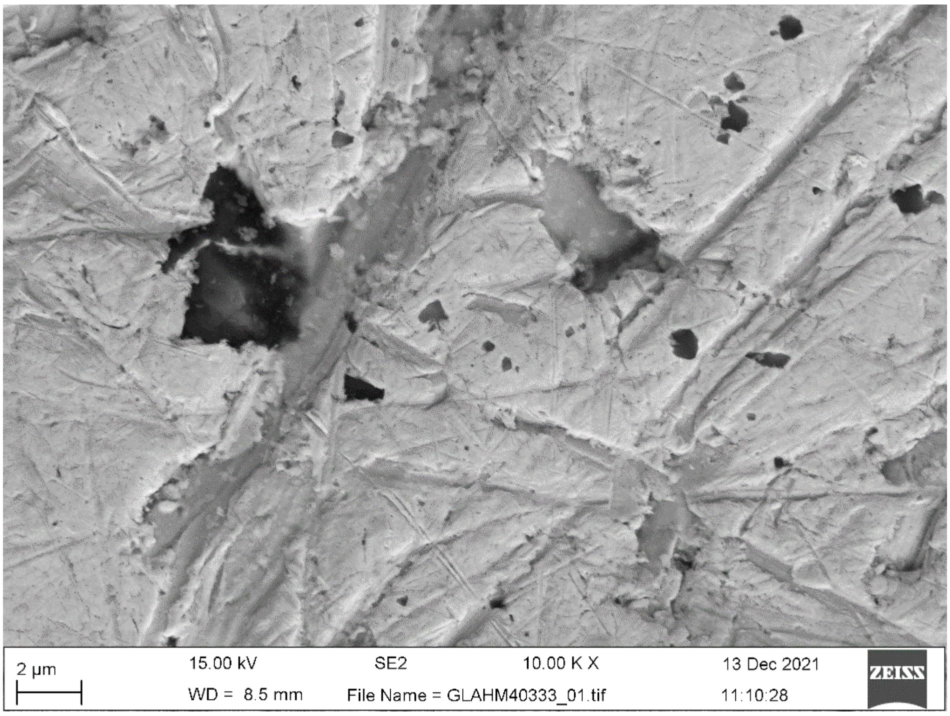

Figure S.3. 92 Area of emperor's head at 10000x magnification

S.3.6.2 Metal composition

Analysis of metal composition was conducted at six locations in two areas of the exposed and worn upper surface on the emperor's head.

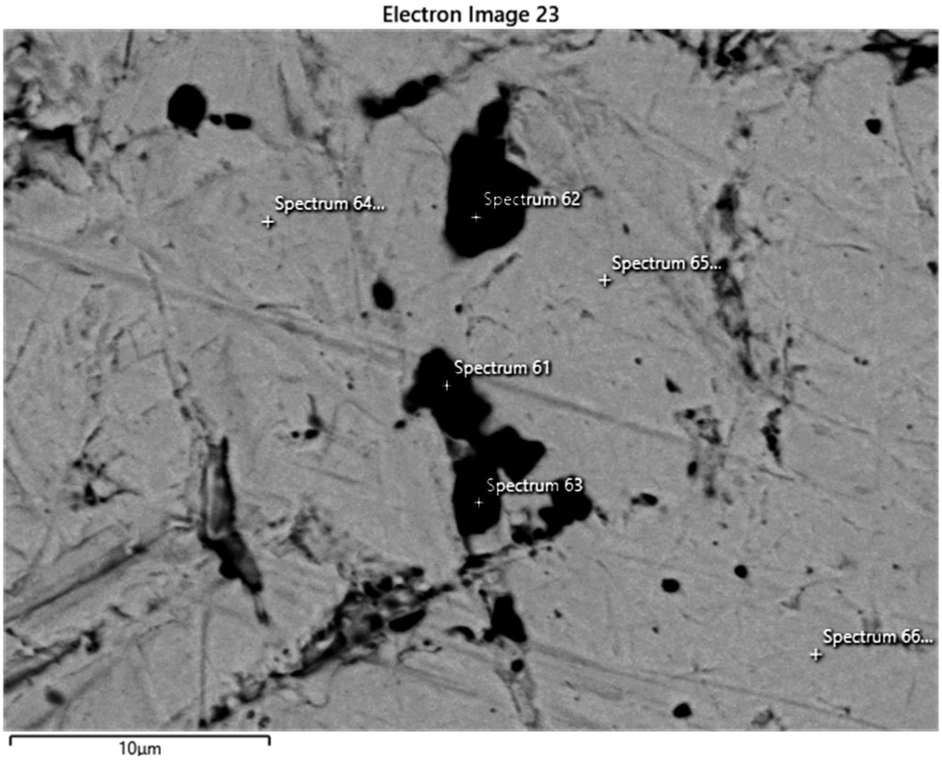

Figure S.3. 93 First area of analyses on worn upper surface of Coin GLAHM:40333

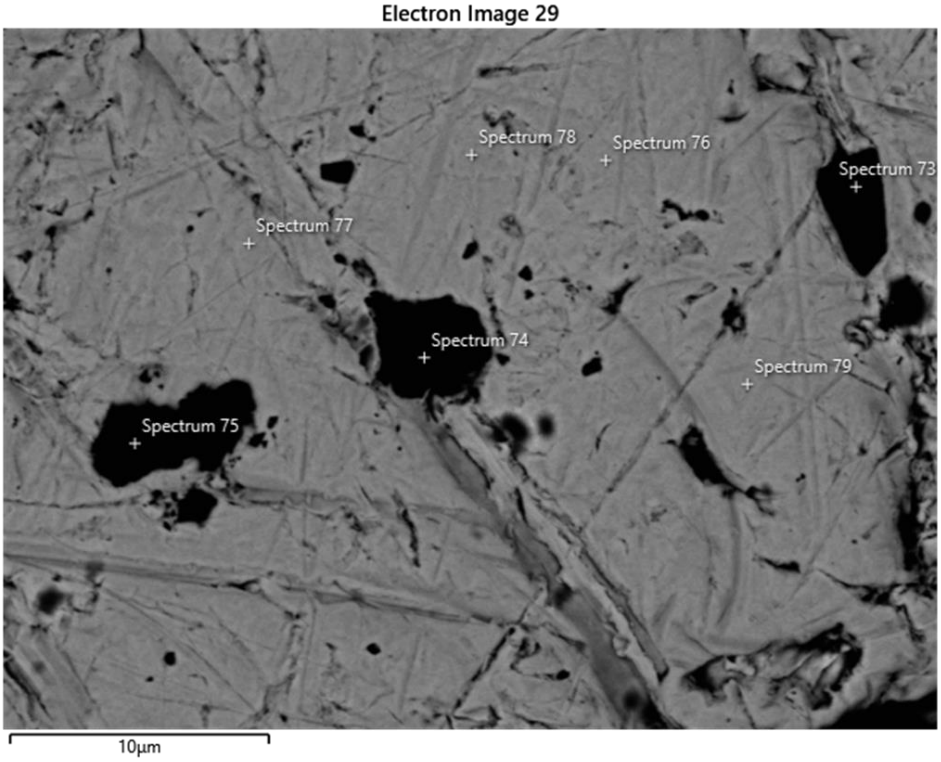

Figure S.3. 94 Second area of analyses on worn upper surface of Coin GLAHM:40333

Spectra indicating metal composition are as follows:

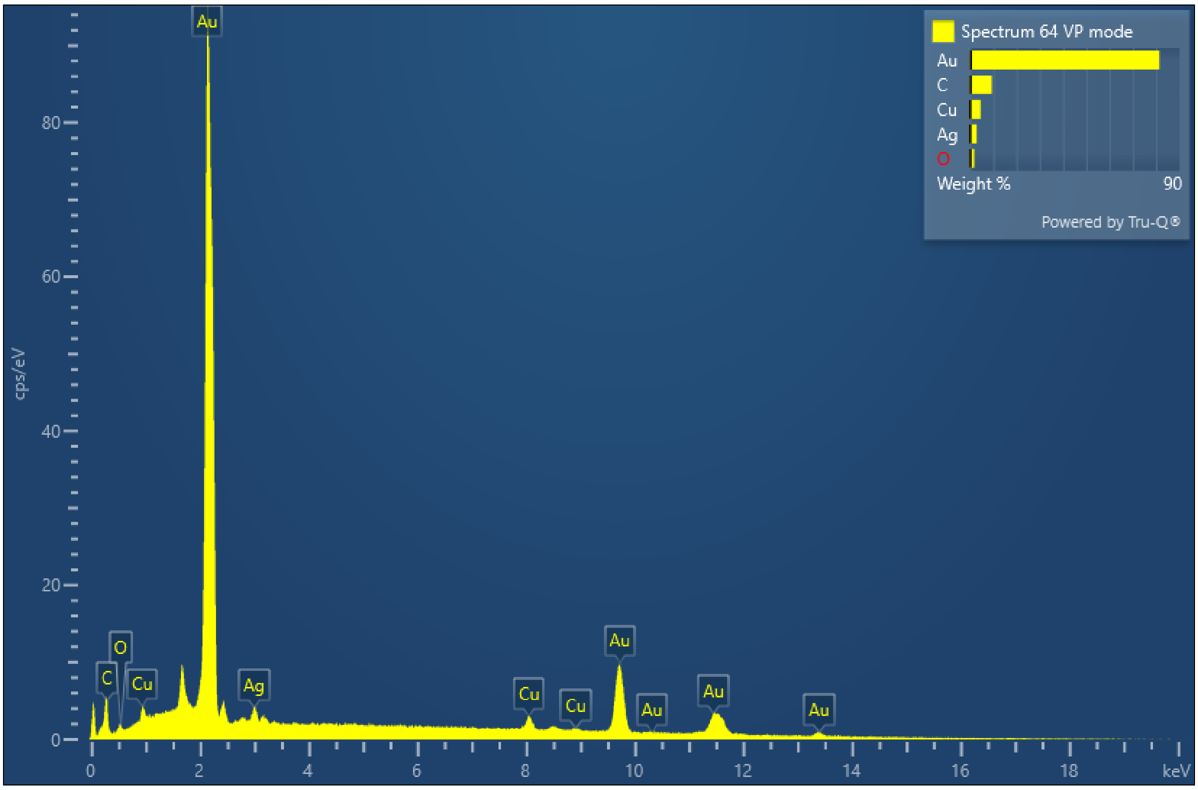

Figure S.3. 95 Spectrum 64 on Coin GLAHM:40333

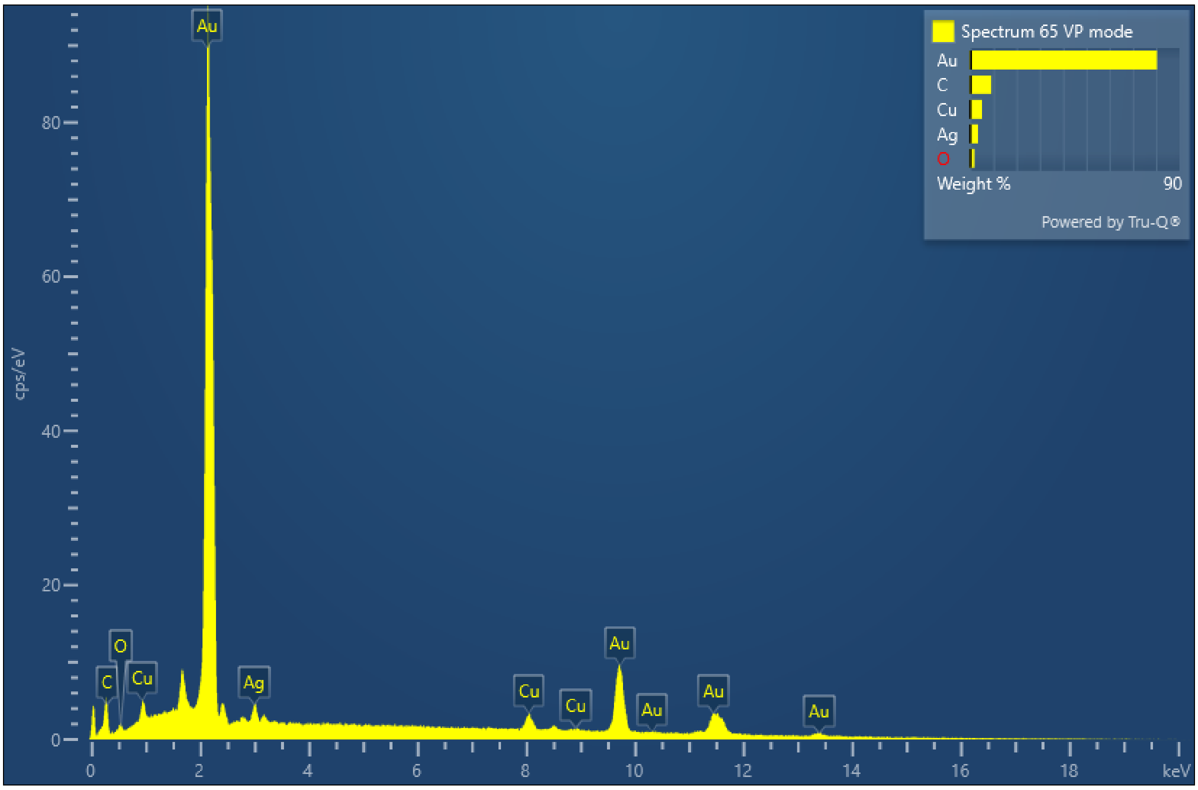

Figure S.3. 96 Spectrum 65 on Coin GLAHM:40333

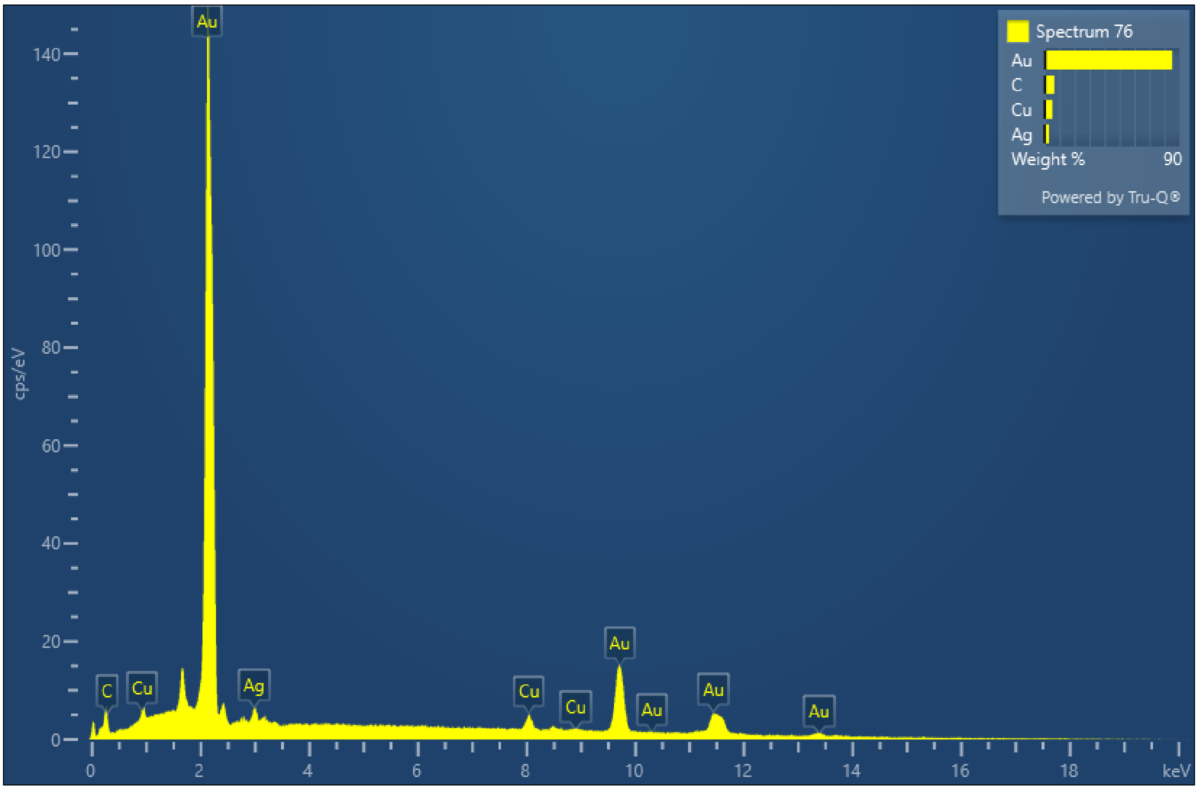

Figure S.3. 97 Spectrum 76 on Coin GLAHM:40333

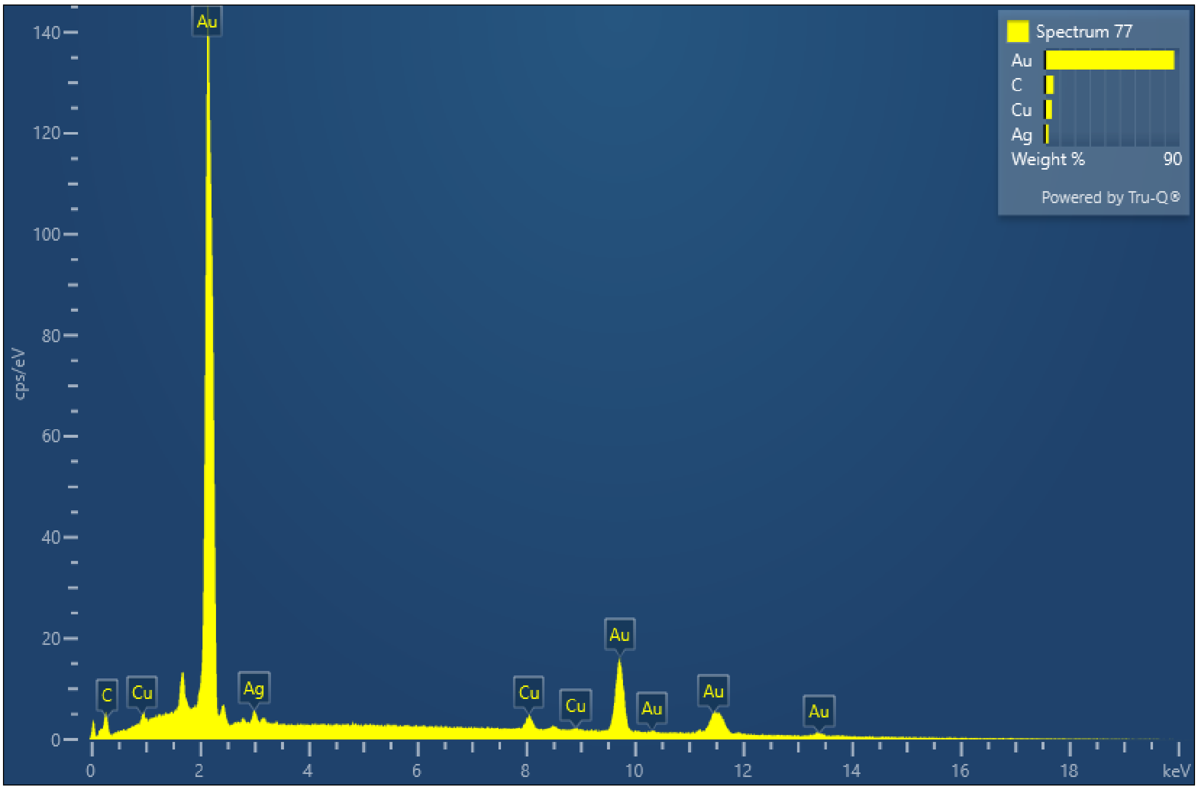

Figure S.3. 98 Spectrum 77 on Coin GLAHM:40333

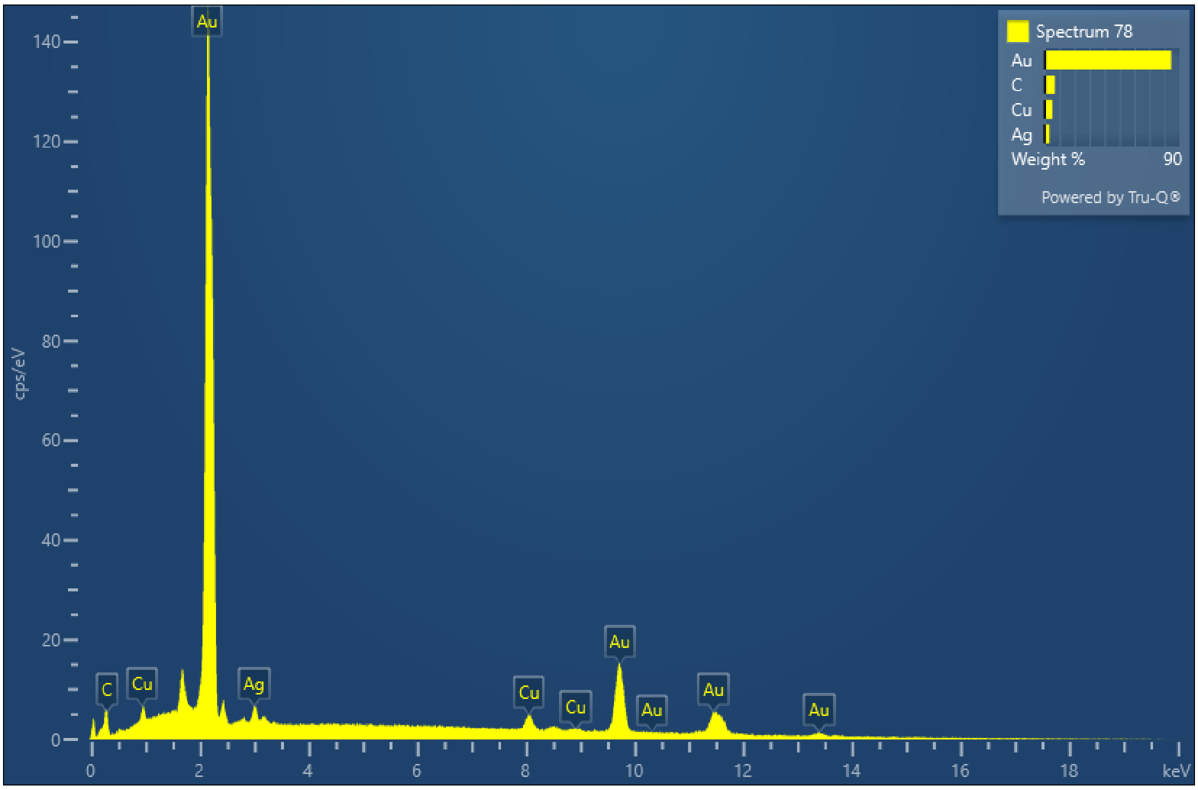

Figure S.3. 99 Spectrum 78 on Coin GLAHM:40333

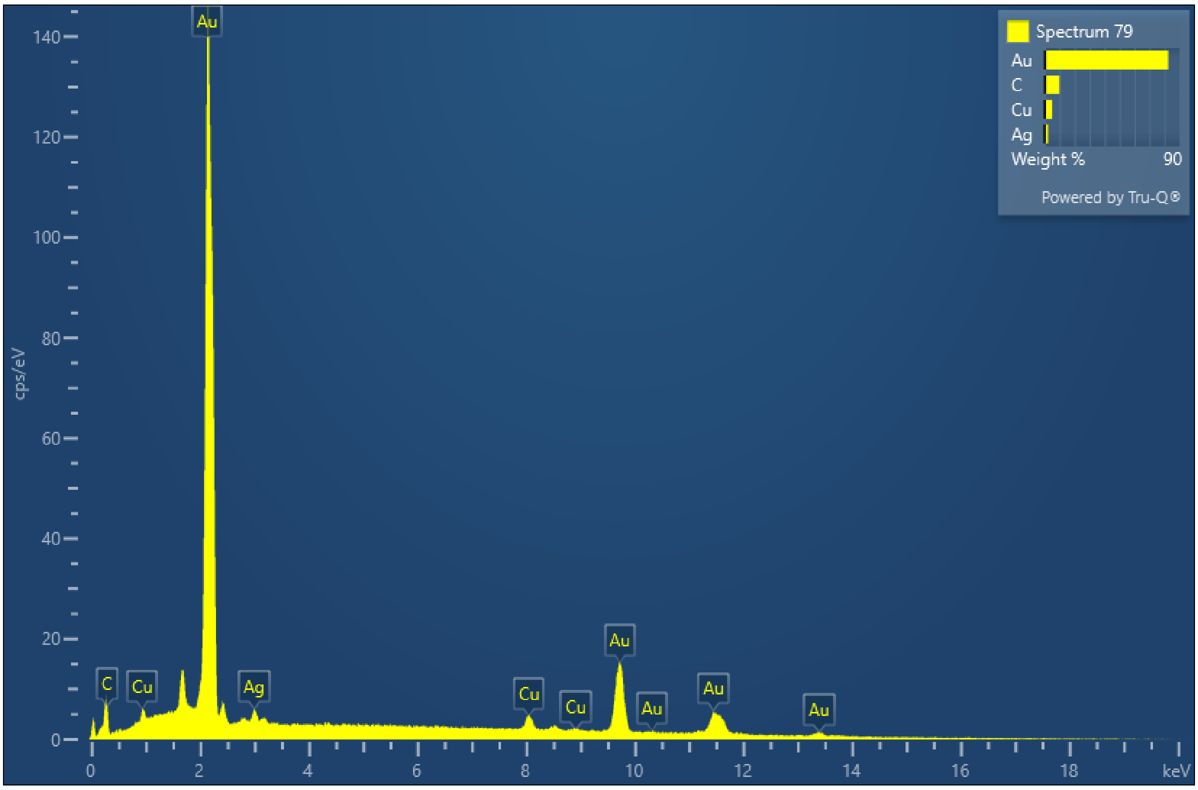

Figure S.3. 100 Spectrum 79 on Coin GLAHM:40333

Discussion: The above spectra are all similar indicating an alloy of about 92% Au, 3.8% Ag and 4.2% Cu (Table S4).

GLAHM 40333

Spectrum Label	Au	Ag	Cu
Spectrum 64 VP mode	92.25	3.83	3.92
Spectrum 65 VP mode	90.86	4.57	4.57
Spectrum 66 VP mode	92.42	3.63	3.95
Spectrum 77	92.78	3.26	3.96
Spectrum 78	91.56	4.08	4.36
Spectrum 79	92.62	3.07	4.31
average	***92.08***	***3.74***	***4.18***
standard dev P	***0.67***	***0.50***	***0.25***
standard dev S	0.73	0.55	0.27

Table S.3. 4 Metal composition of the six spectra obtained from Coin GLAHM:40333

S.3.6.3 Small superficial patches

Several spectra were obtained from amorphous patches that appear dark in SEM (Spectra 61, 62, 63, 73, 74, and 75) as follows:

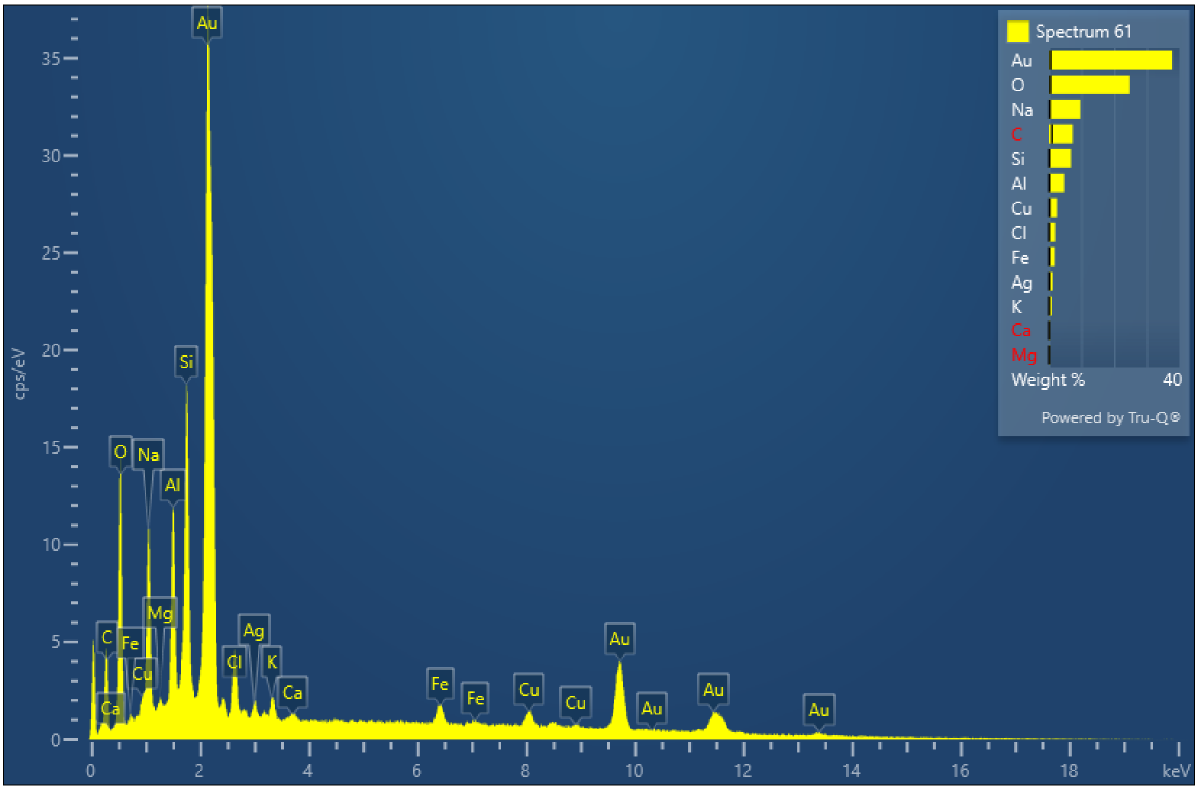

Figure S.3. 101 Spectrum 61 on Coin GLAHM:40333

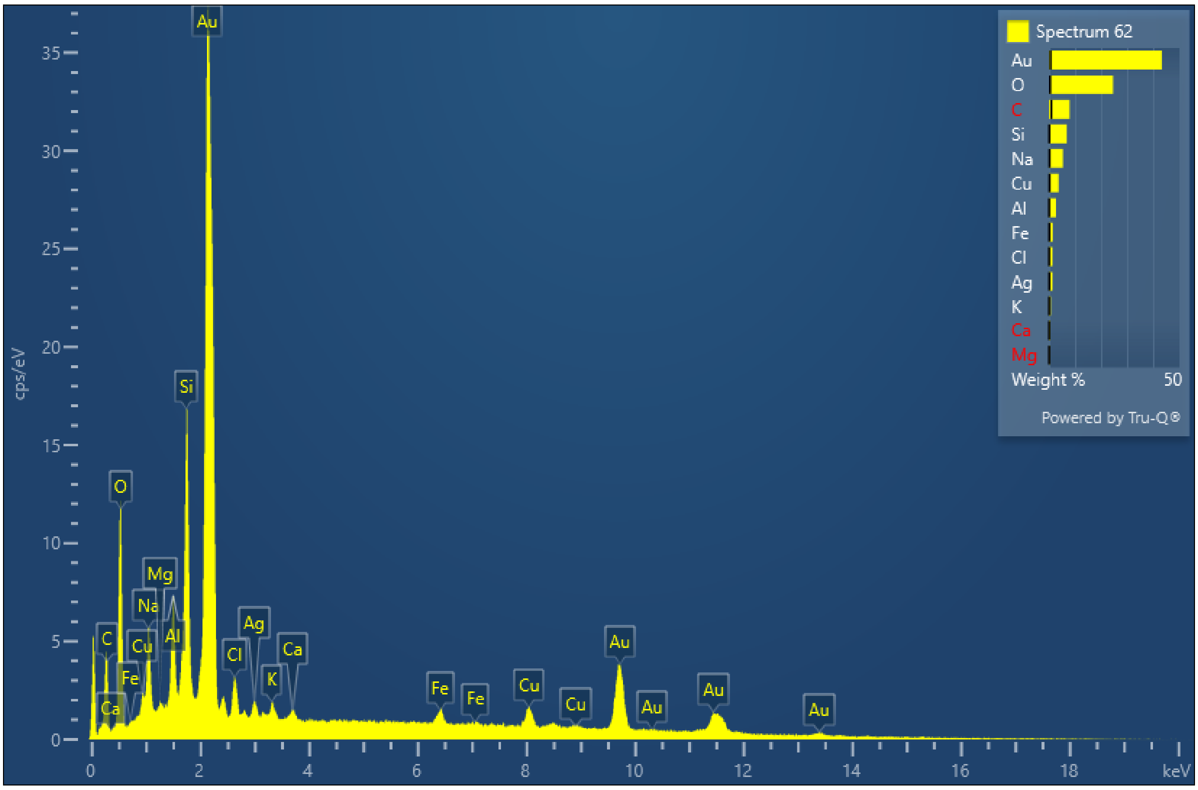

Figure S.3. 102 Spectrum 62 on Coin GLAHM:40333

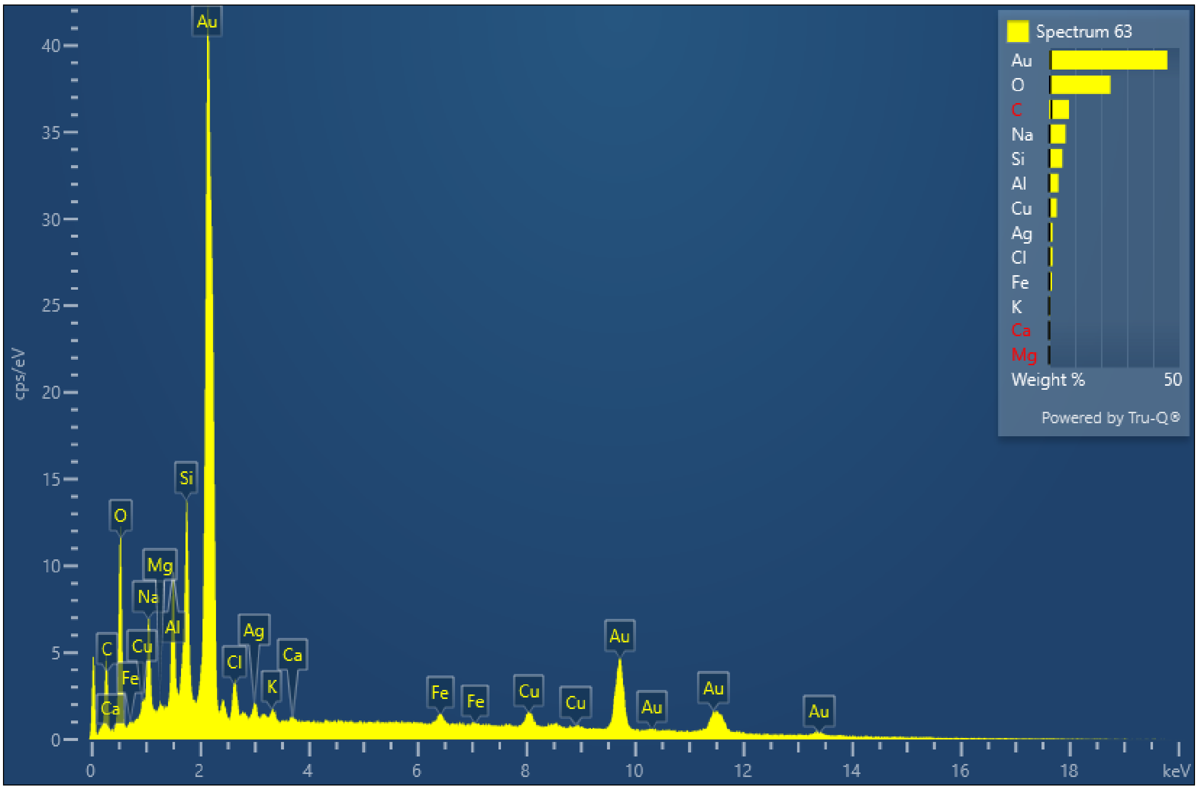

Figure S.3. 103 Spectrum 63 on Coin GLAHM:40333

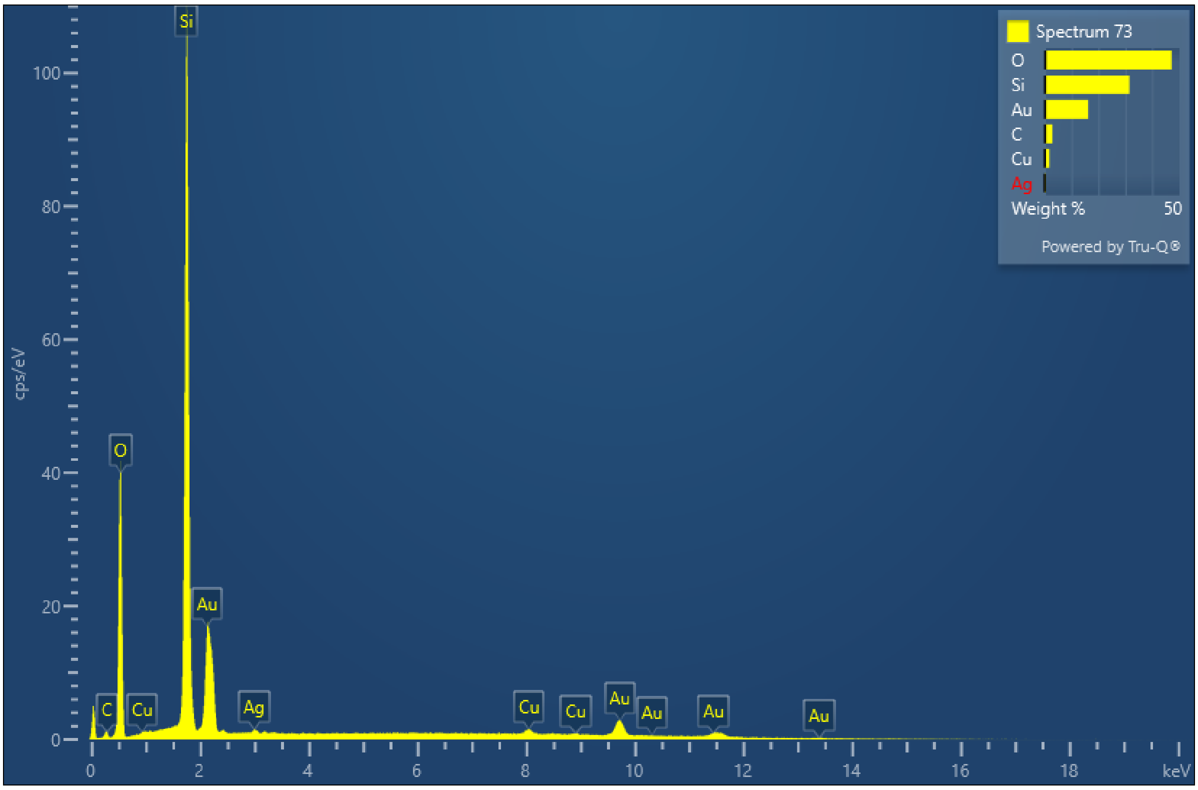

Figure S.3. 104 Spectrum 73 on Coin GLAHM:40333

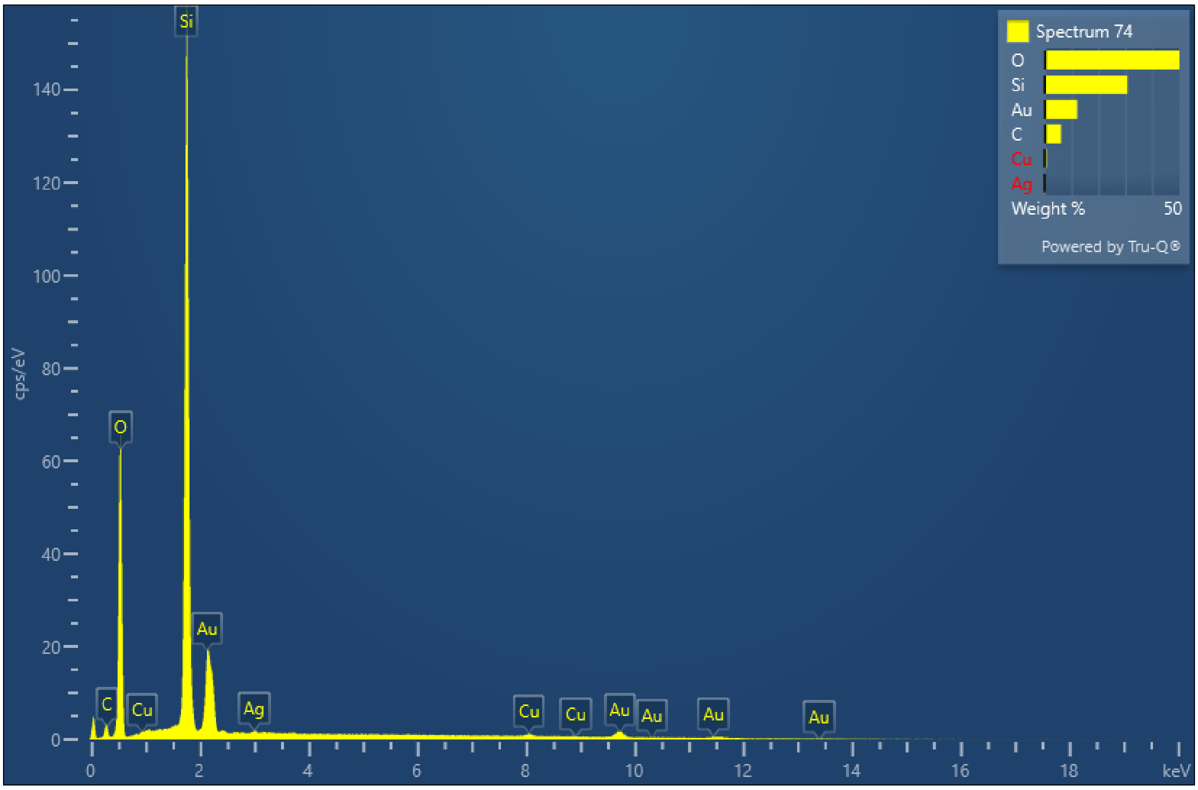

Figure S.3. 105 Spectrum 74 on Coin GLAHM:40333

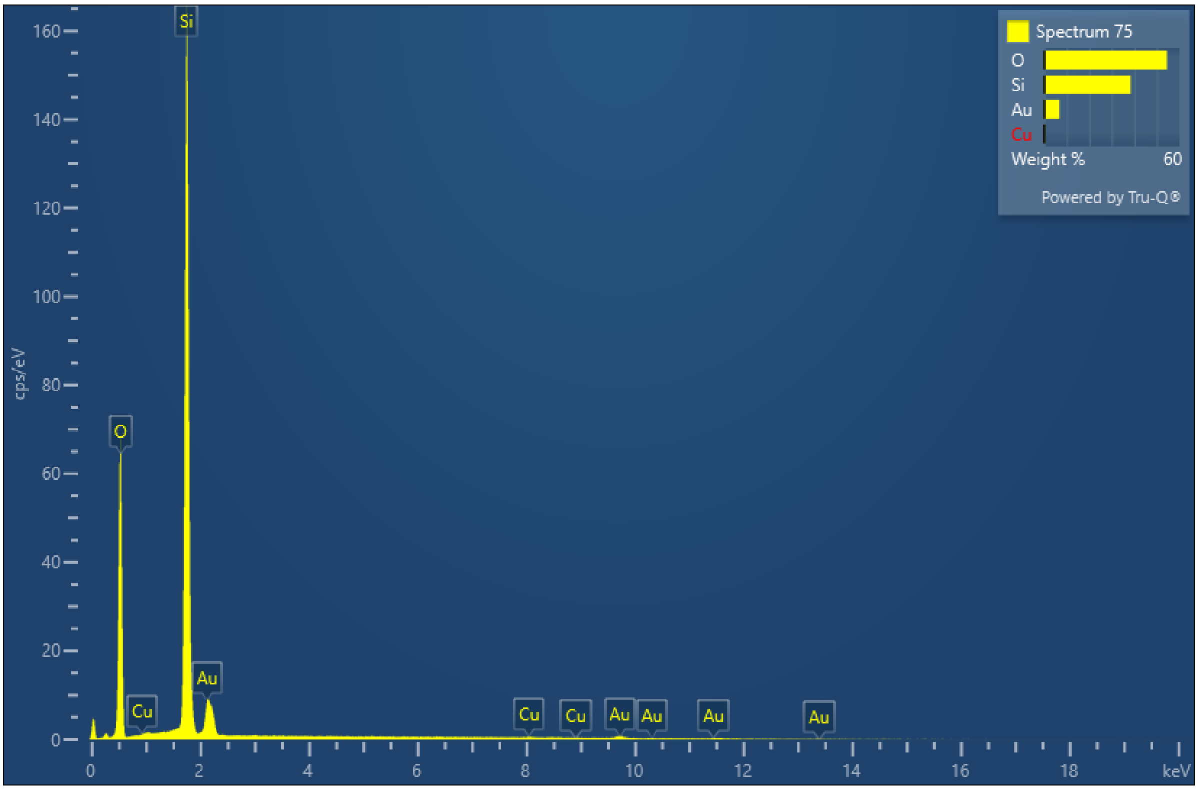

Figure S.3. 106 Spectrum 75 on Coin GLAHM:40333

Discussion: The above six spectra fall into two groups depending on the area analysed. Spectra 73-75 show peaks associated with Si and O and very minor subsidiary peaks associated with the metal composition. These are interpreted as patches of amorphous opaline silica cement. Spectra 61-63 have a much stronger signal arising from the metal with subsidiary Si and O peaks as well as minor but significant Na, Al, Fe and Cl. The deposits in this area are apparently thinner than elsewhere and have a significant admixture of other compounds.

GLAHM 40333

Spectrum Label	O	Si
Spectrum 73	52.96	47.04
Spectrum 74	54.32	45.68
Spectrum 75	53.95	46.05
average	***53.74***	***46.26***
standard dev P	***0.57***	***0.57***
standard dev S	0.70	0.70

Table S.3. 5 Composition of the three spectra obtained from small black spots on Coin GLAHM:40333

S.3.6.4 Larger earthen deposits

Analyses were conducted on an area of apparent earthen deposit between the letters ‘M’ and ‘P’ of the obverse legend (Figure S70). The area appears largely amorphous with discrete areas that were anomalously bright or dark in SEM. Spectrum 55 was taken from an area of ‘groundmass’ and Spectra 56-60 on discrete areas of varying appearance and brightness towards the upper centre part of the ‘earthen deposit’(Figure S71).

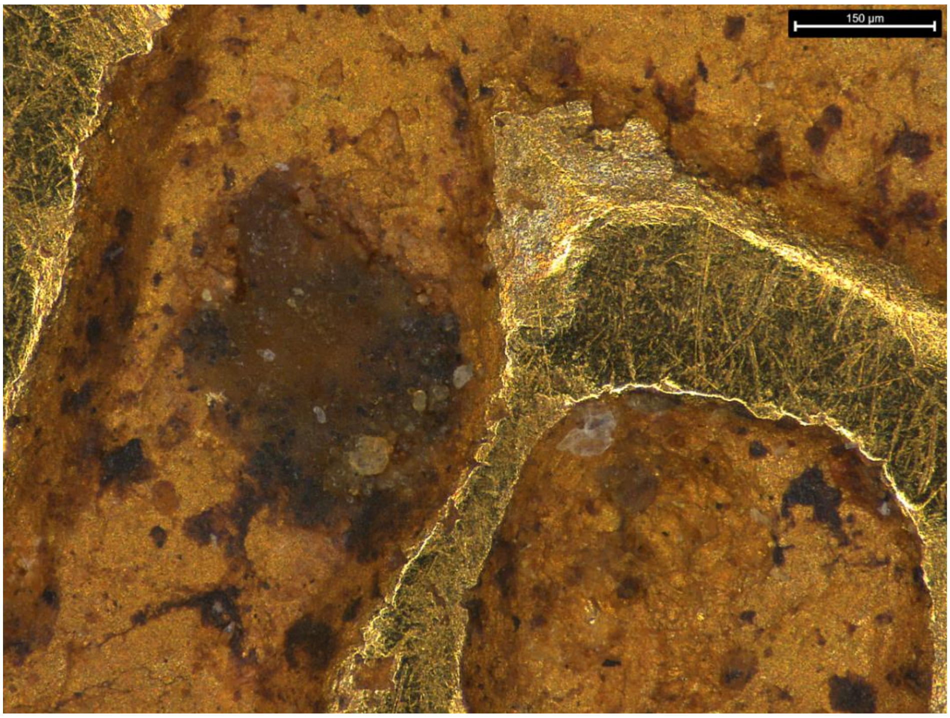

Figure S.3. 107 Area of analyses of possible earthen deposits on Coin GLAHM:40333

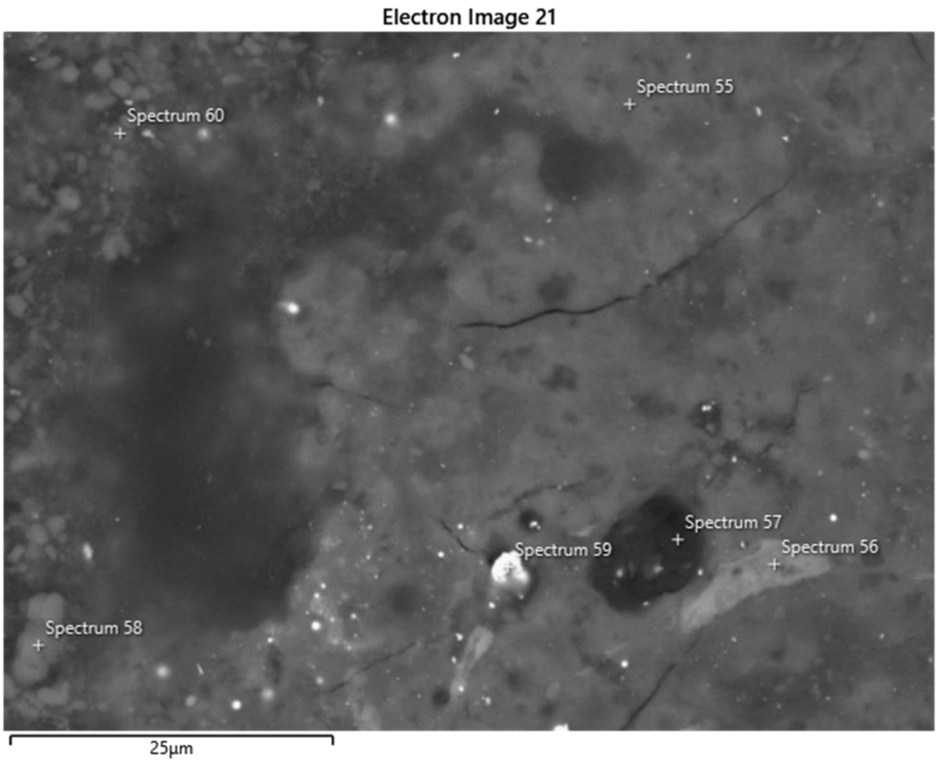

Figure S.3. 108 Areas of Spectra 55 to 60 on Coin GLAHM:40333

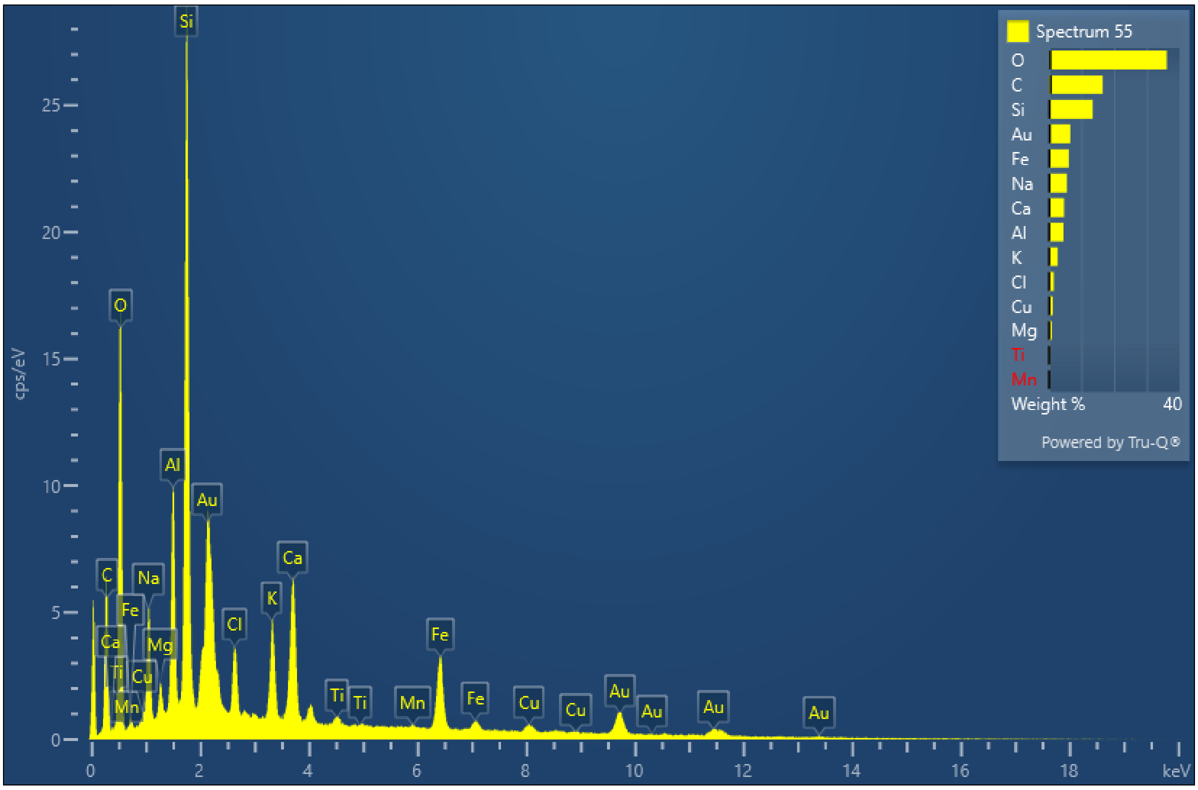

Figure S.3. 109 Spectrum 55 on Coin GLAHM:40333

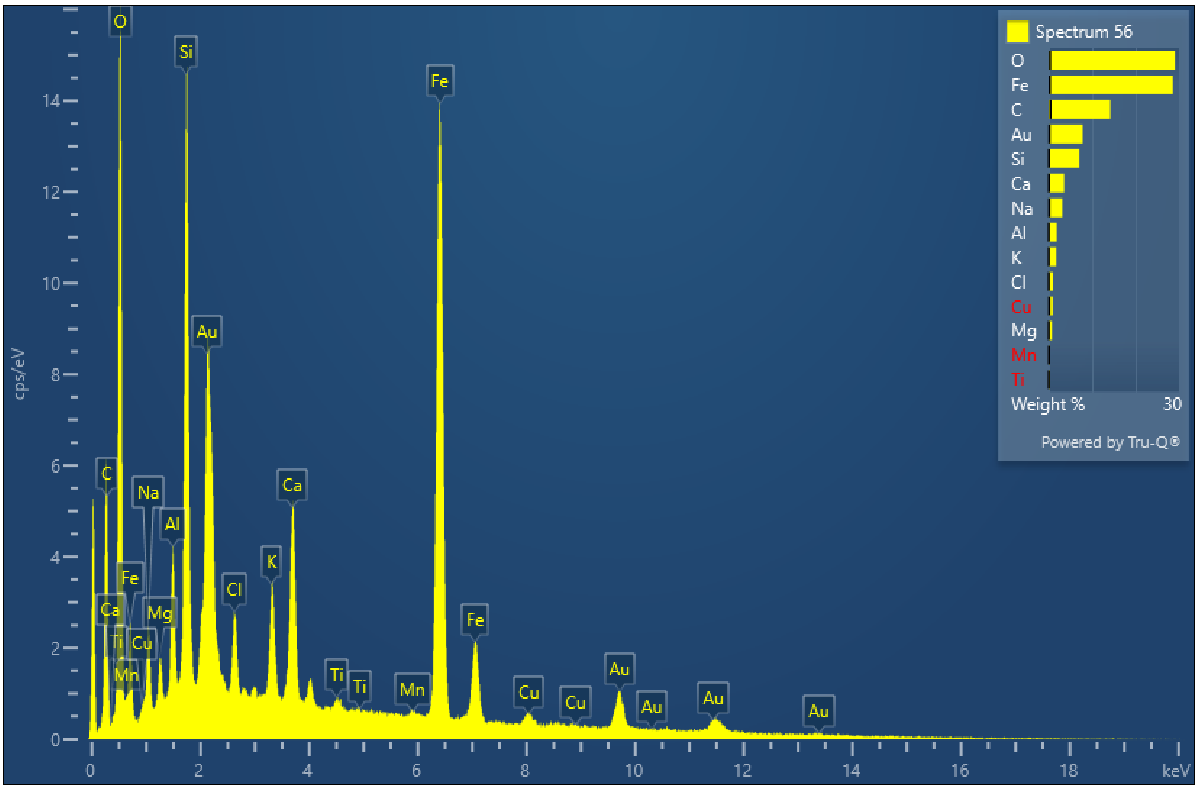

Figure S.3. 110 Spectrum 56 on Coin GLAHM:40333

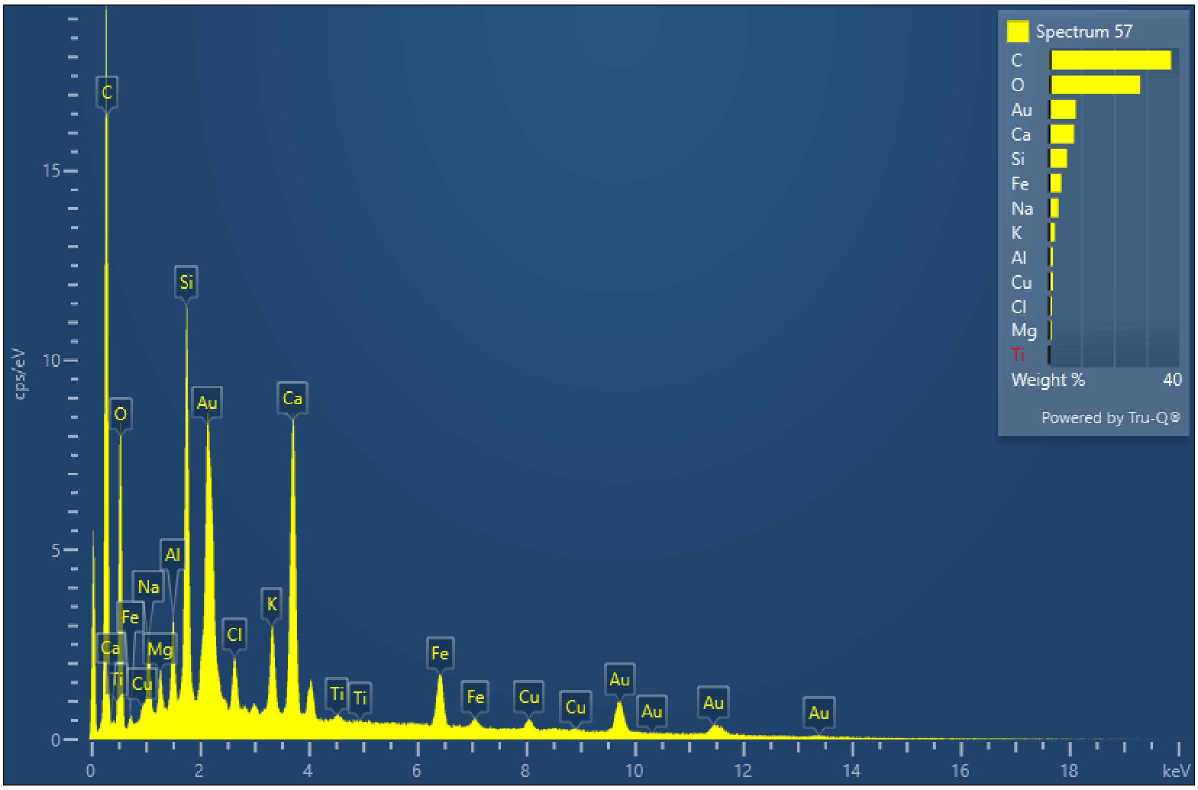

Figure S.3. 111 Spectrum 57 on Coin GLAHM:40333

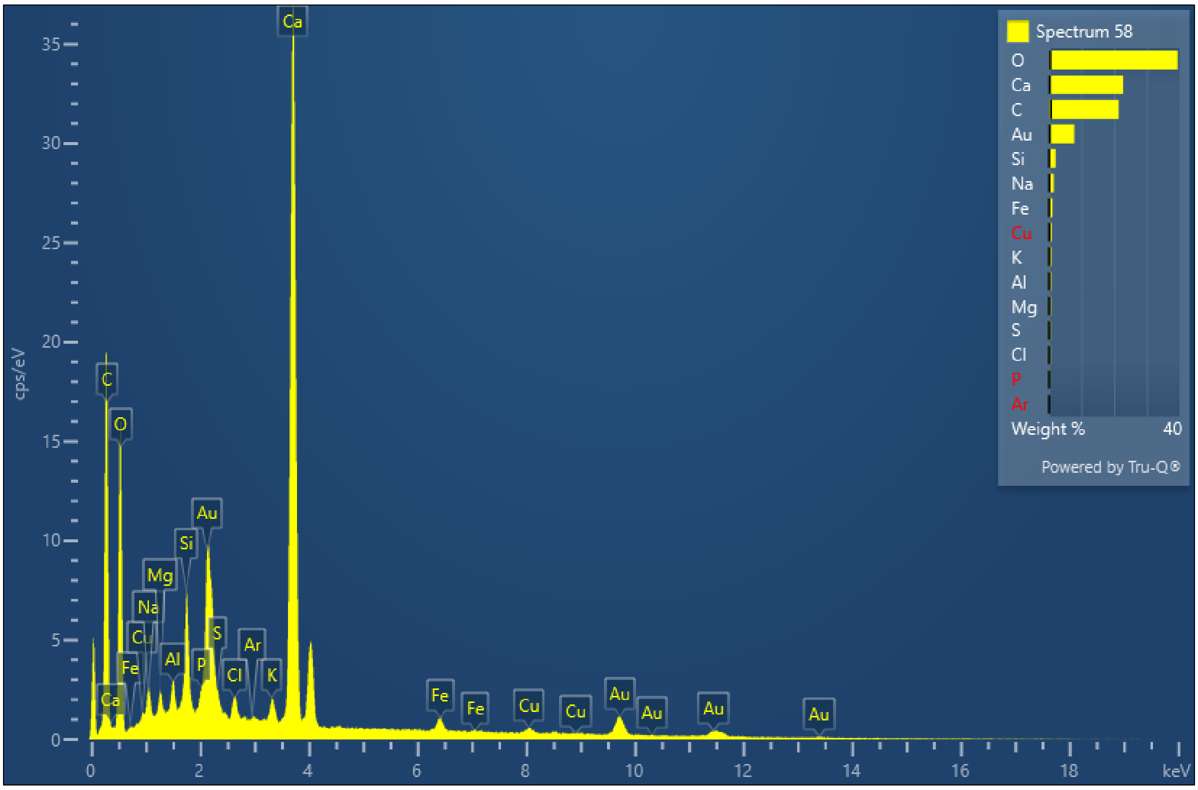

Figure S.3. 112 Spectrum 58 on Coin GLAHM:40333

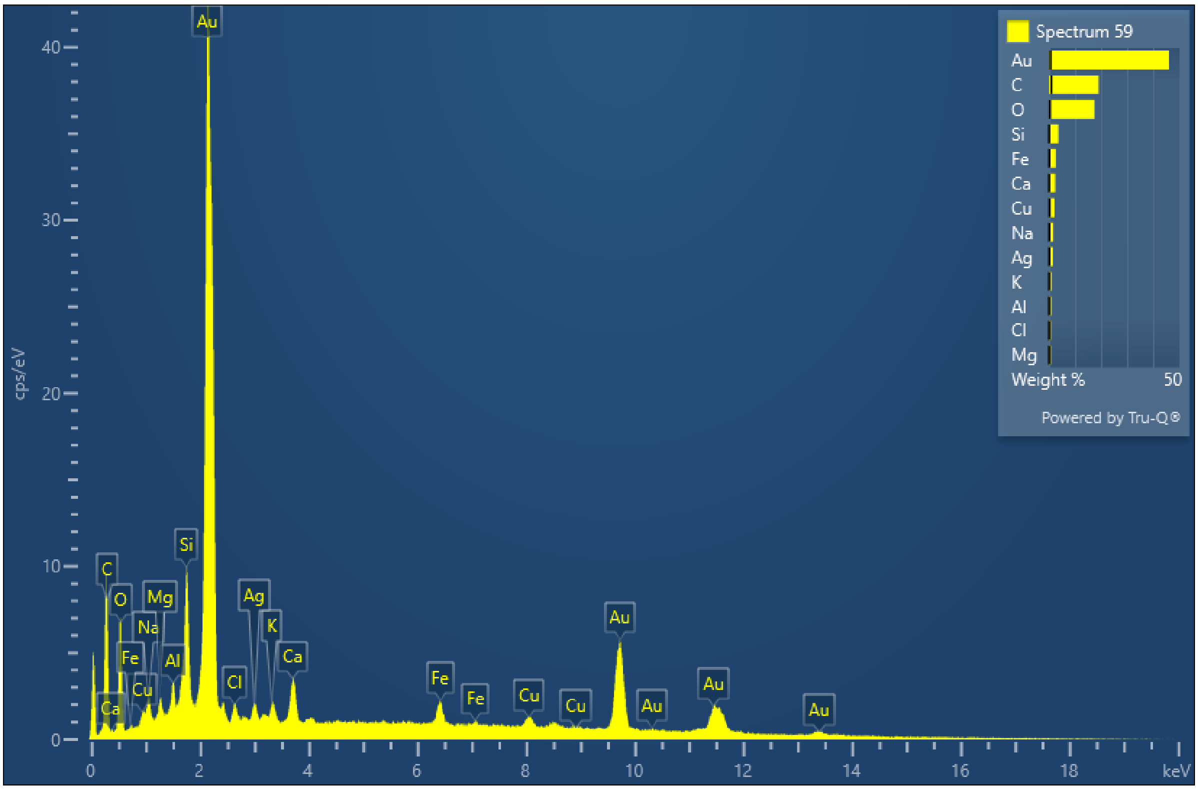

Figure S.3. 113 Spectrum 59 on Coin GLAHM:40333

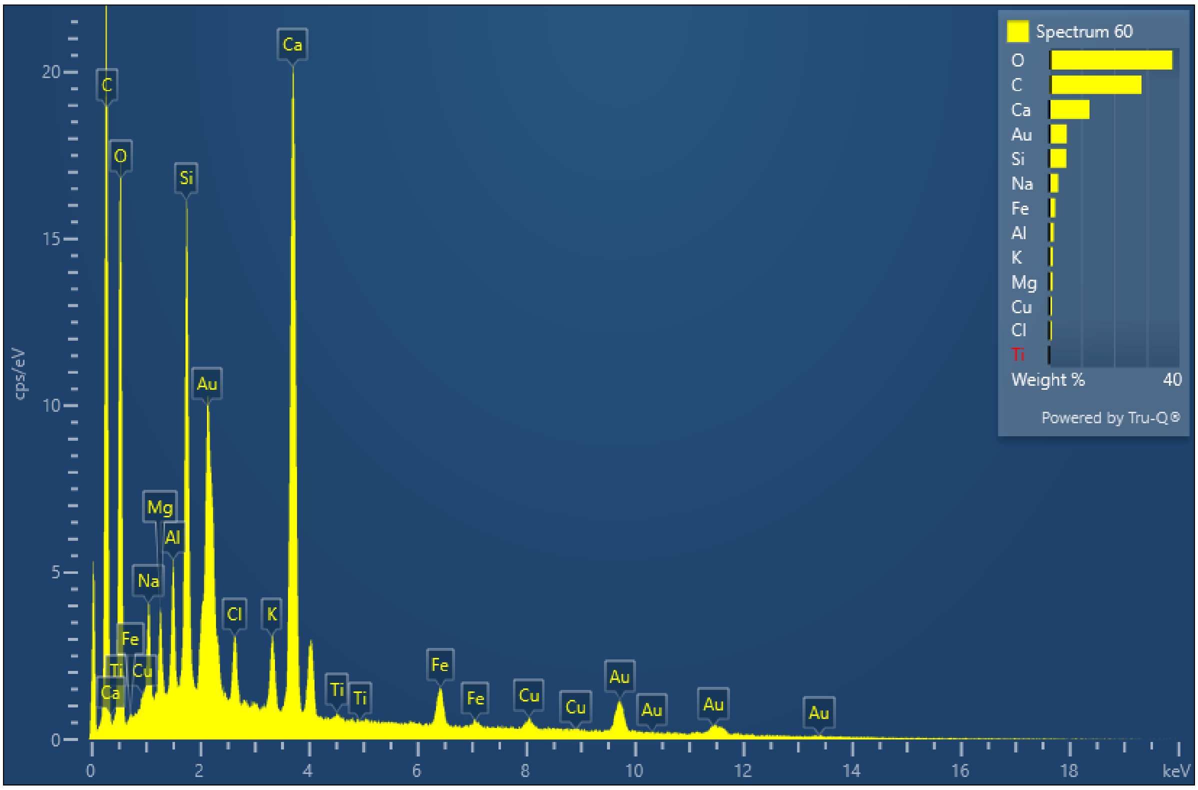

Figure S.3. 114 Spectrum 60 on Coin GLAHM:40333

Discussion: The interpretation of these spectra is as follows. The ‘groundmass’ area (Spectrum 55) has prominent peaks associated with Si and O and a wide range of other elements. It is interpreted as opaline silica cement with an admixture of other compounds that cannot be individually resolved. Spectrum 56 was taken on an anomalously white area. It has strong peaks associated with Fe and may contain siderite ($FeCO_3$) with other minerals with Na, Ca, Si, Mg, and K. Spectrum 57 on an anomalously dark area has a higher concentration of Ca and with Si along with Fe, Na and K and may be a calcic feldspar with other materials. Spectrum 58 on an irregularly shaped mass of bright material has an even higher Ca content and may be calcite ($CaCO_3$). Spectrum 59 on particularly bright flake is gold spalled off the surface of the coin. Several other such patches appear to be mixed in to the groundmass of the deposit (Figure S.3.79). Spectrum 60 on a bright area is similar to Spectrum 58 except it has a higher Si peak.

We finally turned our attention to an area with an equant blocky crystal ~25 µm long (Spectra 67 and 68) beside irregular clumps of amorphous material (Spectra 69 and 70) (Figure S.3.86).

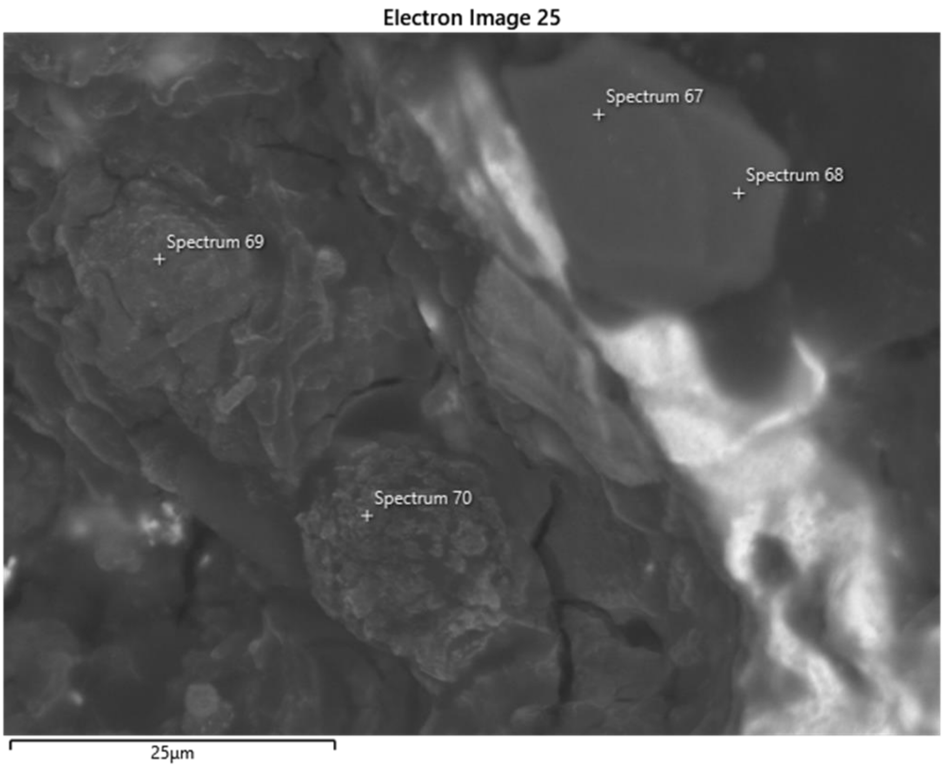

Figure S.3. 115 Area analysed on Coin GLAHM:40333

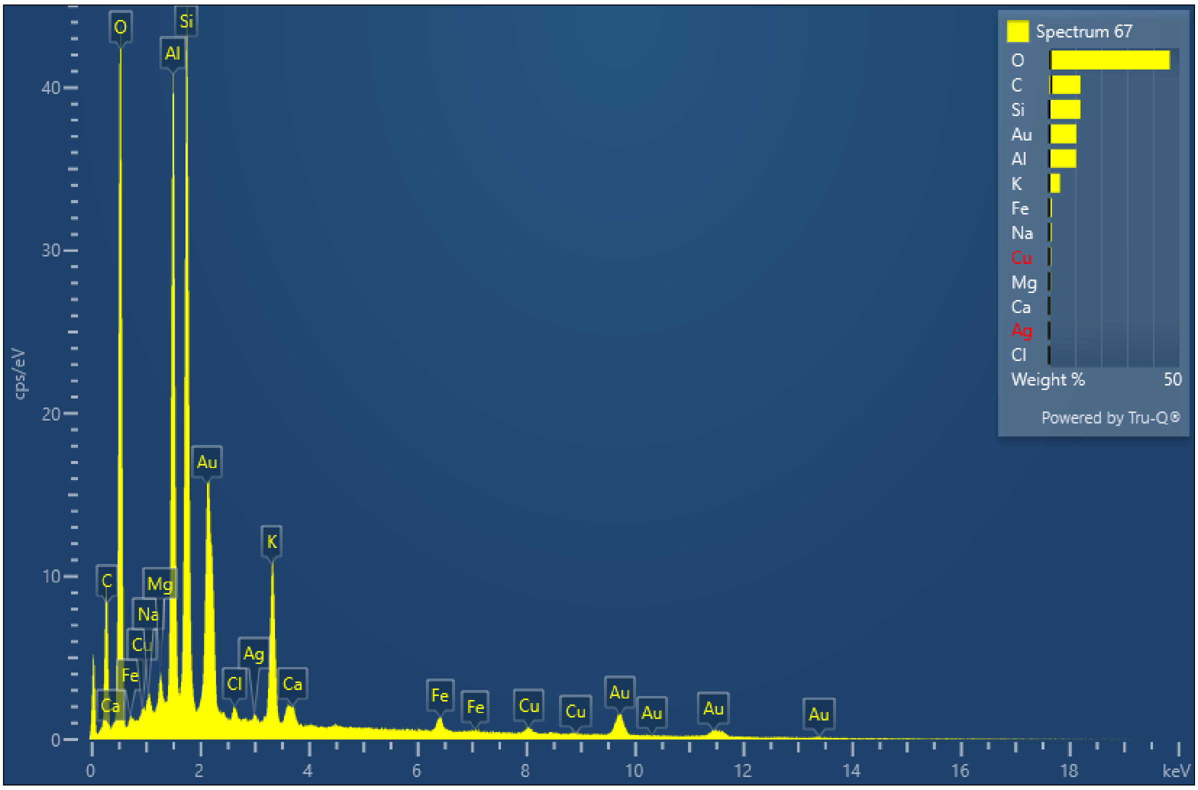

Figure S.3. 116 Spectrum 67 on Coin GLAHM:40333

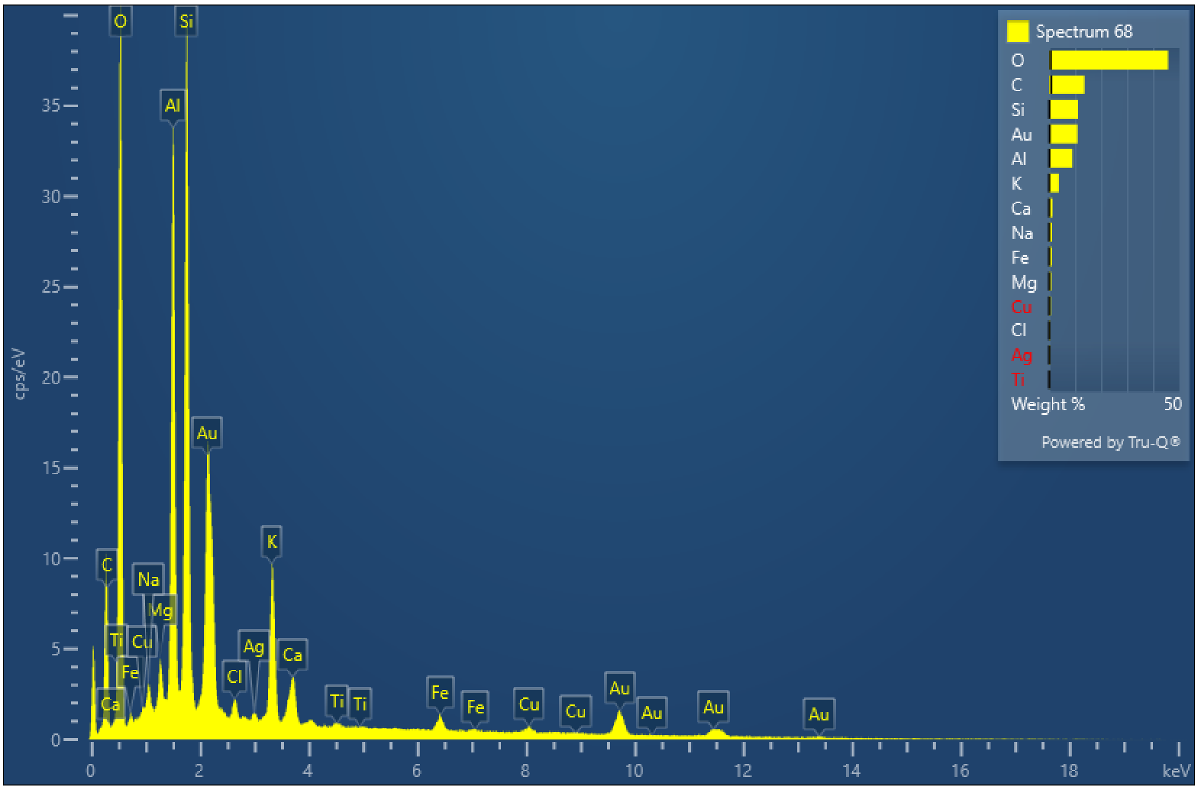

Figure S.3. 117 Spectrum 68 on Coin GLAHM:40333

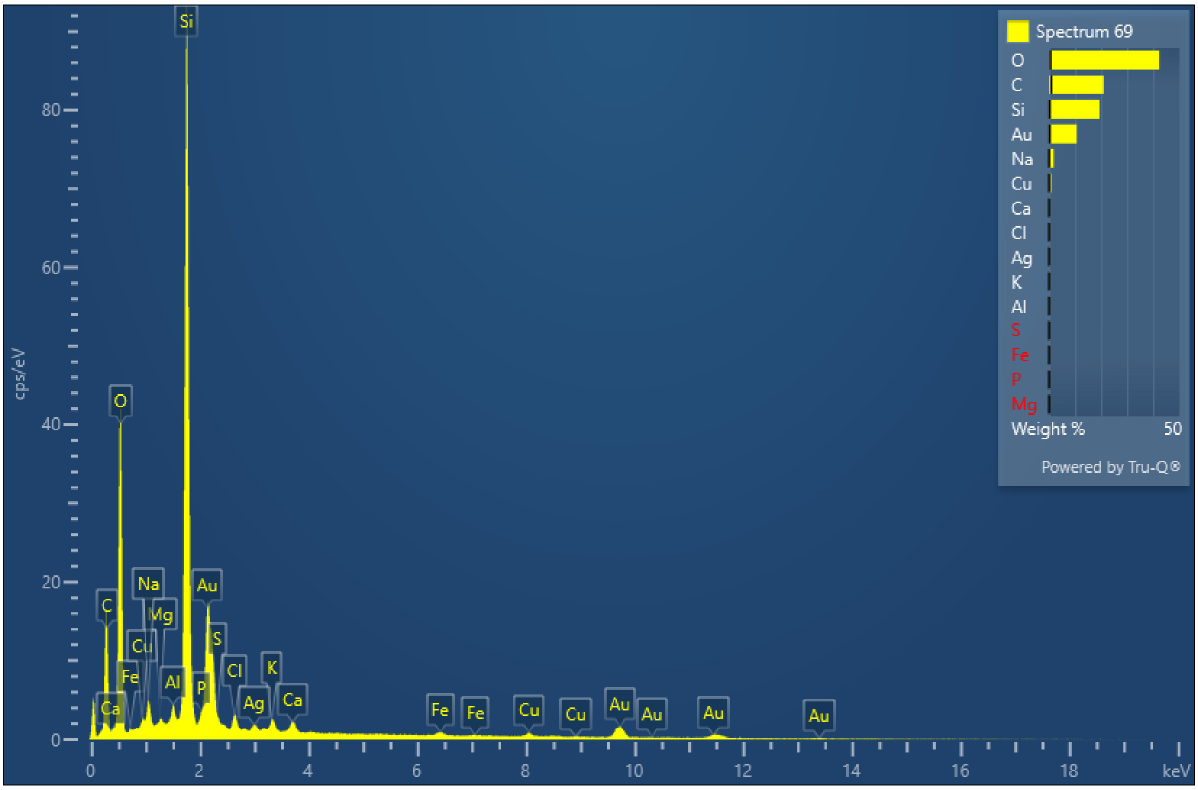

Figure S.3. 118 Spectrum 69 on Coin GLAHM:40333

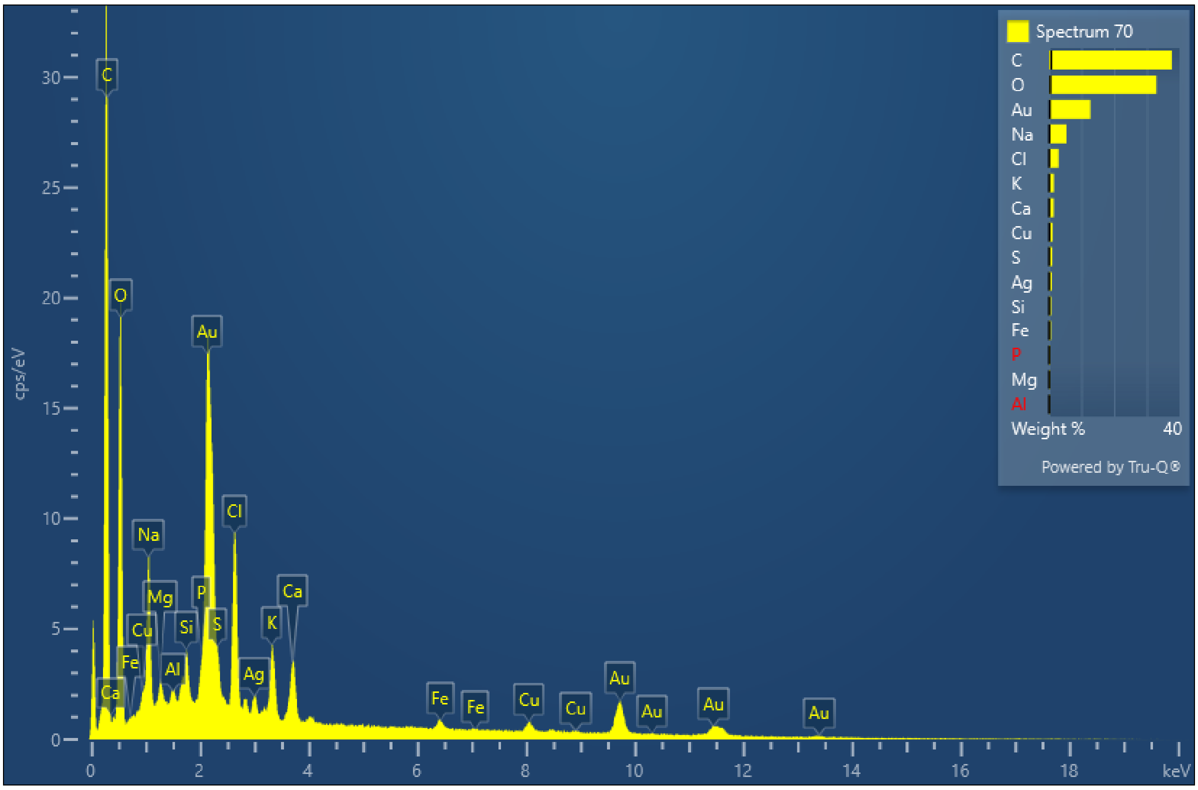

Figure S.3. 119 Spectrum 70 on Coin GLAHM:40333

Discussion: Spectra 67 and 68 are very similar to one another with prominent peaks associated with Si, O, Al and K with minor Ca and Na. This composition, with the equant habit, suggests a potassium feldspar ($KAlSi_3O_8$). Spectra 69 and 70 are surprisingly different to one another considering their proximity and similar appearance. Spectrum 69 indicates amorphous silica with other components whereas Spectrum 70 has peaks associated with Na, Cl and K which may indicate salt, (Na, K)Cl.

We also took two spectra (71 and 72) from a flaky substance beside the feldspar crystal (Figure S83).

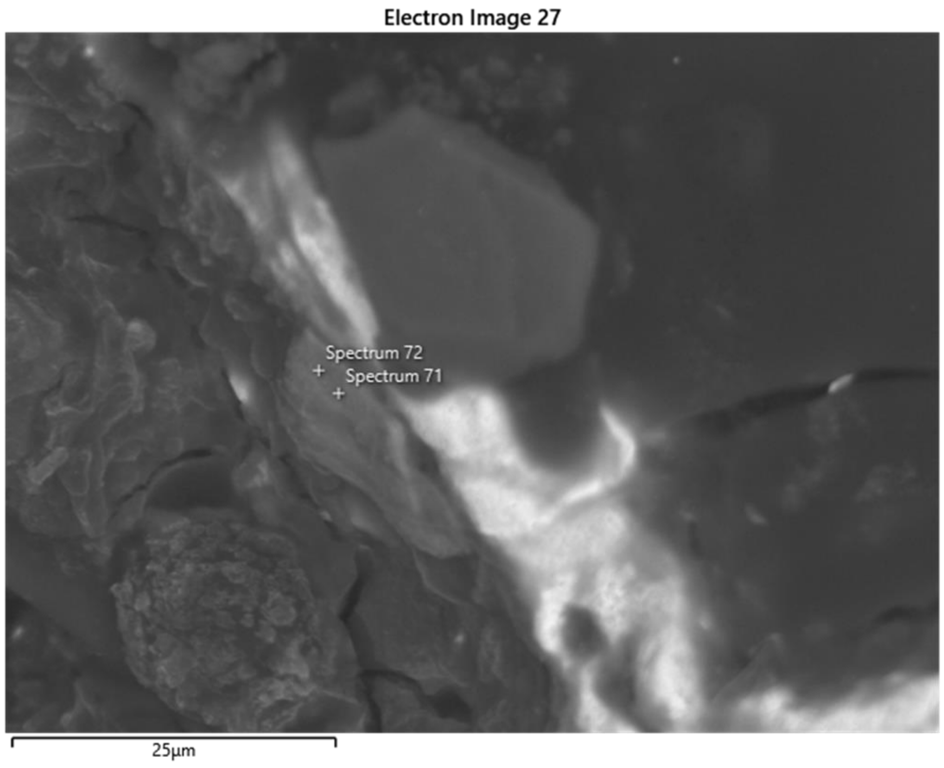

Figure S.3. 120 Area analysed on Coin GLAHM:40333

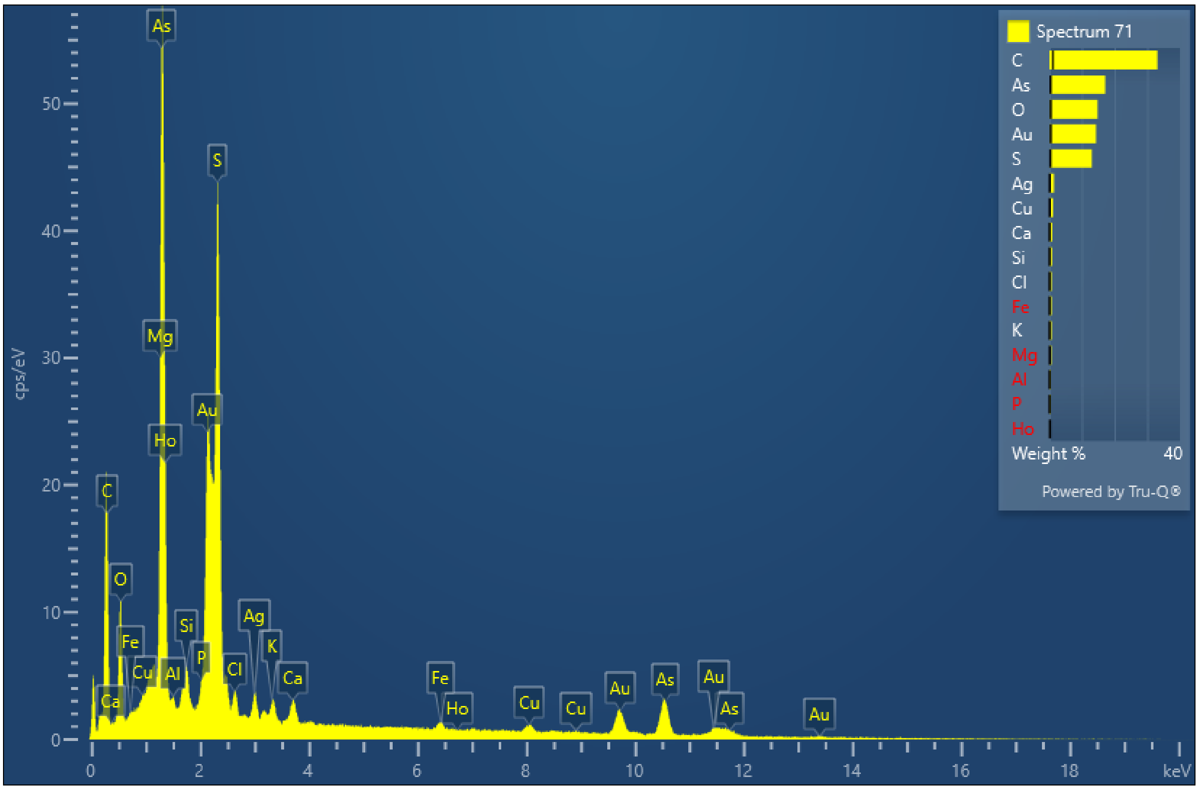

Figure S.3. 121 Spectrum 71 on Coin GLAHM:40333

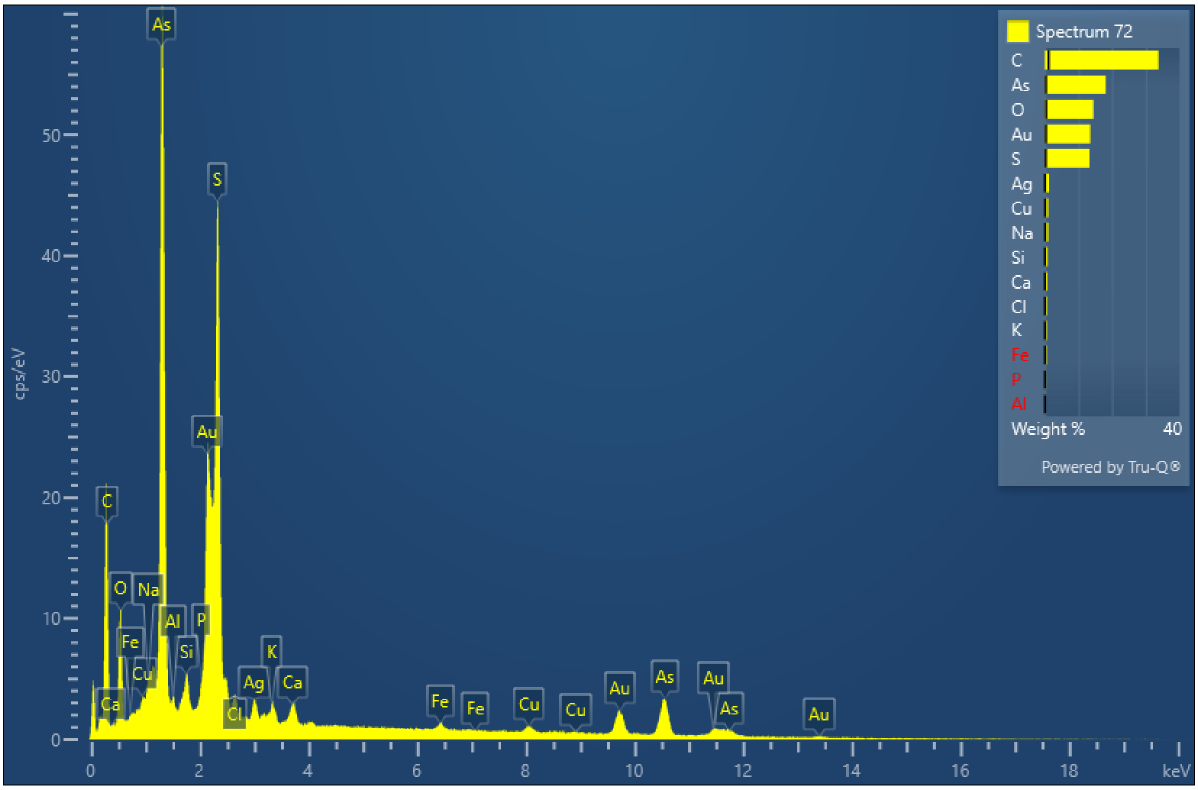

Figure S.3. 122 Spectrum 72 on Coin GLAHM:40333

Discussion: These two spectra are unlike any others obtained with major peaks associated with As and S which may indicate a naturally occurring mineral such as orpiment (As_2S_3) which is often associated with gold mineralization.
