# Supplementary material for: Authenticating coins of the ‘Roman emperor’ Sponsian
Source: PLoS One. 2022 Nov 23;17(11):e0274285. doi: 10.1371/journal.pone.0274285 (PMC9683583; doi:10.1371/journal.pone.0274285)
Supplement: S6 File — (PDF) [file pone.0274285.s006.pdf]

Supporting Information 6: Reflection mode Fourier Transform Infra-red spectroscopy (r-FTIR)

Contents

Introduction 3
S.6.1 Coin GLAHM:29540 (Genuine Gordian III aureus) 4
S.6.1.1 Obverse side 4
Figure S.6.1 Areas of the obverse of Coin GLAHM29540 where analyses were conducted .. 4
Figure S.6.2 Area 1 on Figure S.4.1 showing location of analysis on a large area of earthen deposit. 4
Figure S.6.3 Area 2 on Figure S.4.1 showing location of analysis on a large area of earthen deposit. 5
Table S.6.1 Position and intensity of the main IR bands at Point 1 on Figure S.6.3 and their interpretation 6
Figure S.6.4 Area of interest under LM. Note the prominent white blob 6
Figure S.6.5 Area 3 on Figure S.6.1 showing location of analysis 7
Figure S.6. 6 Spectrum from Point 2 in Figure S.6.5. Note that this spectrum is reproduced in Figure 11 of the main text. 7
Table S.6.2 Position and intensity of the main IR bands at Point 2 and their intensity 8
S.6.1.2 Reverse side 8
Figure S.6. 7 Area of the reverse of Coin GLAHM29540 where analyses were conducted 9
Figure S.6. 8 Detail of reverse of Coin GLAHM29540 showing position of analyses 9
Figure S.6. 9 Spectrum from Point 1 on Figure S.6.8 9
Table S.6.3 Position and intensity of the main IR bands at Point 1 and their intensity 10
S.6.2 Coin GLAHM:29697 (Genuine Philip I aureus), obverse 11
Figure S.6. 10 Area of the obverse of Coin GLAHM:29540 where analyses were conducted 11
Figure S.6. 11 Area of Figure S.4.10 showing location of analysis in a small area of earthen deposit in a recess 11
Figure S.6. 12 Spectrum from Point 1 on Figure S.4.11 compared with Point 2 on Figure S.6.5 12
Table S.6.4 Position and intensity of the main IR bands at Point 1 on Figure S.6.11 12
S.6.3 Coin GLAHM:29596 (Questionable Gordian III medallion/binio) 13
S.6.4 Coin GLAHM:29820 (Questionable Philip I medallion) 13
S.6.5 Coin GLAHM:29821 (Questionable Philip I medallion) 14
S.6.5.1 Obverse side 14
Figure S.6. 14 Area of Figure S.4.13 showing location of analysis in an area of earthen deposit 14

Figure S.6.15 Spectra from 1a (orange), 2a (red) and 3 (blue) on Figure S.6.15 15

Table S.6.5 Position of the main IR bands in spectra from 1a (orange on Figure S.6.16), 2a (red) and 3 (blue) 16

S.6.5.2 Reverse side 16

Figure S.6. 17 Areas of the reverse of Coin GLAHM:29821 where analyses were conducted 17

Figure S.6. 18 Area of Figure S.4.16 showing location of analyses 17

Figure S.6.19 Area of fluorescence in UV light.. 18

Figure S.6. 20 Comparison between the spectrum acquired from point 1 of area A (purple) and a reference spectrum of shellac (after Kramers-Kronig correction) (red)..................... 18

Table S.6.6 Position and intensity of the main IR bands of the spectrum acquired from point 1 of area A 19

Figure S.6.21 Area showing the location of a second analysis in Area A on the reverse side 19

Figure S.6. 22 Spectrum acquired from point 2 on Figure S.4.20 20

Table S.6.7 Position and intensity of main IR bands from Point 2 on Figure S.4.20 21

S.6.6 Coin GLAHM:40333 (Questionable Sponsian medallion), obverse....................... 22

Figure S.6. 23. Areas on the obverse of Coin GLAHM:40333 where several points were analysed. 22

Figure S.6.24 Area 1 on Coin GLAHM:40333 22

Figure S.6.25 Comparison of the spectrum collected from point 2, first area (green) and a reference spectrum of carnauba wax (red, collection in transmission mode)..................... 23

Table S.6.8 Position and intensity of the main IR bands at poin2 and their interpretation . 24

Figure S.6.26 Second area studied on Coin GLAHM:40333 24

Figure S.6.27 Light microscope image of position of point 1.. 25

Figure S.6.28 Spectrum from point 1 in Figure S.4.25 25

Table S.6.9 Position and intensity of the main IR bands at point 1 in Figure S.4.25 26

Figure S.6.29 Light microscope image showing the area of suspected wax analysed at point 3 26

Figure S.6. 30 Spectrum collected from point 3 27

References 27

Introduction

Four coins of the Hunterian collection were analysed non-destructively by μ-FTIR in reflection mode. Each coin, laying on a polystyrene support, was placed under the microscope of a benchtop FTIR instrument, a Nicolet iN10 FTIR AutoImage microscope. A cooled mercury-cadmium-telluride (MCT) detector was selected. The parameters chosen for the analysis at high spectral resolution were: 22 sec, 64 scans, 4 cm^{-1} resolution, 100x100 μm aperture, 4000-675 cm^{-1} spectral range, Beer-Norton strong apodization. No further spectral correction was applied after the collection of each spectrum. Point analysis was carried out on both sides of the coins to characterize the main organic and inorganic compounds present at surface. Points of interest were chosen based on the results of the microscopic investigation by visible and UV light.

S.6.1 Coin GLAHM:29540 (Genuine Gordian III aureus)

S.6.1.1 Obverse side

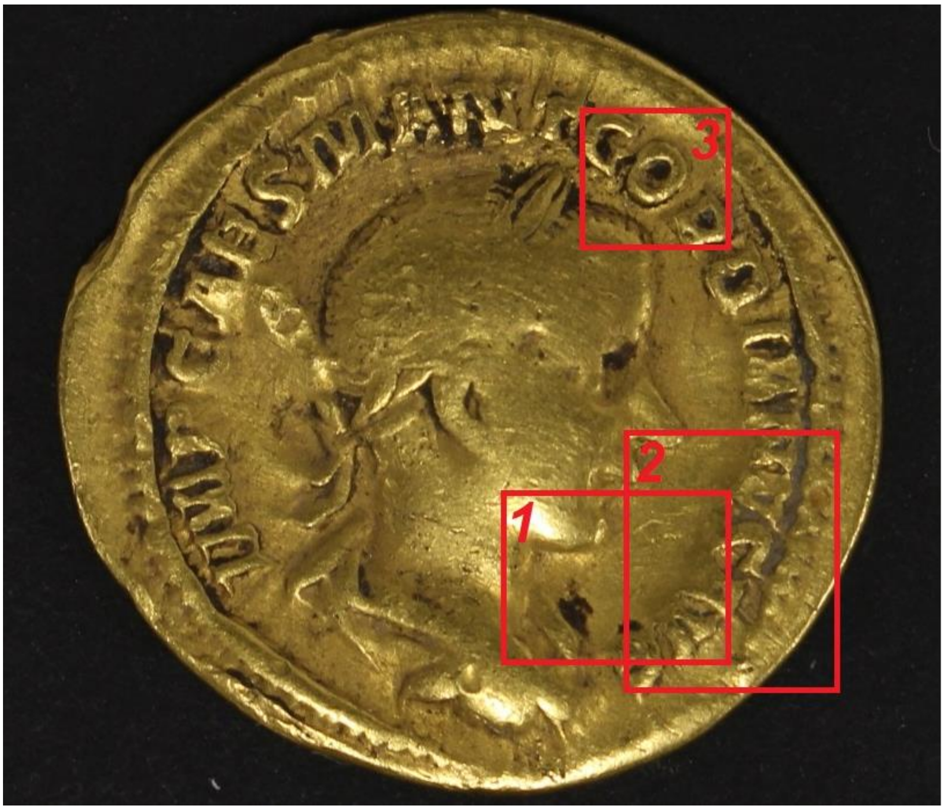

Figure S.6.1 Areas of the obverse of Coin GLAHM29540 where analyses were conducted

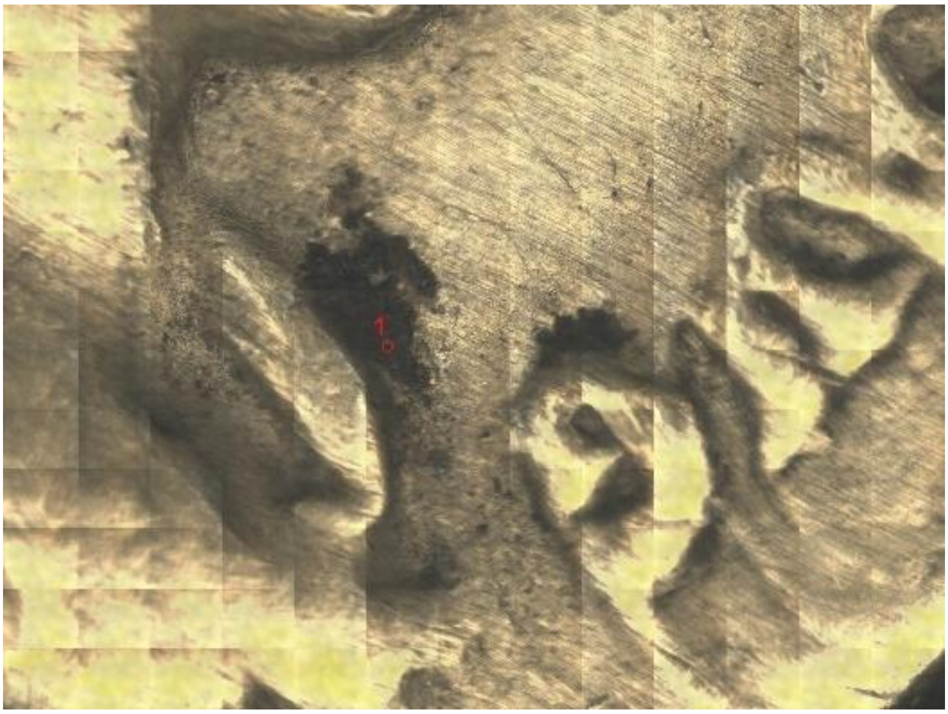

Figure S.6.2 Area 1 on Figure S.6.1 showing location of analysis on a large area of earthen deposit.

No results reported.

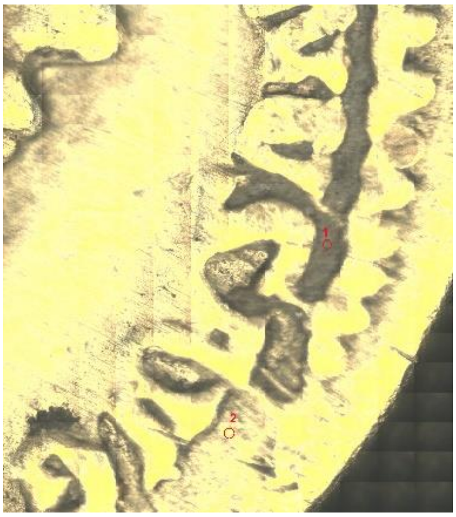

Figure S.6.3 Area 2 on Figure S.6.1 showing location of analysis on a large area of earthen deposit.

Interpretation if the spectrum at Point 1 on Figure S.6.3 is given in Table S.6.1:

**GLAHM29540_2nd area_obverse_point1**		
**Position (cm^{-1})**	**Intensity (arb. u.)**	**Band assignment**
696	1.994	Quartz, Si-O [1]
713	1.988	Calcium carbonate, ν_2(O-C-O) [2]
803	1.960	Clays, OH deformation (linked to Al^{3-}, Mg^{2-}) [1]
849	1.881	Not determined
873	1.911	Calcium carbonate, $\nu_4(CO_3^{-2})$ [2]
924	1.948	Calcium oxalate, ν(C-C) [3,4], Clays, (OH def., due to $2Al^{3-}$) [1]
1049	1.868	Clays, Si–O stretching mode [5], gypsum (ν_1 SO_4 mode) [6]
1098	1.834	Clays, Si–O stretching mode [5]
1182	1.879	Not determined
1322	1.915	Calcium oxalate, ν_5(C-O); overlapping δ(O-C-O) [3,4]
1398	2.013	Calcium oxalate, ν_5(C-O); overlapping δ(O-C-O) [3,4]
1435	1.991	Copper carbonate, $\nu_3(CO_3^{-2})$ [7]
1463	1.988	Copper carbonate, $\nu_3(CO_3^{-2})$ [7]
1633	2.175	Calcium oxalate, ν_3(C-O); overlapping δ(H-O-H) [3,4]
1796	1.535	Calcium carbonate, combination $\nu_1+\nu_4(CO_3^{-2})$ [5,8]
1869	1.322	Copper carbonate, combination $\nu_1+\nu_4(CO_3^{-2})$ [5,8]
1996	1.226	Not determined
2147	1.201	Gypsum (ν_1 + ν_3 SO_4) [9]

2240	1.203	Gypsum (bending and libration modes of H_2O; $\nu_2 + \nu_L$) [6,9]
2521	1.470	Calcium carbonate, combination ν_1+ν_3(CO_3^{-2}) [5,8]
2576	1.355	Copper carbonate, combination ν_1+ν_3(CO_3^{-2}) [5,8]
2627	1.346	Calcium carbonate, combination ν_1+ν_3(CO_3^{-2}) [5,8]
2850	1.885	Organic, not defined
2919	2.001	Organic, not defined
2955	1.870	Organic, not defined
3402	2.028	Gypsum (O-H stretching) [6,9]
3541	1.980	Gypsum (O-H stretching) [6,9]
3618	1.872	Clay, multiplet [10]
3697	1.482	Clay, multiplet [10]

Table S.6.1 Position and intensity of the main IR bands at Point 1 on Figure S.6.3 and their interpretation

Discussion: The above analysis indicates that there are several compounds, including carbonate (calcium or copper-based), oxalate and sulphate species (gypsum; $CaSO_4 \cdot 2H_2O$), as well as silica and clay minerals.

The next area examined was a large area of earthen deposit within the letter 'O' (an area also examined by SEM-EDX; see Supplementary Information 3, Section S.3.1.4).

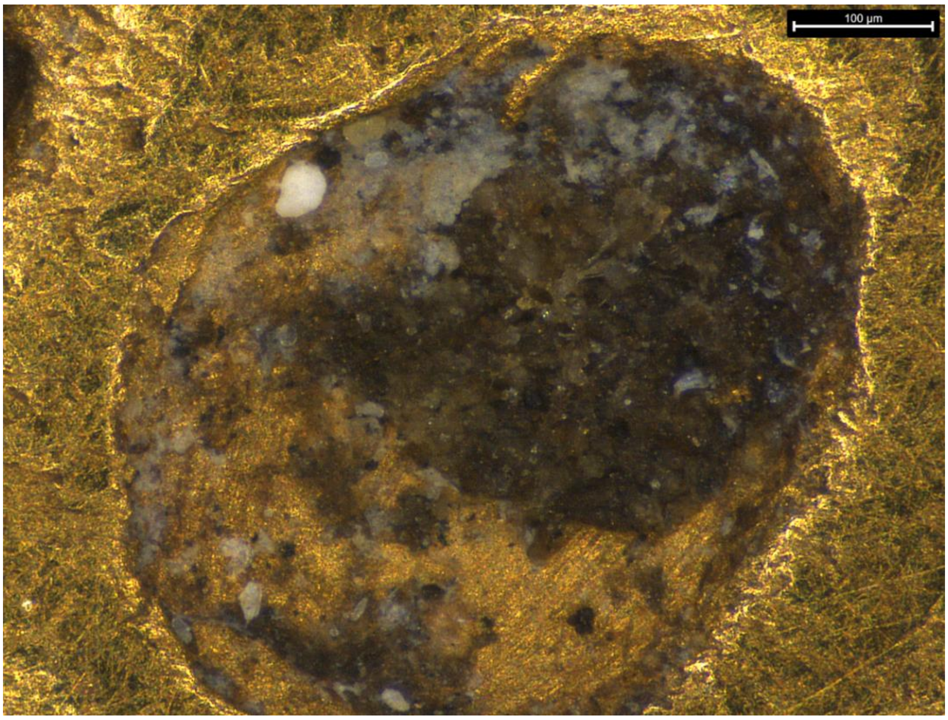

Figure S.6.4 Area of interest under LM. Note the prominent white blob

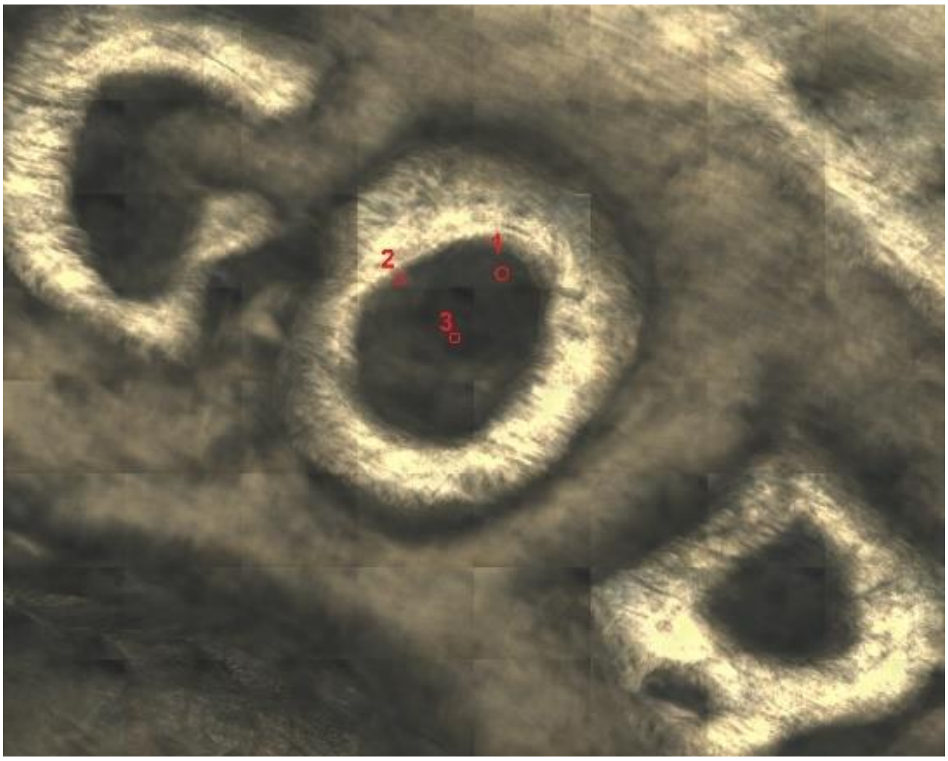

Figure S.6.5 Area 3 on Figure S.6.1 showing location of analysis

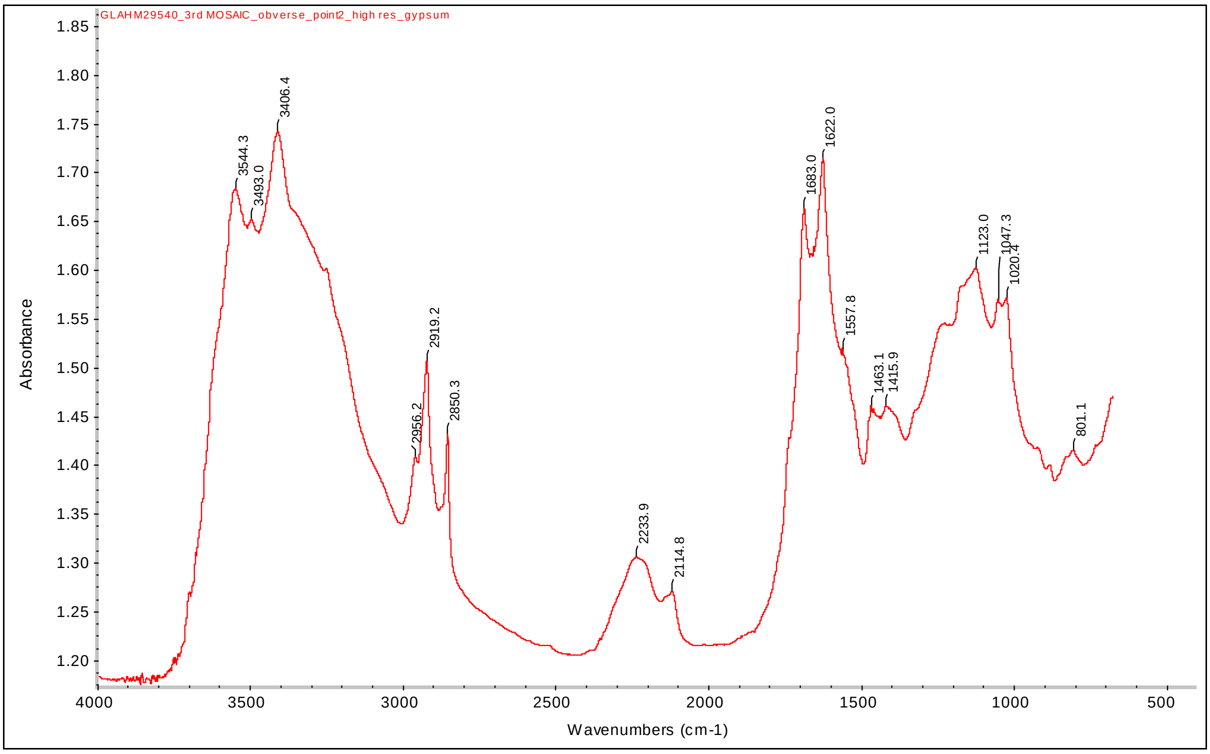

Figure S.6. 6 Spectrum from Point 2 in Figure S.6.5. Note that this spectrum is reproduced in Figure 11 of the main text.

**GLAHM29540_3rd area_obverse_point2**		
**Position (cm^{-1})**	**Intensity (arb. u.)**	**Band assignment**
801	1.414	Quartz, Si-O [1]
1020	1.570	Gypsum (ν_1 SO_4 mode) [9]

1047	1.568	
1123	1.600	Gypsum (ν_3 SO_4 antisymmetric stretching mode) [9]
1416	1.459	
1463	1.460	Possibly organic, not defined
1558 shoulder	1.519	Possibly organic, not defined
1622	1.714	Gypsum (O-H bending) [6,9]
1683	1.663	Gypsum (O-H bending) [6,9]
2114	1.269	Gypsum ($\nu_1 + \nu_3$ SO_4) [6,9]
2233	1.304	Gypsum (bending and libration modes of H_2O; $\nu_2 + \nu_L$) [6,9]
2850	1.430	Organic, not defined
2919	1.506	Organic, not defined
2956	1.407	Organic, not defined
3406	1.741	Gypsum (O-H stretching) [7]
3544	1.681	Gypsum (O-H stretching) [7]

Table S.6.2 Position and intensity of the main IR bands at Point 2 and their intensity

Discussion: the analysis indicates the presence of gypsum and an organic substance. The presence of an organic substance, not better characterized, is also hypothesized due to the bands at 2919, 2850, 1463, 1415 cm^{-1}. These bands might be attributed to calcium distearate [11], possibly coming from a cleaning activity. Calcium stearate is a carboxylate salt of calcium, classified as a calcium soap. The salt is a component of some lubricants, surfactants, as well as many foodstuffs. It is a white waxy powder.

S.6.1.2 Reverse side

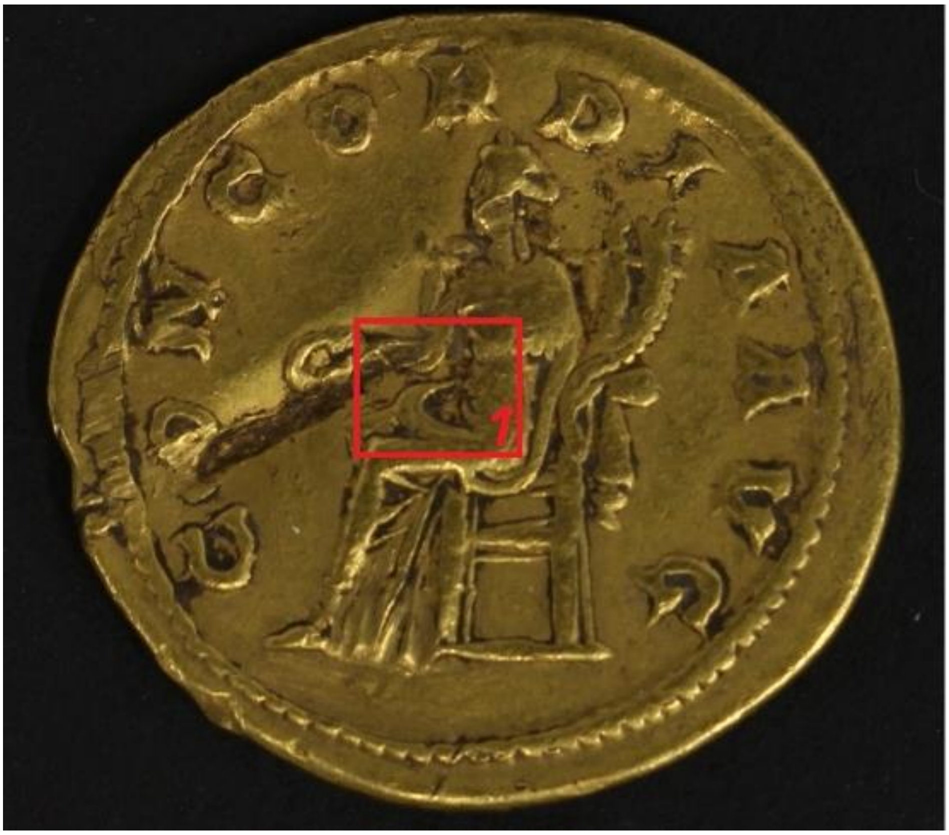

Figure S.6. 7 Area of the reverse of Coin GLAHM29540 where analyses were conducted

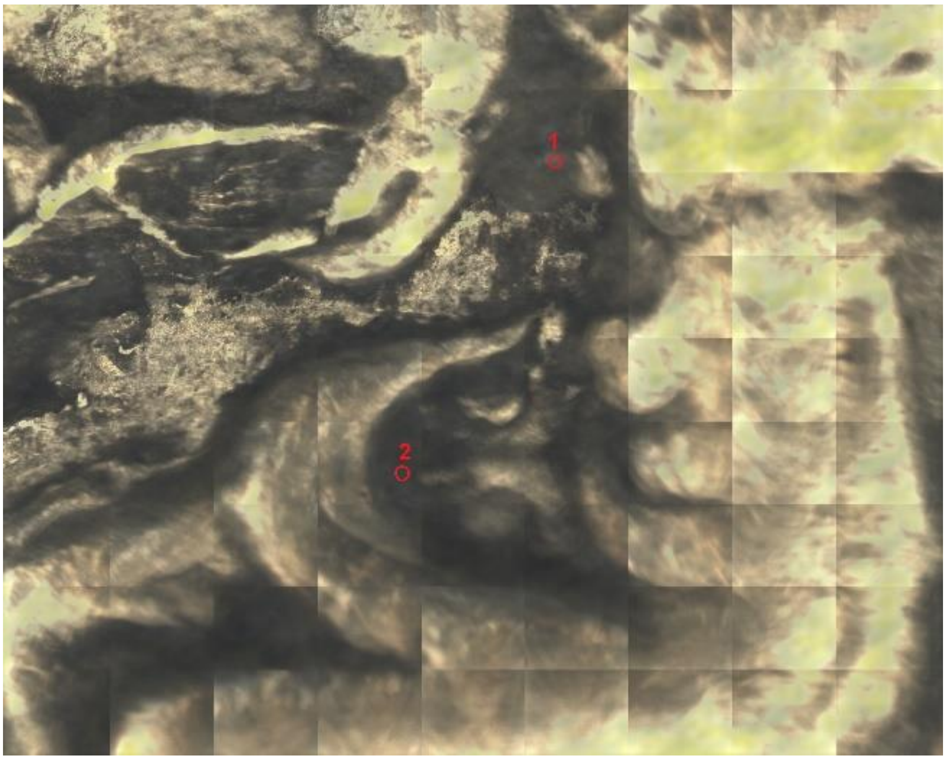

Figure S.6. 8 Detail of reverse of Coin GLAHM29540 showing position of analyses

The spectrum obtained from Point 1 on Figure S.6.8 is given in Figure S.6.9 and interpreted in Table S.6.3 below:

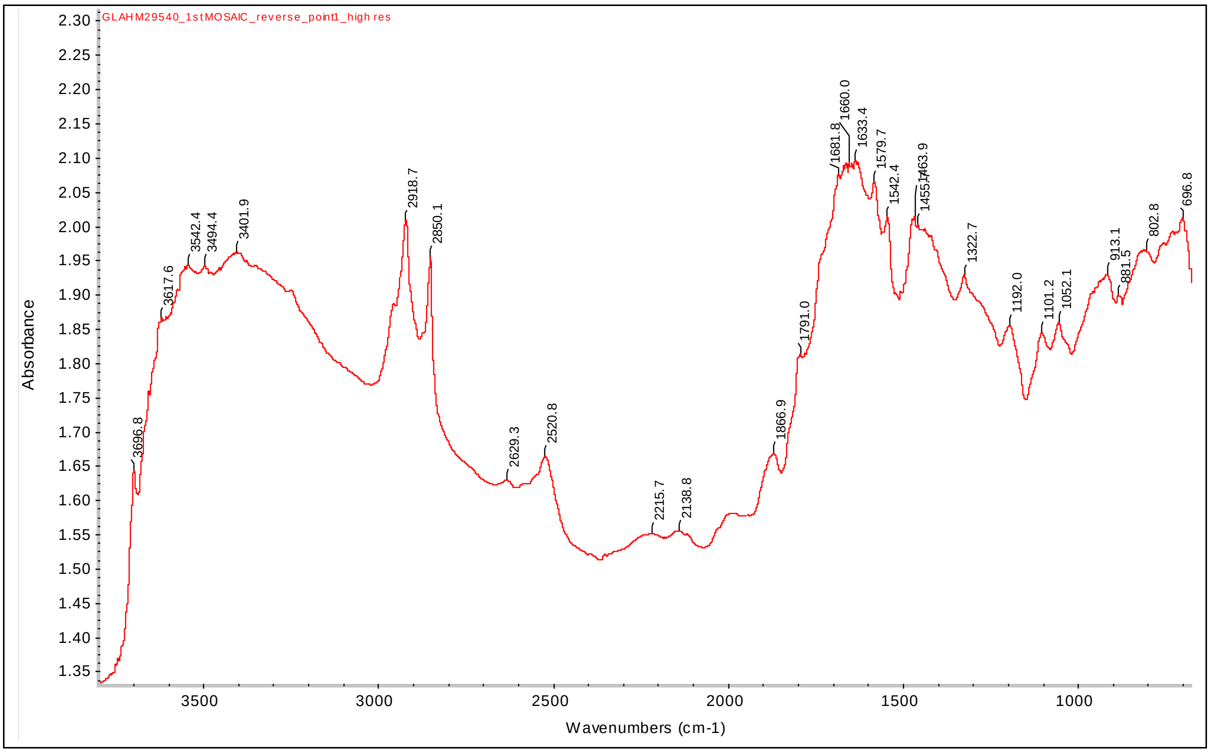

Figure S.6. 9 Spectrum from Point 1 on Figure S.4.8

GLAHM29540_1st area_reverse_point1		
**Position (cm^{-1})**	**Intensity (arb. u.)**	**Band assignment**
697	2.012	Quartz, Si-O [1]
803	1.964	Clays, OH deformation (linked to Al^{3-}, Mg^{2-}) [5]
881	1.897	Calcium carbonate, ν4(CO_3^{-2}) [2]
913	1.928	Calcium oxalate, ν(C-C) [3,4], Clays (OH def., due to $2Al^{3-}$) [5]
1052	1.858	Clays, Si–O stretching mode [1,5]
1101	1.843	Clays, Si–O stretching mode [1,5]
1192	1.852	Gypsum (ν_3 SO_4) [6,9]
1323	1.927	Calcium oxalate, ν_s(C-O); ν(C-C) [3,4]
1456	2.002	Copper carbonate, ν_3(CO_3^{-2}) [7]
1464	2.012	Copper carbonate, ν_3(CO_3^{-2}) [7]
1542	2.013	Possibly organic, not defined
1580	2.063	Possibly organic, not defined
1633	2.094	Calcium oxalate, ν_{as}(C-O); overlapping δ(H-O-H) [3,4]
1660	2.090	Gypsum (O-H bending) [6,9]
1791	1.812	Calcium carbonate, combination ν_1+ν_4(CO_3^{-2}) [5,8]
1867	1.667	Copper carbonate, combination ν_1+ν_4(CO_3^{-2}) [2,6]
2139	1.553	Gypsum (ν_1 + ν_3 SO_4) [6,9]
2216	1.549	Gypsum (bending and libration modes of H_2O; ν_2 + ν_L) [6,9]
2521	1.662	Calcium carbonate, combination ν_1+ν_3(CO_3^{-2}) [5,8]
2629	1.628	Calcium carbonate, combination ν1+ν3(CO_3^{-2}) [5,8]
2850	1.955	Organic, not defined
2919	2.008	Organic, not defined
3402	1.960	Gypsum (O-H stretching) [6,9]
3542	1.942	Gypsum (O-H stretching) [6,9]
3618	1.865	Clay, multiplet [10]
3697	1.642	Clay, multiplet [10]

Table S.6.3 Position and intensity of the main IR bands at Point 1 and their intensity

S.6.2 Coin GLAHM:29697 (Genuine Philip I aureus), obverse

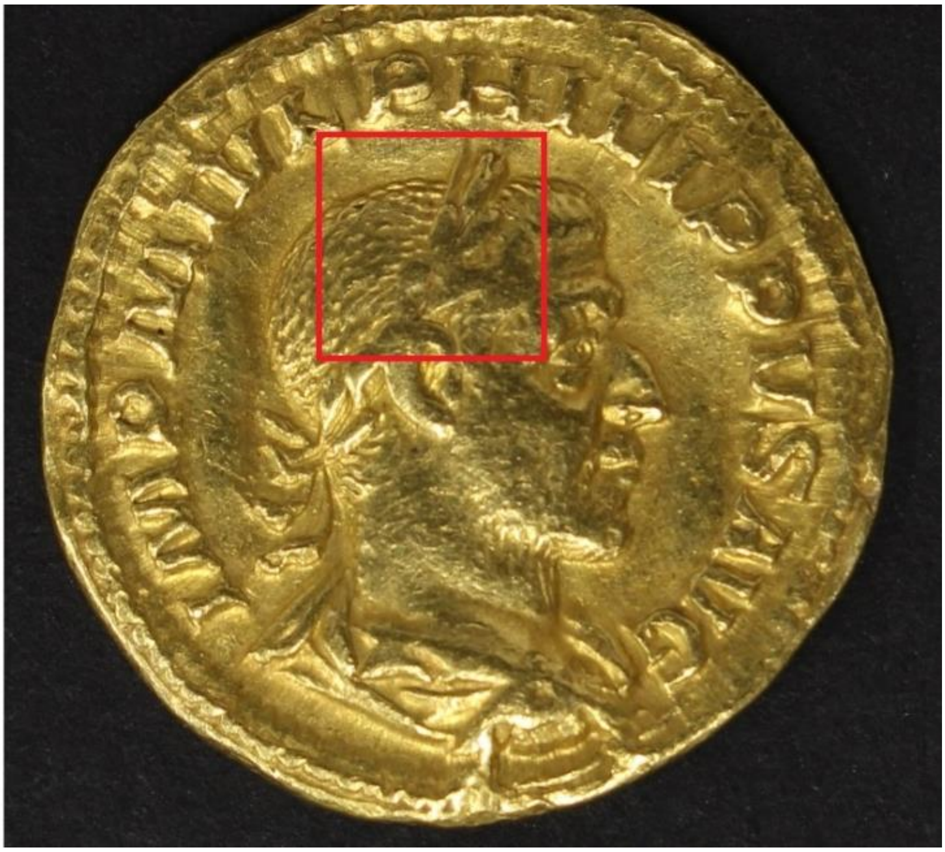

Figure S.6. 10 Area of the obverse of Coin GLAHM:29540 where analyses were conducted

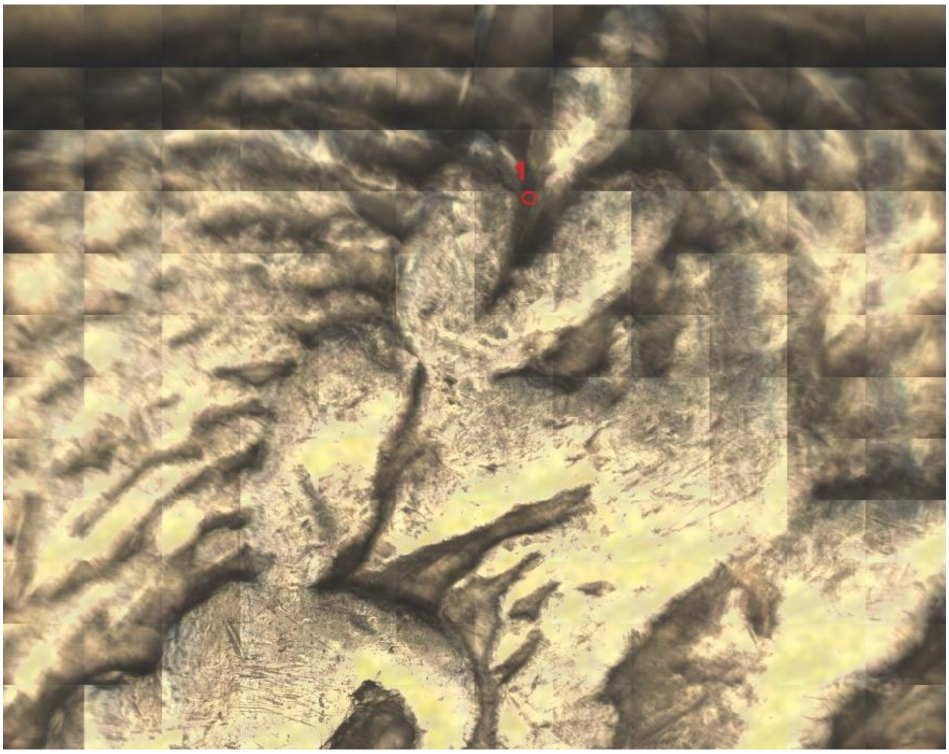

Figure S.6. 11 Area of Figure S.6.10 showing location of analysis in a small area of earthen deposit in a recess

The spectrum obtained from Point 1 on Figure S.6.11 is given in red in Figure S.6.12 alongside the spectrum from Point 2 in Figure S.6.5 of Coin GLAHM:29450 in blue, and interpreted in Table S.6.4 below:

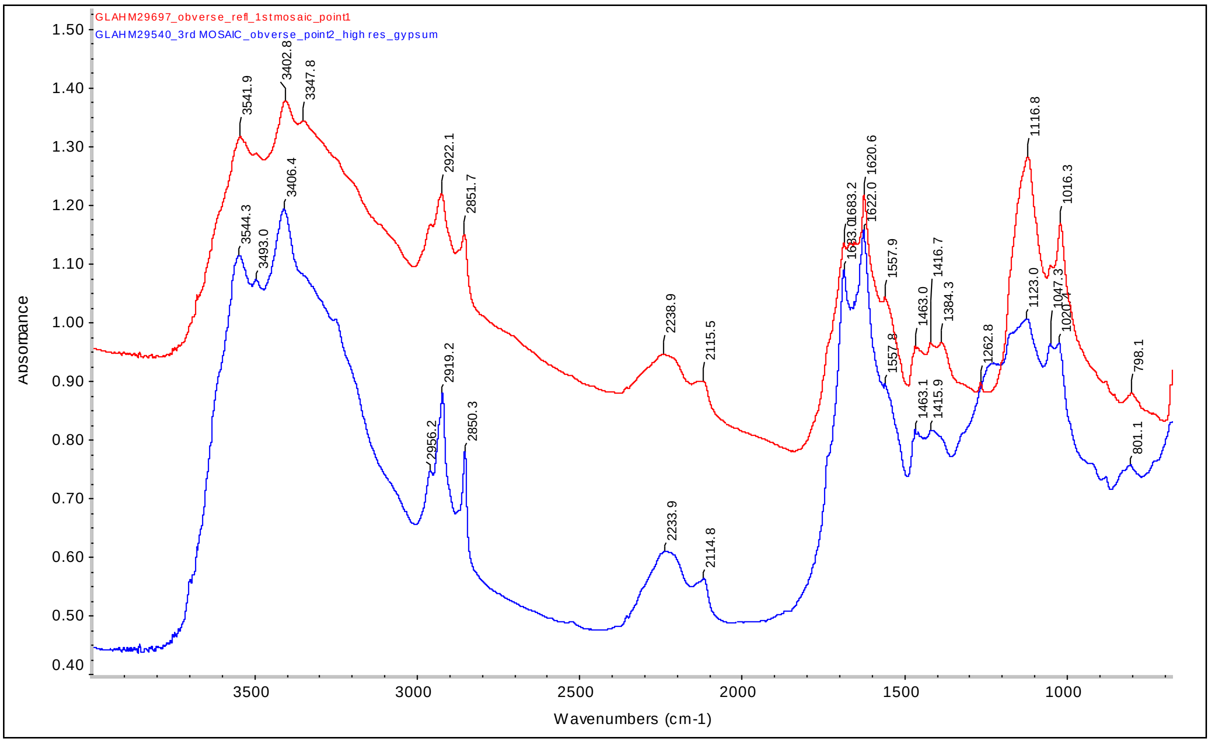

Figure S.6. 12 Spectrum from Point 1 on Figure S.6.11 compared with Point 2 on Figure S.6.5

**GLAHM:29697_obverse_refl_1st mosaic_point1**		
**Position (cm^{-1})**	**Intensity (arb. u.)**	**Band assignment**
798	0.877	Quartz, Si-O [1]
1016	1.167	Anhydrite (ν_3 SO_4) [9]
1117	1.280	Gypsum (ν_3 SO_4) [9]
1263	0.895	Possibly organic, not defined
1416	0.963	Possibly organic, not defined
1463	0.958	Possibly organic, not defined
1558 shoulder	1.042	Not defined
1621	1.215	Gypsum (O-H bending) [6,9]
1683	1.133	Gypsum (O-H bending) [6,9]
2115	0.898	Gypsum ($\nu_1 + \nu_3$ SO_4) [6,9]
2239	0.943	Gypsum (bending and libration modes of H_2O; $\nu_2 + \nu_L$) [6,9]
2852	1.148	Organic, not defined
2922	1.218	Organic, not defined
3403	1.376	Gypsum (O-H stretching) [6,9]
3542	1.315	Gypsum (O-H stretching) [6,9]

Table S.6.4 Position and intensity of the main IR bands at Point 1 on Figure S.4.11

Discussion: The two spectra shown in Figure S.6.12 are similar and both are interpreted as indicating mainly gypsum and an unknown organic compound, with minor quartz.

S.6.3 Coin GLAHM:29596 (Questionable Gordian III medallion/binio)

This coin was not analysed using r-FTIR.

S.6.4 Coin GLAHM:29820 (Questionable Philip I medallion)

This coin was not analysed using r-FTIR.

S.6.5 Coin GLAHM:29821 (Questionable Philip I medallion)

S.6.5.1 Obverse side

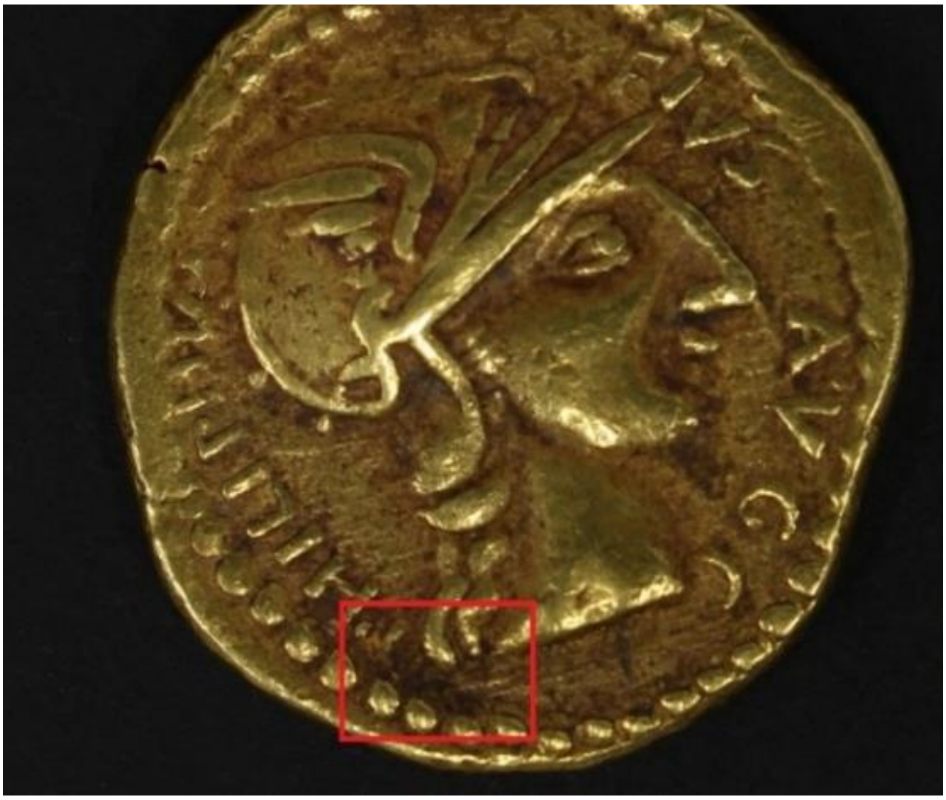

Figure S.6. 13 Area of the obverse of Coin GLAHM:29821 where analyses were conducted

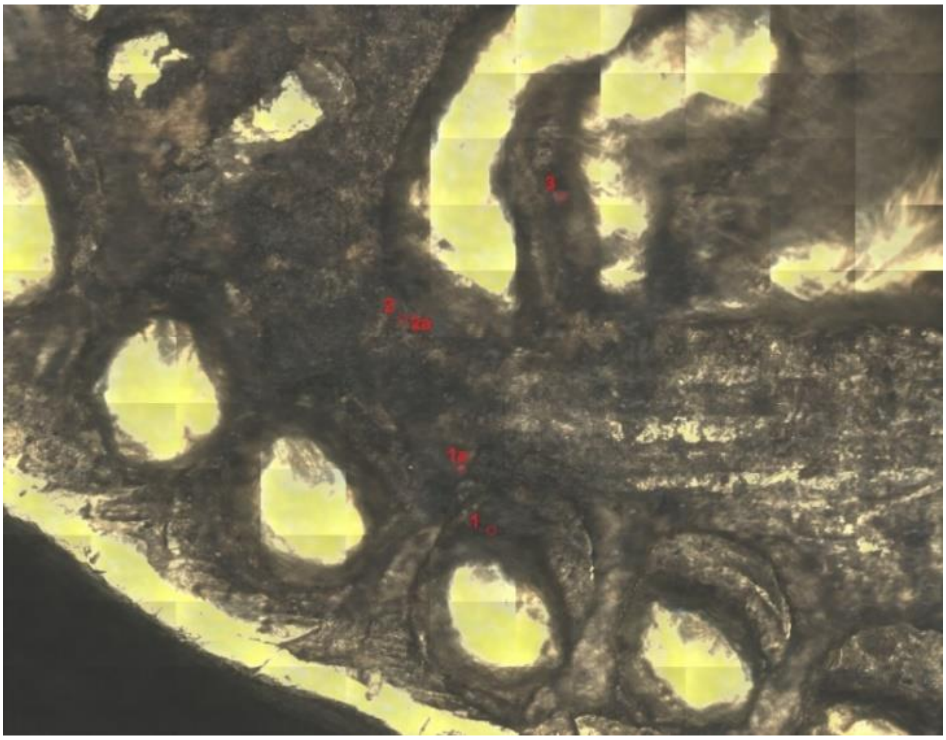

Figure S.6. 14 Area of Figure S.4.13 showing location of analysis in an area of earthen deposit

Spectra for points 1a, 2a and 3 are shown in Figure S.6.15 and compared in Table S.6.5

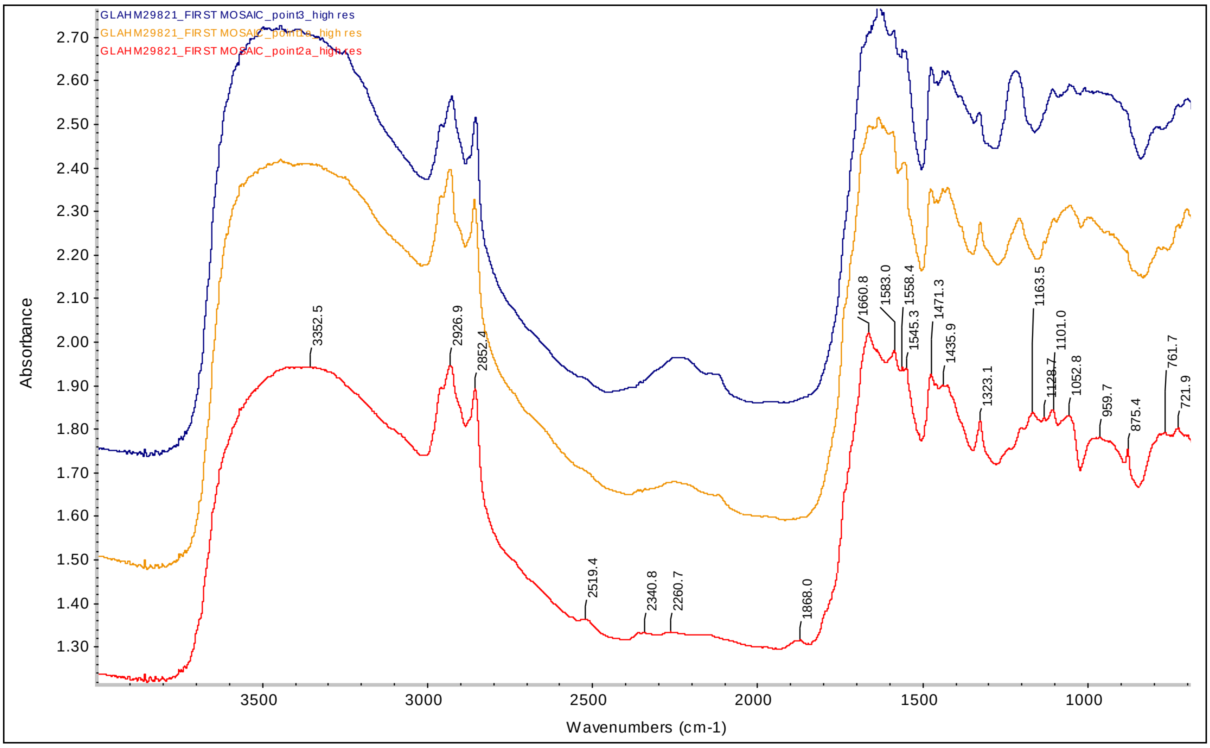

Figure S.6.15 Spectra from 1a (orange), 2a (red) and 3 (blue) on Figure S.6.15

GLAHM:29821_obv_point1a	GLAHM:29821_obv_point2a	GLAHM:29821_obv_point3
**Position (cm^{-1})**	**Position (cm^{-1})**	**Position (cm^{-1})**
696	-	694
721	722	723
-	875	-
-	960	-
994	-	1005
1046	1053	1052
1098	-	-
-	1101	1102
1133sm	1129	-
-	1163	-
1203	-	1216
1323	1323	1325
1422	-	1423
-	1436	1434
1473	1471	1470
1550	1545	1558
1583	1583	1584
-	-	1631
1660	1661	-
-	1868	-
2252	2261	2242
-	2341	-
-	2519	-

GLAHM:29821_obv_point1a	GLAHM:29821_obv_point2a	GLAHM:29821_obv_point3
**Position (cm^{-1})**	**Position (cm^{-1})**	**Position (cm^{-1})**
2854	2852	2851
2929	2927	2923
-	-	2956
-	-	3248
-	3352	-
3442	-	-

Table S.6.5 Position of the main IR bands in spectra from 1a (orange on Figure S.6.16), 2a (red) and 3 (blue)

Discussion: The composition of the deposits found on the obverse side of Coin GLAHM:29821 are quite similar in all points (Table S.6.5, Figure S.6.15). They show:

- **sulphates**, see the combination modes in the spectral region from 2400 to 2100 cm^{-1};
- **carbonates**, possibly Cu-based, because of the small bands at 1868, 1660, 1422, 960 cm^{-1} (see the red spectrum);
- **Ca-oxalates**, due to the small bands in the hydroxyl unit region, 3600-3000 cm^{-1}, the broad band at 1630 cm^{-1} and the sharp peak at 1323 cm^{-1} (see the blue spectrum Figure S.6.15).

The presence of an organic component like stearate is also hypothesised.

S.6.5.2 Reverse side

Two areas were selected for study on the reverse of Coin GLAHM:29821 as shown in Figure S.6.16. The first of these areas is shown in Figure S.6.17.

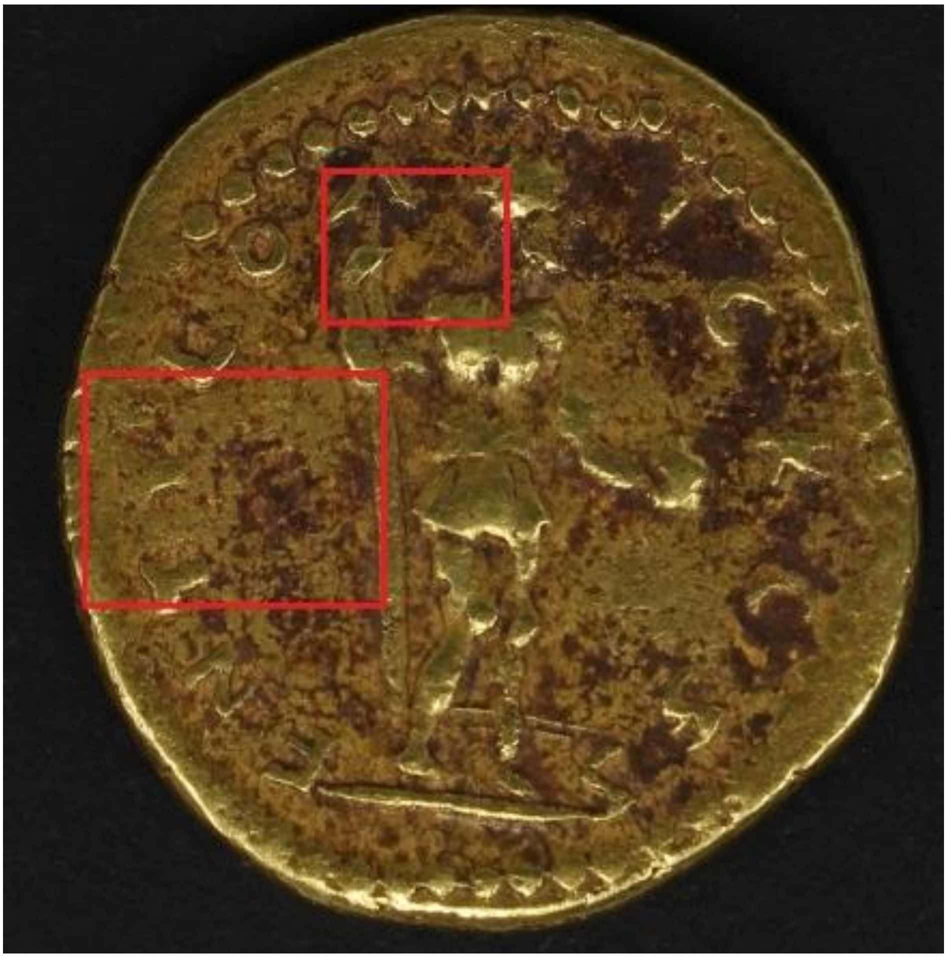

Figure S.6. 17 Areas of the reverse of Coin GLAHM:29821 where analyses were conducted

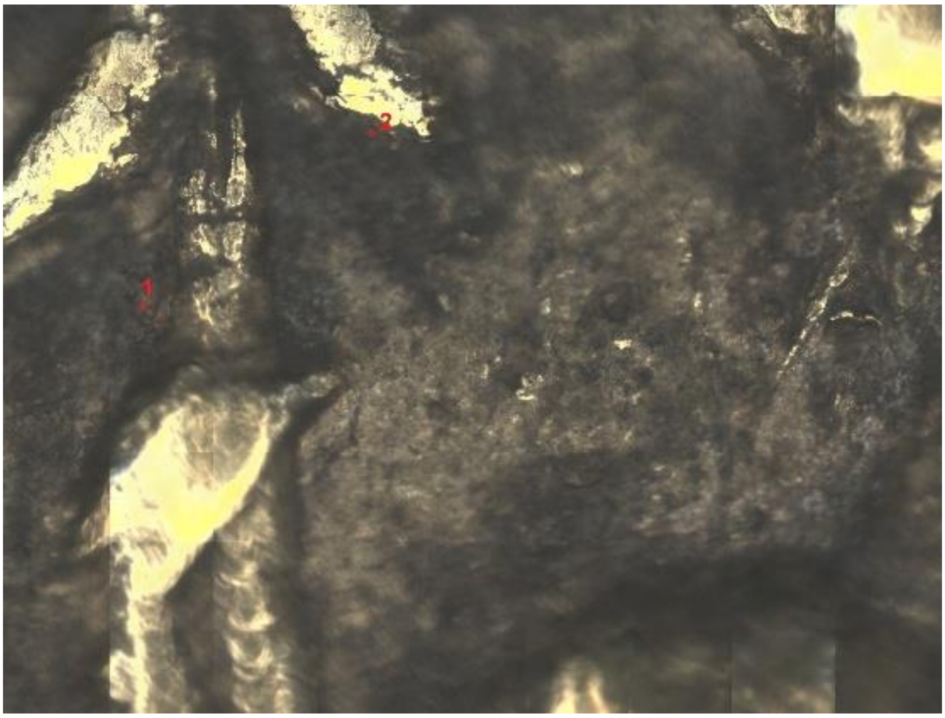

Figure S.6. 18 Area of Figure S.6.16 showing location of analyses

The first of the two spectra was obtained from an area of bright orange fluorescence in UV light which had tentatively been identified as shellac resin (Supplementary information 2 Section S.2.5). This is highlighted in Figure S.6.18. The spectrum obtained is compared with a reference for shellac in Figure S.6.19 with an interpretation in Table S.6.6 below.

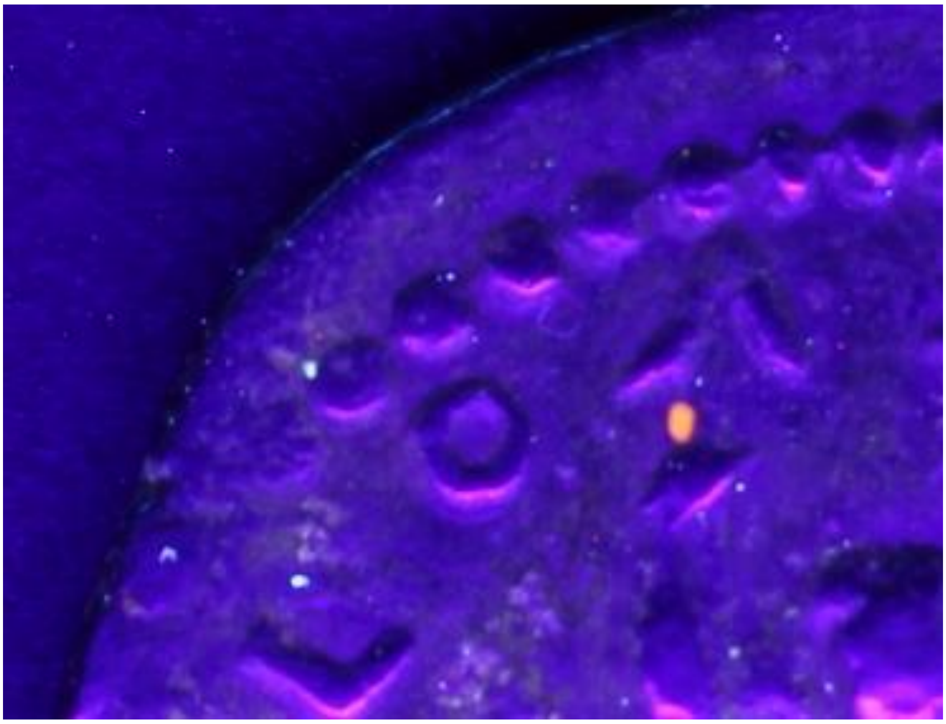

Figure S.6.19 Area of fluorescence in UV light

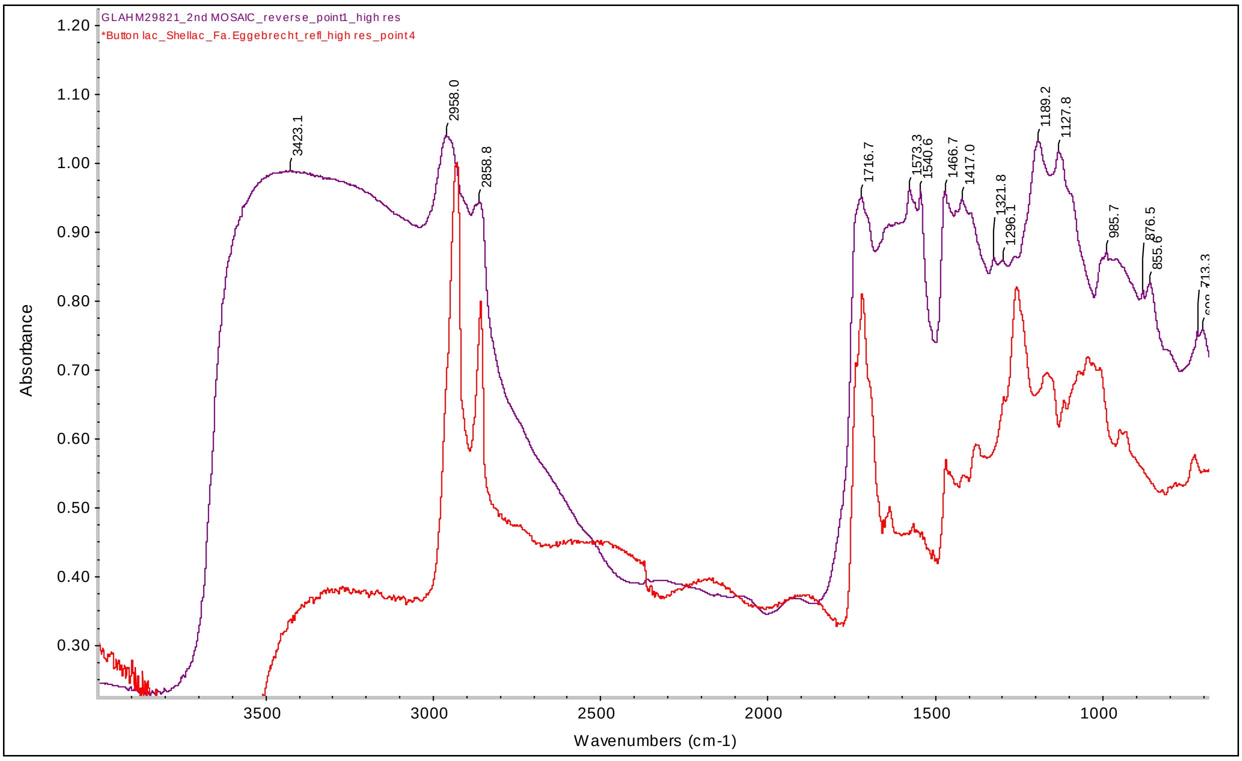

Figure S.6. 20 Comparison between the spectrum acquired from point 1 of area A (purple) and a reference spectrum of shellac (after Kramers-Kronig correction) (red)

GLAHM:29821_area A_reverse_point1		
**Position (cm^{-1})**	**Intensity (arb. u.)**	**Band assignment**
713	1.554	Calcium carbonate, ν_2(O-C-O) [2]
856	1.614	Not defined
877	1.606	Calcium carbonate, $\nu_4(CO_3^{-2})$ [2]
986	1.655	Shellac, ($\nu H_2C=C$) [12]
1128	1.779	Not defined
1189	1.793	Shellac, (νC-O) [12]
1296	1.643	Shellac, (νC-O) [12]
1322	1.646	Calcium oxalate, ν_s(C-O); ν(C-C) [3,4]
1417	1.720	Not defined
1467	1.730	Shellac, (δCH_2) [12], possible further organic contribution
1541	1.731	Possibly organic, not defined
1573	1.731	Possibly organic, not defined
1718	1.722	Shellac, (νC=O) [12]
2859	1.716	Shellac, (νCH_2) [12]
2958	1.799	Shellac, (νCH_2) [12]

Table S.6.6 Position and intensity of the main IR bands of the spectrum acquired from point 1 of area A

Discussion: The analysis confirmed the presence of shellac resin, possibly an adventitious spot on the coin because the substance is common in museum environments and was not detected elsewhere on any of the studied coins. Also present are minor carbonates and possibly an organic substance such as calcium distearate.

A spectrum was also obtained from the area of a mineral grain seen in Figure S.4.20 below.

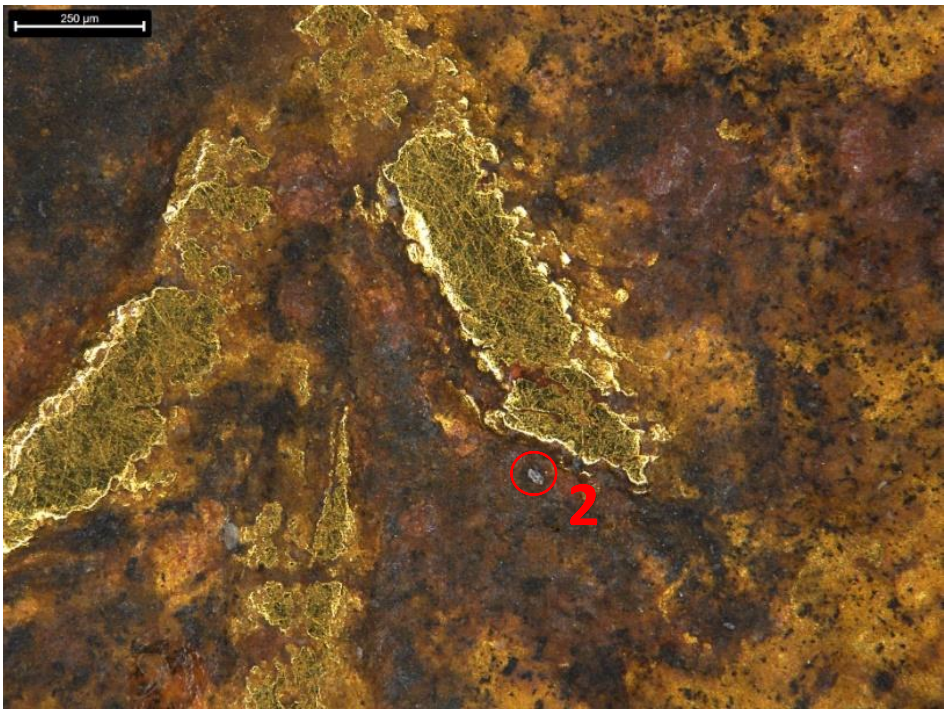

Figure S.6.21 Area showing the location of a second analysis in Area A on the reverse side

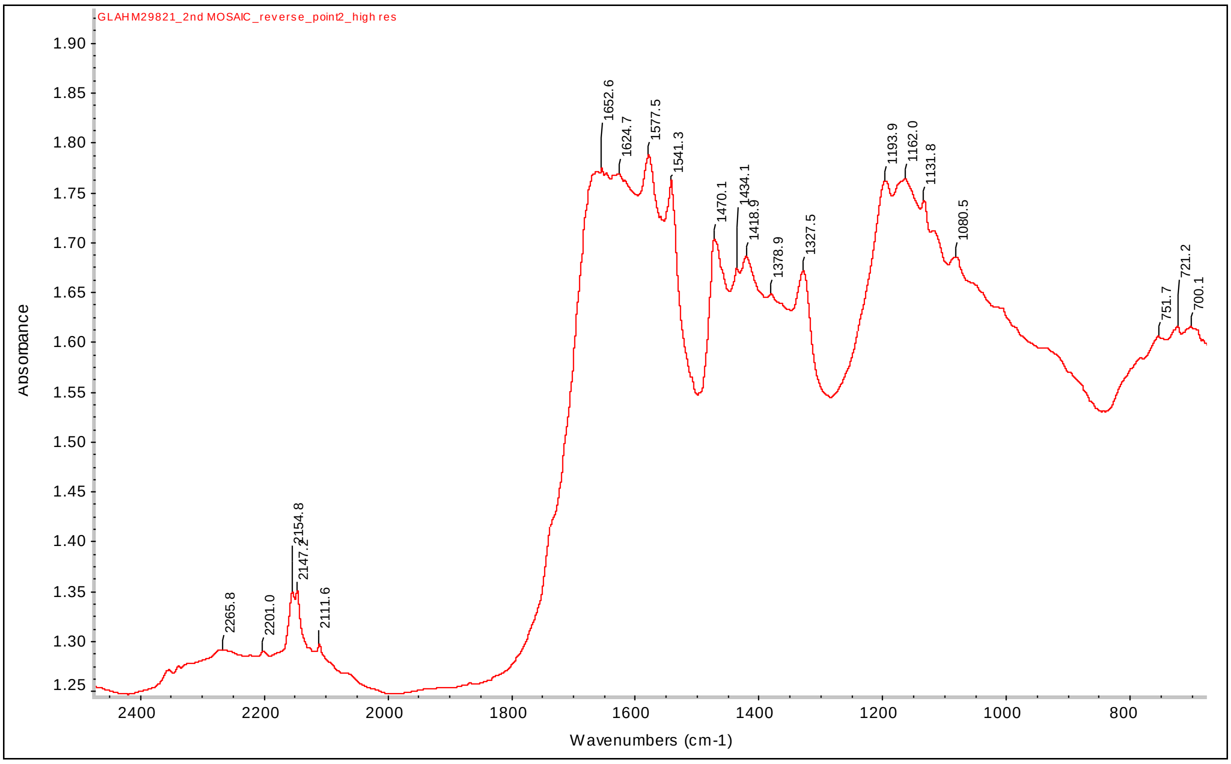

Figure S.6. 22 Spectrum acquired from point 2 on Figure S.6.20

**GLAHM:29821_area A_reverse_point2**	
**Position (cm^{-1})**	**Intensity (arb. u.)**
700	1.614
721	1.614
752	1.604
1080	1.684
1132	1.741
1162	1.762
1194	1.761
1327	1.670
1419	1.685
1434	1.673
1470	1.701
1541	1.761
1577	1.786
1625	1.768
1653	1.773
2112	1.296
2147	1.349
2155	1.348
2201	1.288
2266	1.289
2850	1.760
2919	1.831
2956	1.689
3319	1.828

Table S.4.7 Position and intensity of main IR bands from Point 2 on Figure S.6.20

Discussion: Sulphates are present, possibly different from gypsum like jarosite ($KFe_3(SO_4)_2(OH)_6$) [13] as indicated by the combination modes in the region 2200-2000 cm^{-1} and the small, sharp bands at 1193 and 1080 cm^{-1}. An organic component, not better characterized, is also present, as suggested by the sharp bands at 2955, 2918, 2850 cm^{-1}, the doublet at 1576 + 1540, and the band at 1470 cm^{-1}, which might be attributed to calcium distearate. This composition is very similar to that found for the 2 spectra in area B (not shown).

S.4.6 Coin GLAHM:40333 (Questionable Sponsian medallion), obverse

Three areas were examined as shown in Figure S.6.22. The first area is shown in Figure S.6.23.

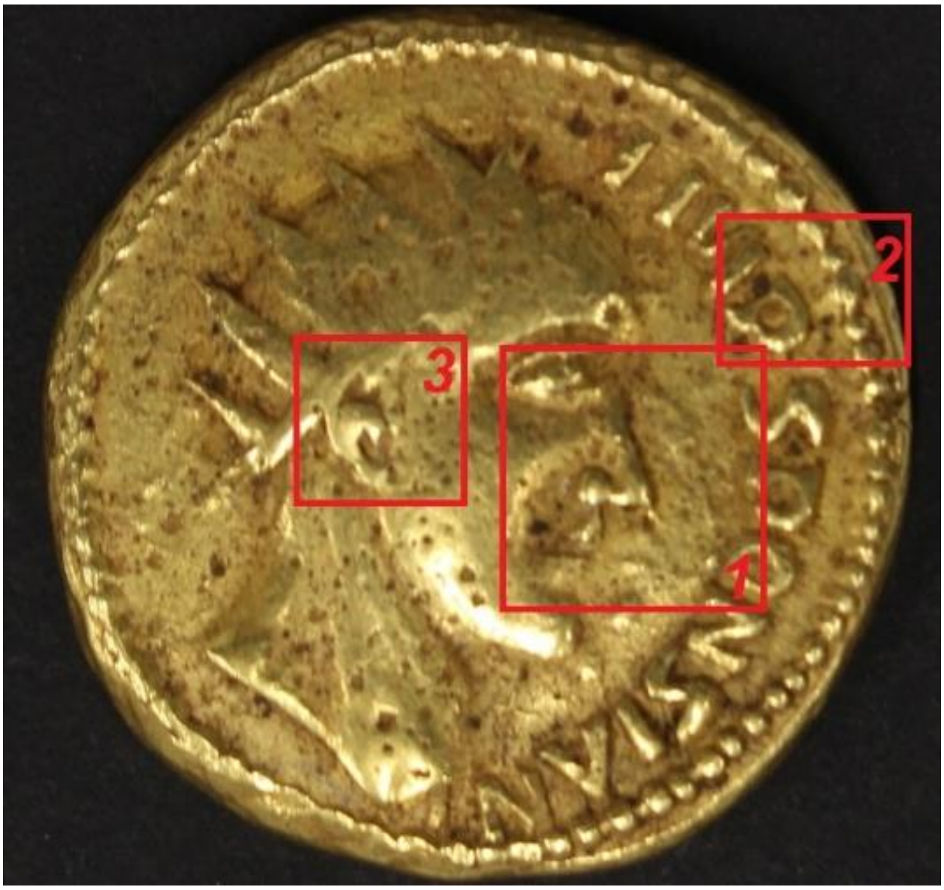

Figure S.6. 23. Areas on the obverse of Coin GLAHM:40333 where several points were analysed.

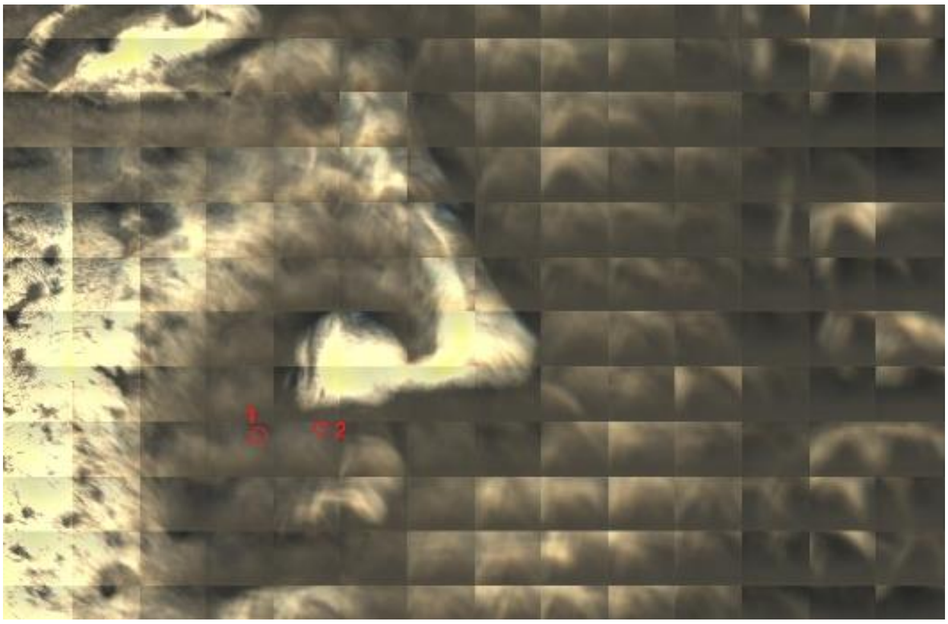

Figure S.6.24 Area 1 on Coin GLAHM:40333

The spectrum from point 2 on a light coloured patch below the emperor's nose is shown in Figure S.6.24 beside a reference spectrum was wax. The main absorption bands are given in Table S.6.8.

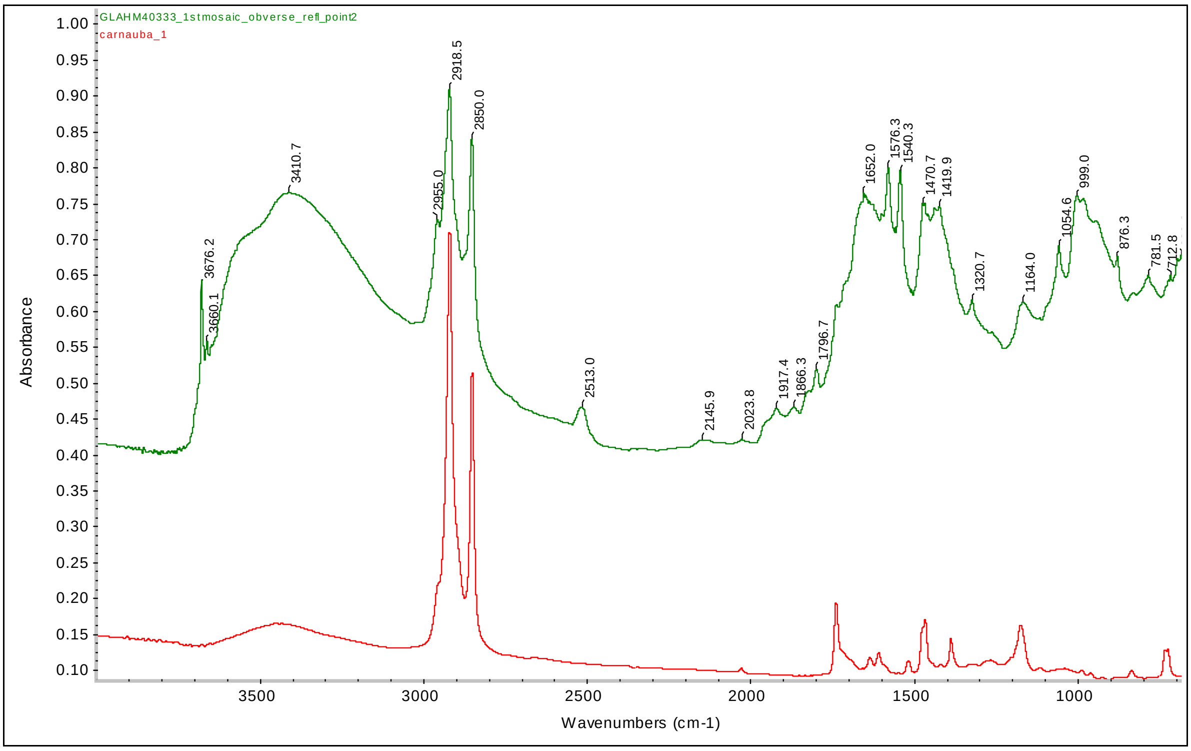

Figure S.6.25 Comparison of the spectrum collected from point 2, first area (green) and a reference spectrum of carnauba wax (red, collection in transmission mode)

**GLAHM40333_1st area_obverse_refl_point2**		
**Position (cm^{-1})**	**Intensity (arb. u.)**	**Band assignment**
680	1.492	Magnesium silicate, Si-O-Mg bond [13]
713	1.457	Calcium carbonate, ν_2(O-C-O) [2]
781	1.457	Quartz, Si-O [1]
876	1.491	Calcium carbonate, $\nu_4(CO_3^{-2})$ [2]
1040 *reststrahlen*	1.596	Magnesium silicate, Si-O-Si [13]
1164	1.409	Not defined
1320	1.413	Calcium oxalate, ν_s(C-O); ν(C-C) [3,4]
1419	1.578	Calcium carbonate, $\nu_3(CO_3^{2-})$ [2]
1471	1.584	Wax, (δCH_2) [12],
1540	1.643	Stearate
1576	1.646	Stearate
1652	1.603	Calcium oxalate, ν_{as}(C-O); overlapping δ(H-O-H) [3,4]
1740	sh	Wax, (νC=O) [12]
1797	1.292	Calcium carbonate, combination $\nu_1+\nu_4(CO_3^{-2})$ [5,8]
1866	1.225	Copper carbonate, combination $\nu_1+\nu_4(CO_3^{-2})$ [5,8]
1917	1.224	Wax

2024	1.168	Wax
2146	1.168	Wax
2513	1.225	Calcium carbonate, combination $\nu_1+\nu_3(CO_3^{-2})$ [5,8]
2850	1.696	Wax, ($\nu_s CH_2$) [12]
2918	1.786	Wax, ($\nu_{as} CH_2$) [12]
2955	1.555	Wax, ($\nu_{as} CH_3$) [12]
3676	1.341	Mg silicate, OH multiplet [13]

Table S.6.8 Position and intensity of the main IR bands at poin2 and their interpretation

Discussion: The above spectrum shows bands assigned to **wax**. Other organic bands are present, specifically the doublet at 1576 + 1540, and the band at 1470 cm^{-1}, which might be attributed to **calcium distearate**. Minor **carbonates** and **oxalates**. This composition is highly compatible with the species detected from spectra 1-2 in the 3rd area analysed, near Sponsian's ear. Silicates are also present, as magnesium silicate hydroxide, talc, which might have come from gloves used to handle the coins.

The second area studies in shown in Figure S.6.25:

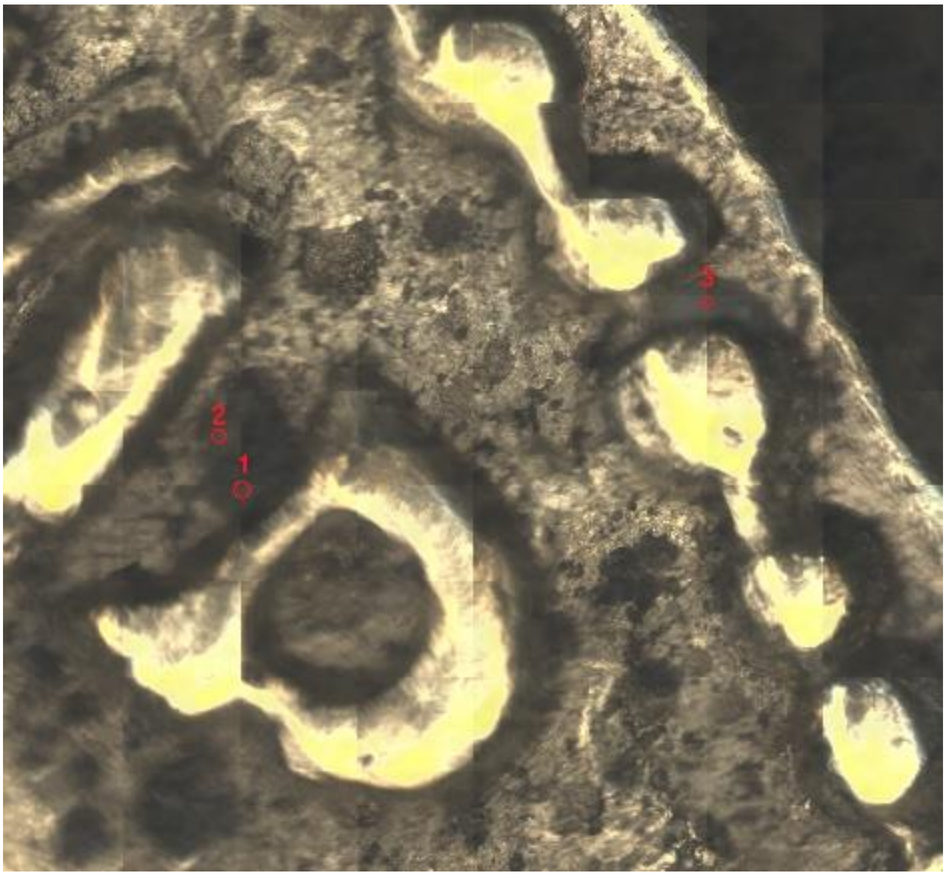

Figure S.6.26 Second area studied on Coin GLAHM:40333

Point 1 was taken in an area of bright reflectance under the light microscope (Figure S.6.26)

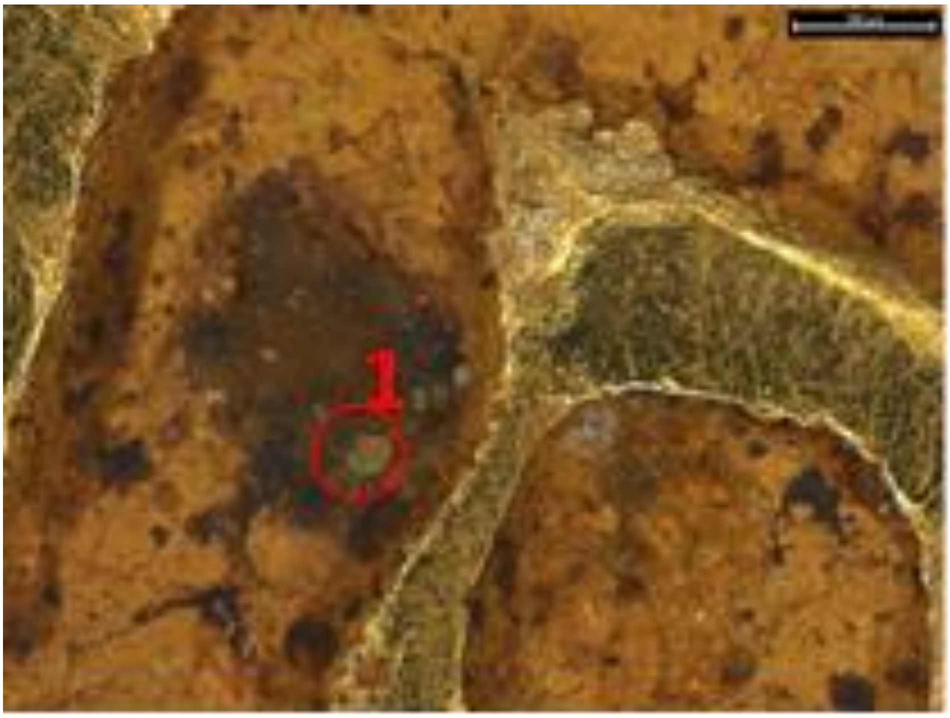

Figure S.6.27 Light microscope image of position of point 1.

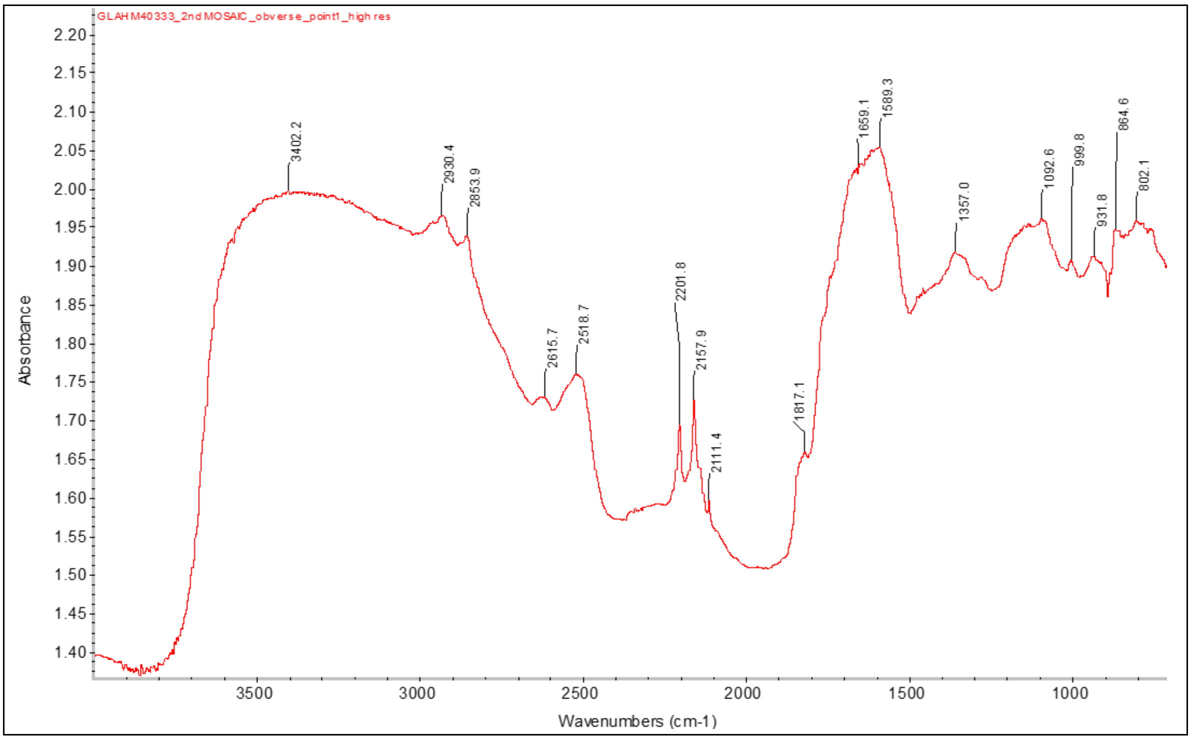

Figure S.6.28 Spectrum from point 1 in Figure S.6.25

The main absorption bands from point 1 in this area are given in Table S.6.9

**GLAHM:40333_2nd area_obverse_point1**	
**Position (cm^{-1})**	**Intensity (arb. u.)**
802.1	1.957
864.6	1.946

931.8	1.910
999.8	1.906
1092.6	1.960
1357.0	1.916
1589.3	2.051
1659.1	2.025
1817.1	1.657
2111.4	1.594
2157.9	1.724
2201.8	1.692
2518.7	1.757
2615.7	1.728
2853.9	1.937
2930.4	1.964
3402.2	1.995

Table S.6.9 Position and intensity of the main IR bands at point 1 in Figure S.6.25

Discussion: Sulphates are present in this area, possibly of the jarosite type, ($KFe_3(SO_4)_2(OH)_6$) [14], as was found for coin GLAHM:29821 (point 2, reverse). They both show similar combination modes in the region 2200-2000 cm^{-1}. However, the bands at 1193 and 1080 cm^{-1} are broader in GLAHM:40333.

Point 3 was positioned on a prominent white amorphous area that was suspected to be wax (Figure S.6.28). The spectrum is given in Figure S.6.29.

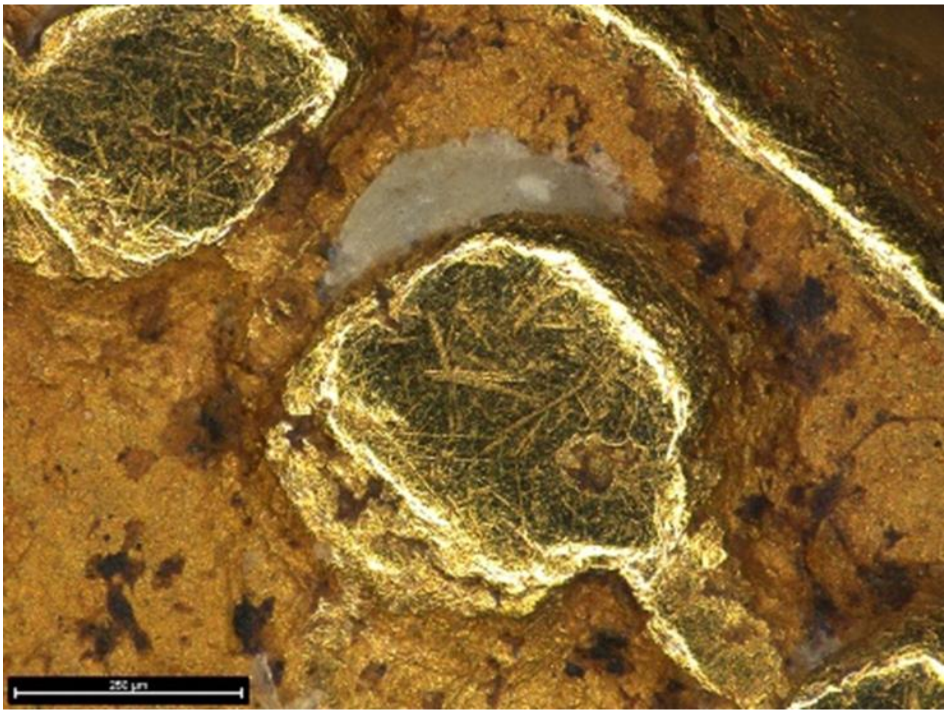

Figure S.6.29 Light microscope image showing the area of suspected wax analysed at point 3

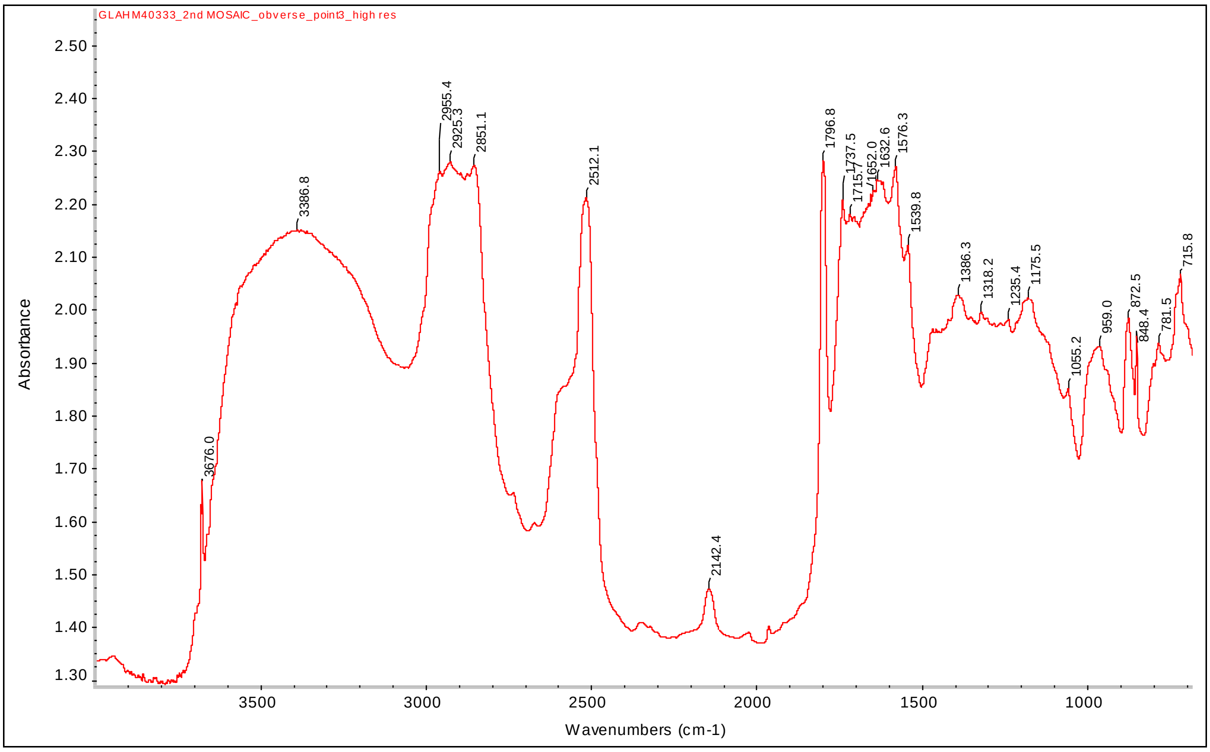

Figure S.6. 30 Spectrum collected from point 3

Discussion: The above spectrum shows absorption bands consistent with the wax reference spectrum shown in Figure 2.4.24, despite carbonate content in this sample is predominant, with minor stearate and talc.

References

[1] Saikia, B. J. & Parthasarathy, G. Fourier Transform Infrared Spectroscopic Characterization of Kaolinite from Assam and Meghalaya, Northeastern India. J. Mod. Phys. 01, 206–210 (2010).

[2] Chakrabarty, D. & Mahapatra, S. Aragonite crystals with unconventional morphologies. J. Mater. Chem. 9, 2953–2957 (1999).

[3] Monico, L., Rosi, F., Miliani, C., Daveri, A. & Brunetti, B. G. Non-invasive identification of metal-oxalate complexes on polychrome artwork surfaces by reflection mid-infrared spectroscopy. Spectrochim. Acta Part A Mol. Biomol. Spectrosc. 116, 270–280 (2013).

[4] Rosi, F. et al. Tracking Metal Oxalates and Carboxylates on Painting Surfaces by Non-invasive Reflection Mid-FTIR Spectroscopy. 173–193 (2019). doi:10.1007/978-3-319-90617-1_10

[5] Miliani, C., Rosi, F., Daveri, A. & Brunetti, B. G. Reflection infrared spectroscopy for the non-invasive in situ study of artists' pigments. Appl. Phys. A Mater. Sci. Process. 106, 295–307 (2012).

[6] Rosi, F., Daveri, A., Doherty, B., et al. On the use of overtone and combination bands for the analysis of the $CaSO_4$-H_2O system by Mid-Infrared reflection spectroscopy. App. Spectrosc. 64(8), 956-963 (2010).

[7] Manfredi, M., Barberis, E., Aceto, M. & Marengo, E. Non-invasive characterization of colorants by portable diffuse reflectance infrared Fourier transform (DRIFT) spectroscopy and chemometrics. Spectrochim. Acta - Part A Mol. Biomol. Spectrosc. 181, 171–179 (2017).

[8] Rosi, F., Cartechini, L., Sali, D. & Miliani, C. Recent trends in the application of fourier transform infrared (FT-IR) spectroscopy in Heritage Science: From micro: From non-invasive FT-IR. Phys. Sci. Rev. 4, 1–19 (2019).

[9] Steger, S., Stege, H., Bretz, S. & Hahn, O. Capabilities and limitations of handheld Diffuse Reflectance Infrared Fourier Transform Spectroscopy (DRIFTS) for the analysis of colourants and binders in 20th-century reverse paintings on glass. Spectrochim. Acta - Part A Mol. Biomol. Spectrosc. 195, 103–112 (2018).

[10] Madejová, J. FTIR techniques in clay mineral studies. Vib. Spectrosc. 31, 1–10 (2003).

[11] Lu, Y. & Miller, J. D. Carboxyl Stretching Vibrations of Spontaneously Adsorbed and LB-Transferred Calcium Carboxylates as Determined by FTIR Internal Reflection Spectroscopy. J. Colloid Interface Sci. 256, 41–52 (2002).

[12] Invernizzi, C., Daveri, A., Vagnini, M. & Malagodi, M. Non-invasive identification of organic materials in historical stringed musical instruments by reflection infrared spectroscopy: a methodological approach. Anal. Bioanal. Chem. 409, 3281–3288 (2017).

[13] Liu, X., Liu, X. & Hu, Y. Investigation of the Thermal Decomposition of Talc. Clays Clay Miner. 62, 137–144 (2014).

[14] Makreski, P., Jovanovski, G. & Dimitrovska, S. Minerals from Macedonia. Vib. Spectrosc. 39, 229–239 (2005).
